# Exploring the causal effects of epilepsy and its subtypes on anthropometric traits: A 2-sample Mendelian randomization study

**DOI:** 10.1097/MD.0000000000044619

**Published:** 2025-09-19

**Authors:** Lianhui Chen, Xiaohao Hu, Min Wu, Zhenzhong Zeng, Yongfen Wang

**Affiliations:** a Department of Pediatrics, The Second Affiliated Hospital of Fujian Medical University, Quanzhou, China; b Rare Disease Medical Center, The Second Affiliated Hospital of Fujian Medical University, Quanzhou, China.

**Keywords:** anthropometric traits, BMI, epilepsy, height, Mendelian randomization, obesity

## Abstract

Epilepsy is frequently associated with altered anthropometric outcomes, including height, weight, and body mass index (BMI). However, the causal relationship between epilepsy and these traits remains uncertain. This Mendelian randomization (MR) study aimed to investigate the causal effects of epilepsy and its subtypes on anthropometric traits, including height, weight, BMI, idiopathic short stature, constitutional tall stature, and obesity. A 2-sample MR analysis was conducted using genetic instruments derived from genome-wide association studies. Genetic instruments for epilepsy phenotypes were sourced from the European-only summary statistics of the International League Against Epilepsy Consortium on Complex Epilepsies. Outcome data were obtained from the FinnGen database and replicated in the pan-UK Biobank. The inverse-variance weighted method was used as the primary analysis, while MR-Egger regression, weighted median, and MR-PRESSO were employed to assess robustness and horizontal pleiotropy. MR analyses did not identify significant causal associations between epilepsy (including subtypes) and any anthropometric outcomes after adjusting for multiple testing. Although nominally significant associations appeared in specific analyses (e.g., focal epilepsy with weight and generalized genetic epilepsy with BMI), none survived correction. Sensitivity analyses confirmed the absence of directional pleiotropy or influential outliers. This MR study provides no evidence supporting a direct causal effect of epilepsy or its subtypes on anthropometric traits. These results indicate that observed correlations from previous studies may reflect confounding factors, such as antiepileptic medications or lifestyle, rather than epilepsy itself. Future research should investigate alternative mechanisms underlying growth and weight changes in individuals with epilepsy.

## 1. Introduction

Epilepsy, one of the most common neurological disorders globally, affects approximately 50 million individuals and is defined by recurrent spontaneous seizures.^[1]^ Beyond seizures, epilepsy is frequently accompanied by various comorbidities, including metabolic dysfunction, altered growth patterns, and obesity.^[2–4]^ A recent meta-analysis indicated that the prevalence of obesity among epilepsy patients is significantly higher compared to the general population.^[5]^ Pediatric patients with epilepsy are particularly susceptible to growth abnormalities, often manifesting as reduced height or impaired weight gain, especially when undergoing long-term treatment with antiepileptic drugs (AEDs).^[6]^

Several factors could explain how epilepsy influences anthropometric traits. Prominent among these are the adverse effects of certain AEDs like valproate (VPA), which is well-documented to induce significant weight gain and metabolic disturbances.^[7,8]^ Furthermore, lower physical activity levels associated with seizure-related anxiety or social stigma, along with various psychosocial factors, may also contribute to weight gain and obesity in epilepsy patients.^[9,10]^ Despite extensive observational evidence, it remains uncertain whether epilepsy itself directly causes these anthropometric changes. While some investigations have emphasized direct effects of epilepsy and its treatments on growth and metabolism, others argue that external environmental factors could underlie these observed associations.^[9,11]^

Mendelian randomization (MR) provides a robust methodological framework to clarify causal relationships by using genetic variants as instrumental variables.^[12]^ Utilizing genetic data from extensive genome-wide association studies (GWAS) on epilepsy and anthropometric traits, MR analyses can differentiate causal effects from mere associations. In this study, we applied a 2-sample MR approach to evaluate the causal influence of epilepsy and its primary subtypes on anthropometric measures, including height, weight, and body mass index (BMI). By investigating genetic evidence linking epilepsy directly to these outcomes, our findings may inform clinical strategies aimed at mitigating potential negative effects on growth and metabolic health in epilepsy patients.

## 2. Materials and methods

### 2.1. Study design and data sources

**Figure 1.**
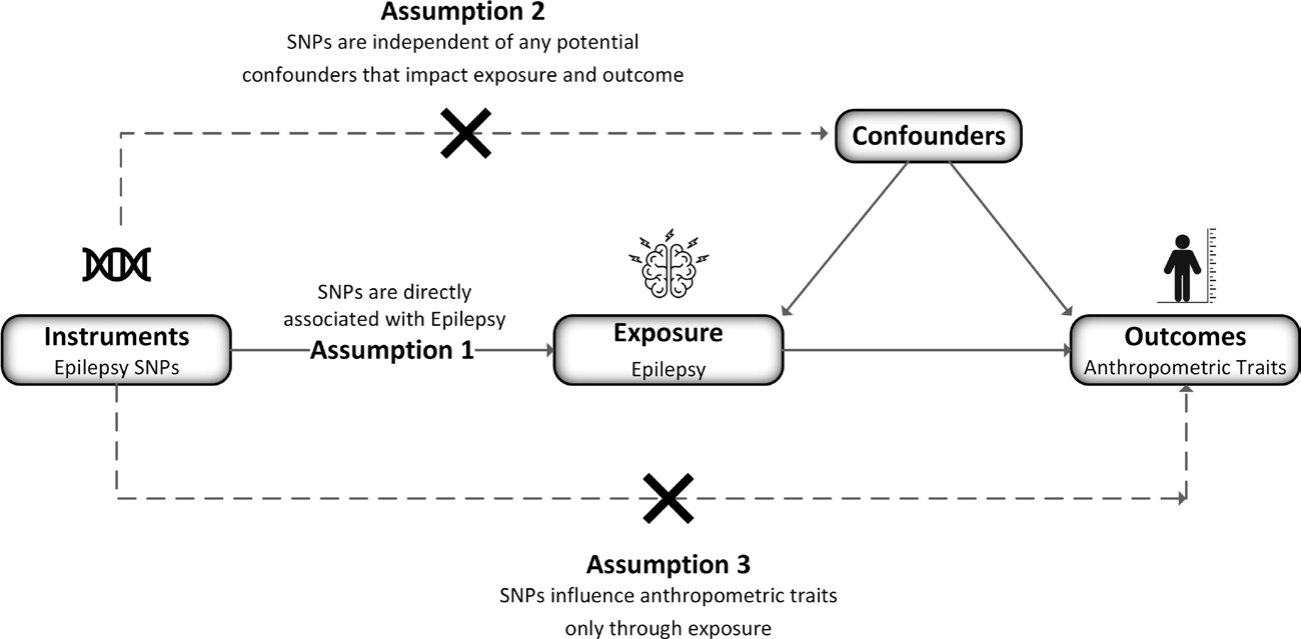
Study overview and Mendelian randomization (MR) model. Schematic diagram of the MR study illustrating the investigation of causal effects of epilepsy and its subtypes on anthropometric outcomes. The figure highlights the key assumptions of MR analysis: (1) genetic variants must strongly associate with epilepsy; (2) genetic variants are independent of confounding factors; and (3) genetic variants influence anthropometric outcomes only through epilepsy. MR = Mendelian randomization, SNP = single nucleotide polymorphism.

We conducted a 2-sample MR analysis to evaluate the potential causal influence of epilepsy and its subtypes on anthropometric outcomes, including height, weight, BMI, idiopathic short stature (ISS), constitutional tall stature (CTS), and obesity. Figure 1 illustrates the overall study design and the fundamental assumptions of MR methodology.^[13]^

Exposure data were acquired from the EpiGAD (Epilepsy Genetics Association Database), which contains European ancestry-specific summary statistics from the International League Against Epilepsy (ILAE) Consortium on Complex Epilepsies.^[14]^ The dataset comprises 3 primary epilepsy phenotypes (all epilepsy, focal epilepsy [FE], and generalized genetic epilepsy [GGE]) and 7 subphenotypes (juvenile myoclonic epilepsy [JME], juvenile absence epilepsy [JAE], childhood absence epilepsy [CAE], generalized tonic-clonic seizures alone [GTCSA], focal epilepsy with hippocampal sclerosis [FE-HS], focal epilepsy with other lesions [FE-OL], and focal epilepsy without lesions [FE-NL]). All epilepsy phenotypes in the GWAS were assigned by epilepsy specialists based on standardized clinical evaluations and classified following the 2010 ILAE criteria. Phenotypic assignments relied on EEG, MRI, and clinical history data, enabling categorization into FE, GGE, or unclassified epilepsy. Further subtyping was applied where detailed clinical information was available.^[14,15]^ Employing these data allowed us to reduce the confounding effects of population stratification, ensuring results relevant to European populations. The original GWAS applied linear mixed models (BOLT-LMM) adjusted for sex and controlled for cryptic relatedness and population structure using genetic relationship matrices.

Detailed information on the GWAS datasets utilized for MR analysis is summarized in Table S1, Supplemental Digital Content, https://links.lww.com/MD/Q43. Outcome variables were extracted from the FINNGEN database, a comprehensive genomic initiative involving over 5,00,000 Finnish biobank participants with extensive linked health records.^[16]^ All anthropometric traits analyzed in this study were drawn from FINNGEN’s latest database release (R12), except ISS and CTS, for which we used data from the earlier R9 release due to their absence in the current version. Association testing in FINNGEN was conducted using REGENIE linear mixed models, with covariates including age, sex, genotyping batch, and the top 10 principal components.

To assess the robustness of our findings, we additionally performed replication analyses using outcome data from the pan-UK Biobank (pan-UKB, https://pan.ukbb.broadinstitute.org/), which provides summary statistics for anthropometric traits.^[17]^ Due to data availability, replication analyses were limited to height, weight, BMI, and obesity, as ISS and CTS were not included in the pan-UKB dataset. All pan-UKB GWAS were conducted using models adjusted for age, sex, age × sex interaction, age², age² × sex interaction, and the top 10 genetic principal components.

The exposure and outcome GWAS datasets were both derived from large-scale European populations, minimizing bias due to population stratification. The similarity in ancestry ensures the validity of the genetic variant-exposure associations across samples. The exposure and outcome GWAS are entirely independent datasets with no known sample overlap, which helps avoid bias from overlapping samples in 2-sample MR.

### 2.2. Selection of genetic instruments for epilepsy

To identify suitable genetic instruments for MR analyses, single nucleotide polymorphisms (SNPs) strongly associated with epilepsy phenotypes were selected from EpiGAD dataset summary statistics. SNPs were primarily chosen using a genome-wide significance threshold of *P* < 5 × 10^−8^. If insufficient variants met this criterion, we relaxed the significance threshold to *P* < 5 × 10^−6^, an established practice in MR studies with limited sample sizes.^[18]^

We performed linkage disequilibrium clumping to ensure that the SNPs were independent, using an linkage disequilibrium threshold of *r*² < 0.001 within a 10 Mb window, based on the European population reference from the 1000 Genomes Project. The harmonization step aligned alleles between exposure and outcome datasets and excluded palindromic SNPs. Table S2, Supplemental Digital Content, https://links.lww.com/MD/Q44 provides detailed characteristics and summaries of the SNPs selected. The proportion of variance explained (*R²*) and *F*-statistics were calculated to assess instrument strength.^[19]^ SNPs with an *F*-statistic exceeding 10 were considered strong instruments as per standard guidelines.^[20]^ Our analysis utilized 46 SNPs for all epilepsy, 23 SNPs for GGE, 29 SNPs for FE, and additional SNPs for subtypes (JME: 36, JAE: 9, CAE: 13, GTCSA: 4, FE-HS: 13, FE-OL: 4, FE-NL: 5). All selected variants exhibited robust instrument strength, with minimum *F*-statistics of 18.4 (Table S2, Supplemental Digital Content, https://links.lww.com/MD/Q44).

### 2.3. Statistical analysis

MR analyses were performed using the TwoSampleMR package (version 2024.12.1 + 563) in R.^[12]^ The primary method of analysis was inverse-variance weighted (IVW), which combines SNP-specific causal estimates under assumptions of no pleiotropy or confounding.^[20]^ To verify result robustness and detect potential pleiotropy, additional sensitivity analyses were conducted using weighted median and MR-Egger regression methods. The weighted median provides reliable causal estimates even with up to 50% invalid instruments, whereas MR-Egger regression can adjust for directional pleiotropy and provide unbiased effect estimates.^[21,22]^

Further evaluation of pleiotropy involved the MR-Egger intercept test and Cochran’s *Q* statistic for heterogeneity.^[23,24]^ To validate the stability of the results, leave-one-out analyses were performed, and MR-PRESSO was used to identify and correct influential outlier SNPs that might distort causal estimates.^[25,26]^

Given the multiple hypotheses tested (10 exposures × 6 outcomes), the Benjamini–Hochberg method was implemented to adjust for multiple comparisons and control the false discovery rate. An adjusted *P*-value (false discovery rate *q*-value) threshold of <.05 was considered statistically significant.

## 3. Results

### 3.1. Primary analyses of epilepsy on height, weight, and BMI

**Figure 2.**
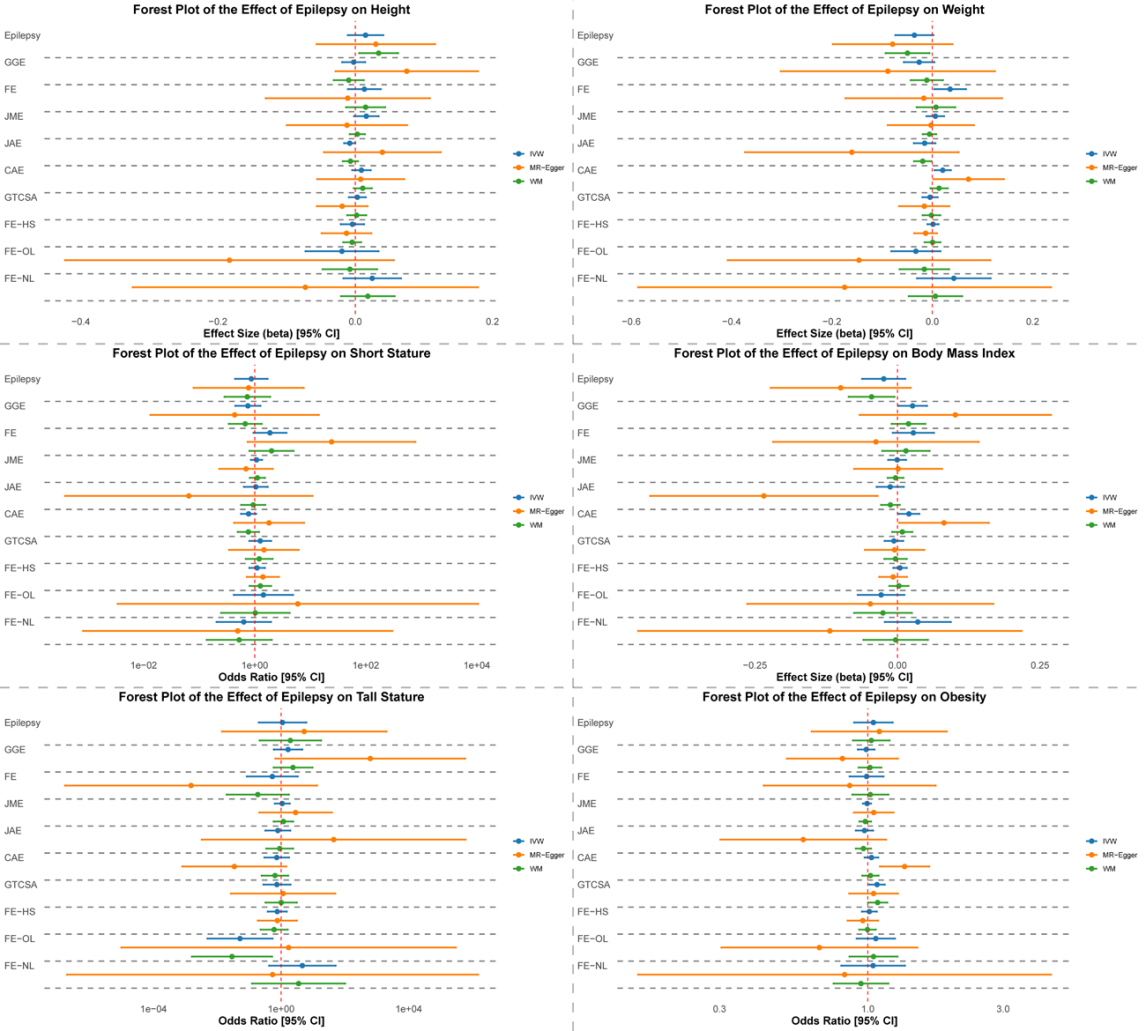
Forest plot of the effect of epilepsy on outcomes using FinnGen data. Summary forest plots displaying causal estimates derived from Mendelian randomization analyses. The plots illustrate the associations between epilepsy phenotypes (including “all epilepsy,” generalized genetic epilepsy [GGE], focal epilepsy [FE], and 7 epilepsy subtypes) and anthropometric traits (height, weight, body mass index [BMI], short stature, tall stature, and obesity). Three MR methods are presented: inverse-variance weighted (IVW), weighted median (WM), and MR-Egger regression. The epilepsy subtypes shown include juvenile myoclonic epilepsy (JME), juvenile absence epilepsy (JAE), childhood absence epilepsy (CAE), generalized tonic-clonic seizures alone (GTCSA), focal epilepsy with hippocampal sclerosis (FE-HS), focal epilepsy with other lesions (FE-OL), and focal epilepsy without lesions (FE-NL). Effect estimates are shown with 95% confidence intervals; vertical lines indicate the null effect. BMI = body mass index, CAE = childhood absence epilepsy, CI = confidence intervals, FE = focal epilepsy , FE-HS = focal epilepsy with hippocampal sclerosis, FE-OL = focal epilepsy with other lesions, FE-NL = focal epilepsy without lesions, GGE = generalized genetic epilepsy, GTCSA = generalized tonic-clonic seizures alone, IVW = inverse-variance weighted, JAE = juvenile absence epilepsy, JME = juvenile myoclonic epilepsy, MR = Mendelian randomization, WM = weighted median.

The MR estimates for the causal influence of “all epilepsy,” GGE, and FE on height, weight, and BMI are summarized in Table 1. After correcting for multiple testing using the Benjamini–Hochberg method, no statistically significant causal relationships were identified. Although nominally significant associations were initially detected, such as FE on weight (β = 0.036, *P* = .028 via IVW) and GGE on BMI (β = 0.026, *P* = .040 via IVW), these findings lost significance after correction. Additionally, sensitivity analyses using MR-Egger and weighted median methods produced similar nonsignificant results without any clear or consistent direction of effects, as illustrated in Figure 2.

**Table 1:** Mendelian randomization estimates of the causal effect of epilepsy on anthropometric traits using FinnGen data.

Method	Number of SNPs	MR analysis			Heterogeneity test			*P* of MR-Egger intercept
		β	SE	*P*	Cochran’s *Q*	*I^2^*	*P*	
Epilepsy (N = 64,629, Ncase = 27,559, Ncontrol = 42,436) on height (N = 3,64,629)								
IVW	33	0.015	0.013	.257	60.8	47.4	.002	–
MR-Egger	33	0.030	0.044	0.501	60.6	48.8	.001	.724
Weighted Median	33	0.034	0.014	.018	–	–	–	–
Epilepsy (N = 64,629, Ncase = 27,559, Ncontrol = 42,436) on weight (N = 3,70,087)								
IVW	32	−0.036	0.019	.066	69.4	55.3	.000	–
MR-Egger	32	−0.079	0.061	.203	68.1	56.0	.000	.457
Weighted Median	32	−0.049	0.022	0.027	–	–	–	–
Epilepsy (N = 64,629, Ncase = 27,559, Ncontrol = 42,436) on BMI (N = 3,62,327)								
IVW	36	−0.024	0.019	.214	76.7	54.4	.000	–
MR-Egger	36	−0.100	0.063	.122	73.3	53.6	.000	.214
Weighted Median	36	−0.045	0.021	.027	–	–	–	–
GGE (N = 49,388, Ncase = 6952, Ncontrol = 42,436) on height (N = 3,64,629)								
IVW	13	−0.002	0.009	.789	15.1	20.6	.236	–
MR-Egger	13	0.075	0.053	.185	12.6	12.8	.320	.168
Weighted Median	13	−0.009	0.011	.395	–	–	–	–
GGE (N = 49,388, Ncase = 6952, Ncontrol = 42,436) on weight (N = 3,70,087)								
IVW	11	−0.026	0.016	.094	20.8	51.9	.023	–
MR-Egger	11	−0.089	0.109	.437	20.0	55.1	.018	.577
Weighted Median	11	−0.011	0.016	.507	–	–	–	–
GGE (N = 49,388, Ncase = 6952, Ncontrol = 42,436) on BMI (N = 362,327)								
IVW	14	0.026	0.013	.040	20.4	36.1	.087	–
MR-Egger	14	0.101	0.086	.259	19.1	37.2	.086	.393
Weighted Median	14	0.019	0.015	.201	–	–	–	–
FE (N = 57,375, Ncase = 14,939, Ncontrol = 42,436) on height (N = 3,64,629)								
IVW	27	0.013	0.012	.279	44.8	41.9	.012	–
MR-Egger	27	−0.011	0.061	.859	44.5	43.8	.010	.688
Weighted Median	27	0.015	0.015	.3	–	–	–	–
FE (N = 57,375, Ncase = 14,939, Ncontrol = 42,436) on weight (N = 3,70,087)								
IVW	25	0.036	0.016	.028	38.7	38.0	.029	–
MR-Egger	25	−0.017	0.080	.833	38.0	39.4	.026	.506
Weighted Median	25	0.007	0.020	.706	–	–	–	–
FE (N = 57,375, Ncase = 14,939, Ncontrol = 42,436) on BMI (N = 3,62,327)								
IVW	26	0.028	0.018	.132	47.6	47.5	.004	–
MR-Egger	26	−0.038	0.092	.684	46.6	48.5	.004	.473
Weighted Median	26	0.015	0.021	.478	–	–	–	–

BMI = body mass index, FE = focal epilepsy, GGE = generalized genetic epilepsy, LCI = lower confidence interval, IVW = inverse-variance weighted, MR = Mendelian randomization, SE = standard error, SNP = single nucleotide polymorphism, UCI = upper confidence interval.

In the “all epilepsy” group, point estimates for height, weight, and BMI were either slightly positive or slightly negative (β = 0.015 for height; β = −0.036 for weight; β* *= −0.024 for BMI using IVW), but none met the significance threshold. GGE showed borderline significance for BMI in the IVW analysis (β = 0.026, *P* = .040), whereas FE showed a nominally significant effect on weight (β = 0.036, *P* = .028). Both signals lost significance after Benjamini–Hochberg adjustment.

### 3.2. Analyses of epilepsy on ISS, CTS, and obesity

Table 2 displays the MR findings for the causal effects of “all epilepsy,” GGE, and FE on ISS, CTS, and obesity. None of these analyses yielded statistically significant outcomes, even before correcting for multiple tests. For example, “all epilepsy” showed no significant association with ISS (OR = 0.87; *P* = .682) or obesity (OR = 1.05; *P* = .569). Further subgroup analyses examining GGE and FE also revealed nonsignificant associations across all evaluated anthropometric outcomes. Sensitivity analyses utilizing MR-Egger and weighted median methods produced consistent results without highlighting any noteworthy trends.

**Table 2:** Mendelian randomization estimates of the causal effect of epilepsy on stature and obesity using FinnGen data.

Method	Number of SNPs	MR analysis				Heterogeneity test			MR-Egger intercept *P*
		OR	LCI	UCI	*P*	Cochran’s *Q*	*I^2^*	*P*	
Epilepsy (N = 64,629, Ncase = 27,559, Ncontrol = 42,436) on ISS (N = 3,62,599, Ncase = 6,11, Ncontrol = 3,61,988)									
IVW	39	0.87	0.44	1.70	.682	31.7	0.0	.757	–
MR-Egger	39	0.77	0.08	7.61	.828	31.6	0.0	.718	.918
Weighted Median	39	0.74	0.29	1.89	.516	–	–	–	–
Epilepsy (N = 64,629, Ncase = 27,559, Ncontrol = 42,436) on CTS (N = 3,62,116, Ncase = 128, Ncontrol = 3,61,988)									
IVW	39	1.11	0.20	6.19	.905	55.5	31.5	.033	–
MR-Egger	39	5.30	0.01	2014.75	.585	55.0	32.8	.028	.593
Weighted Median	39	1.96	0.21	18.08	.540	–	–	–	–
Epilepsy (N = 64,629, Ncase = 27,559, Ncontrol = 42,436) on Obesity (N = 500,192, Ncase = 31,499, Ncontrol = 4,68,693)									
IVW	41	1.05	0.89	1.22	.569	119.4	66.5	.000	–
MR-Egger	41	1.10	0.63	1.90	.740	119.3	67.3	.000	.859
Weighted Median	41	1.03	0.89	1.19	.706	–	–	–	–
GGE (N = 49,388, Ncase = 6952, Ncontrol = 42,436) on ISS (N = 3,62,599, Ncase = 611, Ncontrol = 3,61,988)									
IVW	18	0.75	0.45	1.27	.286	19.4	12.4	.306	–
MR-Egger	18	0.43	0.01	14.31	.647	19.3	17.0	.254	.758
Weighted Median	18	0.67	0.34	1.34	.256	–	–	–	–
GGE (N = 49,388, Ncase = 6952, Ncontrol = 42,436) on CTS (N = 3,62,116, Ncase = 128, Ncontrol = 3,61,988)									
IVW	18	1.66	0.59	4.66	.336	12.2	0.0	.790	–
MR-Egger	18	627.74	0.66	5,98,000.00	.084	9.2	0.0	.904	.105
Weighted Median	18	2.37	0.58	9.63	.227	–	–	–	–
GGE (N = 49,388, Ncase = 6952, Ncontrol = 42,436) on Obesity (N = 5,00,192, Ncase = 31,499, Ncontrol = 4,68,693)									
IVW	18	0.99	0.92	1.06	.708	16.5	0.0	.490	–
MR-Egger	18	0.81	0.52	1.28	.384	15.8	0.0	.469	.409
Weighted Median	18	1.02	0.93	1.12	.706	–	–	–	–
FE (N = 57,375, Ncase = 14,939, Ncontrol = 42,436) on ISS (N = 3,62,599, Ncase = 611, Ncontrol = 3,61,988)									
IVW	28	1.88	0.94	3.75	.073	24.5	0.0	.603	–
MR-Egger	28	24.22	0.75	783.34	.084	22.3	0.0	.670	.154
Weighted Median	28	2.01	0.80	5.03	.154	–	–	–	–
FE (N = 57,375, Ncase = 14,939, Ncontrol = 42,436) on CTS (N = 3,62,116, Ncase = 128, Ncontrol = 3,61,988)									
IVW	28	0.53	0.08	3.36	.502	42.4	36.3	.030	–
MR-Egger	28	0.00	0.00	13.41	.173	39.9	34.8	.040	.208
Weighted Median	28	0.19	0.02	1.76	.142	–	–	–	–
FE (N = 57,375, Ncase = 14,939, Ncontrol = 42,436) on Obesity (N = 5,00,192, Ncase = 31,499, Ncontrol = 4,68,693)									
IVW	28	0.99	0.86	1.14	.888	55.3	51.2	.001	–
MR-Egger	28	0.86	0.43	1.73	.682	55.0	52.7	.001	.696
Weighted Median	28	1.02	0.88	1.18	.782	–	–	–	–

CTS = constitutional tall stature, FE = focal epilepsy, GGE = generalized genetic epilepsy, ISS = idiopathic short stature, LCI = lower confidence interval, MR = Mendelian randomization, OR = odds ratio, IVW = inverse-variance weighted, SNP = single nucleotide polymorphism, UCI = upper confidence interval.

### 3.3. Subphenotype analyses

Additional MR analyses involving 7 epilepsy subphenotypes and anthropometric traits are provided in Tables S3 and S4, Supplemental Digital Content, https://links.lww.com/MD/Q43. Following correction for multiple comparisons, none of the epilepsy subtypes demonstrated robust causal relationships with any anthropometric traits. Though certain analyses produced nominally significant results (for example, CAE on weight via IVW, *P* = .013), these associations were no longer statistically significant after applying Benjamini–Hochberg correction. Similarly, sensitivity tests did not reveal any meaningful or consistent relationships.

### 3.4. Pleiotropy and sensitivity analyses

Evaluation of horizontal pleiotropy through the MR-Egger intercept generally indicated nonsignificant results across all analyses, except for CAE on obesity (*P* = .022). Cochran’s *Q* tests revealed moderate-to-high heterogeneity across some analyses (*I*² ranging between 50% and 89.1%), prompting the use of random-effects IVW models. Nevertheless, this approach did not alter the overall null conclusions. Additionally, MR-PRESSO analyses identified and adjusted for a limited number of outlier SNPs; however, their removal did not impact the primary inference of nonsignificant causal effects between epilepsy and anthropometric outcomes. Leave-one-out analyses (Fig. S1, Supplemental Digital Content, https://links.lww.com/MD/Q45) further confirmed result stability, showing no undue influence from any single genetic instrument.

### 3.5. Replication analyses using pan-UKB data

**Figure 3.**
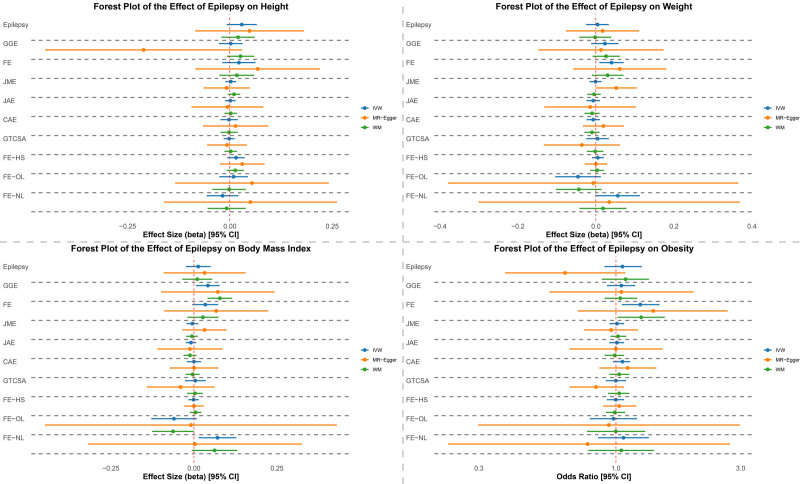
Forest plot of the effect of epilepsy on outcomes using pan-UKB data. Summary forest plots displaying causal estimates derived from Mendelian randomization analyses. The plots illustrate the associations between epilepsy phenotypes (including “all epilepsy,” generalized genetic epilepsy [GGE], focal epilepsy [FE], and 7 epilepsy subtypes) and anthropometric traits (height, weight, body mass index [BMI], and obesity). Three MR methods are presented: inverse-variance weighted (IVW), weighted median (WM), and MR-Egger regression. The epilepsy subtypes shown include juvenile myoclonic epilepsy (JME), juvenile absence epilepsy (JAE), childhood absence epilepsy (CAE), generalized tonic-clonic seizures alone (GTCSA), focal epilepsy with hippocampal sclerosis (FE-HS), focal epilepsy with other lesions (FE-OL), and focal epilepsy without lesions (FE-NL). Effect estimates are shown with 95% confidence intervals; vertical lines indicate the null effect. BMI = body mass index, CAE = childhood absence epilepsy, CI = confidence intervals, FE = focal epilepsy , FE-HS = focal epilepsy with hippocampal sclerosis, FE-OL = focal epilepsy with other lesions, FE-NL = focal epilepsy without lesions, GGE = generalized genetic epilepsy, GTCSA = generalized tonic-clonic seizures alone, IVW = inverse-variance weighted, JAE = juvenile absence epilepsy, JME = juvenile myoclonic epilepsy, MR = Mendelian randomization, WM = weighted median.

To validate the primary findings, we conducted replication analyses using outcome data from the pan-UK Biobank for height, weight, BMI, and obesity (Fig. 3; Tables 3 and 4, S5 and S6, Supplemental Digital Content, https://links.lww.com/MD/Q43). Overall, the results from pan-UKB were largely consistent with the FinnGen-based analyses, showing no strong evidence of causal associations between most epilepsy phenotypes and anthropometric traits.

**Table 3:** Mendelian randomization estimates of the causal effect of epilepsy on anthropometric traits using pan-UKB data.

Method	Number of SNPs	MR analysis			Heterogeneity test			*P* of MR-Egger intercept
		β	SE	*P*	Cochran’s *Q*	*I^2^*	*P*	
Epilepsy (N = 64,629, Ncase = 27,559, Ncontrol = 42,436) on height (N = 4,19,596)								
IVW	34	0.029	0.018	.109	69.1	53.7	.000	.761
MR-Egger	34	0.048	0.067	.475	69.3	52.4	.000	–
Weighted Median	34	0.020	0.020	.319	–	–	–	–
Epilepsy (N = 64,629, Ncase = 27,559, Ncontrol = 42,436) on weight (N = 4,19,316)								
IVW	35	0.004	0.014	.779	34.9	5.5	.377	–
MR-Egger	35	0.017	0.047	.725	35.0	2.9	.420	.777
Weighted Median	35	-0.002	0.020	.904	–	–	–	–
Epilepsy (N = 64,629, Ncase = 27,559, Ncontrol = 42,436) on BMI (N = 4,19,163)								
IVW	37	0.013	0.018	0.472	55.2	36.6	.016	–
MR-Egger	37	0.032	0.062	0.616	55.4	35.0	.020	.759
Weighted Median	37	0.010	0.022	0.646	–	–	–	–
GGE (N = 49,388, Ncase = 6952, Ncontrol = 42,436) on height (N = 4,19,596)								
IVW	13	0.002	0.014	.902	21.6	49.1	.028	–
MR-Egger	13	−0.211	0.122	.112	27.6	56.6	.006	.107
Weighted Median	13	0.026	0.016	.089	–	–	–	–
GGE (N = 49,388, Ncase = 6952, Ncontrol = 42,436) on weight (N = 4,19,316)								
IVW	16	0.023	0.017	.172	43.2	67.6	.000	–
MR-Egger	16	0.013	0.081	.877	43.3	65.4	.000	.903
Weighted Median	16	0.026	0.017	.122	–	–	–	–
GGE (N = 49,388, Ncase = 6952, Ncontrol = 42,436) on BMI (N = 4,19,163)								
IVW	15	0.042	0.017	.015	33.1	60.8	.002	–
MR-Egger	15	0.072	0.086	.418	33.5	58.2	.002	.731
Weighted Median	15	0.078	0.018	.000	–	–	–	–
FE (N = 57,375, Ncase = 14,939, Ncontrol = 42,436) on height (N = 4,19,596)								
IVW	22	0.022	0.020	.270	47.6	58.0	.000	–
MR-Egger	22	0.068	0.077	.390	48.5	56.7	.001	.544
Weighted Median	22	0.017	0.021	.413	–	–	–	–
FE (N = 57,375, Ncase = 14,939, Ncontrol = 42,436) on weight (N = 4,19,316)								
IVW	25	0.040	0.015	.008	27.7	17.1	.226	–
MR-Egger	25	0.061	0.060	.323	27.9	14.0	.264	.724
Weighted Median	25	0.030	0.020	0140	–	–	–	–
FE (N = 57,375, Ncase = 14,939, Ncontrol = 42,436) on BMI (N = 4,19,163)								
IVW	27	0.034	0.019	.082	43.5	42.5	.012	–
MR-Egger	27	0.067	0.079	.405	43.8	40.7	.016	.667
Weighted Median	27	0.027	0.023	.232	–	–	–	–

BMI = body mass index, FE = focal epilepsy, GGE = generalized genetic epilepsy, IVW = inverse-variance weighted, MR = Mendelian randomization, SE = standard error, SNP = single nucleotide polymorphism.

**Table 4:** Mendelian randomization estimates of the causal effect of epilepsy on obesity using pan-UKB data.

Method	Number of SNPs	MR analysis				Heterogeneity test			MR-Egger intercept *P*
		OR	LCI	UCI	*P*	Cochran’s *Q*	*I^2^*	*P*	
Epilepsy (N = 64,629, Ncase = 27,559, Ncontrol = 42,436) on obesity (N = 4,20,531, Ncase = 15,917, Ncontrol = 4,04,614)									
IVW	40	1.06	0.91	1.25	.441	52.4	27.4	.061	–
MR-Egger	40	0.64	0.38	1.08	.106	57.8	32.5	.027	.055
Weighted Median	40	1.09	0.89	1.33	.394	–	–	–	–
GGE (N = 49,388, Ncase = 6952, Ncontrol = 42,436) on obesity (N = 4,20,531, Ncase = 15,917, Ncontrol = 4,04,614)									
IVW	19	1.05	0.93	1.18	.445	30.8	44.9	.021	–
MR-Egger	19	1.05	0.56	1.97	.872	30.8	41.6	.030	.985
Weighted Median	19	1.04	0.91	1.20	.570	–	–	–	–
FE (N = 57,375, Ncase = 14,939, Ncontrol = 42,436) on obesity (N = 4,20,531, Ncase = 15,917, Ncontrol = 4,04,614)									
IVW	28	1.24	1.06	1.46	.008	37.4	30.6	.068	–
MR-Egger	28	1.39	0.72	2.66	.337	37.6	28.2	.084	.734
Weighted Median	28	1.25	1.02	1.53	.030	–	–	–	–

FE = focal epilepsy, GGE = generalized genetic epilepsy, IVW = inverse-variance weighted, LCI = lower confidence interval, MR = Mendelian randomization, OR = odds ratio, SNP = single nucleotide polymorphism, UCI = upper confidence interval.

Notably, FE demonstrated nominally significant associations with both increased weight (β = 0.040, *P* = .008) and higher obesity risk (OR = 1.24, *P* = .008) using the IVW method. While the weight association was directionally consistent with the FinnGen findings (β = 0.036, *P* = .028), the obesity signal was not supported by the original dataset (OR = 0.99, *P *= .888). Both associations did not remain significant after multiple testing correction.

Additionally, GGE showed a nominally significant association with higher BMI (β = 0.042, *P* = .015), in line with the FinnGen estimate (β = 0.026, *P* = .040). FE-NL also exhibited positive associations with weight (β = 0.056, *P* = .048) and BMI (β = 0.071, *P* = .011), though these results were not statistically significant in the FinnGen dataset.

## 4. Discussion

In this study, we applied MR methods to evaluate whether epilepsy or its major subtypes exert causal effects on key anthropometric outcomes, including height, weight, BMI, ISS, CTS, and obesity. Despite an extensive exploration of these associations using genetically predicted epilepsy phenotypes, our analysis provided no robust evidence supporting direct causal relationships. Specifically, neither the primary epilepsy phenotypes (“all epilepsy,” GGE, and FE) nor the 7 subphenotypes showed significant effects on anthropometric traits after correcting for multiple testing. Replication analyses using pan-UKB data yielded largely consistent results. These findings challenge the hypothesis that epilepsy itself contributes directly to variations in growth and obesity at the population level.

Previous observational studies frequently reported associations between epilepsy and altered anthropometric traits, particularly within pediatric populations. Growth disturbances in children with epilepsy have commonly been attributed to endocrinological alterations involving growth hormone and insulin-like growth factor, or to side effects from long-term AEDs treatment, particularly VPA-induced obesity.^[27–29]^ Additionally, Daniels et al previously noted that obesity often emerges as a comorbidity in newly diagnosed, untreated pediatric epilepsy patients.^[30]^ However, observational designs in prior research inherently risk confounding from environmental, behavioral, and medication-related factors, limiting conclusions regarding direct causality.^[9–11,31]^

Interestingly, our uncorrected results indicated nominal associations between certain epilepsy phenotypes and anthropometric traits. For instance, GGE was nominally associated with BMI, and FE exhibited a suggestive association with weight. However, these preliminary findings did not persist after adjustment using the Benjamini–Hochberg method. Such outcomes underscore the necessity of controlling for multiple comparisons to prevent false-positive inferences in genetic epidemiological studies. Moreover, the consistency of null results across various sensitivity analyses, including weighted median^[9,10]^and MR-Egger methods, as well as replication analyses using pan-UKB data, reinforces the interpretation that epilepsy alone likely does not exert meaningful direct effects on these growth-related traits.

The absence of significant causal effects observed in our MR analyses suggests that the correlations previously noted between epilepsy and anthropometric outcomes in observational studies likely arise from residual confounding. Factors such as prolonged use of AEDs (particularly VPA), which is known to induce weight gain, insulin resistance, and metabolic syndrome, may substantially contribute to these associations.^[7,8,32]^ Moreover, psychosocial stressors, depression, and endocrine dysregulation (e.g., altered growth hormone and leptin signaling) have been implicated in growth and metabolic abnormalities in individuals with epilepsy.^[6,27]^ In pediatric populations, low physical activity, inadequate dietary support, and socioeconomic adversity, including low parental education, poverty, and unmet medical needs, further increase the risk of obesity.^[9,10]^ These findings underscore the importance of considering multilevel nongenetic factors when interpreting observational associations. Our MR-based findings emphasize the critical distinction between correlation and causation, and highlight the need for comprehensive management strategies that address both biological and environmental contributors to adverse metabolic profiles in epilepsy.

Further MR analysis of epilepsy subtypes also failed to yield significant causal relationships with anthropometric traits after correcting for multiple testing. Although isolated nominal associations, such as CAE on weight and BMI, emerged initially, these results became nonsignificant upon multiple-test corrections. This further supports the conclusion that epilepsy subtypes, despite differing pathophysiology or onset patterns, may not independently impact anthropometric outcomes directly, at least not to a measurable degree within the power constraints of current GWAS datasets. It is conceivable that subtle, subtype-specific effects on growth could exist but would necessitate even larger sample sizes or alternative analytic strategies to detect reliably.

Our study offers important insights into the complex genetic relationship between epilepsy and growth-related metrics. A principal strength of our investigation lies in the use of MR methodology, which leverages genetic variants as instrumental variables to reduce confounding and reverse causality, thus providing more reliable evidence regarding causation. Additionally, our use of comprehensive and robust GWAS datasets from both the EpiGAD and FINNGEN consortia, as well as the pan-UK Biobank for replication analyses, ensures high-quality genetic instruments and outcome measures. Nevertheless, MR studies have inherent limitations. One important assumption underlying MR is that the selected genetic variants affect the outcomes solely through their relationship with the exposure. While our sensitivity analyses, including MR-Egger intercept tests and MR-PRESSO, did not suggest significant pleiotropic effects or violations of this assumption, MR cannot fully exclude biases arising from unmeasured confounders or gene-environment interactions.

Finally, our findings do not eliminate the possibility of indirect mechanisms by which epilepsy might influence growth or obesity. Factors such as AEDs exposure, seizure-related behavioral changes, and comorbid psychiatric or metabolic conditions may significantly affect anthropometric outcomes independent of epilepsy itself. Thus, the present results emphasize the complexity inherent in dissecting the interplay of genetic susceptibility, environment, and clinical management in epilepsy-associated growth disturbances.

In conclusion, this MR analysis provides no evidence supporting direct causal effects of epilepsy or its subtypes on height, weight, BMI, or obesity. Rather, associations previously reported in observational studies likely reflect confounding influences such as AEDs use, lifestyle behaviors, or psychosocial and medical comorbidities. Future research, including studies utilizing larger-scale datasets, detailed clinical phenotyping, and molecular analyses of relevant genetic pathways, is necessary to further clarify the relationship between epilepsy, its treatments, and anthropometric outcomes.

## Acknowledgments

The authors would like to acknowledge the EpiGAD for providing the exposure data from the ILAE consortium on complex epilepsies, and the FINNGEN study and pan-UK Biobank for contributing the outcome data used in this study. We also extend our thanks to all the genetic consortia for making their GWAS summary data publicly available, which was instrumental in the completion of this research.

## Author contributions

**Conceptualization:** Lianhui Chen, Yongfen Wang.

**Methodology:** Xiaohao Hu, Min Wu, Zhenzhong Zeng.

**Software:** Min Wu, Zhenzhong Zeng.

**Supervision:** Yongfen Wang.

**Validation:** Xiaohao Hu.

**Writing – original draft:** Lianhui Chen.

**Writing – review & editing:** Xiaohao Hu.

## Supplementary Material


