# Supplementary material for: Exploring the causal effects of epilepsy and its subtypes on anthropometric traits: A 2-sample Mendelian randomization study
Source: Medicine (Baltimore). 2025 Sep 19;104(38):e44619. doi: 10.1097/MD.0000000000044619 (PMC12459504; doi:10.1097/MD.0000000000044619)
Supplement: Supplementary file 1 [file medi-104-e44619-s001.docx]

Table S1. Data source of genome-wide association studies included in the Mendelian randomization analysis

| Category | Phenotype | Sources | Sample size | SNPs | Cases | Controls | Population |
| --- | --- | --- | --- | --- | --- | --- | --- |
| Exposures | Epilepsy | ILAE Consortium | 69,995 | 4,880,197 | 27,559 | 42,436 | European Ancestry |
|  | GGE | ILAE Consortium | 49,388 | 4,866,064 | 6,952 | 42,436 | European Ancestry |
|  | FE | ILAE Consortium | 57,375 | 4,861,640 | 14,939 | 42,436 | European Ancestry |
|  | JME | ILAE Consortium | 44,168 | 4,983,224 | 1,732 | 42,436 | European Ancestry |
|  | JAE | ILAE Consortium | 43,098 | 4,986,339 | 662 | 42,436 | European Ancestry |
|  | CAE | ILAE Consortium | 43,485 | 4,979,764 | 1,049 | 42,436 | European Ancestry |
|  | GTCSA | ILAE Consortium | 42,921 | 4,974,437 | 485 | 42,436 | European Ancestry |
|  | FE with hippocampal sclerosis | ILAE Consortium | 43,696 | 4,819,099 | 1,260 | 42,436 | European Ancestry |
|  | FE with other lesions | ILAE Consortium | 46,649 | 4,901,535 | 4,213 | 42,436 | European Ancestry |
|  | FE without lesions | ILAE Consortium | 48,214 | 4,970,383 | 5,778 | 42,436 | European Ancestry |
| Outcomes | Body Height | FINNGEN R12 | 364,629 | 21,324,955 | - | - | European Ancestry |
|  | Body Weight | FINNGEN R12 | 370,087 | 21,325,087 | - | - | European Ancestry |
|  | Body-mass index | FINNGEN R12 | 362,327 | 21,324,896 | - | - | European Ancestry |
|  | Obesity | FINNGEN R12 | 500,192 | 21,327,062 | 31,499 | 468,693 | European Ancestry |
|  | ISS* | FINNGEN R9 | 362,599 | 20,175,454 | 611 | 361,988 | European Ancestry |
|  | CTS^#^ | FINNGEN R9 | 362,116 | 20,169,902 | 128 | 361,988 | European Ancestry |
|  | Body Height | Pan-UKB | 419,596 | 28,987,534 | - | - | European Ancestry |
|  | Body Weight | Pan-UKB | 419,316 | 28,987,534 | - | - | European Ancestry |
|  | Body-mass index | Pan-UKB | 419,163 | 28,987,534 | - | - | European Ancestry |
|  | Obesity | Pan-UKB | 420,531 | 28,987,534 | 15,917 | 404,614 | European Ancestry |

GGE: genetic generalized epilepsy; FE: focal epilepsy; JME: juvenile myoclonic epilepsy; JAE: juvenile absence epilepsy; CAE: childhood absence epilepsy; GTCSA: generalized tonic-clonic seizures alone; ILAE: the International League against Epilepsy.

* ISS: idiopathic short stature (short stature, not elsewhere classified, ICD10: E34.3) refers to a height more than two standard deviations below the mean height for age, sex, and ethnicity, without an identifiable pathological cause such as endocrine disorders, chromosomal abnormalities, nutritional deficiencies, or metabolic diseases; ^#^ CTS: Constitutional tall stature (ICD10: E34.4) defined as a height more than two standard deviations above the mean height for age, sex, and ethnicity, without an identifiable pathological cause such as endocrine disorders, chromosomal abnormalities, or genetic syndromes.

Table S3. Mendelian Randomization Estimates of the Causal Effect of 7 Epilepsy Subtypes on Anthropometric Traits using FinnGen Data

| Method | NO. of SNPs | MR analysis | | | Heterogeneity Test | | | *p* of MR-Egger intercept |
| --- | --- | --- | --- | --- | --- | --- | --- | --- |
|  |  | *β* | *se* | *p* | Cochran’s *Q* | *I^2^* | *p* |  |
| JME (N = 44,168, Ncase = 1,732, Ncontrol = 42,436) on Height (N = 364,629) | | | | | | | | |
| IVW | 36 | 0.016 | 0.009 | 0.081 | 315.0 | 88.9 | 0.000 | - |
| MR-Egger | 36 | -0.012 | 0.045 | 0.789 | 311.2 | 89.1 | 0.000 | 0.524 |
| Weighted Median | 36 | 0.003 | 0.006 | 0.591 | - | - | - | - |
| JME (N = 44,168, Ncase = 1,732, Ncontrol = 42,436) on Weight (N = 370,087) | | | | | | | | |
| IVW | 36 | 0.006 | 0.009 | 0.499 | 174.0 | 79.9 | 0.000 | - |
| MR-Egger | 36 | -0.003 | 0.044 | 0.950 | 173.8 | 80.4 | 0.000 | 0.838 |
| Weighted Median | 36 | -0.006 | 0.007 | 0.420 | - | - | - | - |
| JME (N = 44,168, Ncase = 1,732, Ncontrol = 42,436) on BMI (N = 362,327) | | | | | | | | |
| IVW | 36 | -0.001 | 0.008 | 0.944 | 121.9 | 71.3 | 0.000 | - |
| MR-Egger | 36 | 0.001 | 0.039 | 0.977 | 121.9 | 72.1 | 0.000 | 0.965 |
| Weighted Median | 36 | -0.003 | 0.007 | 0.641 | - | - | - | - |
| JAE (N = 43,098, Ncase = 662, Ncontrol = 42,436) on Height (N = 364,629) | | | | | | | | |
| IVW | 8 | -0.008 | 0.004 | 0.055 | 5.7 | 0.0 | 0.581 | - |
| MR-Egger | 8 | 0.039 | 0.044 | 0.400 | 4.5 | 0.0 | 0.615 | 0.315 |
| Weighted Median | 8 | -0.007 | 0.006 | 0.221 | - | - | - | - |
| JAE (N = 43,098, Ncase = 662, Ncontrol = 42,436) on Weight (N = 370,087) | | | | | | | | |
| IVW | 8 | -0.015 | 0.011 | 0.167 | 28.2 | 75.1 | 0.000 | - |
| MR-Egger | 8 | -0.160 | 0.109 | 0.190 | 21.7 | 72.3 | 0.001 | 0.228 |
| Weighted Median | 8 | -0.019 | 0.009 | 0.030 | - | - | - | - |
| JAE (N = 43,098, Ncase = 662, Ncontrol = 42,436) on BMI (N = 362,327) | | | | | | | | |
| IVW | 8 | -0.013 | 0.012 | 0.295 | 29.8 | 76.5 | 0.000 | - |
| MR-Egger | 8 | -0.235 | 0.102 | 0.061 | 16.6 | 63.8 | 0.011 | 0.071 |
| Weighted Median | 8 | -0.012 | 0.008 | 0.140 | - | - | - | - |
| CAE (N = 43,485, Ncase = 1,049, Ncontrol = 42,436) on Height (N = 364,629) | | | | | | | | |
| IVW | 12 | 0.009 | 0.007 | 0.192 | 29.3 | 62.5 | 0.002 | - |
| MR-Egger | 12 | 0.008 | 0.032 | 0.812 | 28.5 | 64.9 | 0.001 | 0.604 |
| Weighted Median | 12 | 0.011 | 0.007 | 0.105 | - | - | - | - |
| CAE (N = 43,485, Ncase = 1,049, Ncontrol = 42,436) on Weight (N = 370,087) | | | | | | | | |
| IVW | 12 | 0.021 | 0.008 | 0.013 | 24.8 | 55.7 | 0.010 | - |
| MR-Egger | 12 | 0.072 | 0.036 | 0.072 | 20.4 | 51.1 | 0.025 | 0.173 |
| Weighted Median | 12 | 0.013 | 0.009 | 0.131 | - | - | - | - |
| CAE (N = 43,485, Ncase = 1,049, Ncontrol = 42,436) on BMI (N = 362,327) | | | | | | | | |
| IVW | 12 | 0.020 | 0.009 | 0.033 | 27.8 | 60.4 | 0.004 | - |
| MR-Egger | 12 | 0.082 | 0.040 | 0.070 | 22.3 | 55.1 | 0.014 | 0.147 |
| Weighted Median | 12 | 0.009 | 0.009 | 0.350 | - | - | - | - |
| GTCSA (N = 42,921, Ncase = 485, Ncontrol = 42,436) on Height (N = 364,629) | | | | | | | | |
| IVW | 4 | 0.003 | 0.006 | 0.628 | 3.4 | 11.6 | 0.335 | - |
| MR-Egger | 4 | -0.019 | 0.019 | 0.419 | 1.9 | 0.0 | 0.394 | 0.342 |
| Weighted Median | 4 | 0.002 | 0.007 | 0.774 | - | - | - | - |
| GTCSA (N = 42,921, Ncase = 485, Ncontrol = 42,436) on Weight (N = 370,087) | | | | | | | | |
| IVW | 4 | -0.005 | 0.008 | 0.563 | 2.4 | 0.0 | 0.500 | - |
| MR-Egger | 4 | -0.016 | 0.026 | 0.596 | 2.1 | 6.1 | 0.345 | 0.683 |
| Weighted Median | 4 | -0.002 | 0.009 | 0.843 | - | - | - | - |
| GTCSA (N = 42,921, Ncase = 485, Ncontrol = 42,436) on BMI (N = 362,327) | | | | | | | | |
| IVW | 4 | -0.006 | 0.008 | 0.457 | 1.7 | 0.0 | 0.639 | - |
| MR-Egger | 4 | -0.005 | 0.027 | 0.866 | 1.7 | 0.0 | 0.430 | 0.967 |
| Weighted Median | 4 | -0.003 | 0.010 | 0.732 | - | - | - | - |
| FE-HS (N = 43,696, Ncase = 1,260, Ncontrol = 42,436) on Height (N = 364,629) | | | | | | | | |
| IVW | 13 | -0.004 | 0.009 | 0.637 | 49.1 | 75.6 | 0.000 | - |
| MR-Egger | 13 | -0.013 | 0.018 | 0.505 | 47.8 | 77.0 | 0.000 | 0.603 |
| Weighted Median | 13 | -0.004 | 0.007 | 0.507 | - | - | - | - |
| FE-HS (N = 43,696, Ncase = 1,260, Ncontrol = 42,436) on Weight (N = 370,087) | | | | | | | | |
| IVW | 13 | 0.001 | 0.006 | 0.798 | 9.3 | 0.0 | 0.679 | - |
| MR-Egger | 13 | -0.013 | 0.012 | 0.276 | 7.2 | 0.0 | 0.786 | 0.174 |
| Weighted Median | 13 | 0.001 | 0.008 | 0.933 | - | - | - | - |
| FE-HS (N = 43,696, Ncase = 1,260, Ncontrol = 42,436) on BMI (N = 362,327) | | | | | | | | |
| IVW | 13 | 0.004 | 0.006 | 0.462 | 11.6 | 0.0 | 0.479 | - |
| MR-Egger | 13 | -0.008 | 0.012 | 0.552 | 10.4 | 0.0 | 0.498 | 0.291 |
| Weighted Median | 13 | 0.003 | 0.009 | 0.768 | - | - | - | - |
| FE-OL (N = 46,649, Ncase = 4,213, Ncontrol = 42,436) on Height (N = 364,629) | | | | | | | | |
| IVW | 4 | -0.019 | 0.027 | 0.476 | 10.0 | 70.1 | 0.018 | - |
| MR-Egger | 4 | -0.183 | 0.122 | 0.272 | 5.2 | 61.5 | 0.075 | 0.305 |
| Weighted Median | 4 | -0.008 | 0.02 | 0.704 | - | - | - | - |
| FE-OL (N = 46,649, Ncase = 4,213, Ncontrol = 42,436) on Weight (N = 370,087) | | | | | | | | |
| IVW | 4 | -0.033 | 0.025 | 0.191 | 4.9 | 39.3 | 0.176 | - |
| MR-Egger | 4 | -0.146 | 0.134 | 0.388 | 3.6 | 44.4 | 0.166 | 0.478 |
| Weighted Median | 4 | -0.016 | 0.025 | 0.529 | - | - | - | - |
| FE-OL (N = 46,649, Ncase = 4,213, Ncontrol = 42,436) on BMI (N = 362,327) | | | | | | | | |
| IVW | 4 | -0.029 | 0.021 | 0.171 | 2.2 | 0.0 | 0.539 | - |
| MR-Egger | 4 | -0.048 | 0.110 | 0.708 | 2.1 | 6.2 | 0.344 | 0.877 |
| Weighted Median | 4 | -0.025 | 0.026 | 0.325 | - | - | - | - |
| FE-NL (N = 48,214, Ncase = 5,778, Ncontrol = 42,436) on Height (N = 364,629) | | | | | | | | |
| IVW | 5 | 0.025 | 0.021 | 0.249 | 8.2 | 51.1 | 0.085 | - |
| MR-Egger | 5 | -0.073 | 0.128 | 0.611 | 6.8 | 56.0 | 0.078 | 0.497 |
| Weighted Median | 5 | 0.018 | 0.02 | 0.357 | - | - | - | - |
| FE-NL (N = 48,214, Ncase = 5,778, Ncontrol = 42,436) on Weight (N = 370,087) | | | | | | | | |
| IVW | 5 | 0.043 | 0.037 | 0.252 | 14.4 | 72.3 | 0.006 | - |
| MR-Egger | 5 | -0.175 | 0.210 | 0.466 | 10.5 | 71.5 | 0.015 | 0.369 |
| Weighted Median | 5 | 0.006 | 0.027 | 0.823 | - | - | - | - |
| FE-NL (N = 48,214, Ncase = 5,778, Ncontrol = 42,436) on BMI (N = 362,327) | | | | | | | | |
| IVW | 5 | 0.036 | 0.030 | 0.228 | 7.8 | 48.9 | 0.098 | - |
| MR-Egger | 5 | -0.119 | 0.172 | 0.539 | 6.1 | 51.0 | 0.106 | 0.429 |
| Weighted Median | 5 | -0.003 | 0.029 | 0.912 |  |  |  |  |

BMI: Body Mass Index; JME: juvenile myoclonic epilepsy; JAE: juvenile absence epilepsy; CAE: childhood absence epilepsy; GTCSA: generalized tonic-clonic seizures alone; FE-HS: Focal Epilepsy with hippocampal sclerosis; FE-OL: Focal Epilepsy with other lesions; FE-NL: Focal Epilepsy without lesions; IVW: Inverse-Variance Weighted; SNP: Single Nucleotide Polymorphism.

Table S4. Mendelian Randomization Estimates of the Causal Effect of 7 Epilepsy Subtypes on Stature and Obesity using FinnGen Data

| Method | NO. of SNPs | MR analysis | | | | Heterogeneity Test | | | MR-Egger intercept *P* |
| --- | --- | --- | --- | --- | --- | --- | --- | --- | --- |
|  |  | *OR* | *LCI* | *UCI* | *P* | Cochran’s *Q* | *I^2^* | *P* |  |
| JME (N = 44,168, Ncase = 1,732, Ncontrol = 42,436) on ISS (N = 362,599, Ncase = 611, Ncontrol = 361,988) | | | | | | | | | |
| IVW | 35 | 1.08 | 0.85 | 1.36 | 0.525 | 28.4 | 0.0 | 0.736 | - |
| MR-Egger | 35 | 0.70 | 0.23 | 2.12 | 0.530 | 27.8 | 0.0 | 0.722 | 0.437 |
| Weighted Median | 35 | 1.12 | 0.81 | 1.53 | 0.498 | - | - | - | - |
| JME (N = 44,168, Ncase = 1,732, Ncontrol = 42,436) on CTS (N = 362,116, Ncase = 128, Ncontrol = 361,988) | | | | | | | | | |
| IVW | 35 | 1.09 | 0.63 | 1.88 | 0.758 | 41.2 | 17.5 | 0.184 | - |
| MR-Egger | 35 | 2.85 | 0.21 | 39.36 | 0.440 | 40.5 | 18.6 | 0.172 | 0.468 |
| Weighted Median | 35 | 1.19 | 0.58 | 2.40 | 0.638 | - | - | - | - |
| JME (N = 44,168, Ncase = 1,732, Ncontrol = 42,436) on Obesity (N = 500,192, Ncase = 31,499, Ncontrol = 468,693) | | | | | | | | | |
| IVW | 36 | 0.99 | 0.96 | 1.03 | 0.729 | 37.4 | 6.5 | 0.359 | - |
| MR-Egger | 36 | 1.05 | 0.89 | 1.23 | 0.564 | 36.9 | 7.9 | 0.335 | 0.508 |
| Weighted Median | 36 | 0.98 | 0.93 | 1.03 | 0.402 | - | - | - | - |
| JAE (N = 43,098, Ncase = 662, Ncontrol = 42,436) on ISS (N = 362,599, Ncase = 611, Ncontrol = 361,988) | | | | | | | | | |
| IVW | 8 | 1.05 | 0.64 | 1.72 | 0.854 | 17.4 | 59.7 | 0.015 | - |
| MR-Egger | 8 | 0.06 | 0.00 | 10.97 | 0.336 | 14.6 | 58.9 | 0.024 | 0.327 |
| Weighted Median | 8 | 0.94 | 0.57 | 1.56 | 0.809 | - | - | - | - |
| JAE (N = 43,098, Ncase = 662, Ncontrol = 42,436) on CTS (N = 362,116, Ncase = 128, Ncontrol = 361,988) | | | | | | | | | |
| IVW | 8 | 0.79 | 0.32 | 1.95 | 0.615 | 12.6 | 44.5 | 0.082 | - |
| MR-Egger | 8 | 44.64 | 0.00 | 613131.30 | 0.464 | 11.3 | 46.9 | 0.079 | 0.437 |
| Weighted Median | 8 | 0.91 | 0.34 | 2.39 | 0.845 | - | - | - | - |
| JAE (N = 43,098, Ncase = 662, Ncontrol = 42,436) on Obesity (N = 500,192, Ncase = 31,499, Ncontrol = 468,693) | | | | | | | | | |
| IVW | 8 | 0.97 | 0.91 | 1.04 | 0.446 | 17.6 | 60.2 | 0.014 | - |
| MR-Egger | 8 | 0.59 | 0.30 | 1.16 | 0.178 | 13.0 | 53.9 | 0.043 | 0.196 |
| Weighted Median | 8 | 0.97 | 0.91 | 1.03 | 0.266 | - | - | - | - |
| CAE (N = 43,485, Ncase = 1,049, Ncontrol = 42,436) on ISS (N = 362,599, Ncase = 611, Ncontrol = 361,988) | | | | | | | | | |
| IVW | 12 | 0.78 | 0.57 | 1.06 | 0.116 | 9.9 | 0.0 | 0.544 | - |
| MR-Egger | 12 | 1.81 | 0.42 | 7.76 | 0.443 | 8.5 | 0.0 | 0.581 | 0.270 |
| Weighted Median | 12 | 0.77 | 0.49 | 1.20 | 0.252 | - | - | - | - |
| CAE (N = 43,485, Ncase = 1,049, Ncontrol = 42,436) on CTS (N = 362,116, Ncase = 128, Ncontrol = 361,988) | | | | | | | | | |
| IVW | 12 | 0.73 | 0.30 | 1.79 | 0.496 | 19.5 | 43.7 | 0.052 | - |
| MR-Egger | 12 | 0.03 | 0.00 | 1.46 | 0.108 | 15.4 | 35.1 | 0.118 | 0.132 |
| Weighted Median | 12 | 0.65 | 0.25 | 1.69 | 0.372 | - | - | - | - |
| CAE (N = 43,485, Ncase = 1,049, Ncontrol = 42,436) on Obesity (N = 500,192, Ncase = 31,499, Ncontrol = 468,693) | | | | | | | | | |
| IVW | 12 | 1.03 | 0.97 | 1.09 | 0.292 | 17.2 | 36.0 | 0.103 | - |
| MR-Egger | 12 | 1.35 | 1.10 | 1.65 | 0.015 | 9.9 | 0.0 | 0.450 | 0.022 |
| Weighted Median | 12 | 1.02 | 0.96 | 1.09 | 0.524 | - | - | - | - |
| GTCSA (N = 42,921, Ncase = 485, Ncontrol = 42,436) on ISS (N = 362,599, Ncase = 611, Ncontrol = 361,988) | | | | | | | | | |
| IVW | 4 | 1.26 | 0.80 | 1.97 | 0.314 | 2.1 | 0.0 | 0.555 | - |
| MR-Egger | 4 | 1.47 | 0.35 | 6.20 | 0.653 | 2.0 | 1.8 | 0.361 | 0.846 |
| Weighted Median | 4 | 1.20 | 0.69 | 2.10 | 0.495 | - | - | - | - |
| GTCSA (N = 42,921, Ncase = 485, Ncontrol = 42,436) on CTS (N = 362,116, Ncase = 128, Ncontrol = 361,988) | | | | | | | | | |
| IVW | 4 | 0.74 | 0.28 | 1.98 | 0.550 | 3.3 | 8.6 | 0.350 | - |
| MR-Egger | 4 | 1.16 | 0.03 | 50.12 | 0.947 | 3.2 | 37.3 | 0.203 | 0.830 |
| Weighted Median | 4 | 1.01 | 0.32 | 3.14 | 0.986 | - | - | - | - |
| GTCSA (N = 42,921, Ncase = 485, Ncontrol = 42,436) on Obesity (N = 500,192, Ncase = 31,499, Ncontrol = 468,693) | | | | | | | | | |
| IVW | 4 | 1.08 | 1.01 | 1.15 | 0.019 | 1.2 | 0.0 | 0.758 | - |
| MR-Egger | 4 | 1.05 | 0.86 | 1.28 | 0.689 | 1.1 | 0.0 | 0.580 | 0.794 |
| Weighted Median | 4 | 1.08 | 1.00 | 1.17 | 0.045 | - | - | - | - |
| FE-HS (N = 43,696, Ncase = 1,260, Ncontrol = 42,436) on ISS (N = 362,599, Ncase = 611, Ncontrol = 361,988) | | | | | | | | | |
| IVW | 13 | 1.10 | 0.80 | 1.53 | 0.548 | 6.2 | 0.0 | 0.905 | - |
| MR-Egger | 13 | 1.41 | 0.72 | 2.74 | 0.337 | 5.5 | 0.0 | 0.902 | 0.432 |
| Weighted Median | 13 | 1.26 | 0.81 | 1.98 | 0.315 | - | - | - | - |
| FE-HS (N = 43,696, Ncase = 1,260, Ncontrol = 42,436) on CTS (N = 362,116, Ncase = 128, Ncontrol = 361,988) | | | | | | | | | |
| IVW | 13 | 0.76 | 0.38 | 1.51 | 0.435 | 9.6 | 0.0 | 0.650 | - |
| MR-Egger | 13 | 0.77 | 0.19 | 3.15 | 0.718 | 9.6 | 0.0 | 0.566 | 0.993 |
| Weighted Median | 13 | 0.61 | 0.23 | 1.62 | 0.317 | - | - | - | - |
| FE-HS (N = 43,696, Ncase = 1,260, Ncontrol = 42,436) on Obesity (N = 500,192, Ncase = 31,499, Ncontrol = 468,693) | | | | | | | | | |
| IVW | 13 | 1.01 | 0.95 | 1.08 | 0.680 | 21.3 | 43.6 | 0.046 | - |
| MR-Egger | 13 | 0.96 | 0.85 | 1.09 | 0.538 | 19.7 | 44.0 | 0.050 | 0.360 |
| Weighted Median | 13 | 1.00 | 0.93 | 1.07 | 0.940 | - | - | - | - |
| FE-OL (N = 46,649, Ncase = 4,213, Ncontrol = 42,436) on ISS (N = 362,599, Ncase = 611, Ncontrol = 361,988) | | | | | | | | | |
| IVW | 4 | 1.43 | 0.42 | 4.91 | 0.565 | 3.7 | 19.2 | 0.294 | - |
| MR-Egger | 4 | 5.97 | 0.00 | 10531.70 | 0.685 | 3.5 | 42.2 | 0.177 | 0.740 |
| Weighted Median | 4 | 1.03 | 0.25 | 4.26 | 0.973 | - | - | - | - |
| FE-OL (N = 46,649, Ncase = 4,213, Ncontrol = 42,436) on CTS (N = 362,116, Ncase = 128, Ncontrol = 361,988) | | | | | | | | | |
| IVW | 4 | 0.05 | 0.00 | 0.55 | 0.014 | 1.1 | 0.0 | 0.766 | - |
| MR-Egger | 4 | 1.72 | 0.00 | 303340.46 | 0.938 | 0.8 | 0.0 | 0.667 | 0.620 |
| Weighted Median | 4 | 0.03 | 0.00 | 0.53 | 0.017 | - | - | - | - |
| FE-OL (N = 46,649, Ncase = 4,213, Ncontrol = 42,436) on Obesity (N = 500,192, Ncase = 31,499, Ncontrol = 468,693) | | | | | | | | | |
| IVW | 4 | 1.07 | 0.91 | 1.25 | 0.414 | 1.6 | 0.0 | 0.671 | - |
| MR-Egger | 4 | 0.67 | 0.30 | 1.50 | 0.435 | 0.2 | 0.0 | 0.894 | 0.369 |
| Weighted Median | 4 | 1.05 | 0.86 | 1.27 | 0.642 | - | - | - | - |
| FE-NL (N = 48,214, Ncase = 5,778, Ncontrol = 42,436) on ISS (N = 362,599, Ncase = 611, Ncontrol = 361,988) | | | | | | | | | |
| IVW | 5 | 0.63 | 0.21 | 1.96 | 0.431 | 1.9 | 0.0 | 0.762 | - |
| MR-Egger | 5 | 0.49 | 0.00 | 302.06 | 0.843 | 1.9 | 0.0 | 0.604 | 0.943 |
| Weighted Median | 5 | 0.53 | 0.14 | 2.03 | 0.364 | - | - | - | - |
| FE-NL (N = 48,214, Ncase = 5,778, Ncontrol = 42,436) on CTS (N = 362,116, Ncase = 128, Ncontrol = 361,988) | | | | | | | | | |
| IVW | 5 | 4.64 | 0.42 | 51.14 | 0.210 | 3.7 | 0.0 | 0.454 | - |
| MR-Egger | 5 | 0.54 | 0.00 | 1506491.03 | 0.940 | 3.6 | 15.7 | 0.313 | 0.792 |
| Weighted Median | 5 | 3.54 | 0.12 | 101.79 | 0.460 | - | - | - | - |
| FE-NL (N = 48,214, Ncase = 5,778, Ncontrol = 42,436) on Obesity (N = 500,192, Ncase = 31,499, Ncontrol = 468,693) | | | | | | | | | |
| IVW | 5 | 1.04 | 0.81 | 1.35 | 0.745 | 10.8 | 62.9 | 0.029 | - |
| MR-Egger | 5 | 0.83 | 0.15 | 4.43 | 0.840 | 10.5 | 71.5 | 0.015 | 0.801 |
| Weighted Median | 5 | 0.95 | 0.76 | 1.18 | 0.625 | - | - | - | - |

ISS: Idiopathic Short Stature; CTS: Constitutional Tall Stature; JME: juvenile myoclonic epilepsy; JAE: juvenile absence epilepsy; CAE: childhood absence epilepsy; GTCSA: generalized tonic-clonic seizures alone; FE-HS: Focal Epilepsy with hippocampal sclerosis; FE-OL: Focal Epilepsy with other lesions; FE-NL: Focal Epilepsy without lesions; IVW: Inverse-Variance Weighted; SNP: Single Nucleotide Polymorphism.

Table S5. Mendelian Randomization Estimates of the Causal Effect of 7 Epilepsy Subtypes on Anthropometric Traits Using pan-UKB Data

| Method | NO. of SNPs | MR a-lysis | | | Heterogeneity Test | | | *p* of MR-Egger intercept |
| --- | --- | --- | --- | --- | --- | --- | --- | --- |
|  |  | *β* | *se* | *p* | Cochran’s *Q* | *I^2^* | *p* |  |
| JME (N = 44,168, Ncase = 1,732, Ncontrol = 42,436) on Height (N = 419,596) | | | | | | | | |
| IVW | 29 | 0.002 | 0.006 | 0.780 | 56.3 | 52.0 | 0.001 | - |
| MR-Egger | 29 | -0.008 | 0.028 | 0.786 | 56.5 | 50.5 | 0.001 | 0.733 |
| Weighted Median | 29 | 0.010 | 0.007 | 0.129 | - | - | - | - |
| JME (N = 44,168, Ncase = 1,732, Ncontrol = 42,436) on Weight (N = 419,316) | | | | | | | | |
| IVW | 29 | -0.001 | 0.007 | 0.921 | 46.4 | 41.8 | 0.012 | - |
| MR-Egger | 29 | 0.052 | 0.026 | 0.061 | 53.6 | 47.7 | 0.003 | 0.051 |
| Weighted Median | 29 | -0.005 | 0.008 | 0.549 | - | - | - | - |
| JME (N = 44,168, Ncase = 1,732, Ncontrol = 42,436) on BMI (N = 419,163) | | | | | | | | |
| IVW | 31 | -0.005 | 0.008 | 0.557 | 64.4 | 54.9 | 0.000 | - |
| MR-Egger | 31 | 0.032 | 0.033 | 0.340 | 67.3 | 55.4 | 0.000 | 0.261 |
| Weighted Median | 31 | -0.006 | 0.008 | 0.476 | - | - | - | - |
| JAE (N = 43,098, Ncase = 662, Ncontrol = 42,436) on Height (N = 419,596) | | | | | | | | |
| IVW | 9 | 0.001 | 0.006 | 0.925 | 11.7 | 40.1 | 0.111 | - |
| MR-Egger | 9 | -0.006 | 0.044 | 0.895 | 11.7 | 31.8 | 0.164 | 0.884 |
| Weighted Median | 9 | 0.002 | 0.007 | 0.821 | - | - | - | - |
| JAE (N = 43,098, Ncase = 662, Ncontrol = 42,436) on Weight (N = 419,316) | | | | | | | | |
| IVW | 9 | -0.007 | 0.008 | 0.403 | 15.6 | 55.1 | 0.029 | - |
| MR-Egger | 9 | -0.015 | 0.059 | 0.813 | 15.6 | 48.8 | 0.048 | 0.898 |
| Weighted Median | 9 | -0.010 | 0.009 | 0.240 | - | - | - | - |
| JAE (N = 43,098, Ncase = 662, Ncontrol = 42,436) on BMI (N = 419,163) | | | | | | | | |
| IVW | 9 | -0.009 | 0.007 | 0.176 | 8.5 | 18.0 | 0.287 | - |
| MR-Egger | 9 | -0.012 | 0.049 | 0.818 | 8.5 | 6.4 | 0.382 | 0.957 |
| Weighted Median | 9 | -0.012 | 0.009 | 0.211 | - | - | - | - |
| CAE (N = 43,485, Ncase = 1,049, Ncontrol = 42,436) on Height (N = 419,596) | | | | | | | | |
| IVW | 8 | -0.002 | 0.010 | 0.861 | 17.6 | 65.9 | 0.007 | - |
| MR-Egger | 8 | 0.014 | 0.040 | 0.734 | 18.1 | 61.3 | 0.012 | 0.690 |
| Weighted Median | 8 | -0.002 | 0.010 | 0.808 | - | - | - | - |
| CAE (N = 43,485, Ncase = 1,049, Ncontrol = 42,436) on Weight (N = 419,316) | | | | | | | | |
| IVW | 11 | -0.007 | 0.008 | 0.384 | 14.6 | 38.5 | 0.101 | - |
| MR-Egger | 11 | 0.019 | 0.026 | 0.488 | 16.4 | 38.9 | 0.090 | 0.329 |
| Weighted Median | 11 | -0.010 | 0.009 | 0.283 | - | - | - | - |
| CAE (N = 43,485, Ncase = 1,049, Ncontrol = 42,436) on BMI (N = 419,163) | | | | | | | | |
| IVW | 12 | 0.000 | 0.010 | 0.986 | 25.6 | 61.0 | 0.004 | - |
| MR-Egger | 12 | 0.000 | 0.036 | 0.998 | 25.6 | 57.1 | 0.007 | 0.998 |
| Weighted Median | 12 | -0.004 | 0.010 | 0.675 | - | - | - | - |
| GTCSA (N = 42,921, Ncase = 485, Ncontrol = 42,436) on Height (N = 419,596) | | | | | | | | |
| IVW | 4 | -0.002 | 0.006 | 0.789 | 2.8 | 28.9 | 0.245 | - |
| MR-Egger | 4 | -0.007 | 0.024 | 0.813 | 2.9 | 0.0 | 0.411 | 0.853 |
| Weighted Median | 4 | 0.002 | 0.007 | 0.788 | - | - | - | - |
| GTCSA (N = 42,921, Ncase = 485, Ncontrol = 42,436) on Weight (N = 419,316) | | | | | | | | |
| IVW | 4 | 0.004 | 0.014 | 0.756 | 8.6 | 76.9 | 0.013 | - |
| MR-Egger | 4 | -0.036 | 0.049 | 0.543 | 11.8 | 74.7 | 0.008 | 0.481 |
| Weighted Median | 4 | -0.002 | 0.010 | 0.850 | - | - | - | - |
| GTCSA (N = 42,921, Ncase = 485, Ncontrol = 42,436) on BMI (N = 419,163) | | | | | | | | |
| IVW | 4 | 0.004 | 0.015 | 0.783 | 7.5 | 73.5 | 0.023 | - |
| MR-Egger | 4 | -0.040 | 0.051 | 0.510 | 10.8 | 72.1 | 0.013 | 0.454 |
| Weighted Median | 4 | 0.003 | 0.011 | 0.774 | - | - | - | - |
| FE-HS (N = 43,696, Ncase = 1,260, Ncontrol = 42,436) on Height (N = 419,596) | | | | | | | | |
| IVW | 12 | 0.015 | 0.010 | 0.133 | 24.8 | 59.7 | 0.006 | - |
| MR-Egger | 12 | 0.030 | 0.027 | 0.296 | 25.7 | 57.3 | 0.007 | 0.552 |
| Weighted Median | 12 | 0.013 | 0.010 | 0.185 | - | - | - | - |
| FE-HS (N = 43,696, Ncase = 1,260, Ncontrol = 42,436) on Weight (N = 419,316) | | | | | | | | |
| IVW | 13 | 0.005 | 0.007 | 0.464 | 17.6 | 37.5 | 0.091 | - |
| MR-Egger | 13 | 0.000 | 0.014 | 0.982 | 17.9 | 33.0 | 0.118 | 0.670 |
| Weighted Median | 13 | 0.003 | 0.008 | 0.686 | - | - | - | - |
| FE-HS (N = 43,696, Ncase = 1,260, Ncontrol = 42,436) on BMI (N = 419,163) | | | | | | | | |
| IVW | 12 | -0.001 | 0.007 | 0.932 | 14.0 | 28.3 | 0.175 | - |
| MR-Egger | 12 | 0.000 | 0.014 | 0.982 | 14.0 | 21.2 | 0.235 | 0.941 |
| Weighted Median | 12 | 0.005 | 0.008 | 0.549 | - | - | - | - |
| FE-OL (N = 46,649, Ncase = 4,213, Ncontrol = 42,436) on Height (N = 419,596) | | | | | | | | |
| IVW | 4 | 0.009 | 0.017 | 0.594 | 1.0 | 0.0 | 0.595 | - |
| MR-Egger | 4 | 0.054 | 0.095 | 0.627 | 1.3 | 0.0 | 0.737 | 0.680 |
| Weighted Median | 4 | -0.002 | 0.020 | 0.906 | - | - | - | - |
| FE-OL (N = 46,649, Ncase = 4,213, Ncontrol = 42,436) on Weight (N = 419,316) | | | | | | | | |
| IVW | 4 | -0.046 | 0.029 | 0.111 | 6.0 | 66.4 | 0.051 | - |
| MR-Egger | 4 | -0.007 | 0.189 | 0.974 | 6.1 | 50.7 | 0.107 | 0.855 |
| Weighted Median | 4 | -0.044 | 0.029 | 0.135 | - | - | - | - |
| FE-OL (N = 46,649, Ncase = 4,213, Ncontrol = 42,436) on BMI (N = 419,163) | | | | | | | | |
| IVW | 4 | -0.060 | 0.034 | 0.075 | 6.7 | 70.4 | 0.034 | - |
| MR-Egger | 4 | -0.009 | 0.223 | 0.970 | 6.9 | 56.7 | 0.074 | 0.838 |
| Weighted Median | 4 | -0.063 | 0.031 | 0.041 | - | - | - | - |
| FE-NL (N = 48,214, Ncase = 5,778, Ncontrol = 42,436) on Height (N = 419,596) | | | | | | | | |
| IVW | 5 | -0.018 | 0.019 | 0.350 | 3.8 | 20.4 | 0.288 | - |
| MR-Egger | 5 | 0.050 | 0.107 | 0.669 | 4.3 | 6.9 | 0.367 | 0.563 |
| Weighted Median | 5 | -0.008 | 0.023 | 0.748 | - | - | - | - |
| FE-NL (N = 48,214, Ncase = 5,778, Ncontrol = 42,436) on Weight (N = 419,316) | | | | | | | | |
| IVW | 5 | 0.056 | 0.028 | 0.048 | 7.2 | 58.1 | 0.067 | - |
| MR-Egger | 5 | 0.034 | 0.170 | 0.853 | 7.2 | 44.4 | 0.126 | 0.906 |
| Weighted Median | 5 | 0.018 | 0.030 | 0.560 | - | - | - | - |
| FE-NL (N = 48,214, Ncase = 5,778, Ncontrol = 42,436) on BMI (N = 419,163) | | | | | | | | |
| IVW | 5 | 0.071 | 0.028 | 0.011 | 5.3 | 43.8 | 0.149 | - |
| MR-Egger | 5 | 0.003 | 0.163 | 0.985 | 5.7 | 29.3 | 0.226 | 0.702 |
| Weighted Median | 5 | 0.062 | 0.034 | 0.069 | - | - | - | - |

BMI: Body Mass Index; JME: juvenile myoclonic epilepsy; JAE: juvenile absence epilepsy; CAE: childhood absence epilepsy; GTCSA: generalized tonic-clonic seizures alone; FE-HS: Focal Epilepsy with hippocampal sclerosis; FE-OL: Focal Epilepsy with other lesions; FE-NL: Focal Epilepsy without lesions; IVW: Inverse-Variance Weighted; SNP: Single Nucleotide Polymorphism.

Table S6. Mendelian Randomization Estimates of the Causal Effect of 7 Epilepsy Subtypes on Obesity using pan-UKB Data

| Method | NO. of SNPs | MR analysis | | | | Heterogeneity Test | | | MR-Egger intercept *P* |
| --- | --- | --- | --- | --- | --- | --- | --- | --- | --- |
|  |  | *OR* | *LCI* | *UCI* | *P* | Cochran’s *Q* | *I^2^* | *P* |  |
| JME (N = 44,168, Ncase = 1,732, Ncontrol = 42,436) on Obesity (N = 420,531, Ncase = 15,917, Ncontrol = 404,614) | | | | | | | | | |
| IVW | 35 | 1.01 | 0.95 | 1.07 | 0.753 | 48.9 | 32.6 | 0.037 | - |
| MR-Egger | 35 | 0.96 | 0.76 | 1.21 | 0.726 | 49.2 | 30.9 | 0.044 | 0.663 |
| Weighted Median | 35 | 1.02 | 0.96 | 1.09 | 0.521 | - | - | - | - |
| JAE (N = 43,098, Ncase = 662, Ncontrol = 42,436) on Obesity (N = 420,531, Ncase = 15,917, Ncontrol = 404,614) | | | | | | | | | |
| IVW | 9 | 1.01 | 0.95 | 1.07 | 0.855 | 5.8 | 0.0 | 0.565 | - |
| MR-Egger | 9 | 1.00 | 0.67 | 1.50 | 0.999 | 5.8 | 0.0 | 0.672 | 0.980 |
| Weighted Median | 9 | 0.99 | 0.91 | 1.07 | 0.787 | - | - | - | - |
| CAE (N = 43,485, Ncase = 1,049, Ncontrol = 42,436) on Obesity (N = 420,531, Ncase = 15,917, Ncontrol = 404,614) | | | | | | | | | |
| IVW | 13 | 1.06 | 0.98 | 1.13 | 0.126 | 16.8 | 34.6 | 0.113 | - |
| MR-Egger | 13 | 1.11 | 0.87 | 1.42 | 0.426 | 17.1 | 29.8 | 0.146 | 0.686 |
| Weighted Median | 13 | 1.03 | 0.95 | 1.12 | 0.455 | - | - | - | - |
| GTCSA (N = 42,921, Ncase = 485, Ncontrol = 42,436) on Obesity (N = 420,531, Ncase = 15,917, Ncontrol = 404,614) | | | | | | | | | |
| IVW | 4 | 1.00 | 0.92 | 1.09 | 0.986 | 1.5 | 0.0 | 0.466 | - |
| MR-Egger | 4 | 0.84 | 0.67 | 1.07 | 0.301 | 3.7 | 18.1 | 0.300 | 0.281 |
| Weighted Median | 4 | 1.03 | 0.94 | 1.12 | 0.569 | - | - | - | - |
| FE-HS (N = 43,696, Ncase = 1,260, Ncontrol = 42,436) on Obesity (N = 420,531, Ncase = 15,917, Ncontrol = 404,614) | | | | | | | | | |
| IVW | 13 | 1.00 | 0.93 | 1.07 | 0.921 | 17.6 | 37.6 | 0.091 | - |
| MR-Egger | 13 | 1.03 | 0.90 | 1.19 | 0.636 | 18.2 | 34.2 | 0.109 | 0.548 |
| Weighted Median | 13 | 0.99 | 0.92 | 1.08 | 0.878 | - | - | - | - |
| FE-OL (N = 46,649, Ncase = 4,213, Ncontrol = 42,436) on Obesity (N = 420,531, Ncase = 15,917, Ncontrol = 404,614) | | | | | | | | | |
| IVW | 4 | 0.98 | 0.80 | 1.20 | 0.856 | 2.2 | 7.3 | 0.340 | - |
| MR-Egger | 4 | 0.94 | 0.30 | 2.96 | 0.922 | 2.2 | 0.0 | 0.539 | 0.943 |
| Weighted Median | 4 | 1.00 | 0.78 | 1.29 | 0.992 | - | - | - | - |
| FE-NL (N = 48,214, Ncase = 5,778, Ncontrol = 42,436) on Obesity (N = 420,531, Ncase = 15,917, Ncontrol = 404,614) | | | | | | | | | |
| IVW | 5 | 1.07 | 0.86 | 1.33 | 0.537 | 3.7 | 19.7 | 0.292 | - |
| MR-Egger | 5 | 0.78 | 0.23 | 2.71 | 0.725 | 4.0 | 1.2 | 0.400 | 0.650 |
| Weighted Median | 5 | 1.05 | 0.79 | 1.39 | 0.740 | - | - | - | - |

JME: juvenile myoclonic epilepsy; JAE: juvenile absence epilepsy; CAE: childhood absence epilepsy; GTCSA: generalized tonic-clonic seizures alone; FE-HS: Focal Epilepsy with hippocampal sclerosis; FE-OL: Focal Epilepsy with other lesions; FE-NL: Focal Epilepsy without lesions; IVW: Inverse-Variance Weighted; SNP: Single Nucleotide Polymorphism.
