# Supplementary material for: Exploring the causal effects of epilepsy and its subtypes on anthropometric traits: A 2-sample Mendelian randomization study
Source: Medicine (Baltimore). 2025 Sep 19;104(38):e44619. doi: 10.1097/MD.0000000000044619 (PMC12459504; doi:10.1097/MD.0000000000044619)
Supplement: Supplementary file 3 [file medi-104-e44619-s003.pdf]

Leave−One−Out Forest Plot for Epilepsy Effect on Height

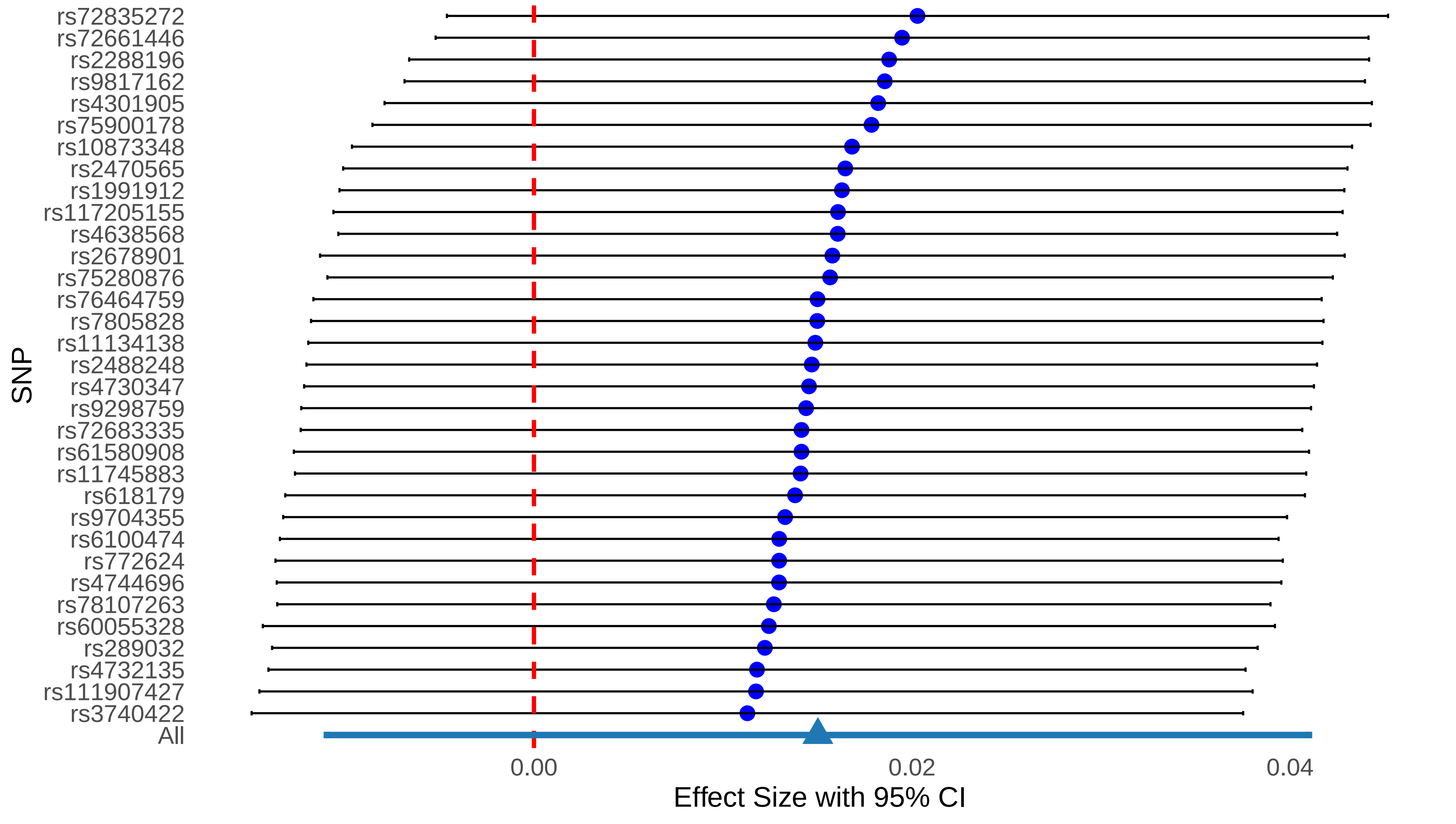

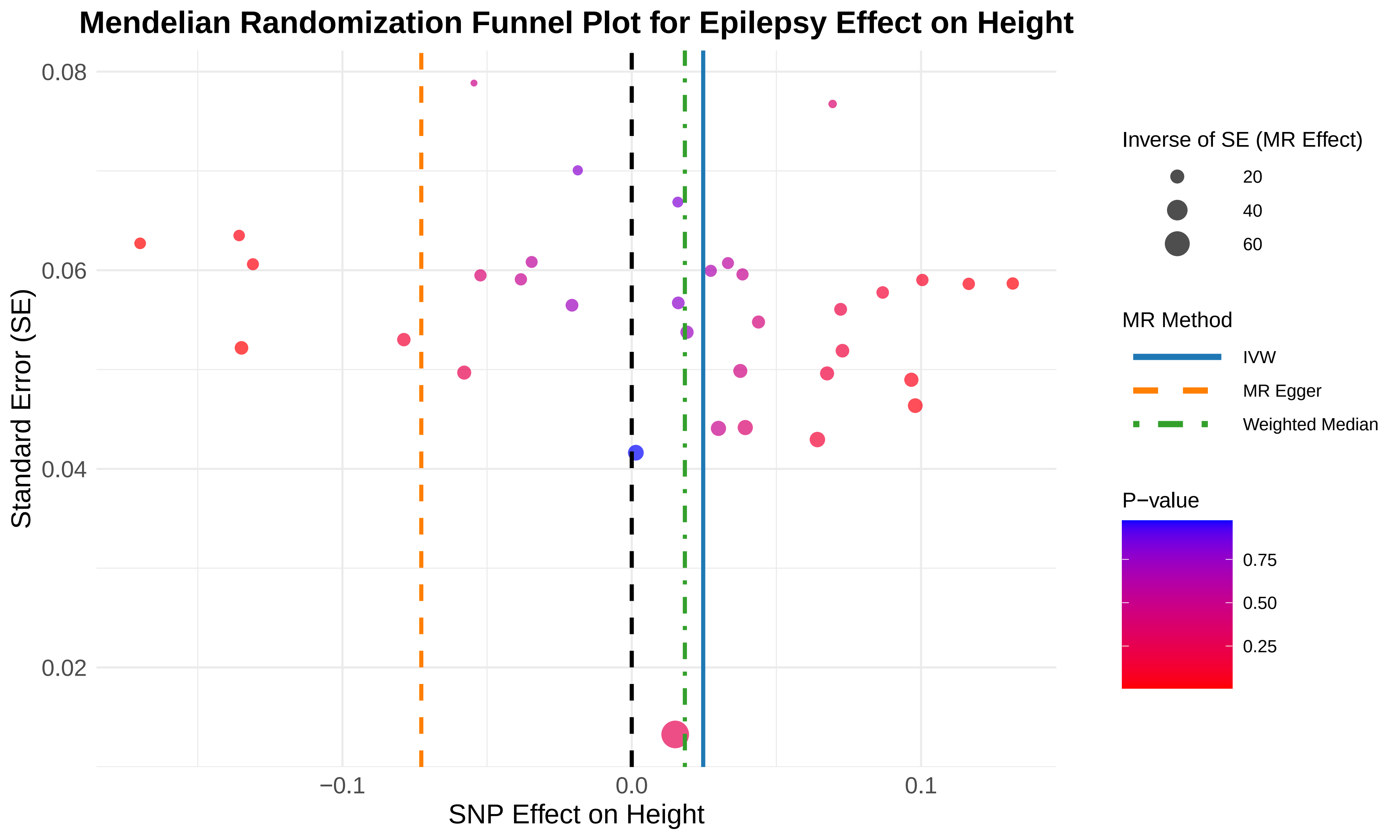
Mendelian Randomization Funnel Plot for Epilepsy Effect on Height
0.08
0.06
0.04
0.02
Standard Error (SE)
−0.1
0.0
0.1
SNP Effect on Height
Inverse of SE (MR Effect)
20
40
60
MR Method
IVW
MR Egger
Weighted Median
P−value
0.75
0.50
0.25

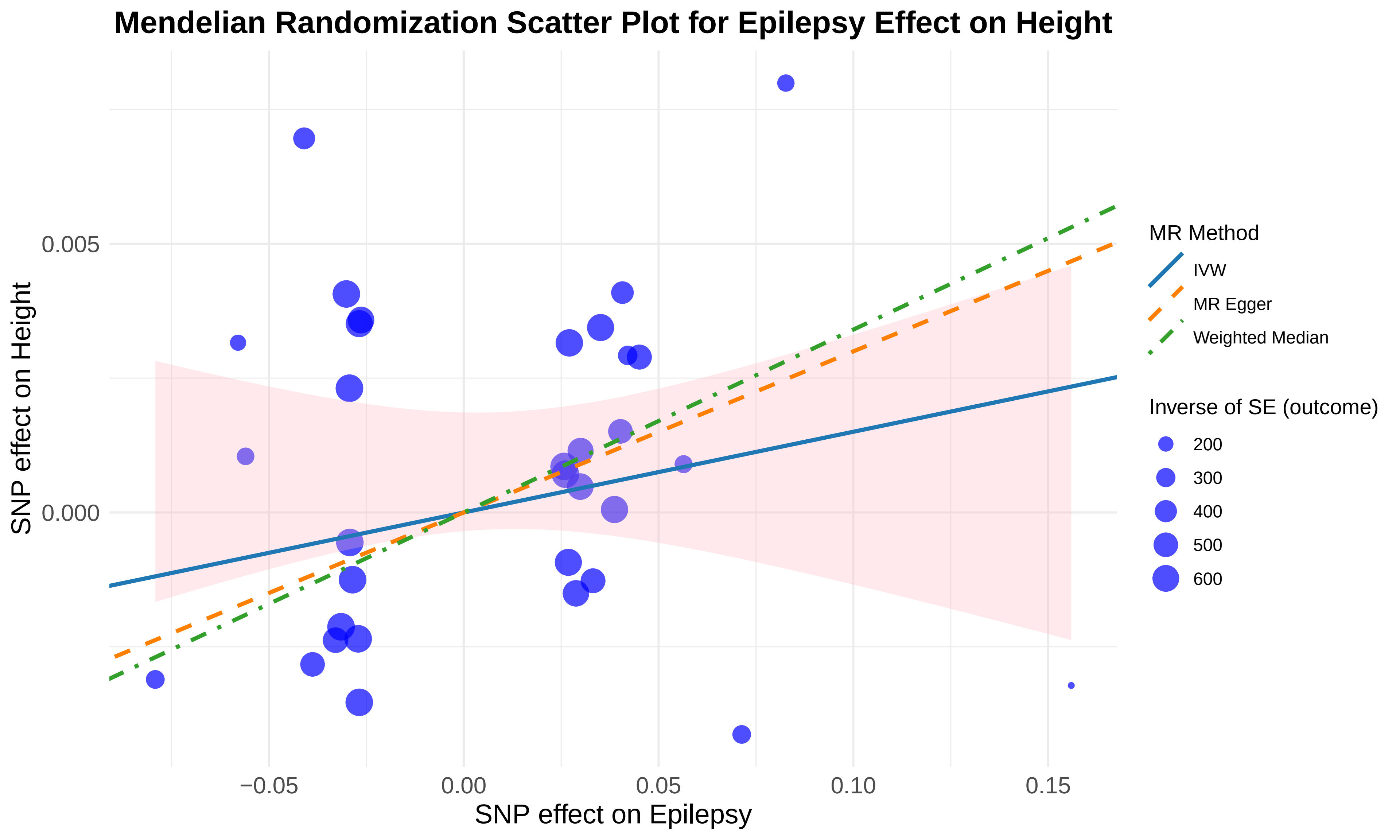

Mendelian Randomization Scatter Plot for Epilepsy Effect on Height
SNP effect on Height
0.005
0.000
−0.05
0.00
0.05
0.10
0.15
SNP effect on Epilepsy
MR Method
IVW
MR Egger
Weighted Median
Inverse of SE (outcome)
200
300
400
500
600

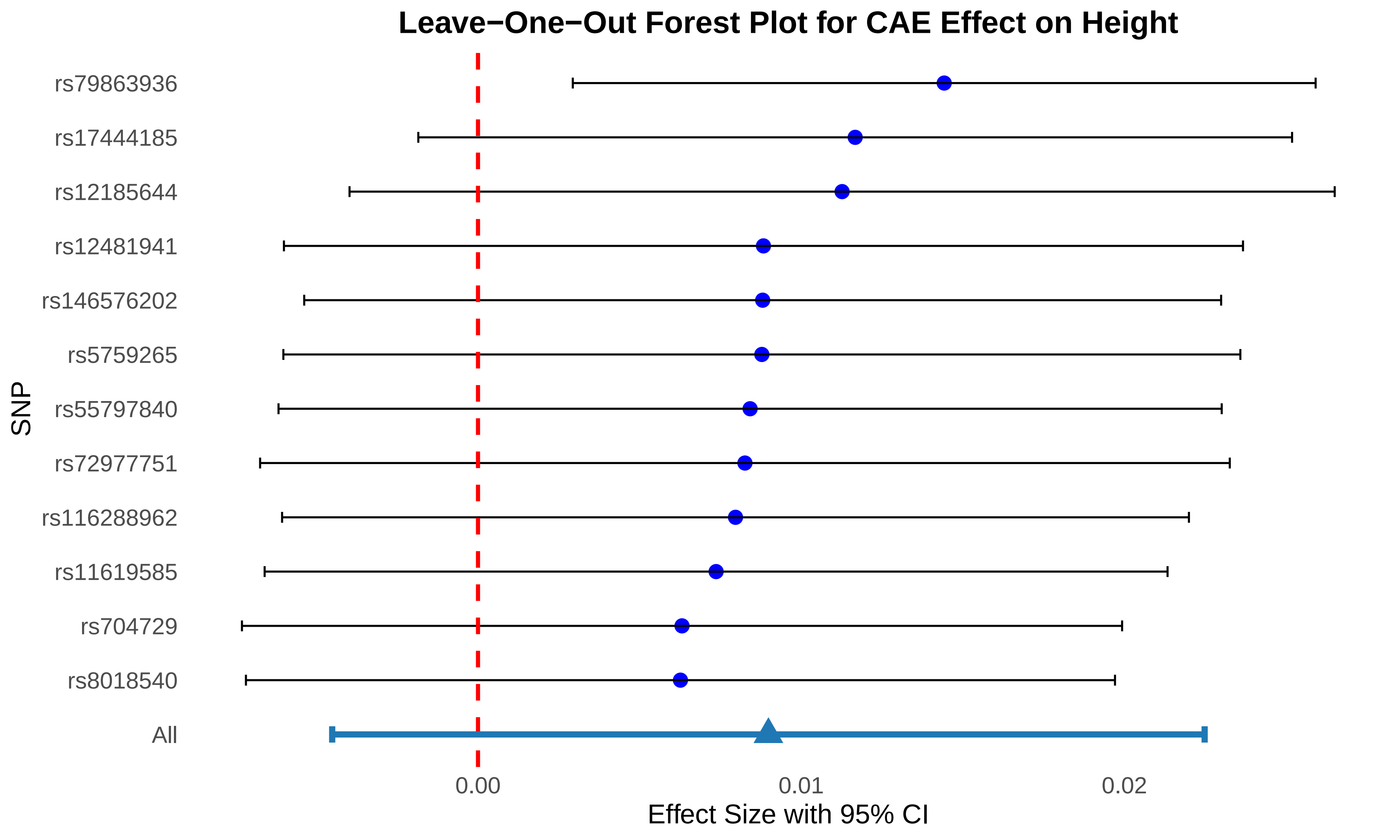

Leave−One−Out Forest Plot for CAE Effect on Height
rs79863936
rs17444185
rs12185644
rs12481941
rs146576202
rs5759265
rs55797840
rs72977751
rs116288962
rs11619585
rs704729
rs8018540
All
SNP
0.00
0.01
0.02
Effect Size with 95% CI

Mendelian Randomization Funnel Plot for CAE Effect on Height

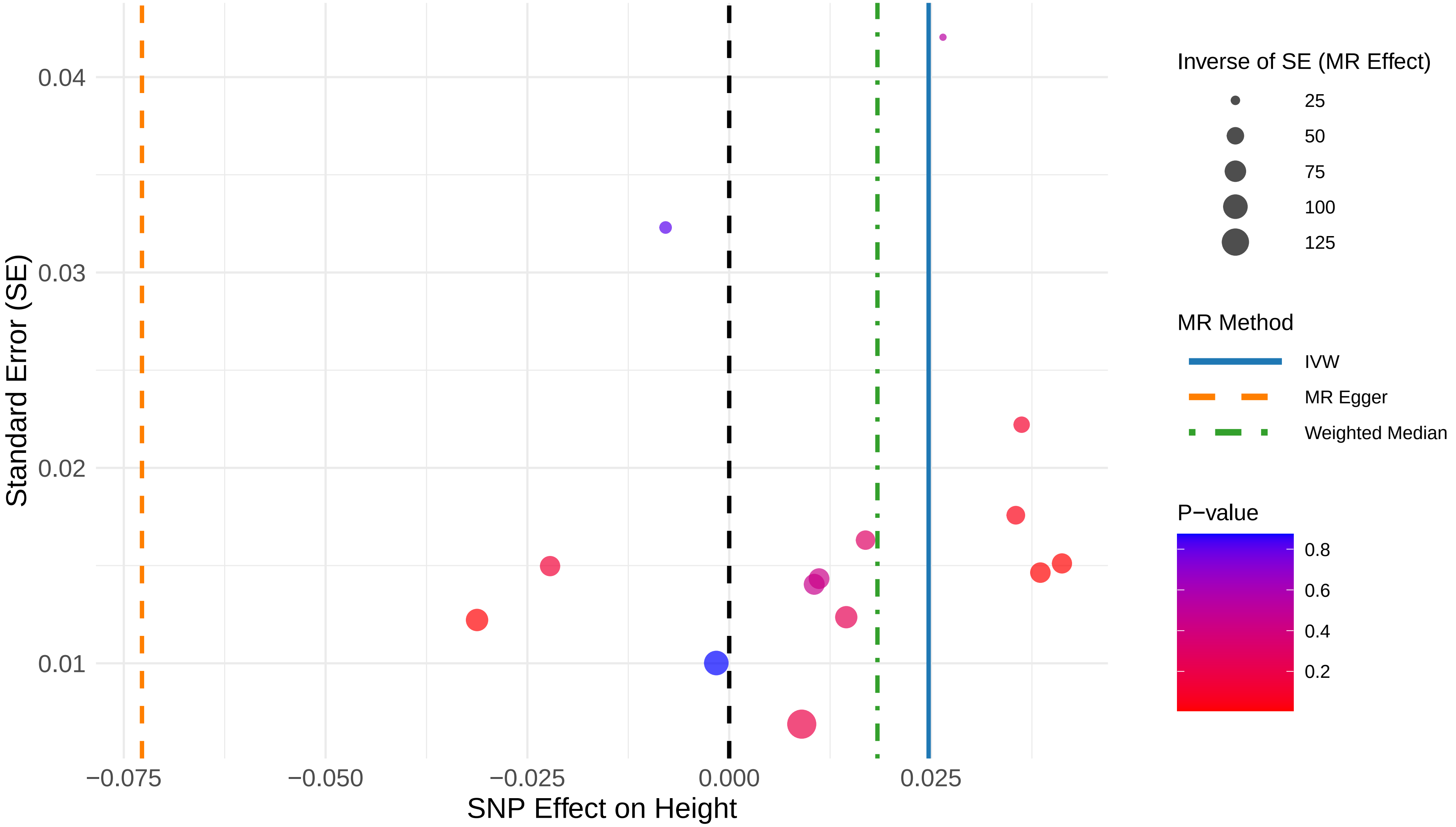

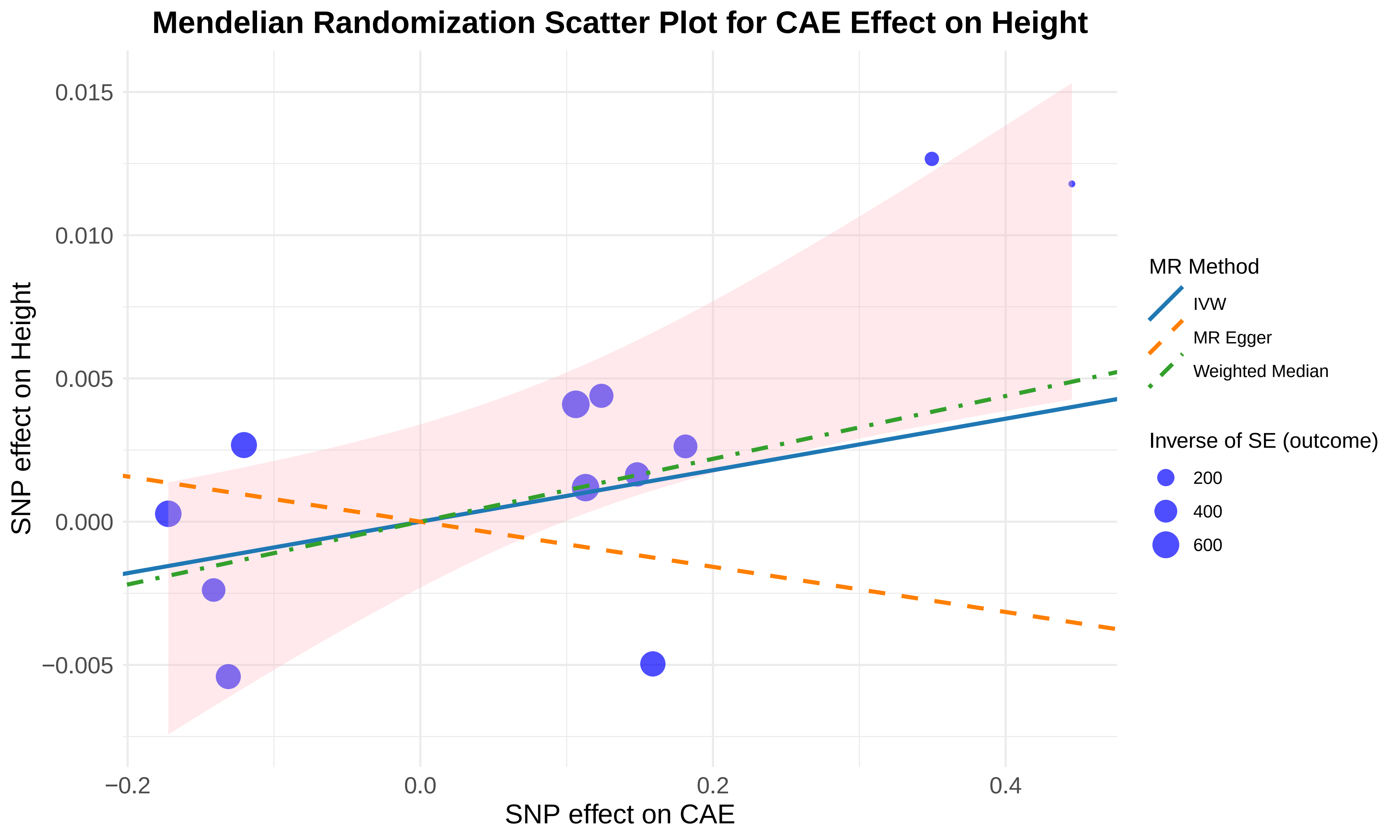
Mendelian Randomization Scatter Plot for CAE Effect on Height
SNP effect on Height
0.015
0.010
0.005
0.000
−0.005
−0.2
0.0
0.2
0.4
SNP effect on CAE
MR Method
IVW
MR Egger
Weighted Median
Inverse of SE (outcome)
200
400
600

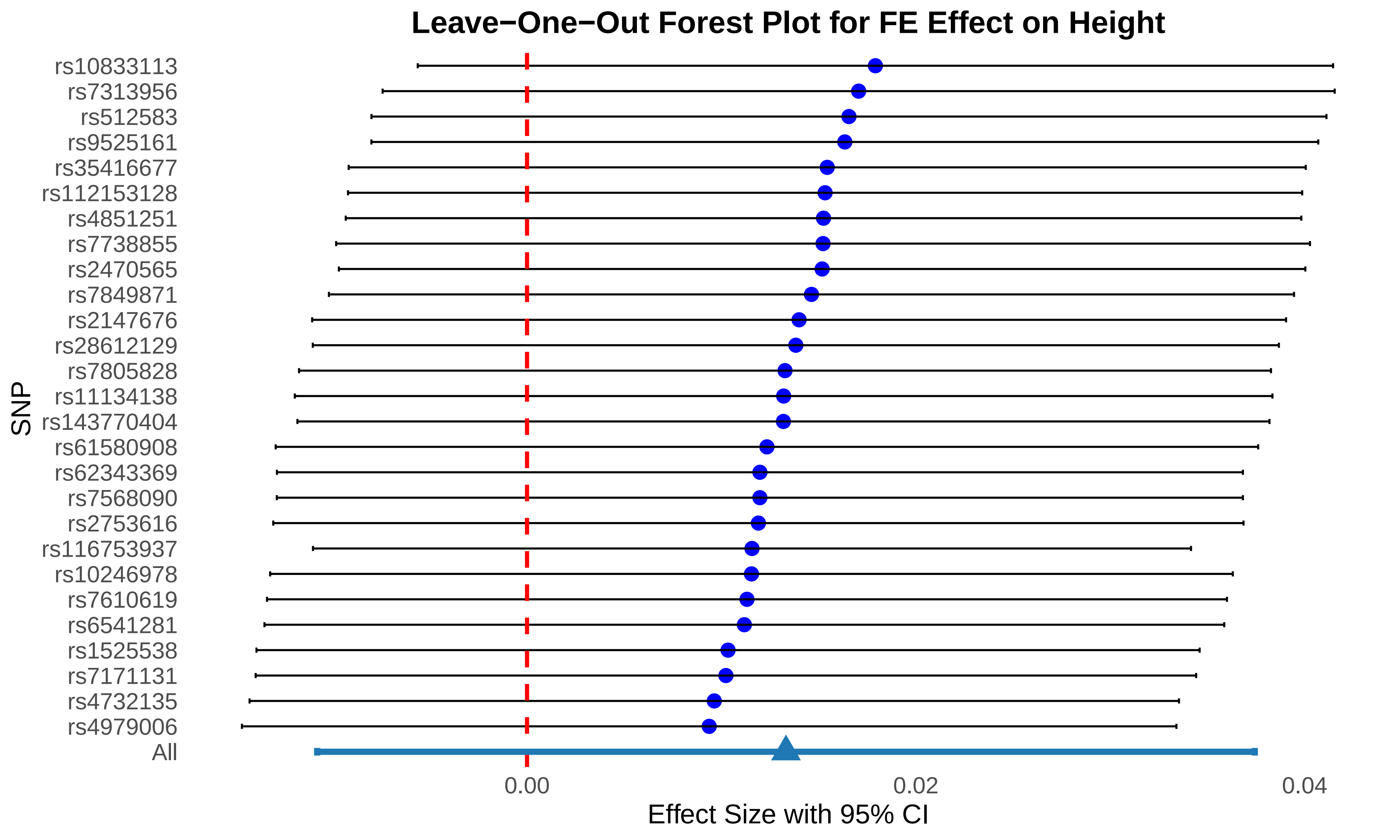
Leave−One−Out Forest Plot for FE Effect on Height
rs10833113
rs7313956
rs512583
rs9525161
rs35416677
rs112153128
rs4851251
rs7738855
rs2470565
rs7849871
rs2147676
rs28612129
rs7805828
rs11134138
rs143770404
rs61580908
rs62343369
rs7568090
rs2753616
rs116753937
rs10246978
rs7610619
rs6541281
rs1525538
rs7171131
rs4732135
rs4979006
All
SNP
0.00
0.02
0.04
Effect Size with 95% CI

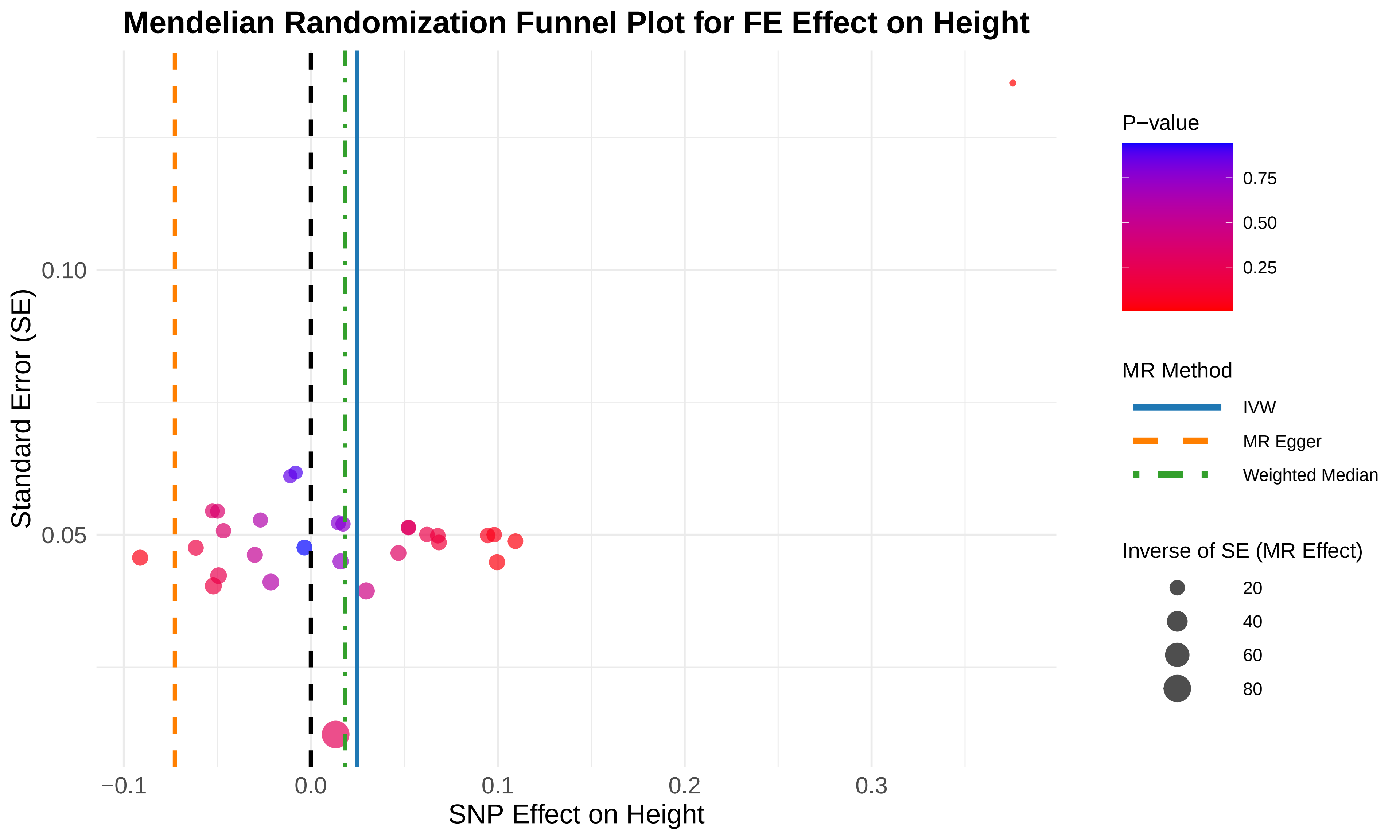

Mendelian Randomization Funnel Plot for FE Effect on Height
Standard Error (SE)
0.10
0.05
−0.1
0.0
0.1
0.2
0.3
SNP Effect on Height
P−value
0.75
0.50
0.25
MR Method
IVW
MR Egger
Weighted Median
Inverse of SE (MR Effect)
20
40
60
80

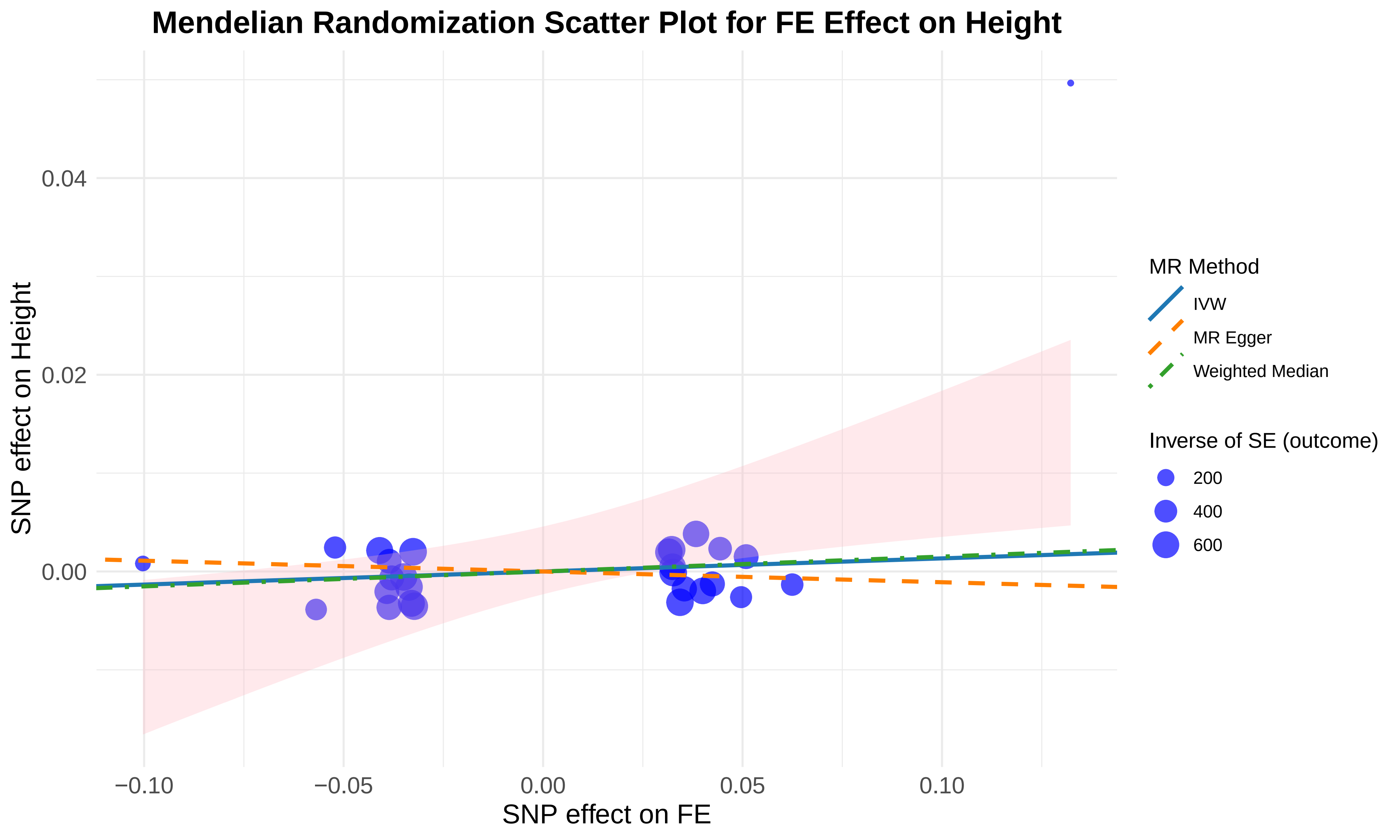
Mendelian Randomization Scatter Plot for FE Effect on Height
SNP effect on Height
SNP effect on FE
0.04
0.02
0.00
−0.10
−0.05
0.00
0.05
0.10
MR Method
IVW
MR Egger
Weighted Median
Inverse of SE (outcome)
200
400
600

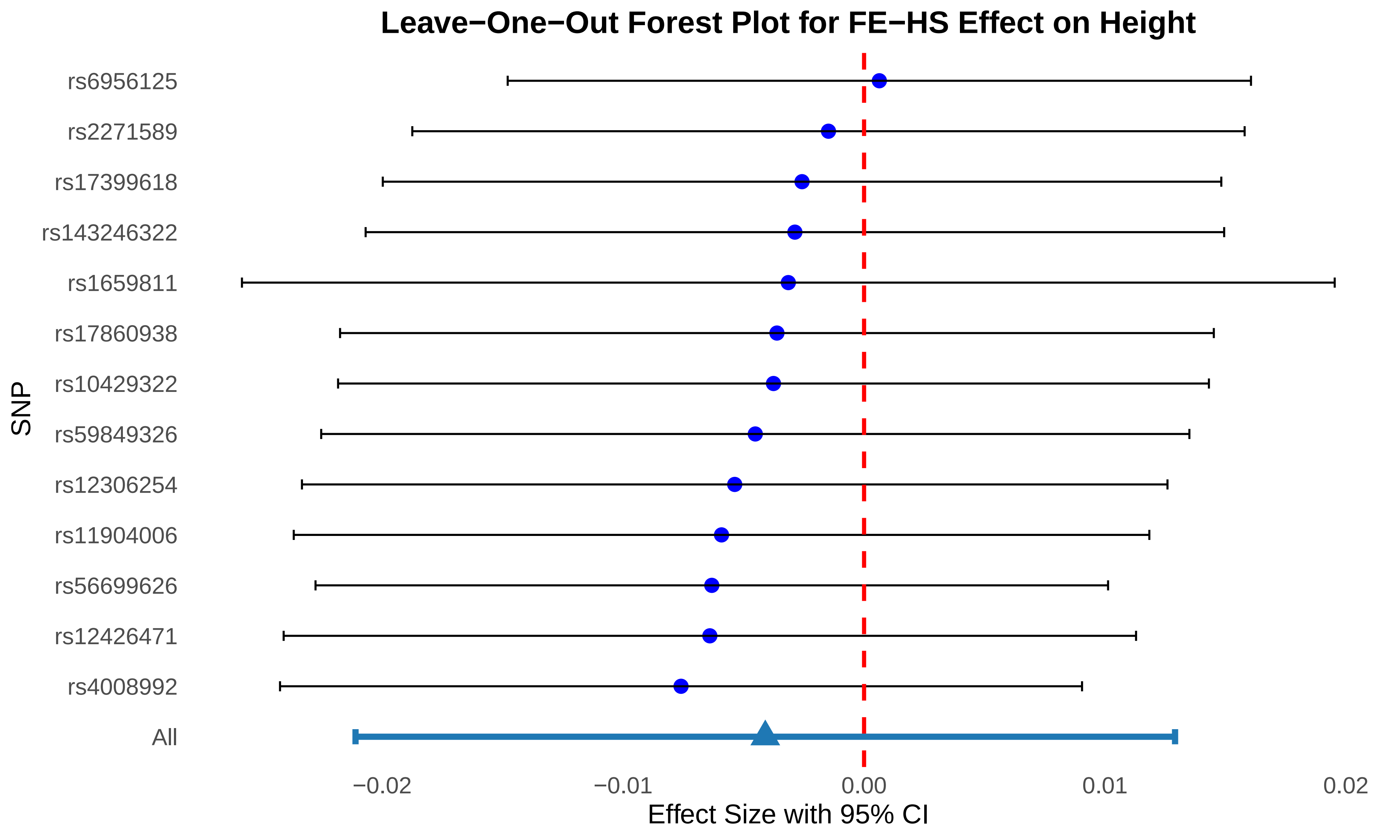
Leave−One−Out Forest Plot for FE−HS Effect on Height
rs6956125
rs2271589
rs17399618
rs143246322
rs1659811
rs17860938
rs10429322
rs59849326
rs12306254
rs11904006
rs56699626
rs12426471
rs4008992
All
SNP
−0.02
−0.01
0.00
0.01
0.02
Effect Size with 95% CI

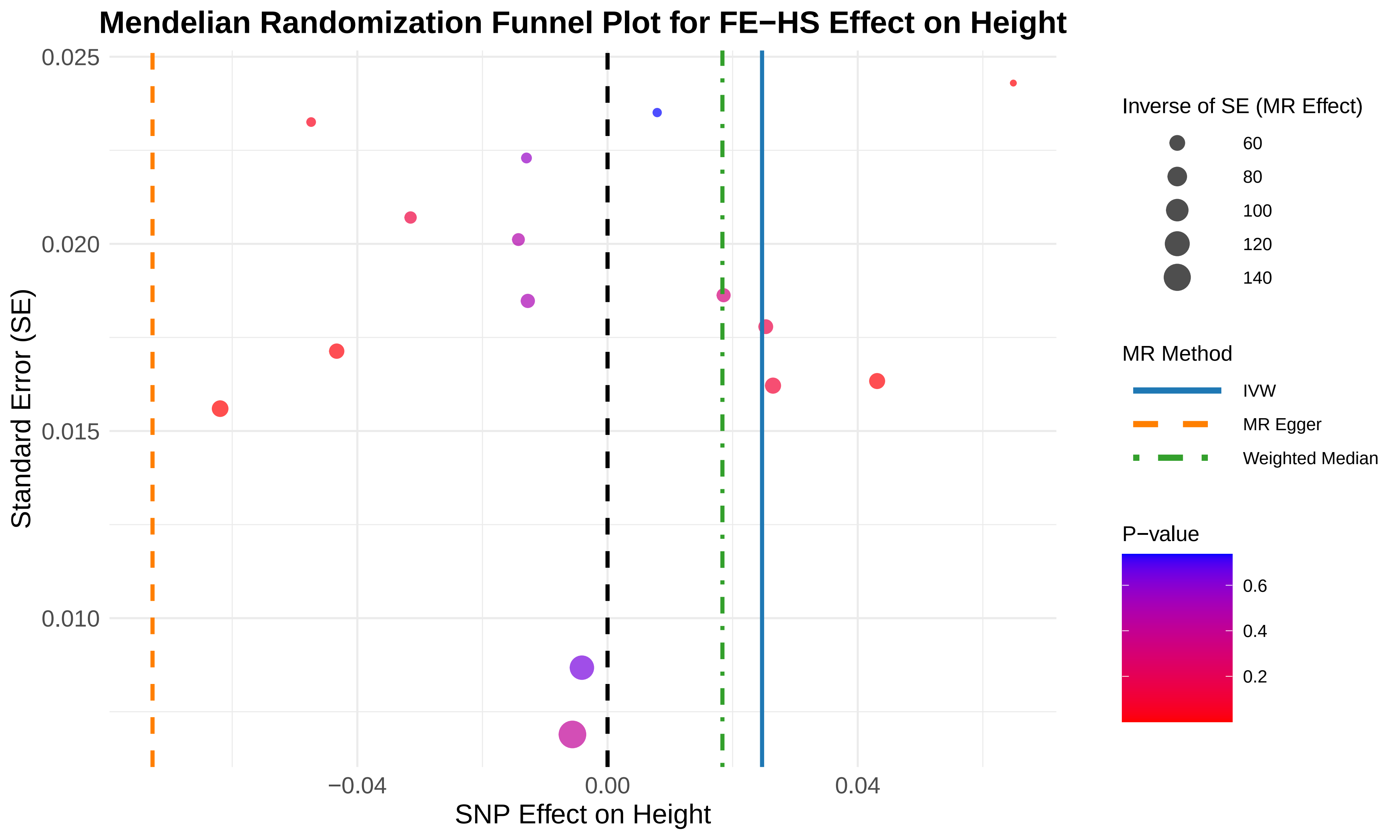

Mendelian Randomization Funnel Plot for FE−HS Effect on Height
Standard Error (SE)
0.025
0.020
0.015
0.010
−0.04
0.00
0.04
SNP Effect on Height
Inverse of SE (MR Effect)
60
80
100
120
140
MR Method
IVW
MR Egger
Weighted Median
P−value
0.6
0.4
0.2

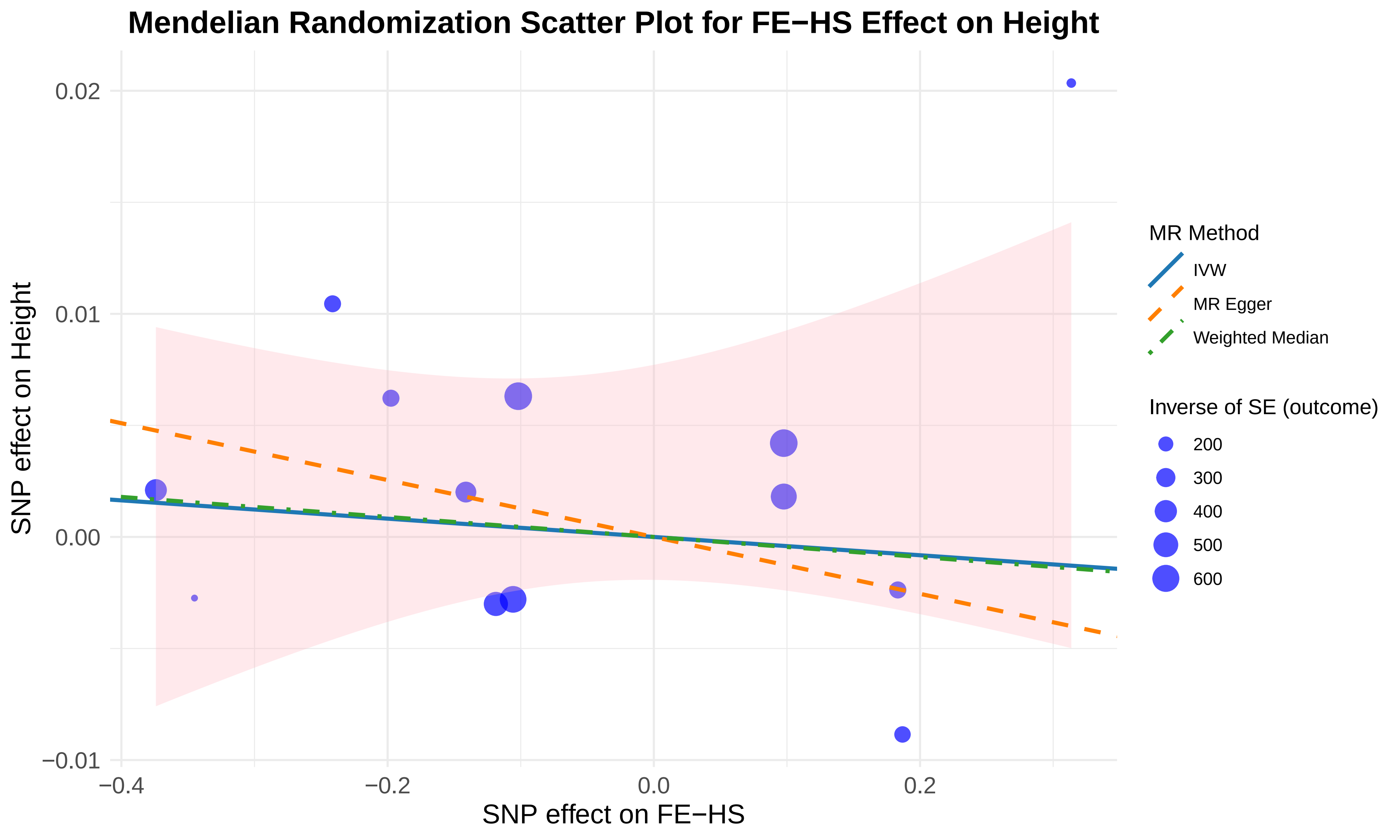

Mendelian Randomization Scatter Plot for FE−HS Effect on Height
SNP effect on Height
SNP effect on FE−HS
0.02
0.01
0.00
−0.01
−0.4
−0.2
0.0
0.2
MR Method
IVW
MR Egger
Weighted Median
Inverse of SE (outcome)
200
300
400
500
600

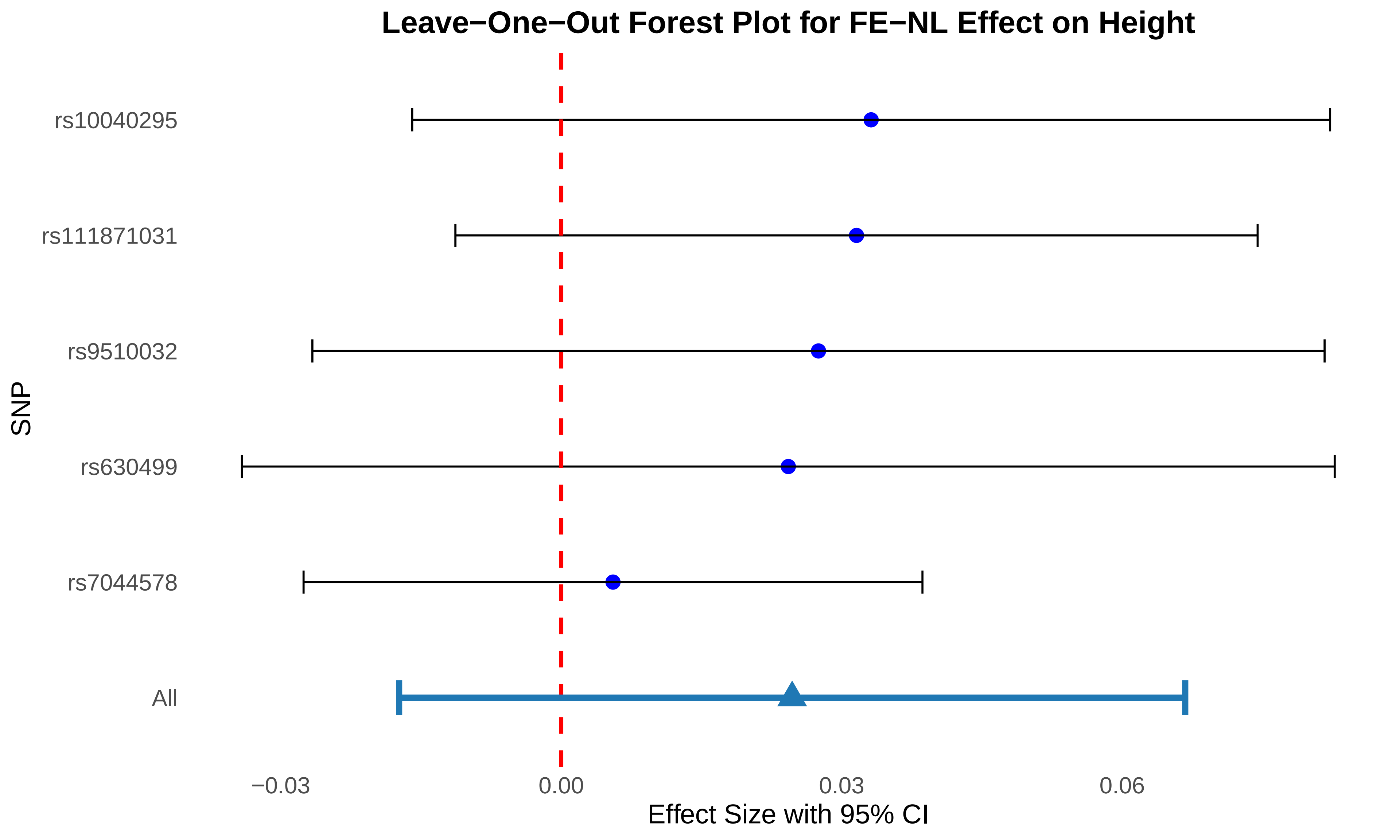
Leave−One−Out Forest Plot for FE−NL Effect on Height
rs10040295
rs111871031
rs9510032
rs630499
rs7044578
All
SNP
−0.03
0.00
0.03
0.06
Effect Size with 95% CI

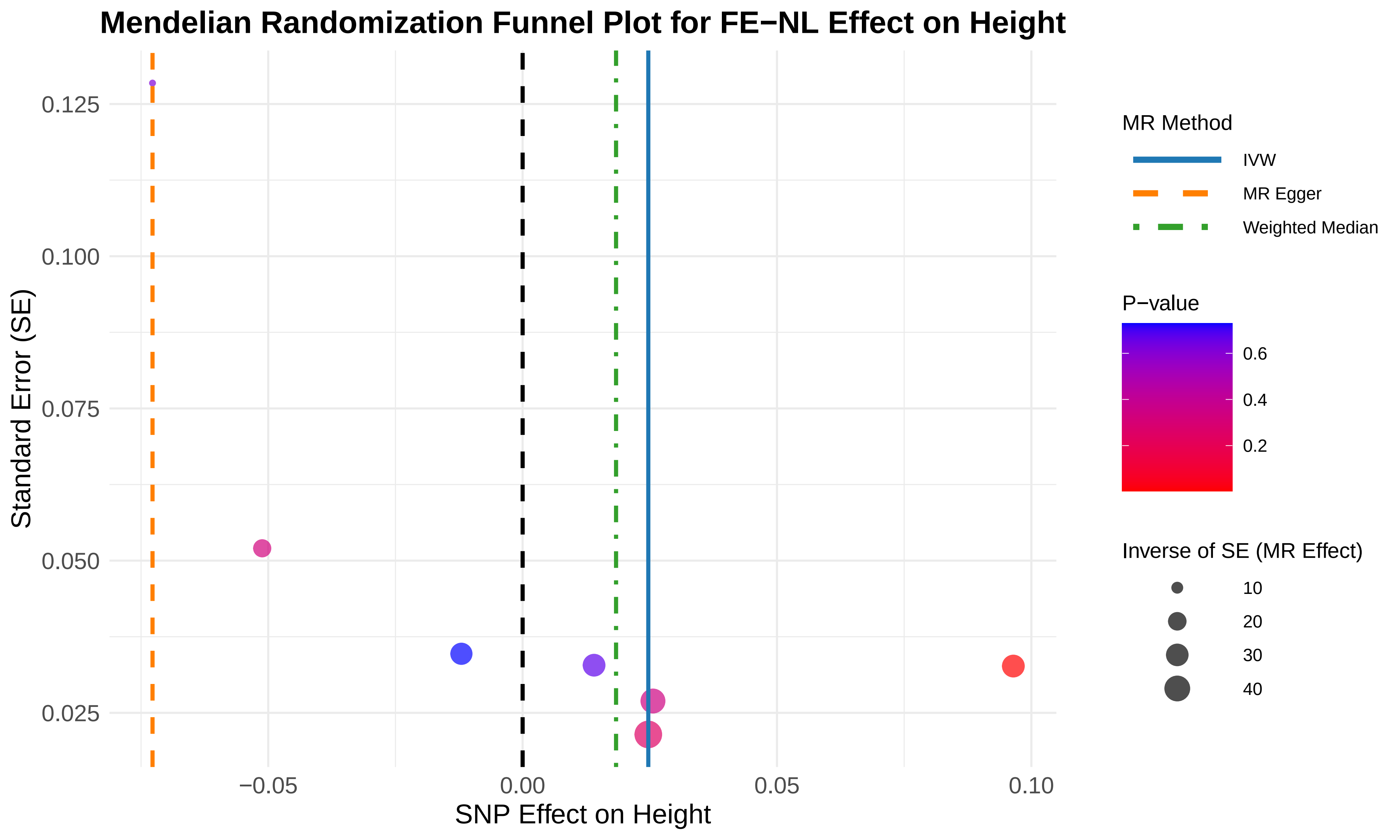
Mendelian Randomization Funnel Plot for FE−NL Effect on Height
Standard Error (SE)
0.125
0.100
0.075
0.050
0.025
−0.05
0.00
0.05
0.10
SNP Effect on Height
MR Method
IVW
MR Egger
Weighted Median
P−value
0.6
0.4
0.2
Inverse of SE (MR Effect)
10
20
30
40

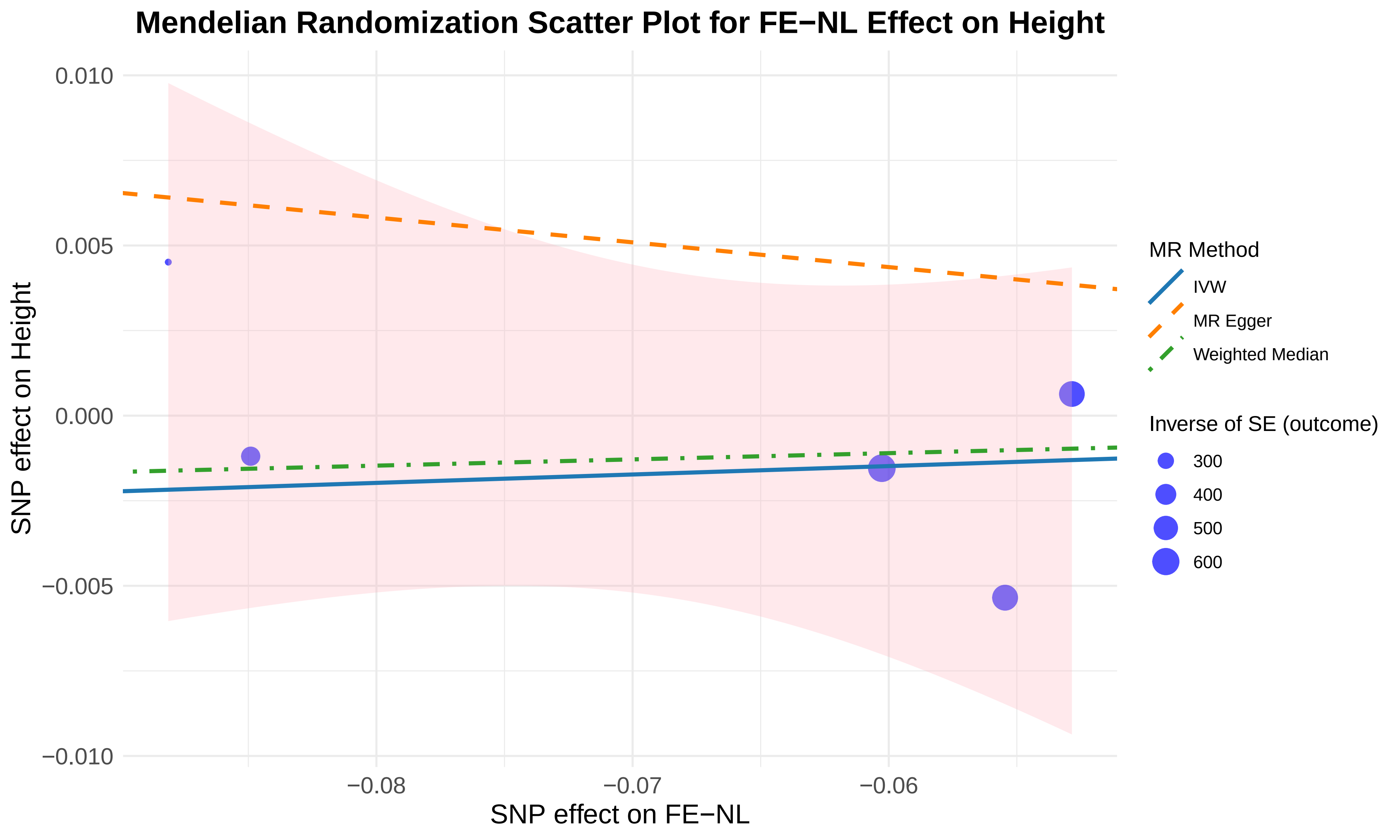
Mendelian Randomization Scatter Plot for FE−NL Effect on Height
0.010
0.005
0.000
−0.005
−0.010
SNP effect on Height
−0.08
−0.07
−0.06
SNP effect on FE−NL
MR Method
IVW
MR Egger
Weighted Median
Inverse of SE (outcome)
300
400
500
600

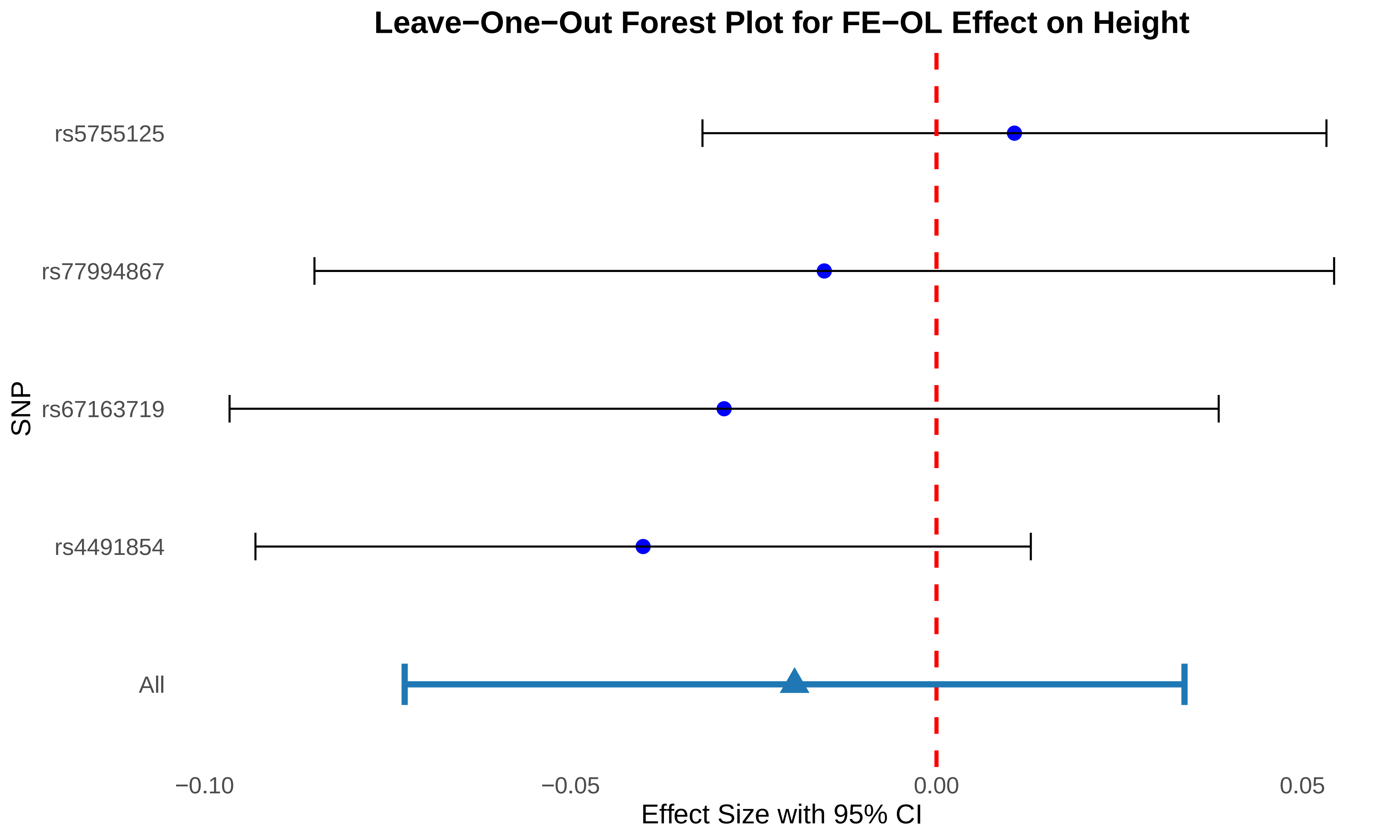
Leave−One−Out Forest Plot for FE−OL Effect on Height
rs5755125
rs77994867
rs67163719
rs4491854
All
SNP
−0.10
−0.05
0.00
0.05
Effect Size with 95% CI

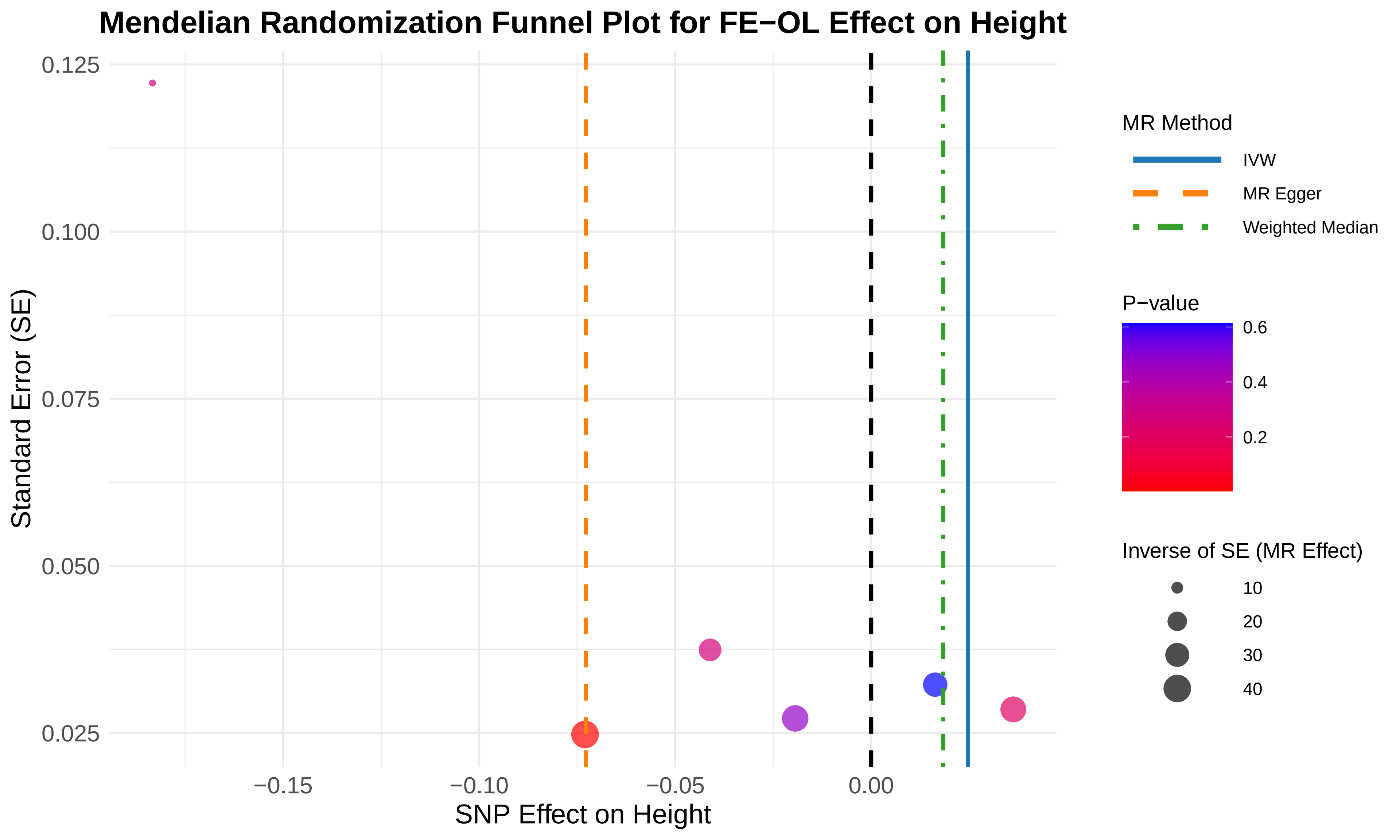
Mendelian Randomization Funnel Plot for FE−OL Effect on Height
Standard Error (SE)
0.125
0.100
0.075
0.050
0.025
−0.15
−0.10
−0.05
0.00
SNP Effect on Height
MR Method
IVW
MR Egger
Weighted Median
P−value
0.6
0.4
0.2
Inverse of SE (MR Effect)
10
20
30
40

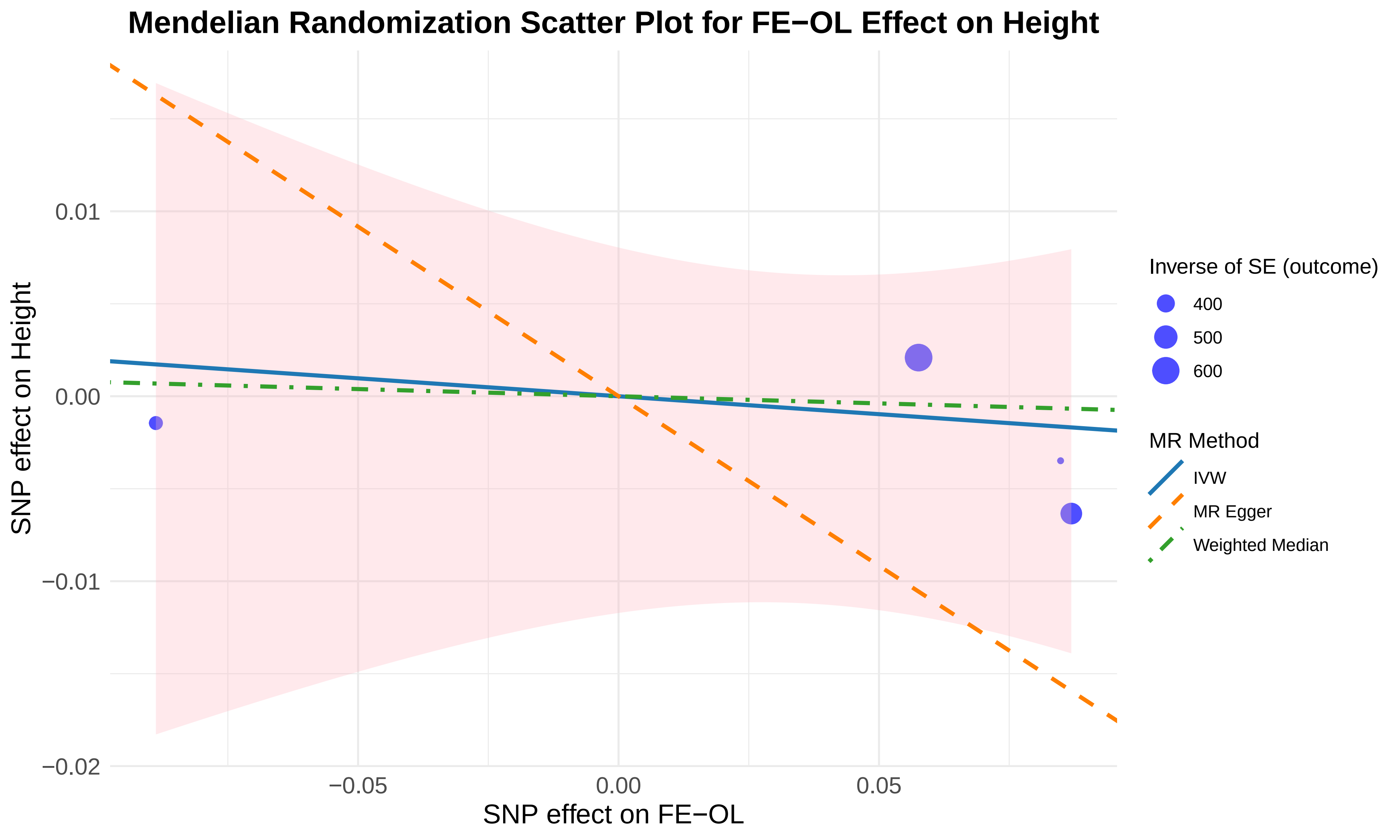
Mendelian Randomization Scatter Plot for FE−OL Effect on Height
SNP effect on Height
SNP effect on FE−OL
0.01
0.00
−0.01
−0.02
−0.05
0.00
0.05
Inverse of SE (outcome)
400
500
600
MR Method
IVW
MR Egger
Weighted Median

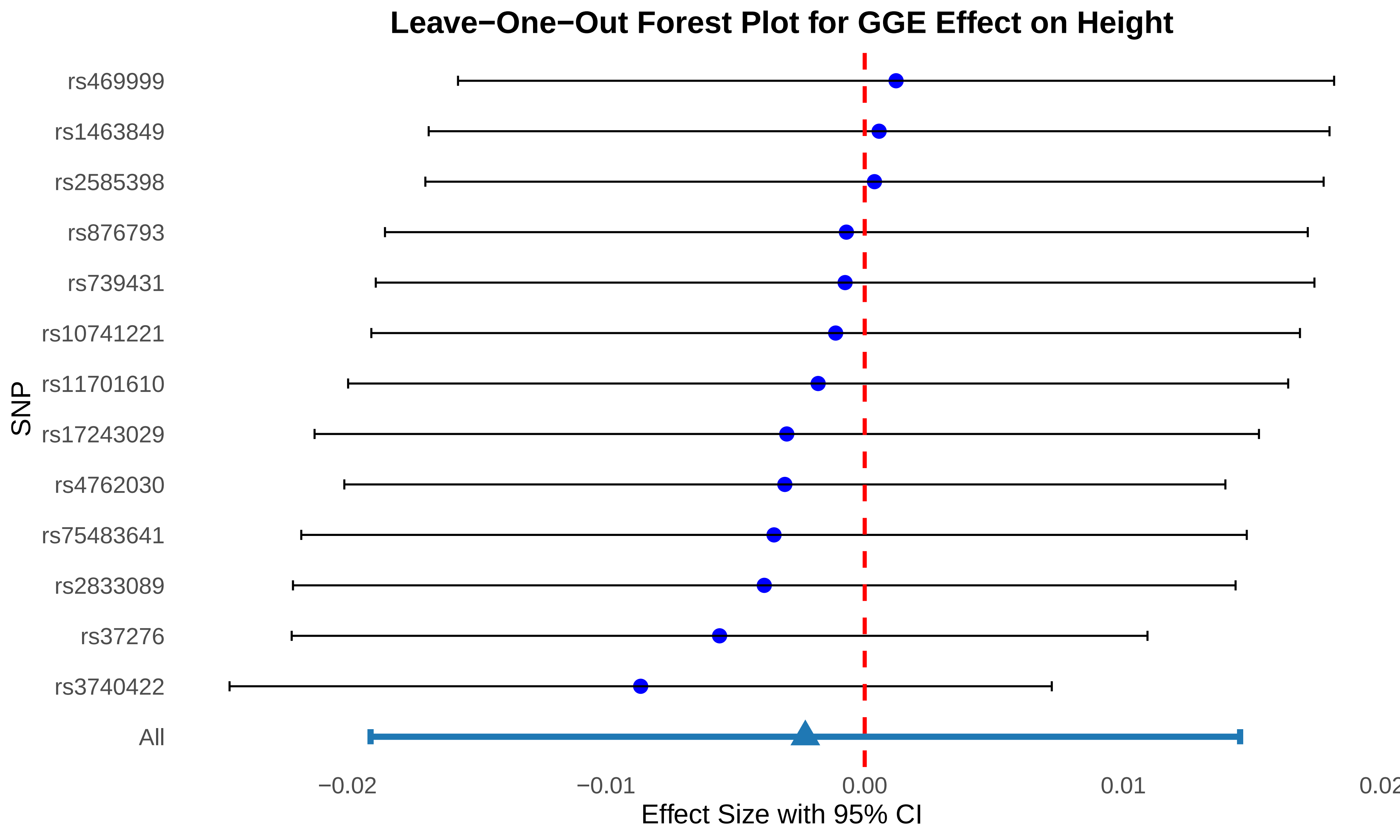
Leave−One−Out Forest Plot for GGE Effect on Height
SNP
rs469999
rs1463849
rs2585398
rs876793
rs739431
rs10741221
rs11701610
rs17243029
rs4762030
rs75483641
rs2833089
rs37276
rs3740422
All
−0.02
−0.01
0.00
0.01
0.02
Effect Size with 95% CI

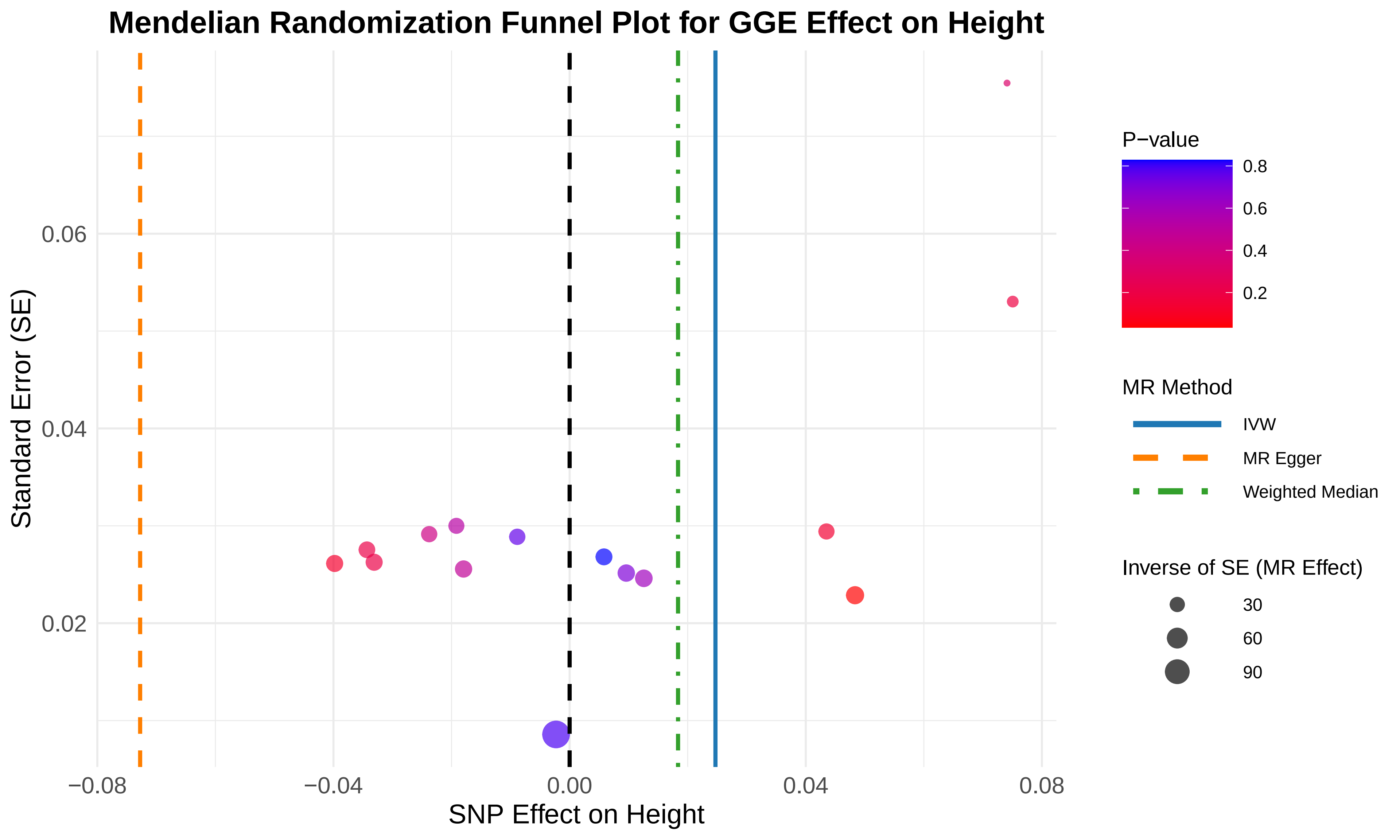

Mendelian Randomization Funnel Plot for GGE Effect on Height
Standard Error (SE)
0.06
0.04
0.02
−0.08
−0.04
0.00
0.04
0.08
SNP Effect on Height
P−value
0.8
0.6
0.4
0.2
MR Method
IVW
MR Egger
Weighted Median
Inverse of SE (MR Effect)
30
60
90

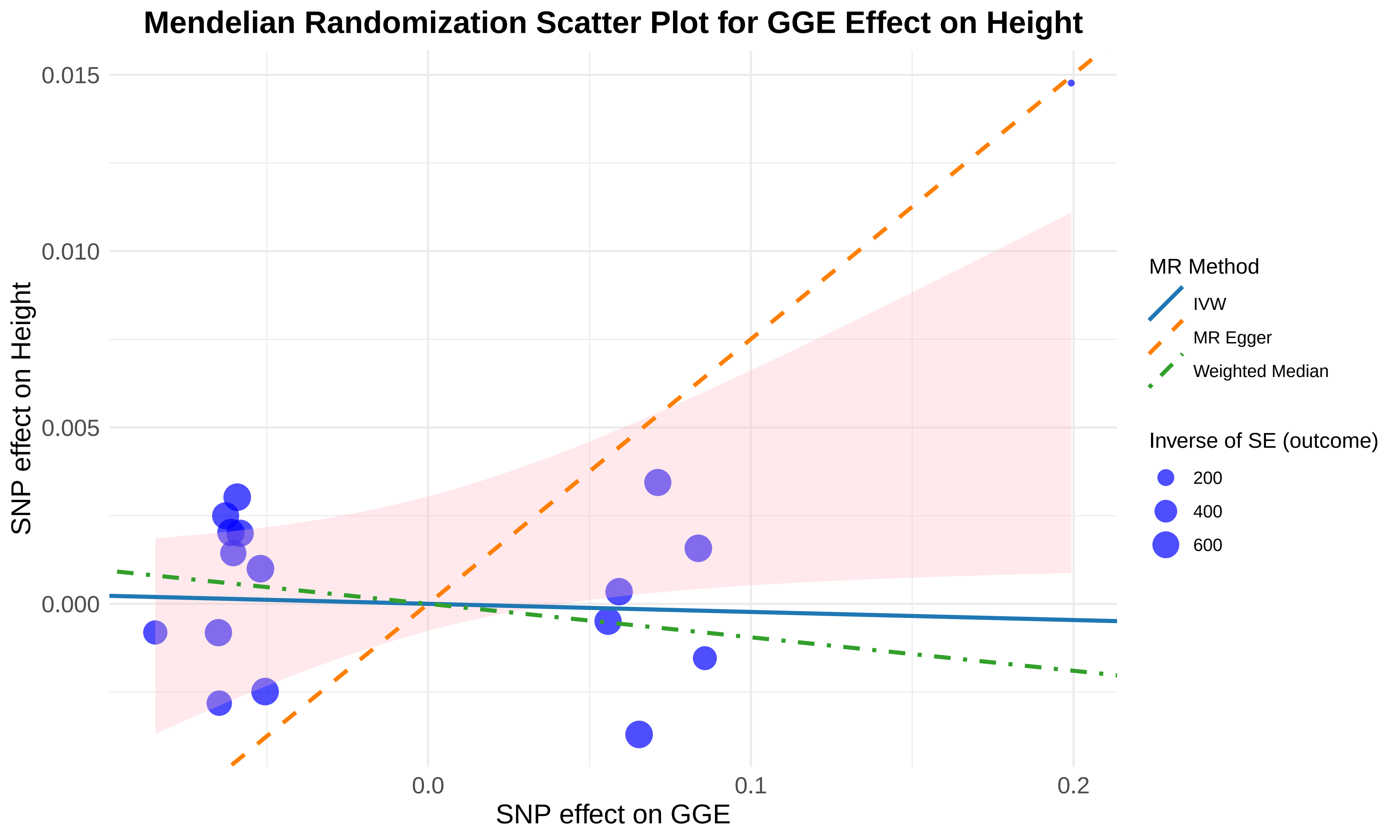
Mendelian Randomization Scatter Plot for GGE Effect on Height
SNP effect on Height
0.015
0.010
0.005
0.000
0.0
0.1
0.2
SNP effect on GGE
MR Method
IVW
MR Egger
Weighted Median
Inverse of SE (outcome)
200
400
600

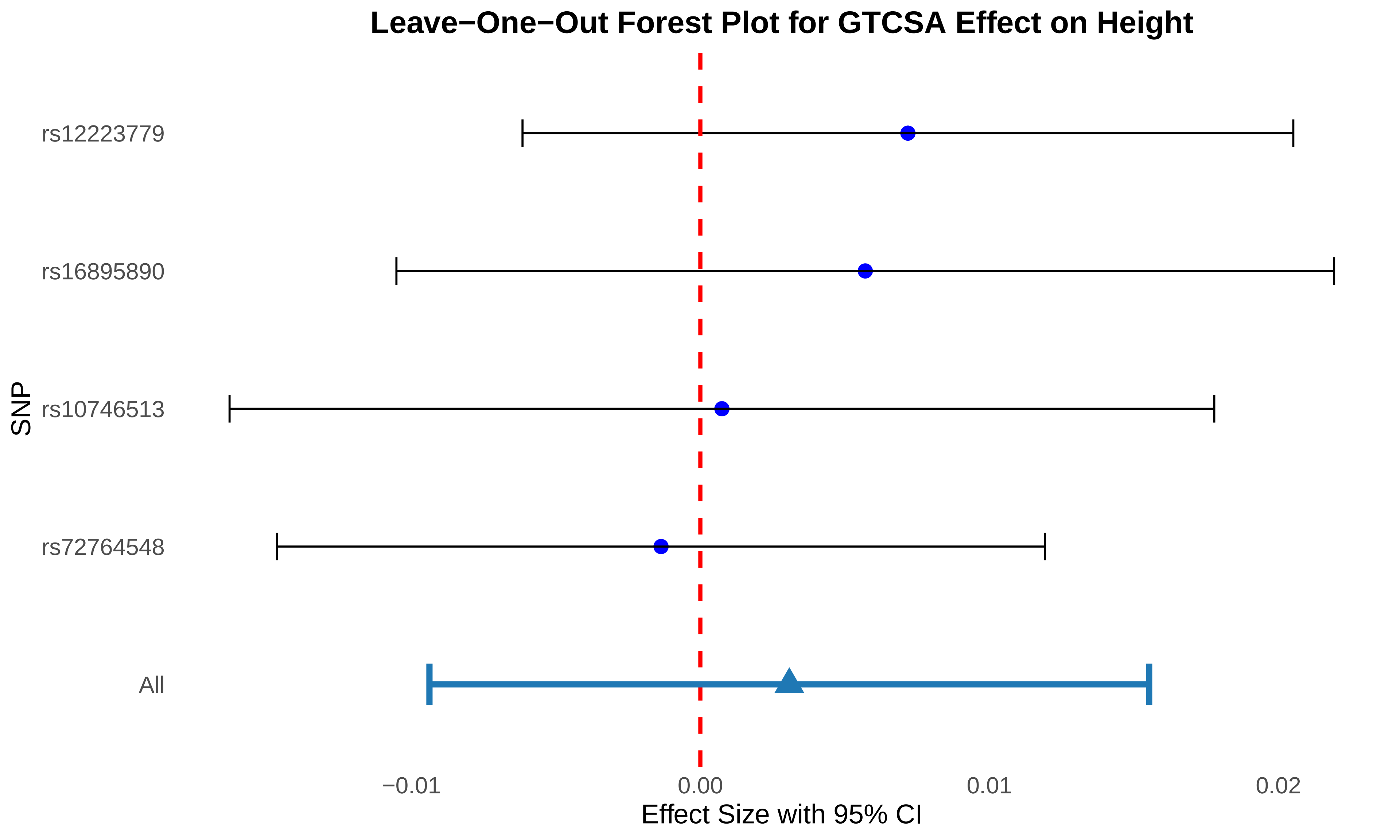
Leave−One−Out Forest Plot for GTCSA Effect on Height
SNP
rs12223779
rs16895890
rs10746513
rs72764548
All
−0.01
0.00
0.01
0.02
Effect Size with 95% CI

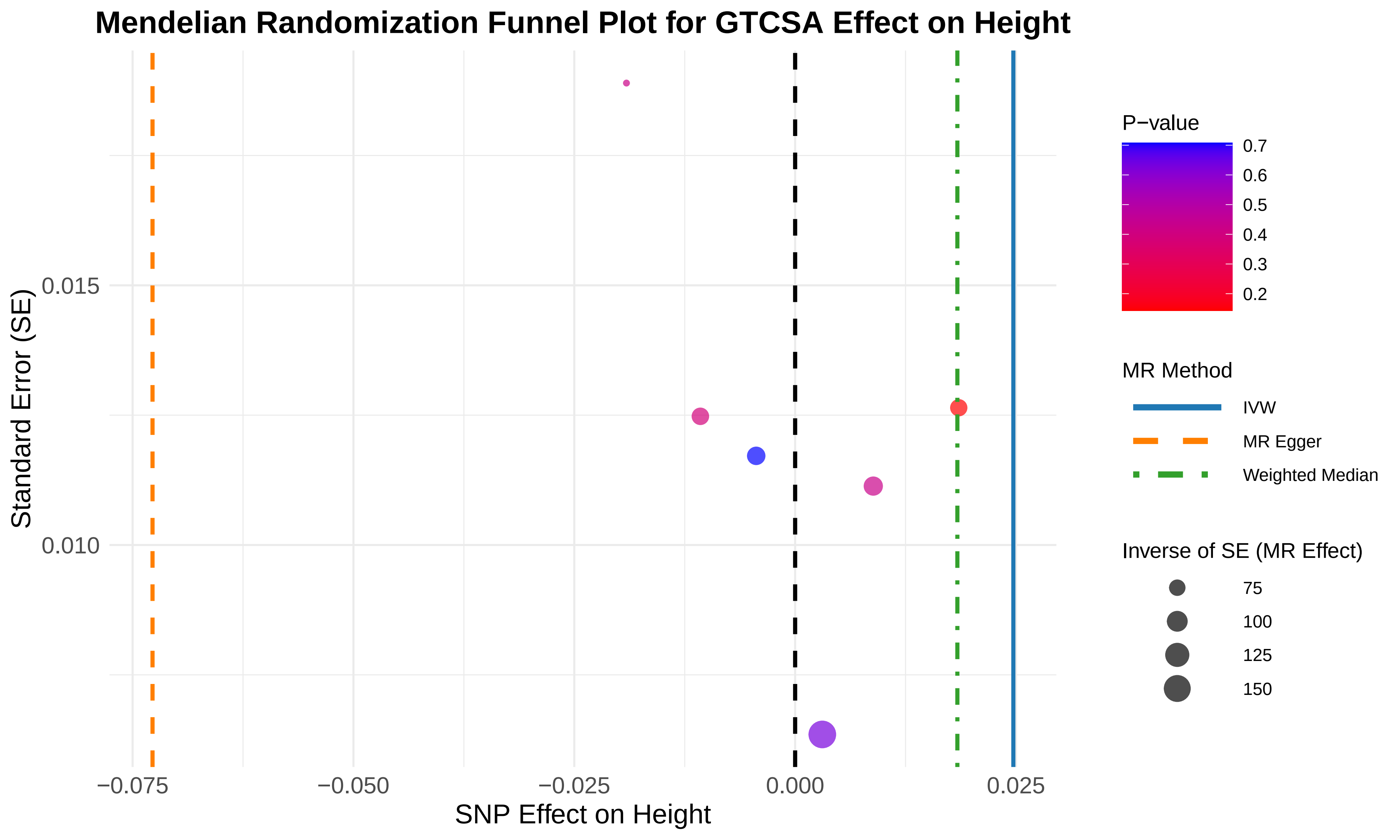

Mendelian Randomization Funnel Plot for GTCSA Effect on Height
Standard Error (SE)
0.015
0.010
−0.075
−0.050
−0.025
0.000
0.025
SNP Effect on Height
P−value
0.7
0.6
0.5
0.4
0.3
0.2
MR Method
IVW
MR Egger
Weighted Median
Inverse of SE (MR Effect)
75
100
125
150

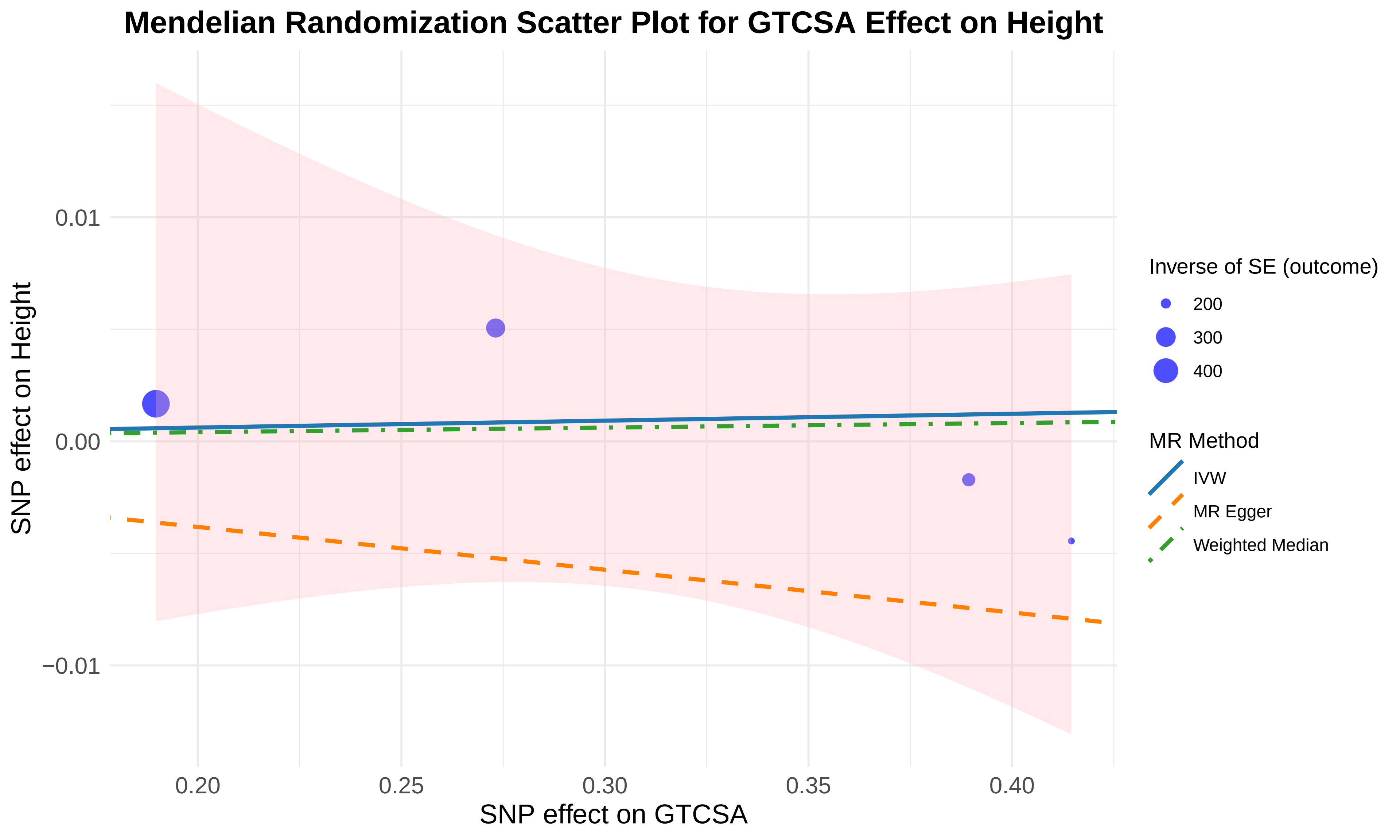
Mendelian Randomization Scatter Plot for GTCSA Effect on Height
SNP effect on Height
0.01
0.00
−0.01
0.20
0.25
0.30
0.35
0.40
SNP effect on GTCSA
Inverse of SE (outcome)
200
300
400
MR Method
IVW
MR Egger
Weighted Median

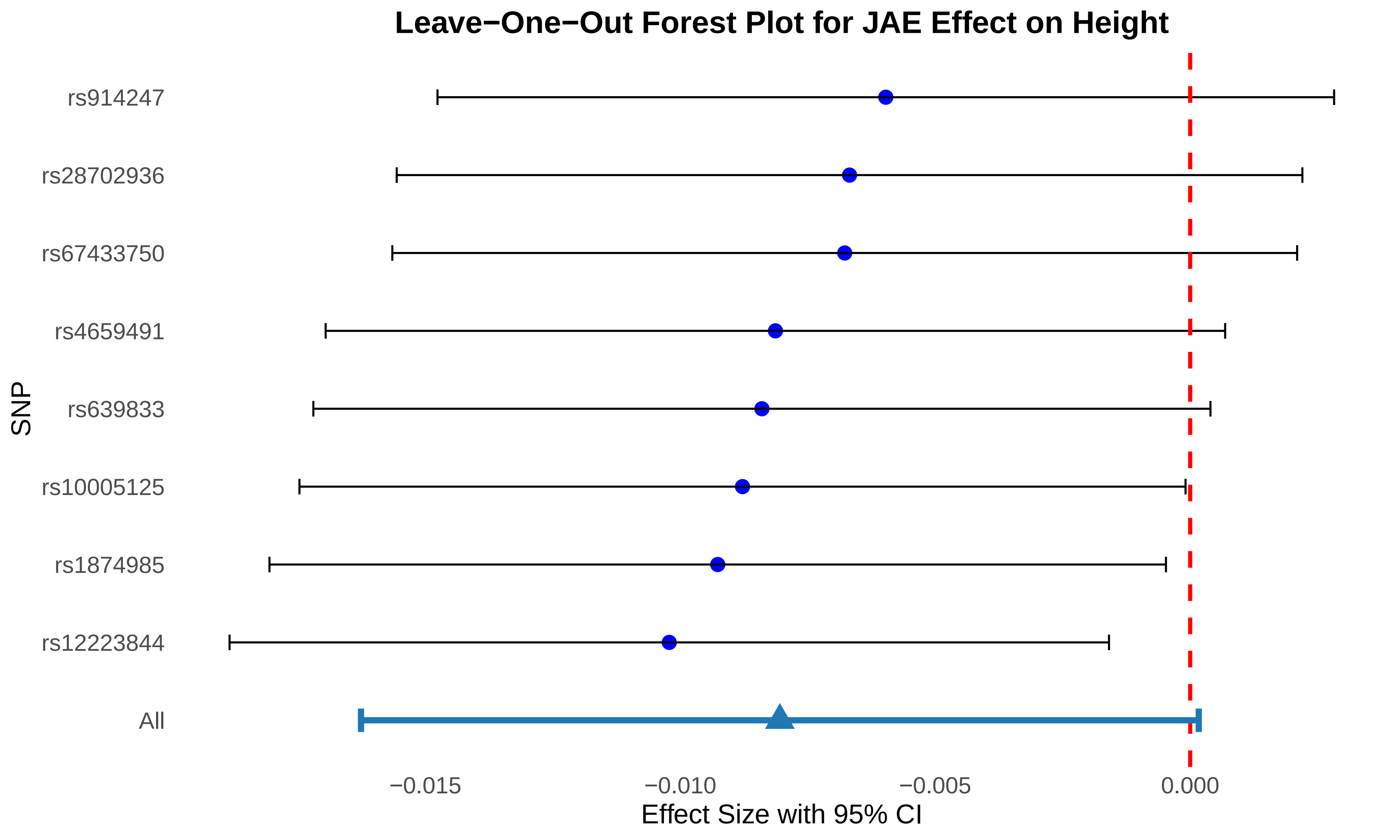
Leave−One−Out Forest Plot for JAE Effect on Height
rs914247
rs28702936
rs67433750
rs4659491
rs639833
rs10005125
rs1874985
rs12223844
All
SNP
−0.015
−0.010
−0.005
0.000
Effect Size with 95% CI

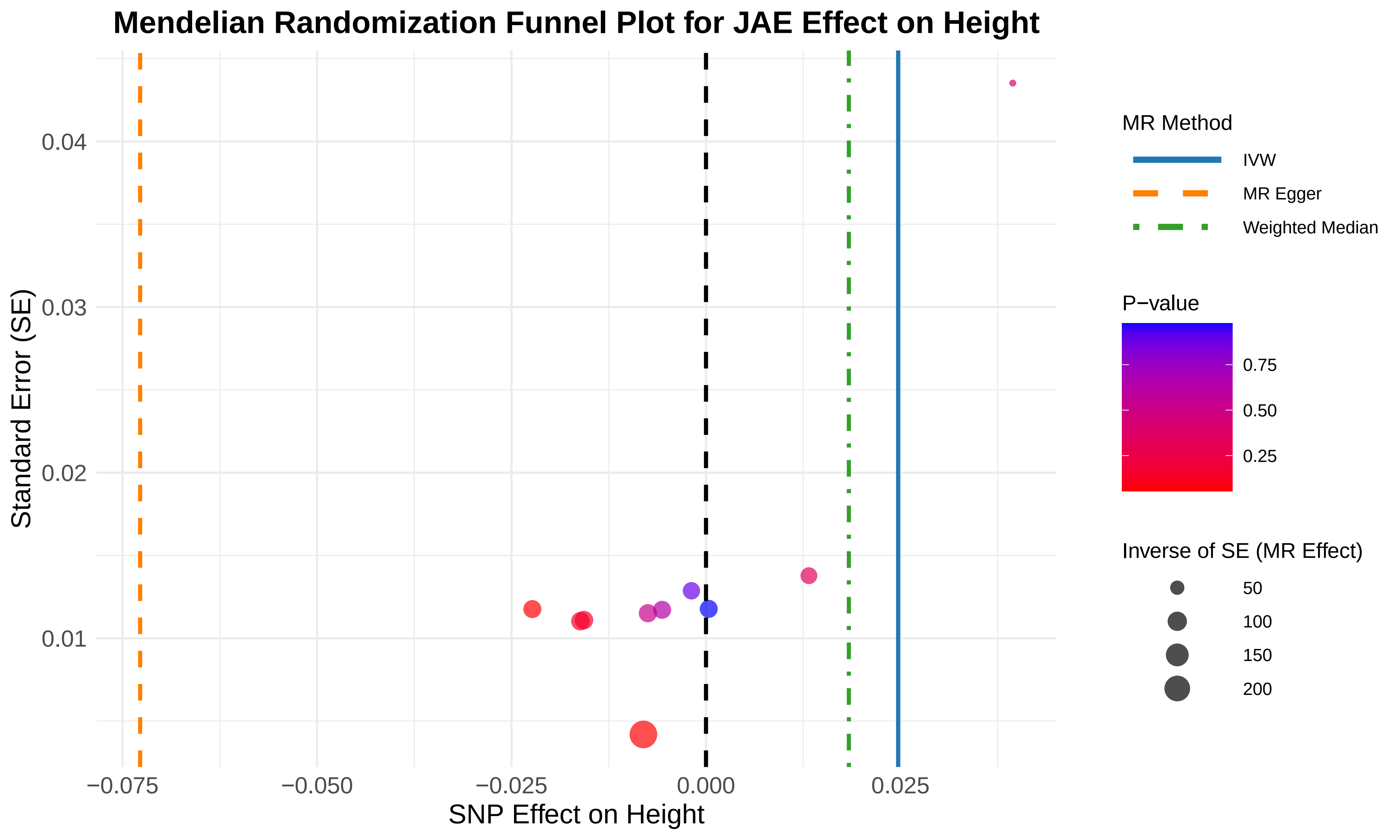

Mendelian Randomization Funnel Plot for JAE Effect on Height
Standard Error (SE)
0.04
0.03
0.02
0.01
−0.075
−0.050
−0.025
0.000
0.025
SNP Effect on Height
MR Method
IVW
MR Egger
Weighted Median
P−value
0.75
0.50
0.25
Inverse of SE (MR Effect)
50
100
150
200

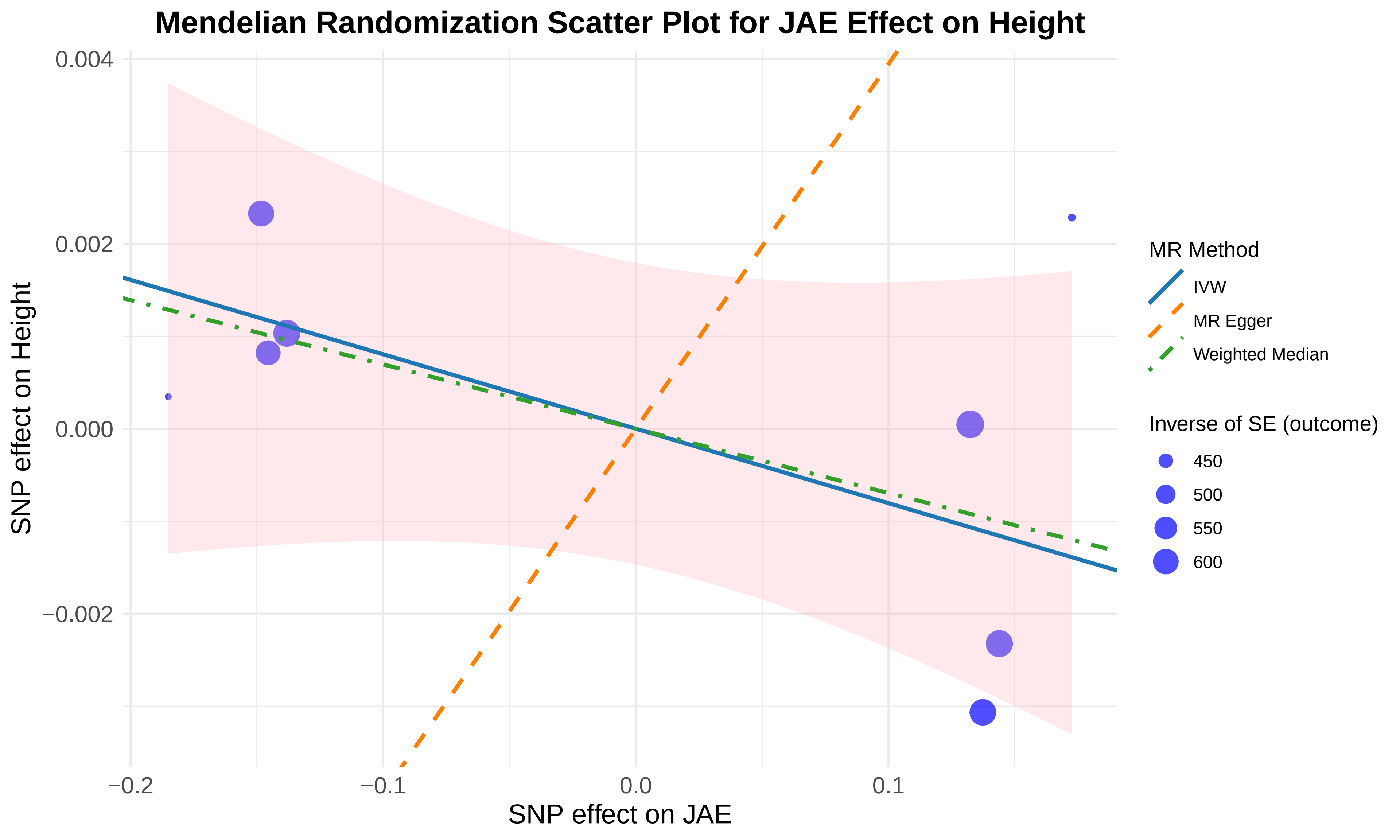
Mendelian Randomization Scatter Plot for JAE Effect on Height
0.004
0.002
0.000
−0.002
SNP effect on Height
−0.2
−0.1
0.0
0.1
SNP effect on JAE
MR Method
IVW
MR Egger
Weighted Median
Inverse of SE (outcome)
450
500
550
600

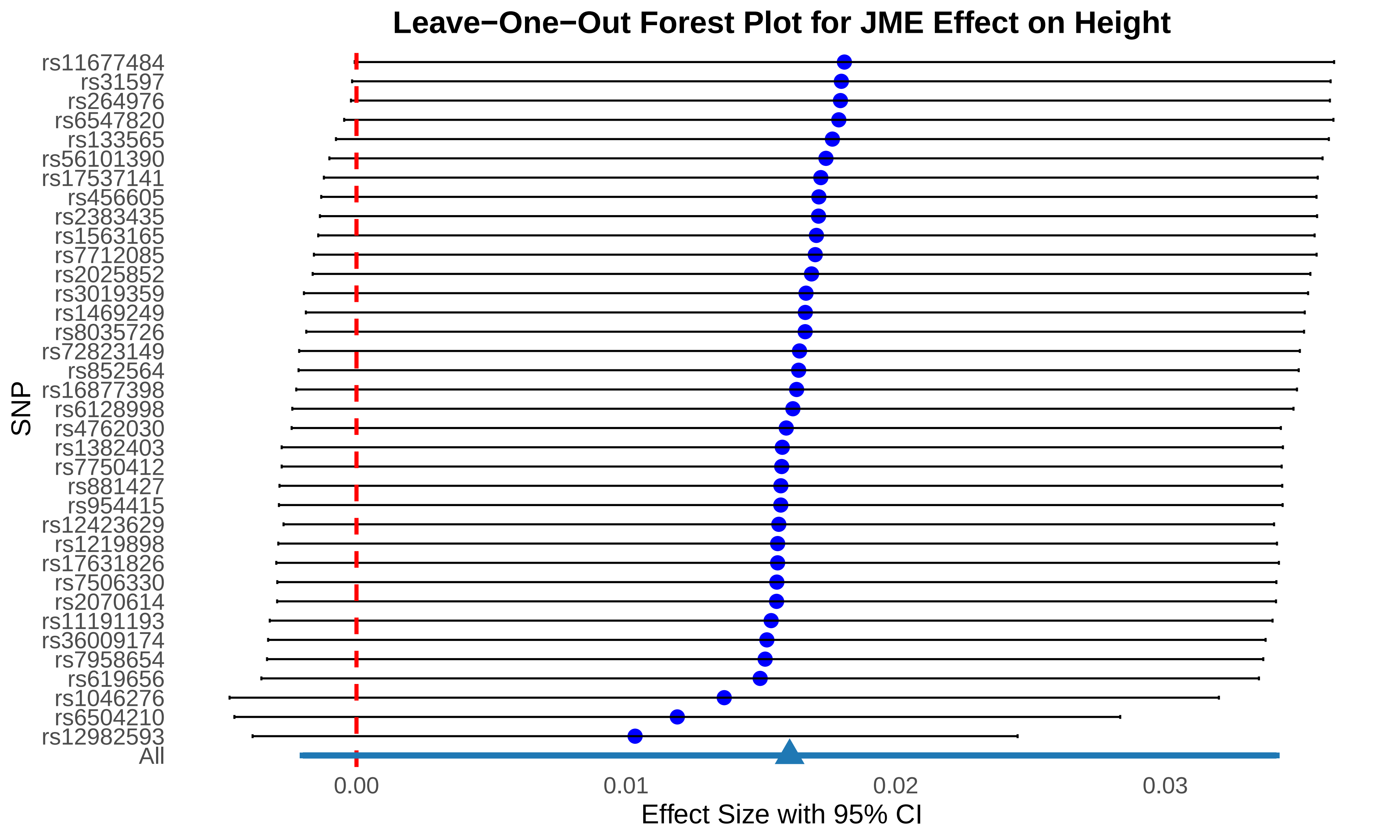

Leave−One−Out Forest Plot for JME Effect on Height
SNP
rs11677484
rs31597
rs264976
rs6547820
rs133565
rs56101390
rs17537141
rs456605
rs2383435
rs1563165
rs7712085
rs2025852
rs3019359
rs1469249
rs8035726
rs72823149
rs852564
rs16877398
rs6128998
rs4762030
rs1382403
rs7750412
rs881427
rs954415
rs12423629
rs1219898
rs17631826
rs7506330
rs2070614
rs11191193
rs36009174
rs7958654
rs619656
rs1046276
rs6504210
rs12982593
All
0.00
0.01
0.02
0.03
Effect Size with 95% CI

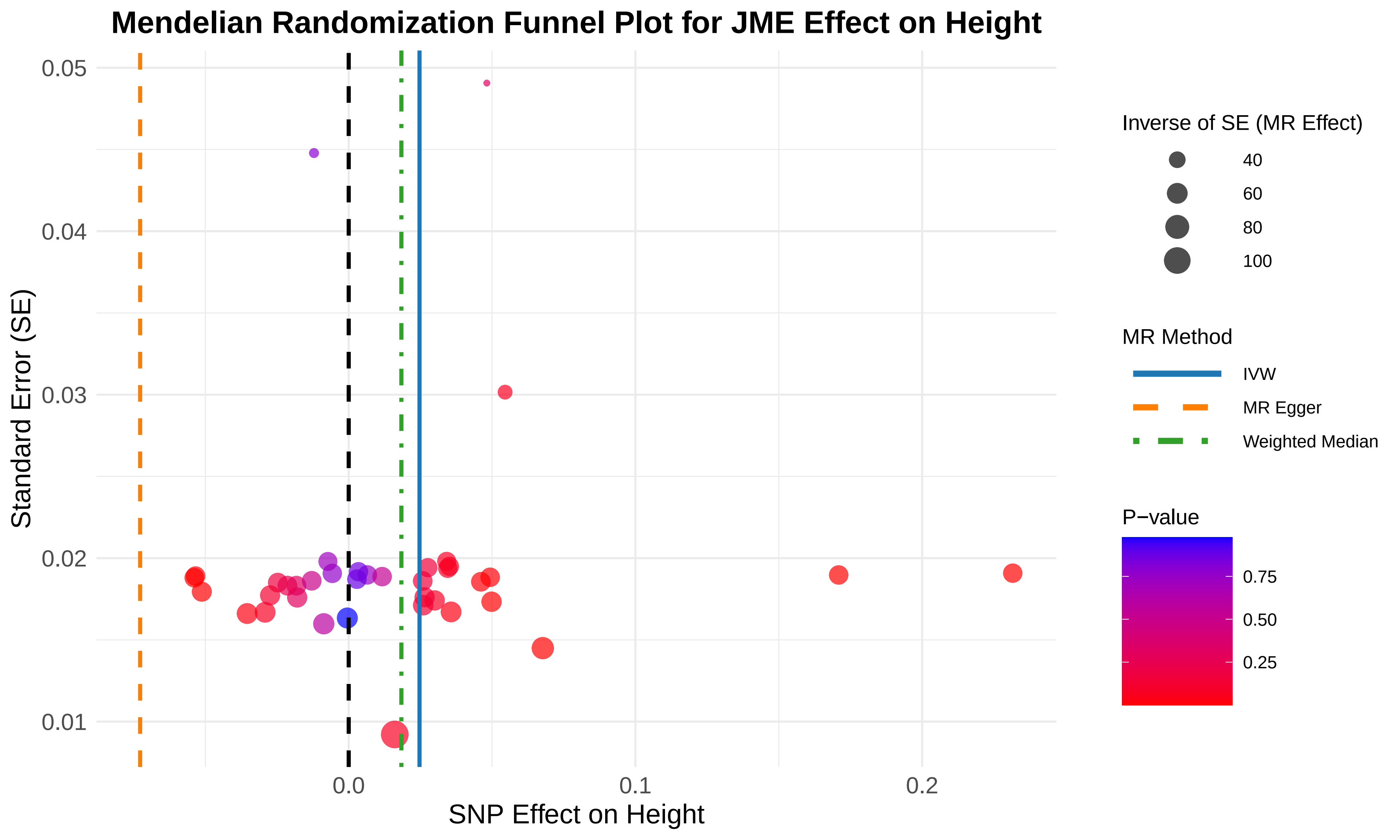
Mendelian Randomization Funnel Plot for JME Effect on Height
Standard Error (SE)
0.05
0.04
0.03
0.02
0.01
0.0
0.1
0.2
SNP Effect on Height
Inverse of SE (MR Effect)
40
60
80
100
MR Method
IVW
MR Egger
Weighted Median
P−value
0.75
0.50
0.25

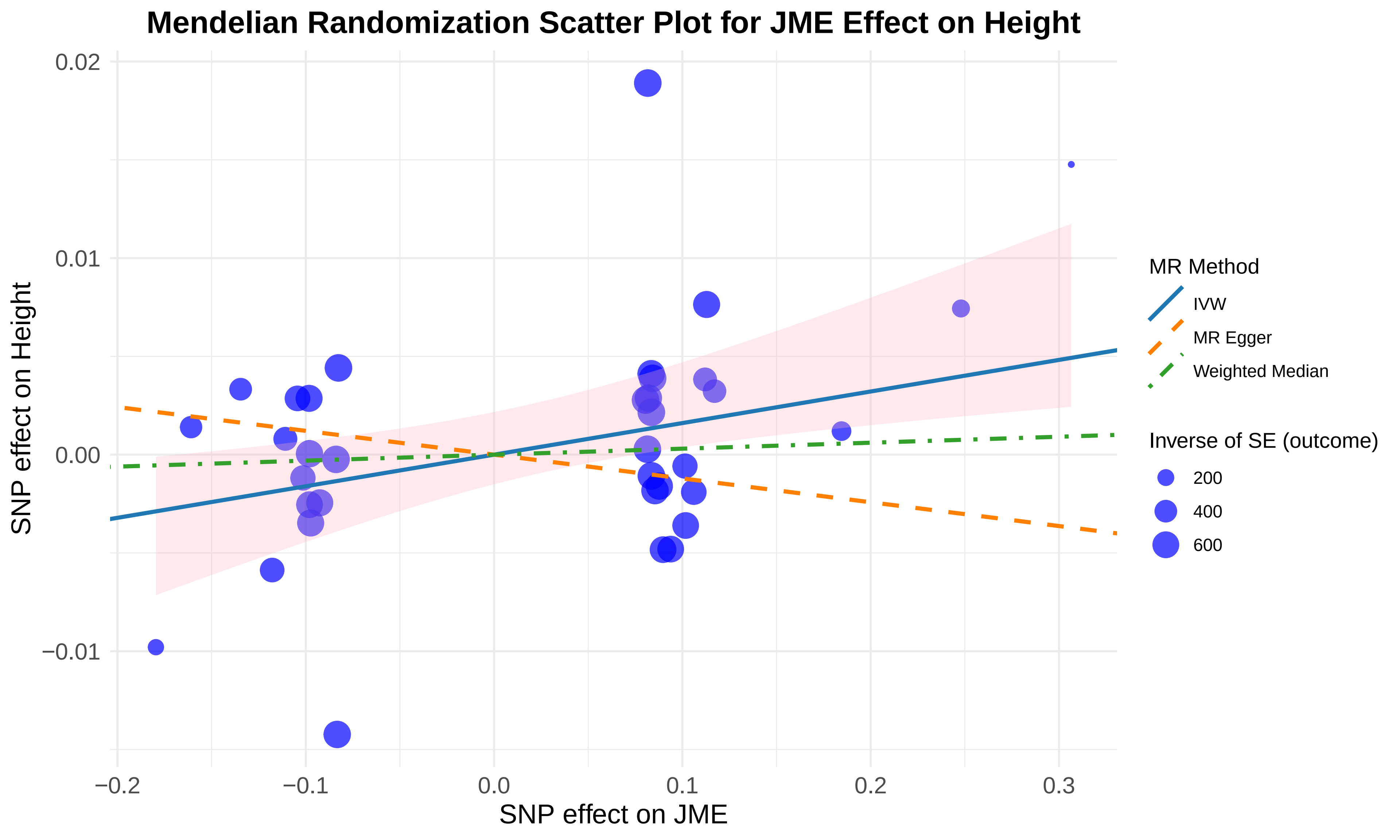
Mendelian Randomization Scatter Plot for JME Effect on Height
SNP effect on Height
0.02
0.01
0.00
−0.01
−0.2
−0.1
0.0
0.1
0.2
0.3
SNP effect on JME
MR Method
IVW
MR Egger
Weighted Median
Inverse of SE (outcome)
200
400
600

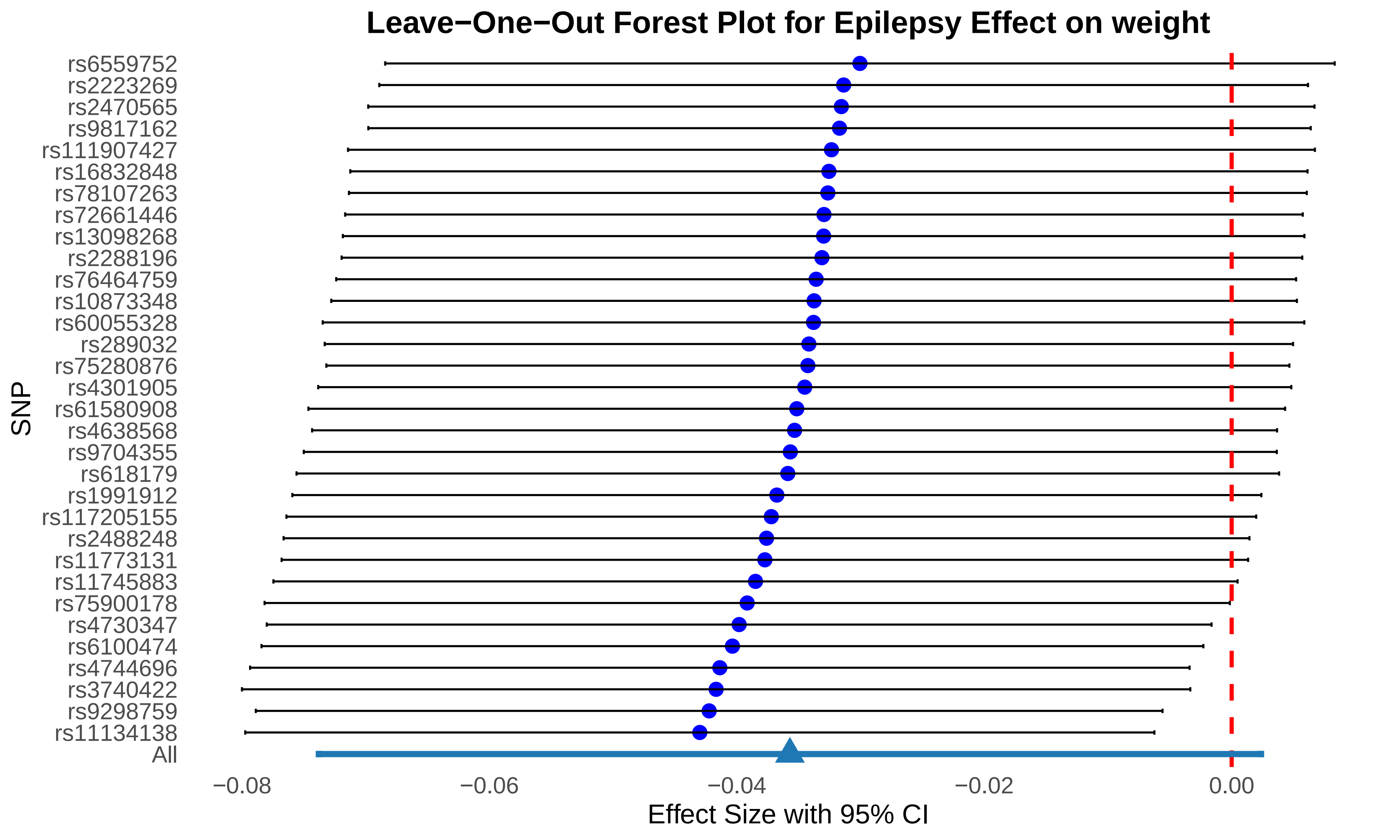

Leave−One−Out Forest Plot for Epilepsy Effect on weight
SNP
rs6559752
rs2223269
rs2470565
rs9817162
rs111907427
rs16832848
rs78107263
rs72661446
rs13098268
rs2288196
rs76464759
rs10873348
rs60055328
rs289032
rs75280876
rs4301905
rs61580908
rs4638568
rs9704355
rs618179
rs1991912
rs117205155
rs2488248
rs11773131
rs11745883
rs75900178
rs4730347
rs6100474
rs4744696
rs3740422
rs9298759
rs11134138
All
−0.08
−0.06
−0.04
−0.02
0.00
Effect Size with 95% CI

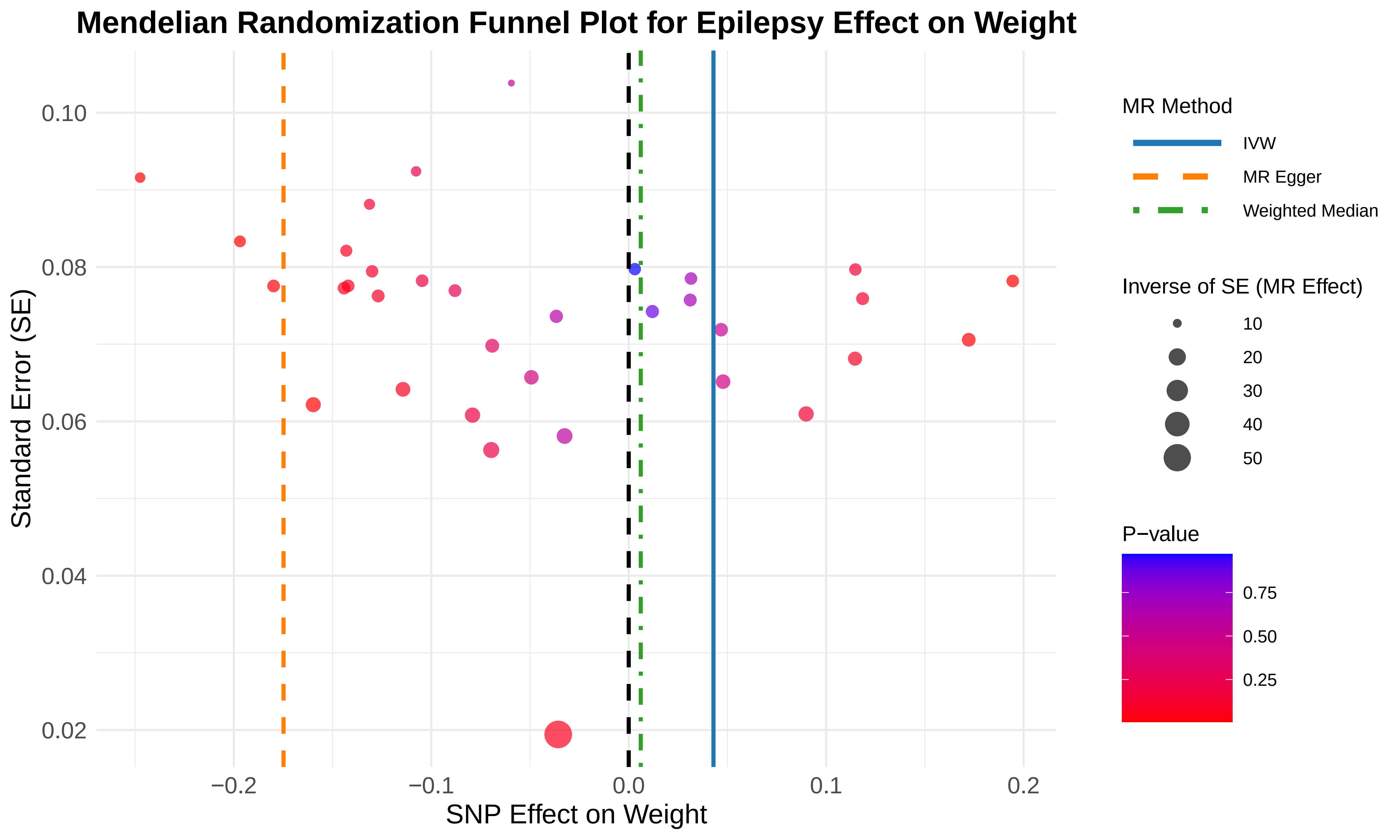

Mendelian Randomization Funnel Plot for Epilepsy Effect on Weight
Standard Error (SE)
0.10
0.08
0.06
0.04
0.02
−0.2
−0.1
0.0
0.1
0.2
SNP Effect on Weight
MR Method
IVW
MR Egger
Weighted Median
Inverse of SE (MR Effect)
10
20
30
40
50
P−value
0.75
0.50
0.25

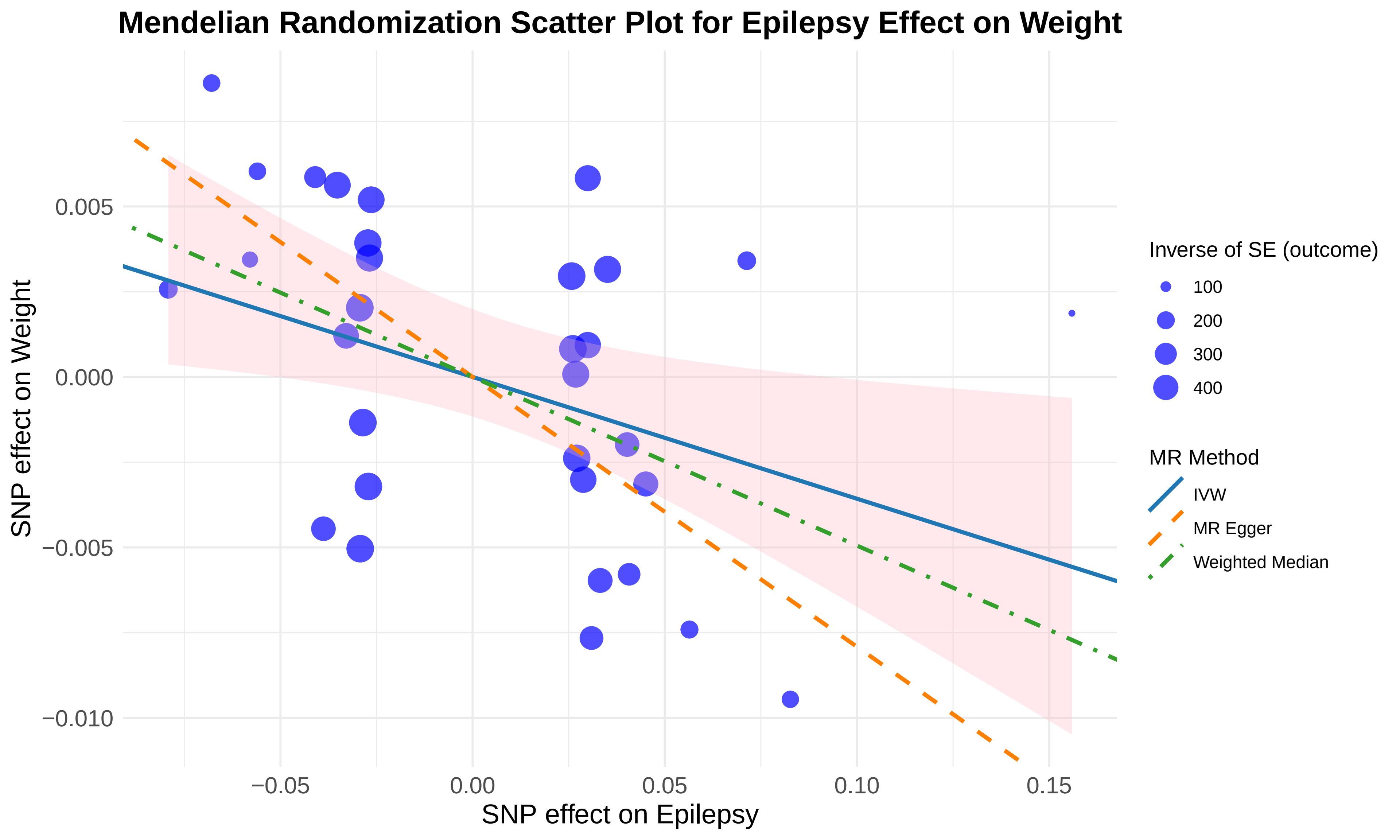

Mendelian Randomization Scatter Plot for Epilepsy Effect on Weight
SNP effect on Weight
SNP effect on Epilepsy
0.005
0.000
−0.005
−0.010
−0.05
0.00
0.05
0.10
0.15
Inverse of SE (outcome)
100
200
300
400
MR Method
IVW
MR Egger
Weighted Median

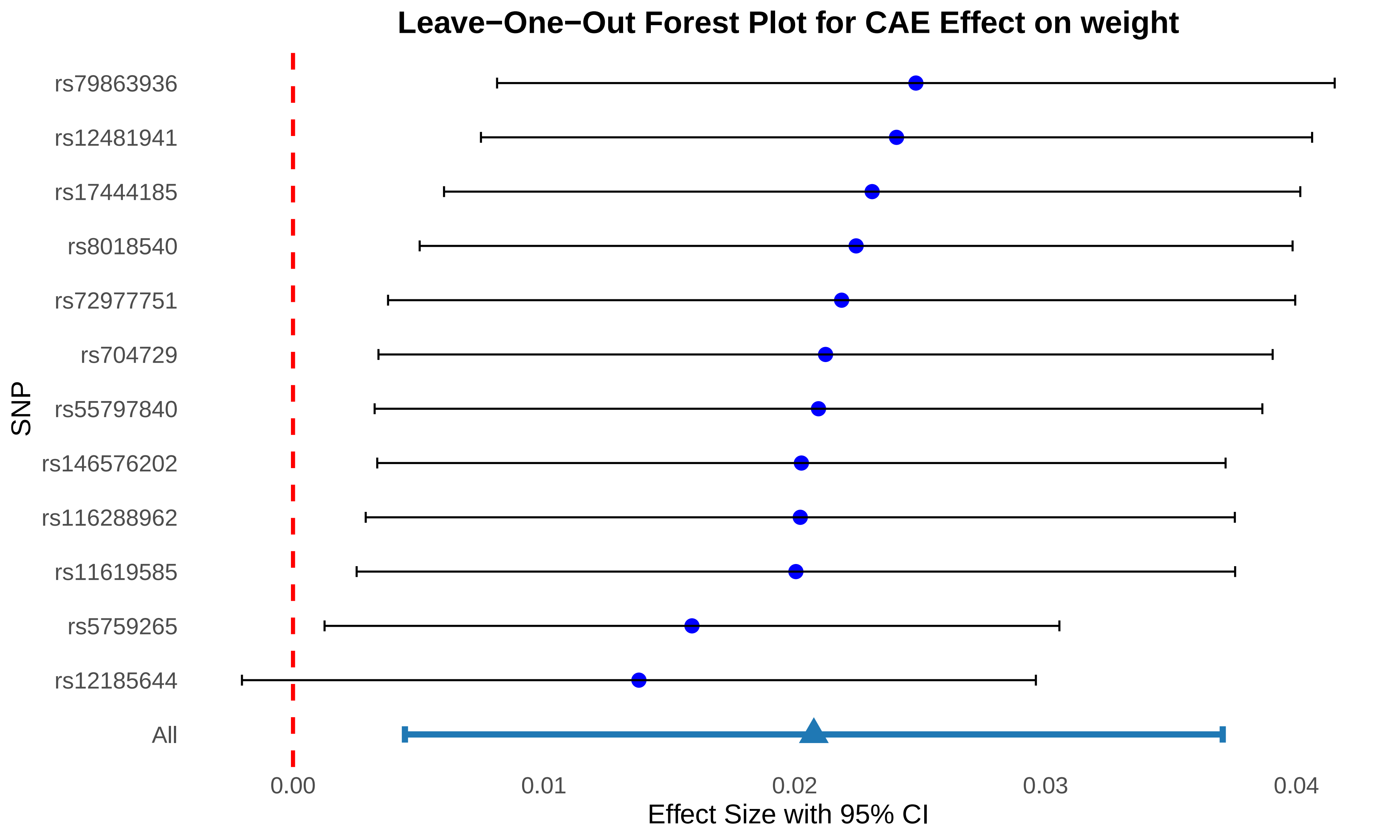

Leave−One−Out Forest Plot for CAE Effect on weight
rs79863936
rs12481941
rs17444185
rs8018540
rs72977751
rs704729
rs55797840
rs146576202
rs116288962
rs11619585
rs5759265
rs12185644
All
SNP
0.00
0.01
0.02
0.03
0.04
Effect Size with 95% CI

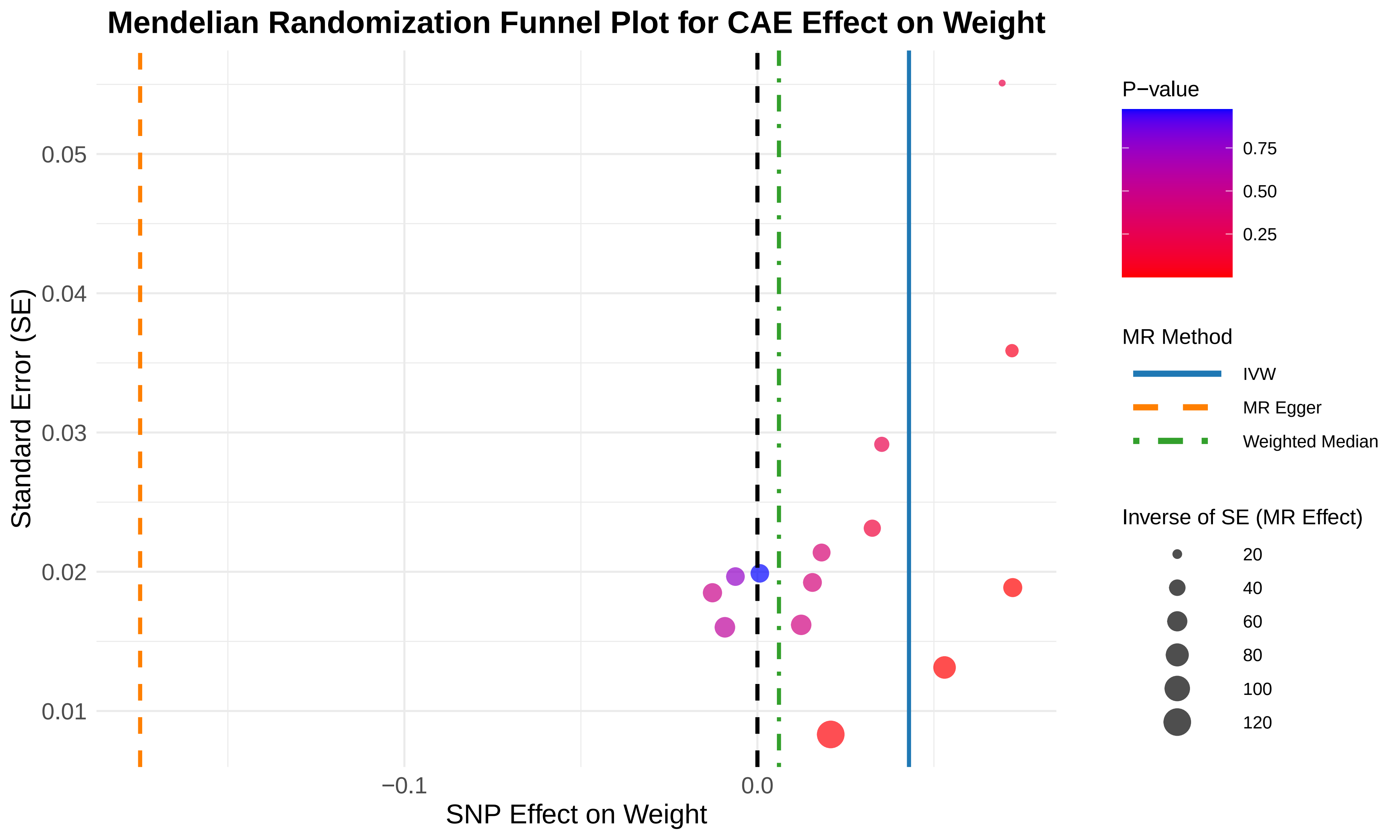

Mendelian Randomization Funnel Plot for CAE Effect on Weight
Standard Error (SE)
0.05
0.04
0.03
0.02
0.01
−0.1
0.0
SNP Effect on Weight
P−value
0.75
0.50
0.25
MR Method
IVW
MR Egger
Weighted Median
Inverse of SE (MR Effect)
20
40
60
80
100
120

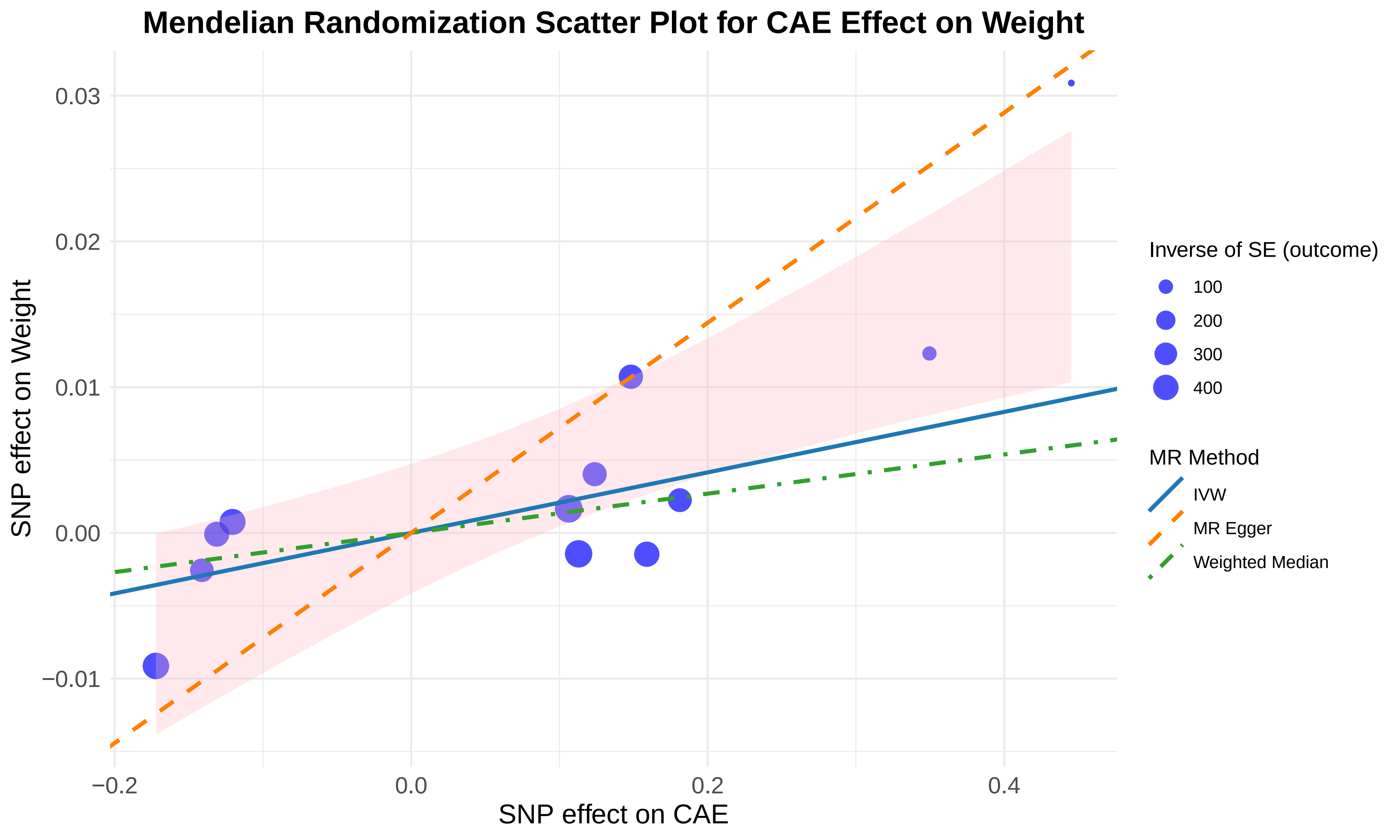

Mendelian Randomization Scatter Plot for CAE Effect on Weight
0.03
0.02
0.01
0.00
−0.01
−0.2
0.0
0.2
0.4
SNP effect on CAE
SNP effect on Weight
Inverse of SE (outcome)
100
200
300
400
MR Method
IVW
MR Egger
Weighted Median

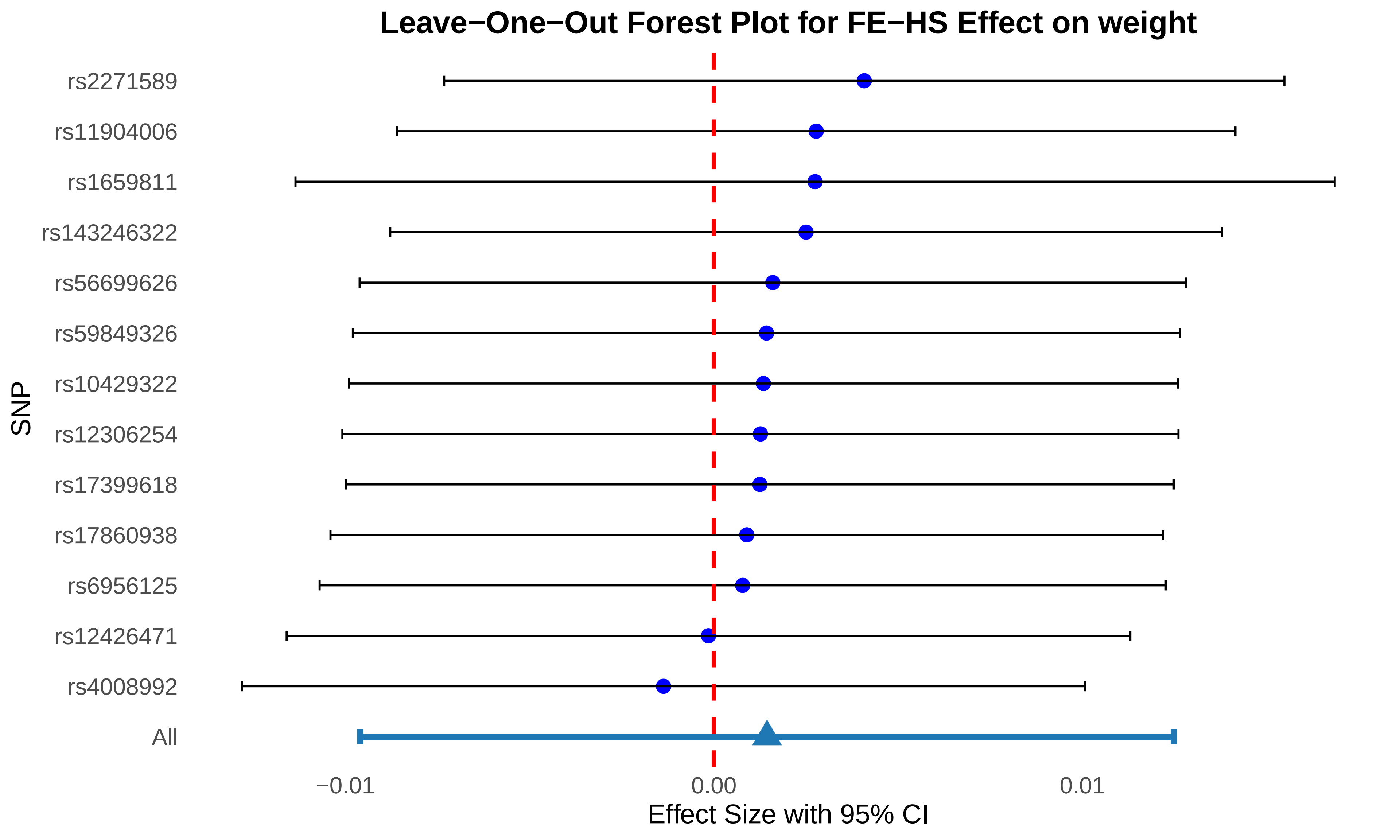

Leave−One−Out Forest Plot for FE−HS Effect on weight
SNP
rs2271589
rs11904006
rs1659811
rs143246322
rs56699626
rs59849326
rs10429322
rs12306254
rs17399618
rs17860938
rs6956125
rs12426471
rs4008992
All
−0.01
0.00
0.01
Effect Size with 95% CI

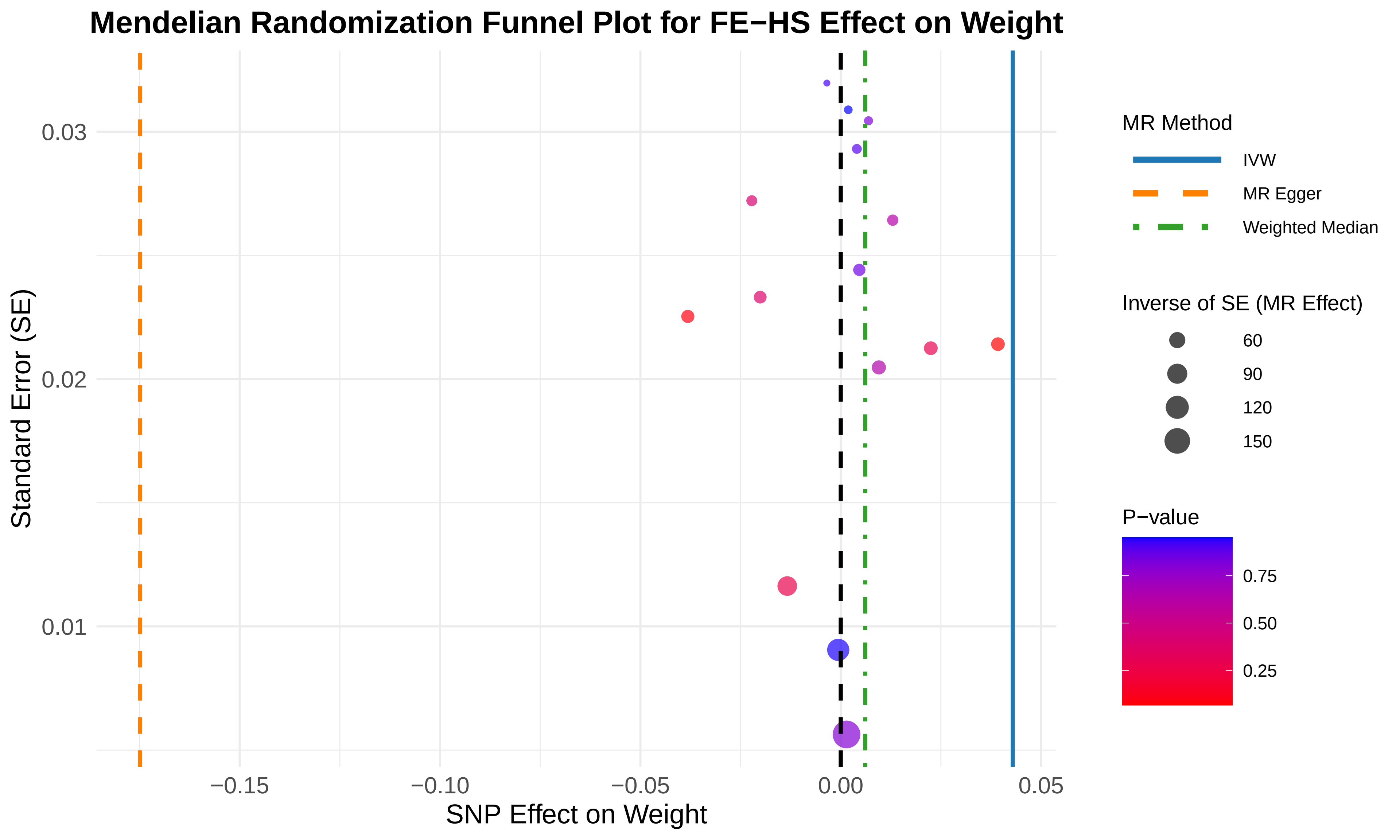
Mendelian Randomization Funnel Plot for FE−HS Effect on Weight
Standard Error (SE)
0.03
0.02
0.01
−0.15
−0.10
−0.05
0.00
0.05
SNP Effect on Weight
MR Method
IVW
MR Egger
Weighted Median
Inverse of SE (MR Effect)
60
90
120
150
P−value
0.75
0.50
0.25

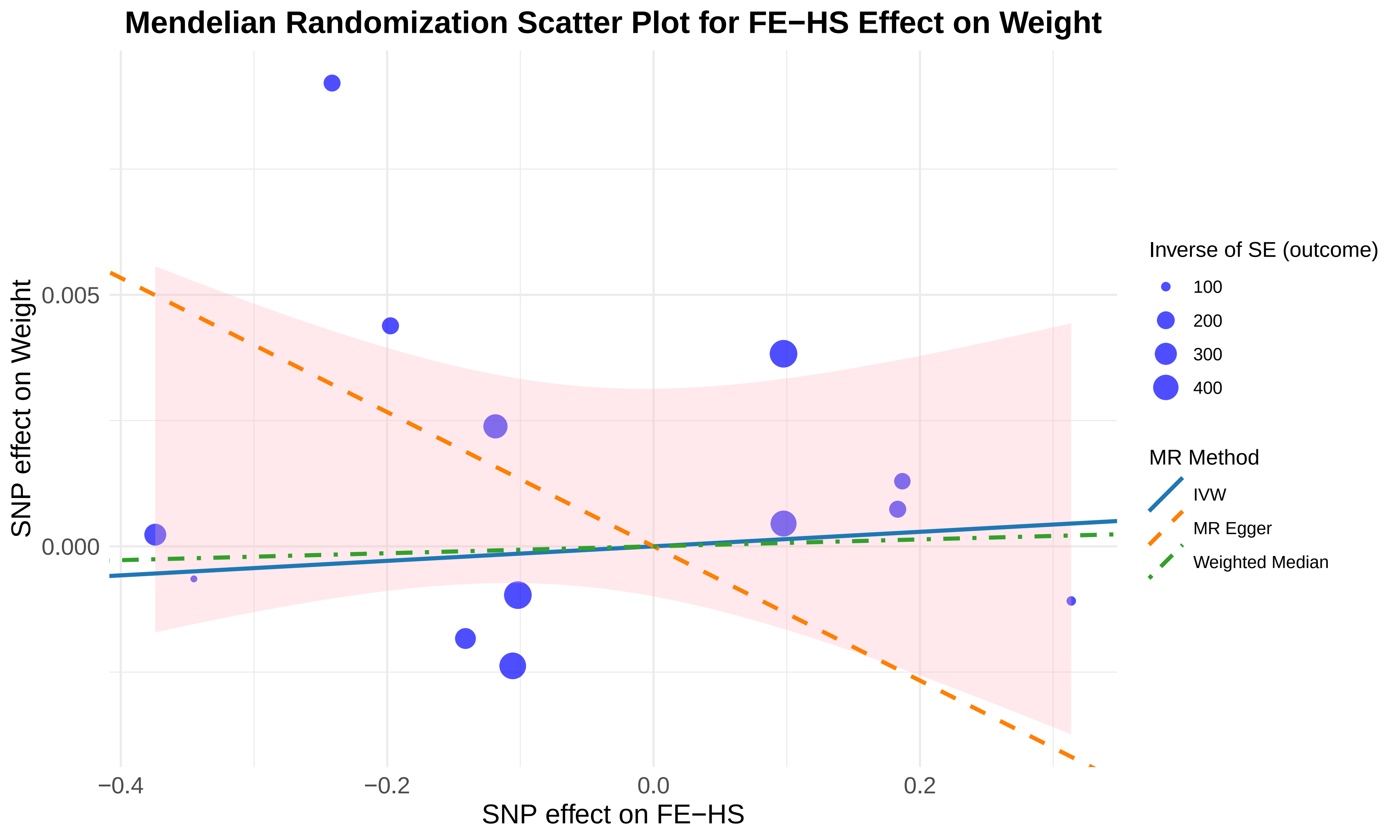

Mendelian Randomization Scatter Plot for FE−HS Effect on Weight
SNP effect on Weight
0.005
0.000
−0.4
−0.2
0.0
0.2
SNP effect on FE−HS
Inverse of SE (outcome)
100
200
300
400
MR Method
IVW
MR Egger
Weighted Median

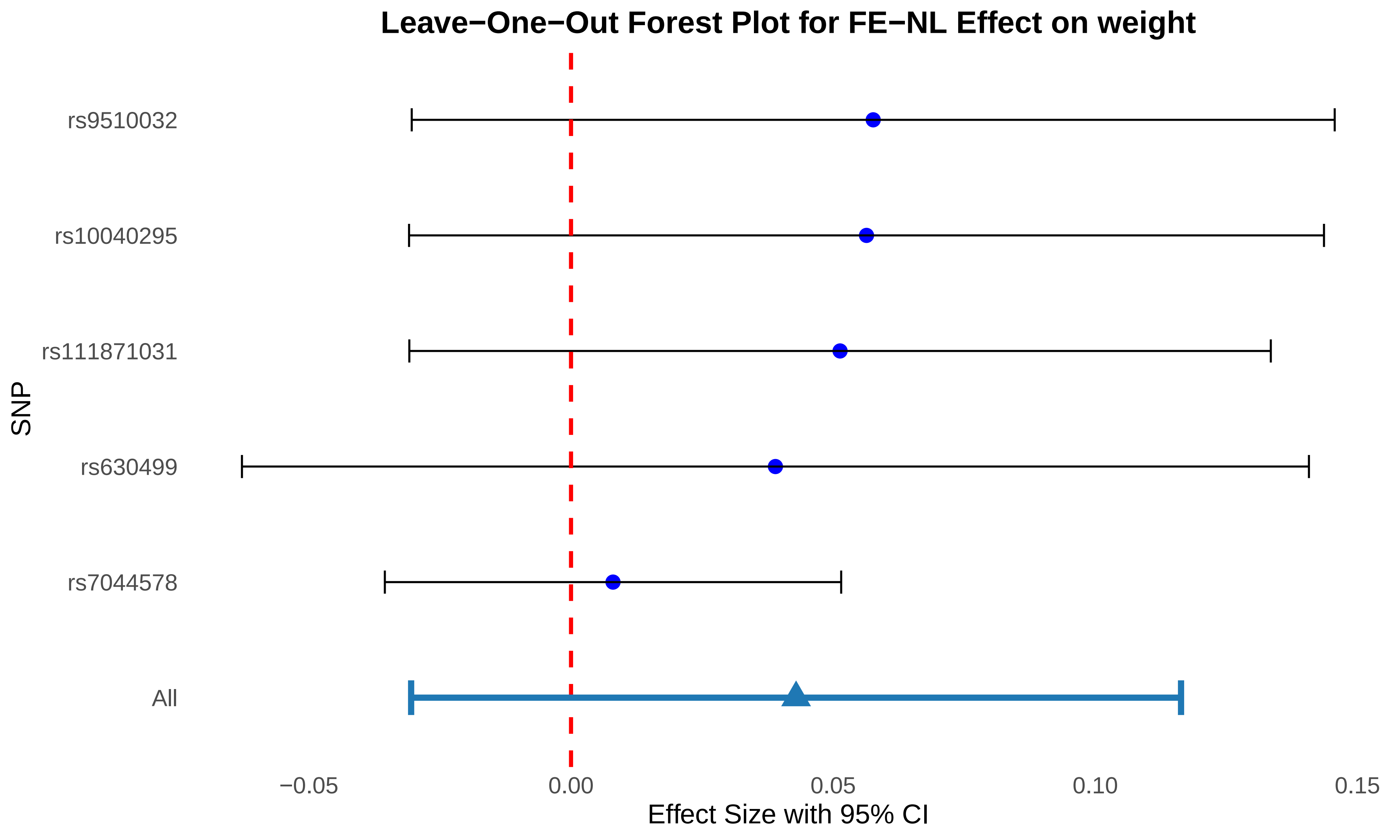
Leave−One−Out Forest Plot for FE−NL Effect on weight
rs9510032
rs10040295
rs111871031
rs630499
rs7044578
All
SNP
−0.05
0.00
0.05
0.10
0.15
Effect Size with 95% CI

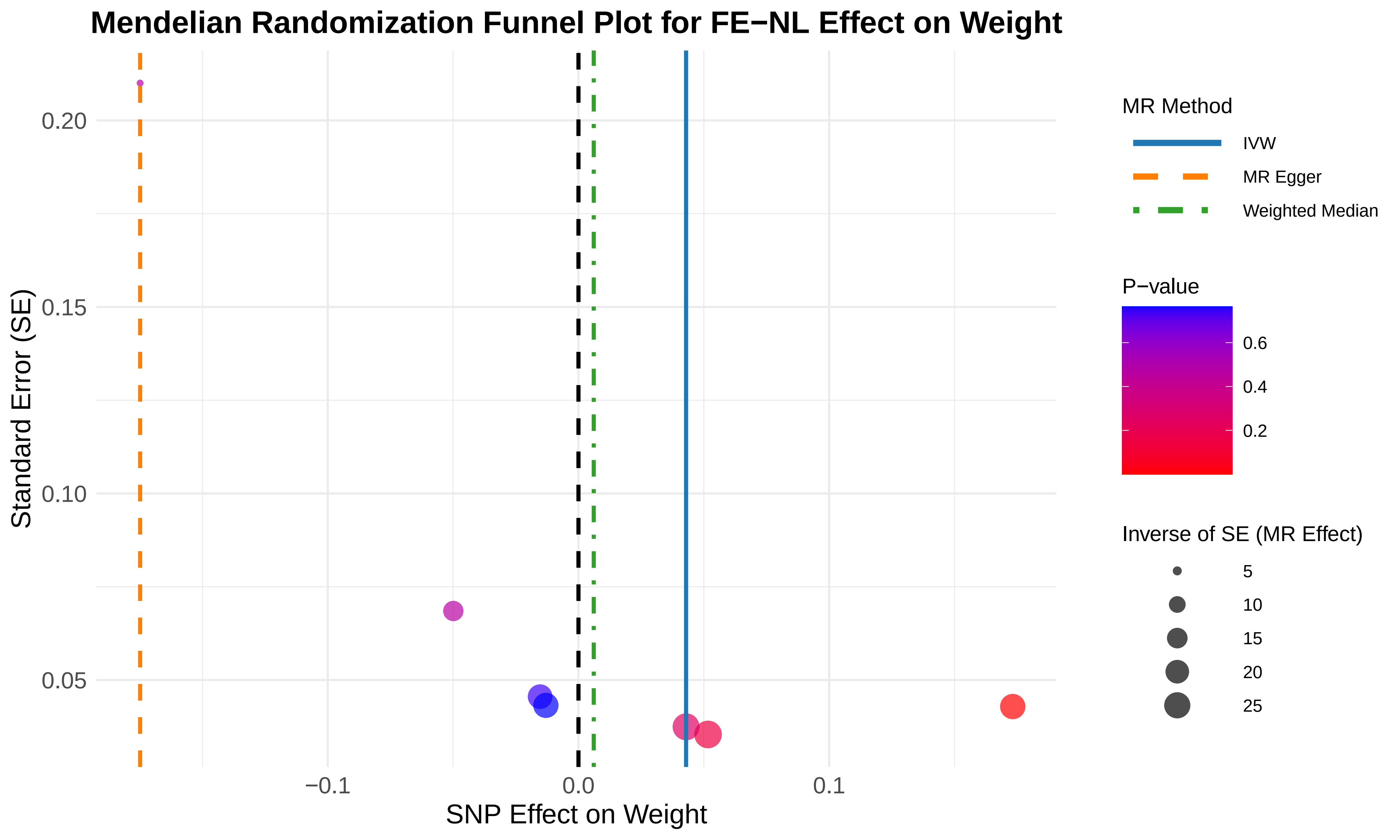

Mendelian Randomization Funnel Plot for FE−NL Effect on Weight
Standard Error (SE)
0.20
0.15
0.10
0.05
−0.1
0.0
0.1
SNP Effect on Weight
MR Method
IVW
MR Egger
Weighted Median
P−value
0.6
0.4
0.2
Inverse of SE (MR Effect)
5
10
15
20
25

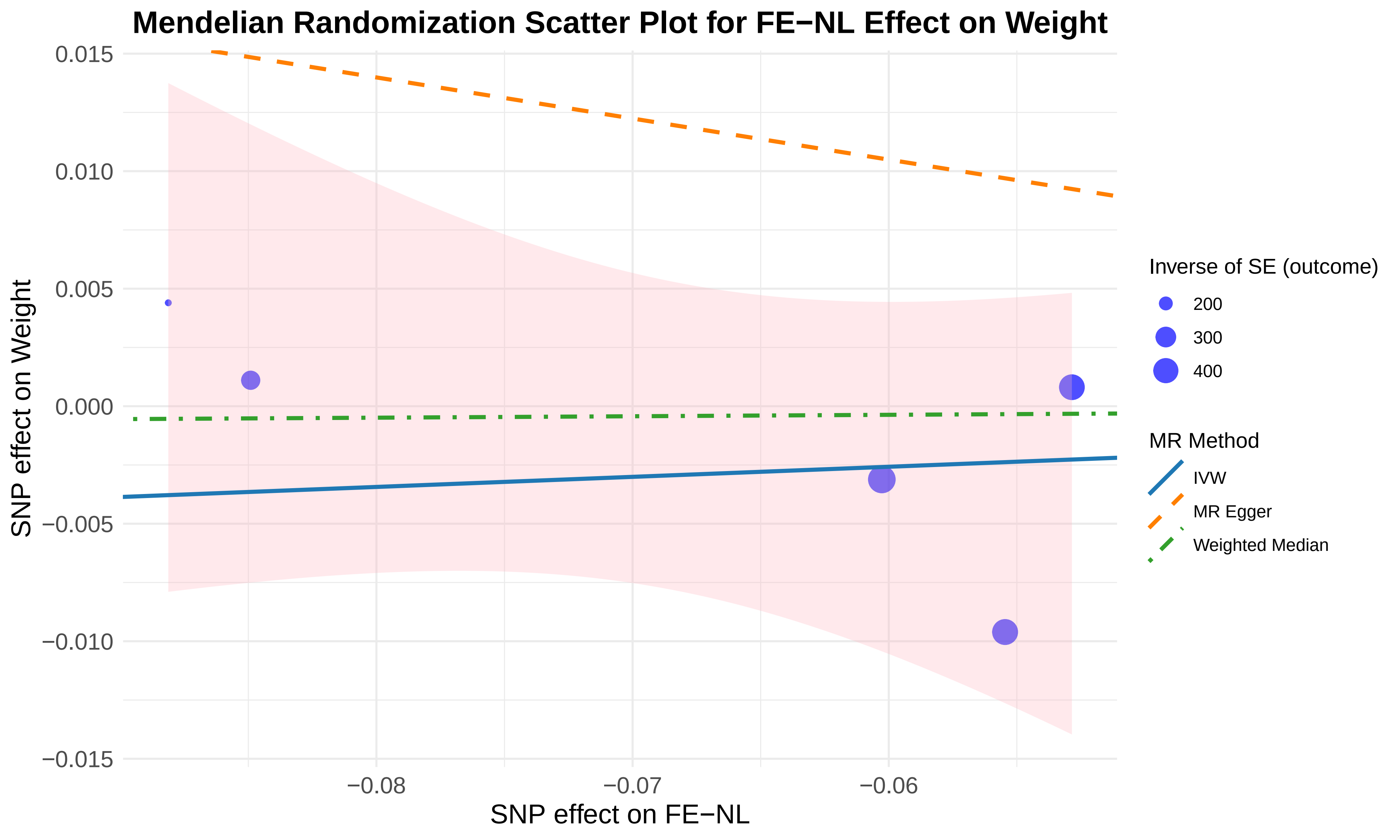

Mendelian Randomization Scatter Plot for FE−NL Effect on Weight
0.015
0.010
0.005
0.000
−0.005
−0.010
−0.015
SNP effect on Weight
−0.08
−0.07
−0.06
SNP effect on FE−NL
Inverse of SE (outcome)
200
300
400
MR Method
IVW
MR Egger
Weighted Median

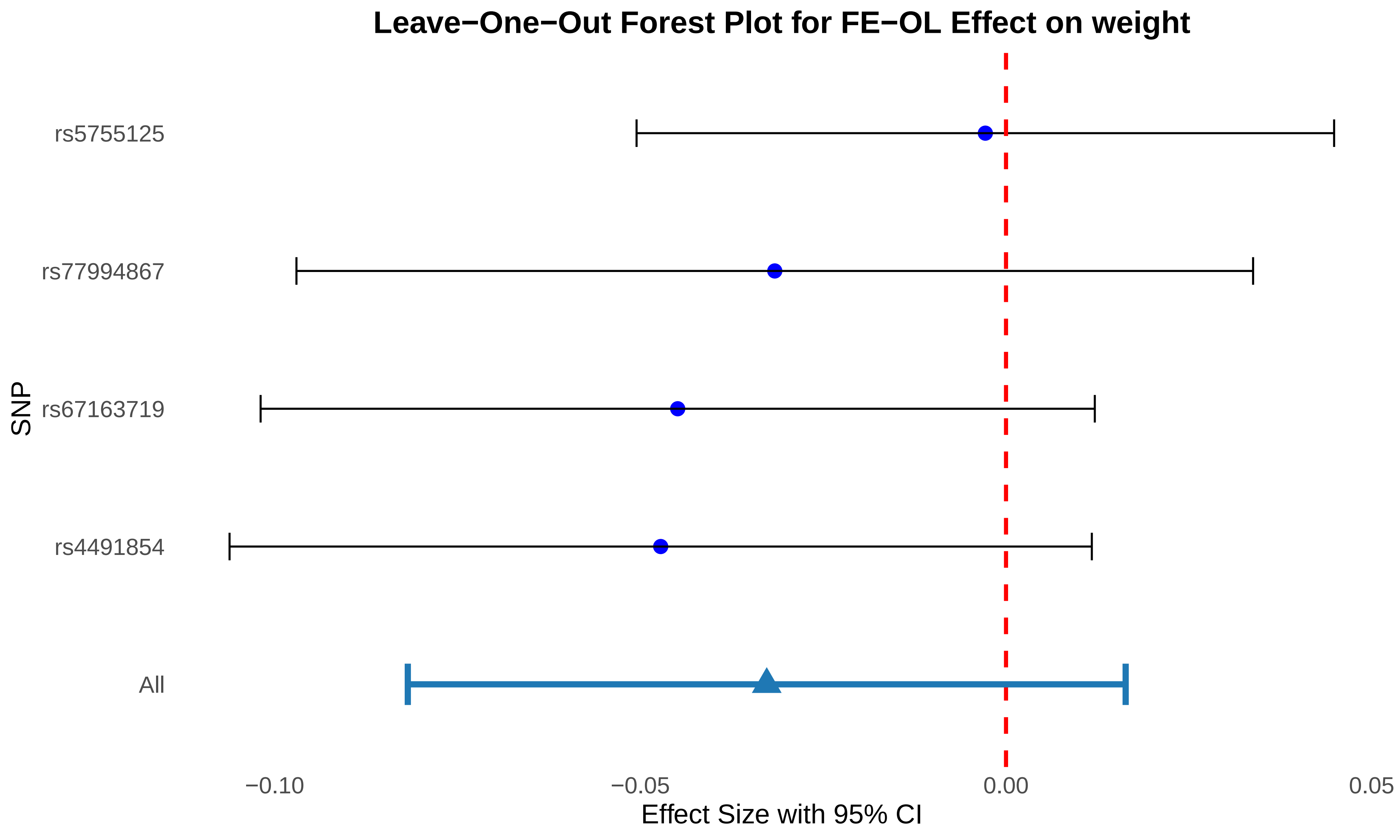
Leave−One−Out Forest Plot for FE−OL Effect on weight
SNP
rs5755125
rs77994867
rs67163719
rs4491854
All
−0.10
−0.05
0.00
0.05
Effect Size with 95% CI

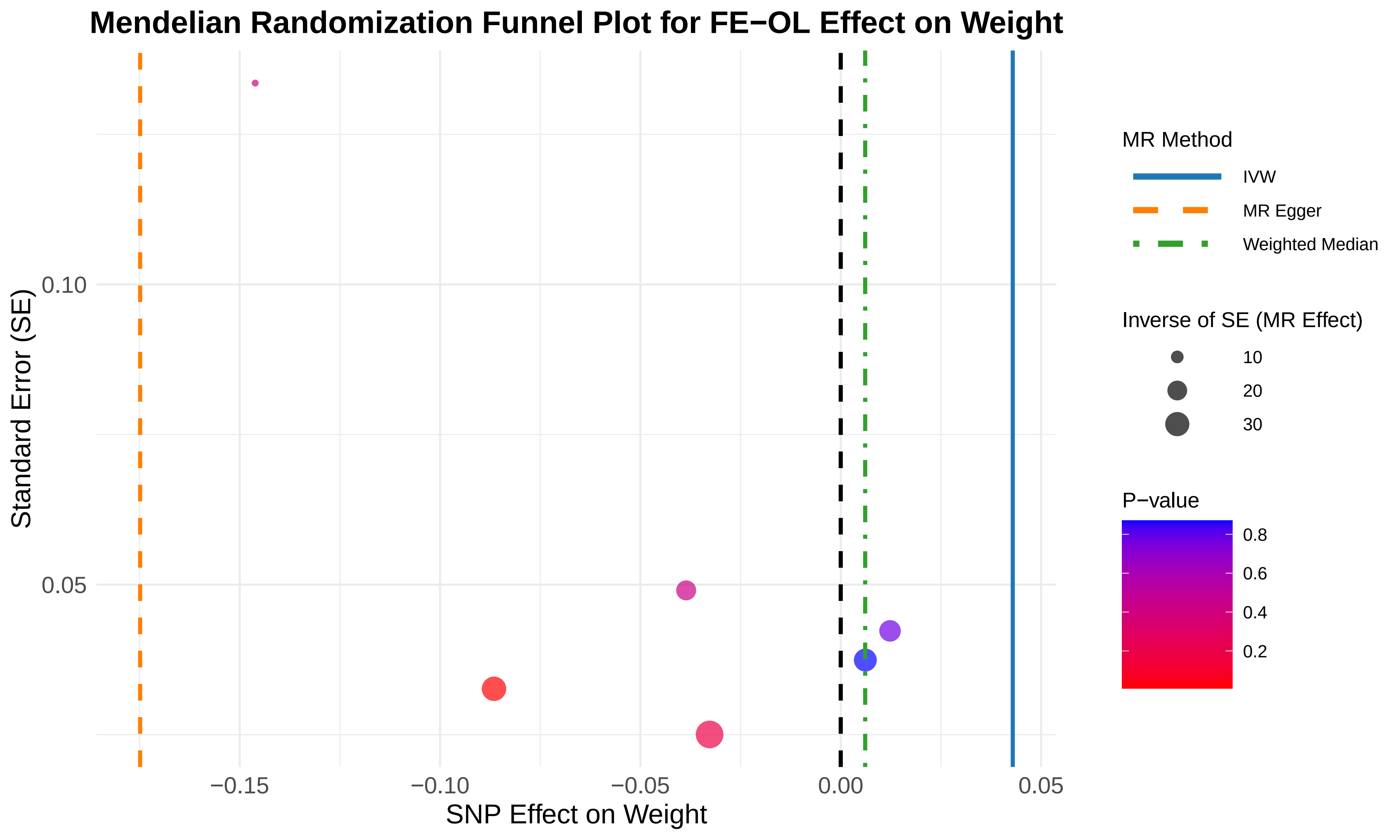

Mendelian Randomization Funnel Plot for FE−OL Effect on Weight
Standard Error (SE)
0.10
0.05
−0.15
−0.10
−0.05
0.00
0.05
SNP Effect on Weight
MR Method
IVW
MR Egger
Weighted Median
Inverse of SE (MR Effect)
10
20
30
P−value
0.8
0.6
0.4
0.2

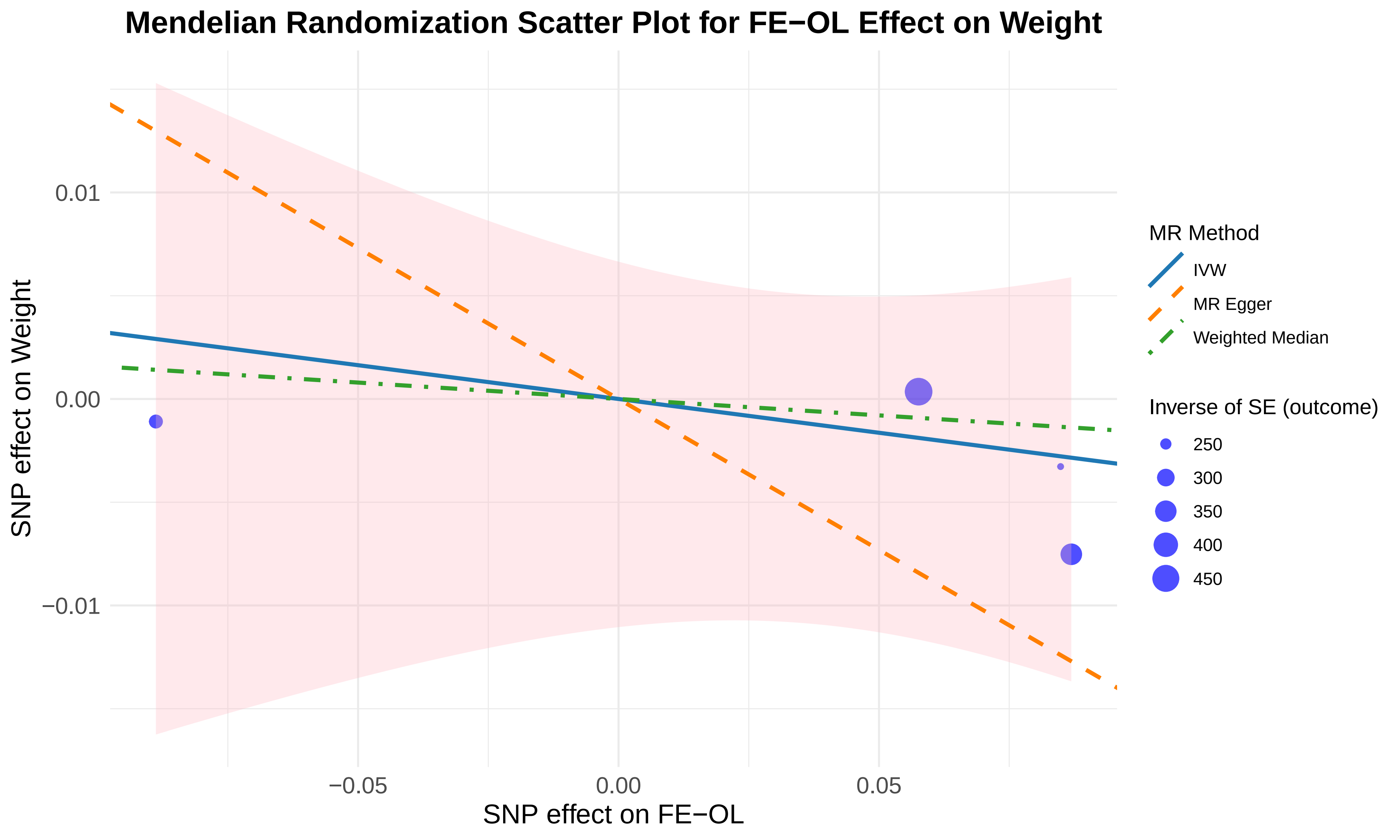

Mendelian Randomization Scatter Plot for FE−OL Effect on Weight
SNP effect on Weight
0.01
0.00
−0.01
−0.05
0.00
0.05
SNP effect on FE−OL
MR Method
IVW
MR Egger
Weighted Median
Inverse of SE (outcome)
250
300
350
400
450

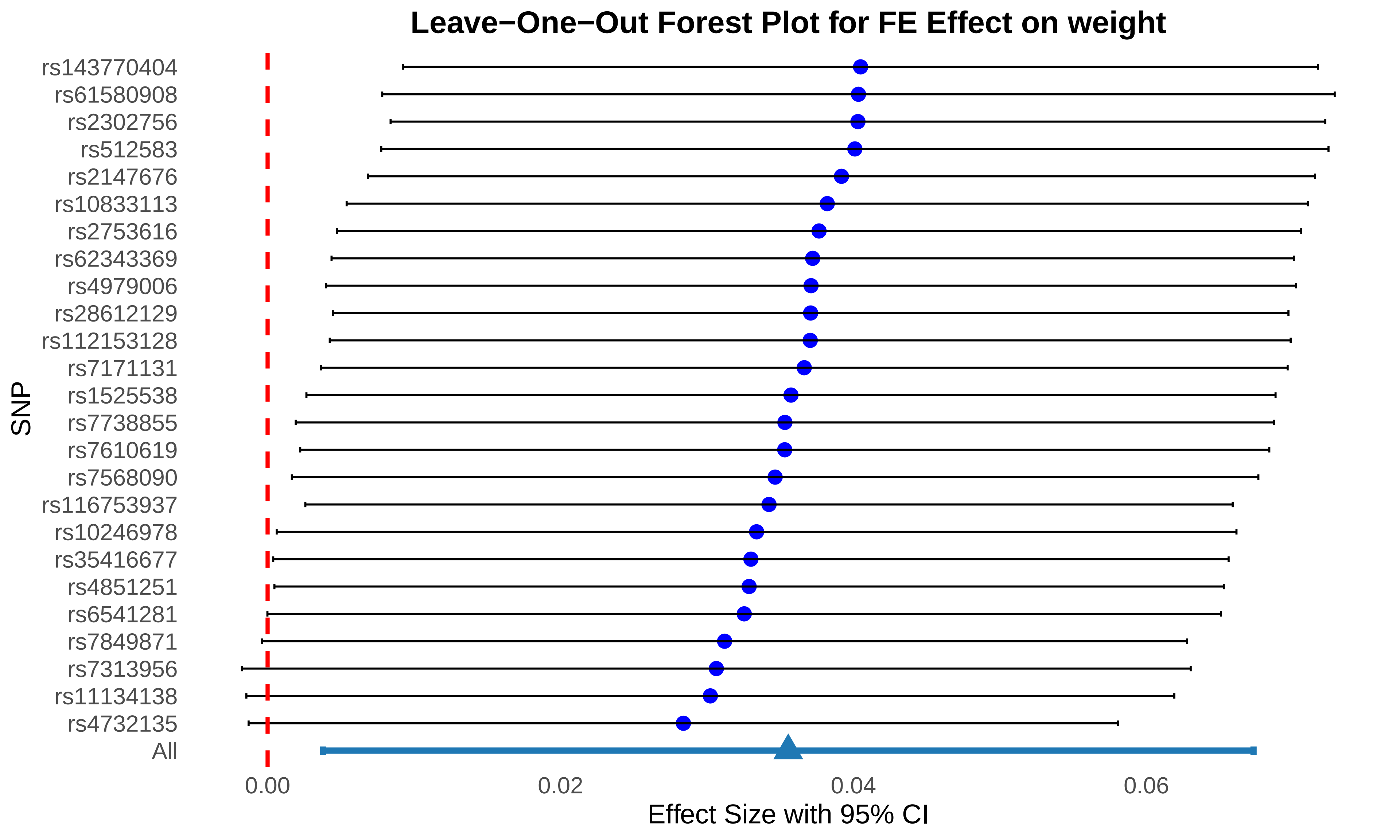

Leave−One−Out Forest Plot for FE Effect on weight
rs143770404
rs61580908
rs2302756
rs512583
rs2147676
rs10833113
rs2753616
rs62343369
rs4979006
rs28612129
rs112153128
rs7171131
rs1525538
rs7738855
rs7610619
rs7568090
rs116753937
rs10246978
rs35416677
rs4851251
rs6541281
rs7849871
rs7313956
rs11134138
rs4732135
All
SNP
0.00
0.02
0.04
0.06
Effect Size with 95% CI

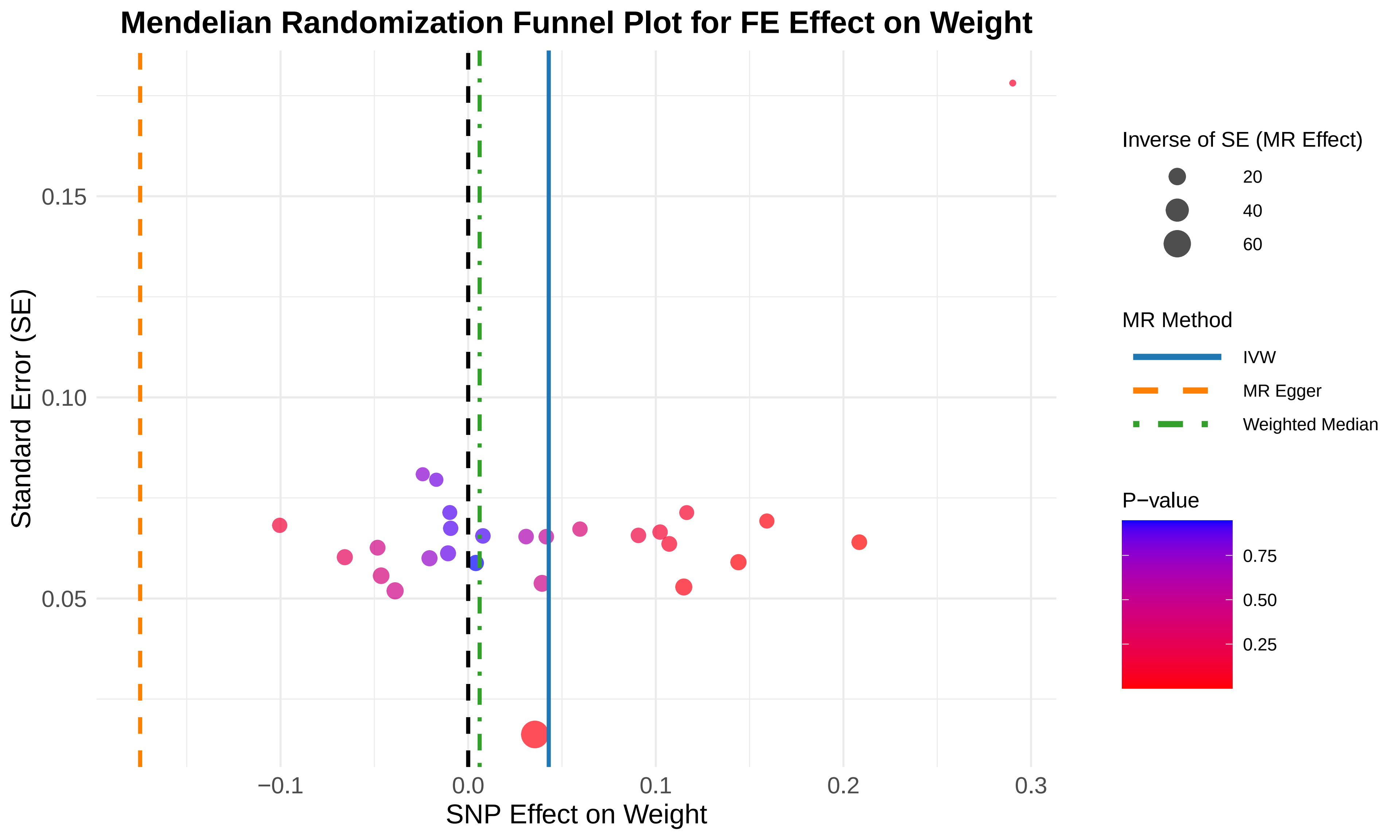

Mendelian Randomization Funnel Plot for FE Effect on Weight
Standard Error (SE)
0.15
0.10
0.05
−0.1
0.0
0.1
0.2
0.3
SNP Effect on Weight
Inverse of SE (MR Effect)
20
40
60
MR Method
IVW
MR Egger
Weighted Median
P−value
0.75
0.50
0.25

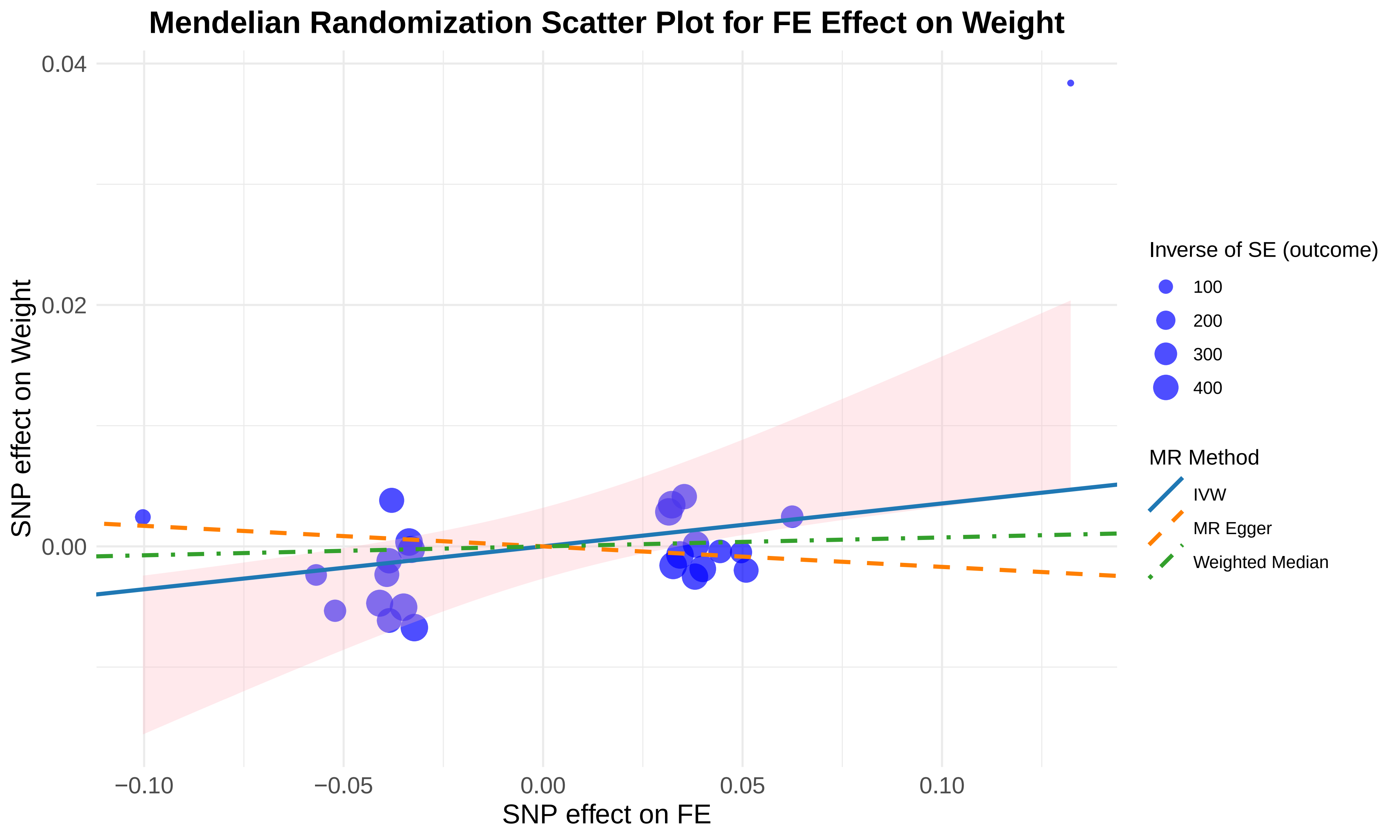

Mendelian Randomization Scatter Plot for FE Effect on Weight
0.04
0.02
0.00
SNP effect on Weight
−0.10
−0.05
0.00
0.05
0.10
SNP effect on FE
Inverse of SE (outcome)
100
200
300
400
MR Method
IVW
MR Egger
Weighted Median

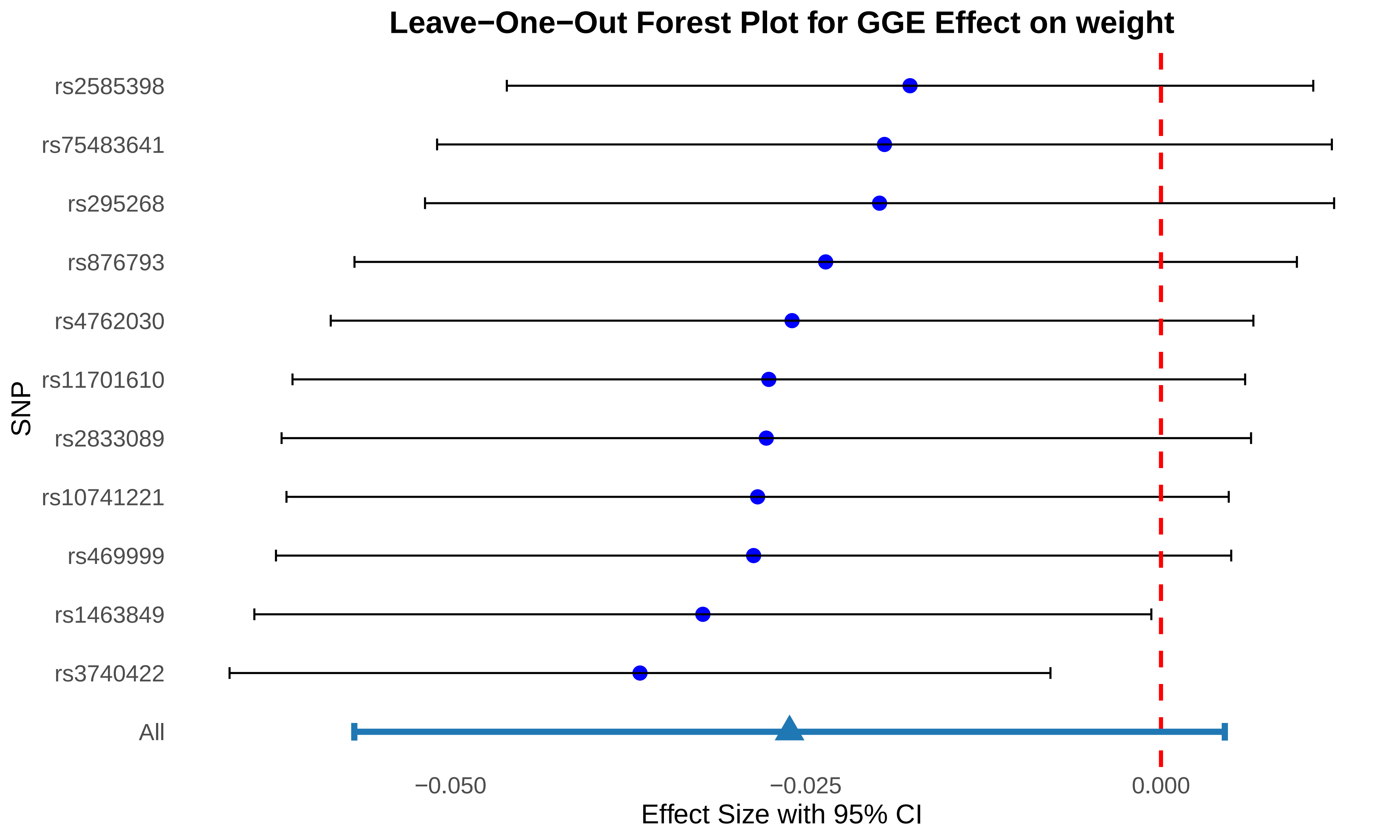

Leave−One−Out Forest Plot for GGE Effect on weight
SNP
rs2585398
rs75483641
rs295268
rs876793
rs4762030
rs11701610
rs2833089
rs10741221
rs469999
rs1463849
rs3740422
All
−0.050
−0.025
0.000
Effect Size with 95% CI

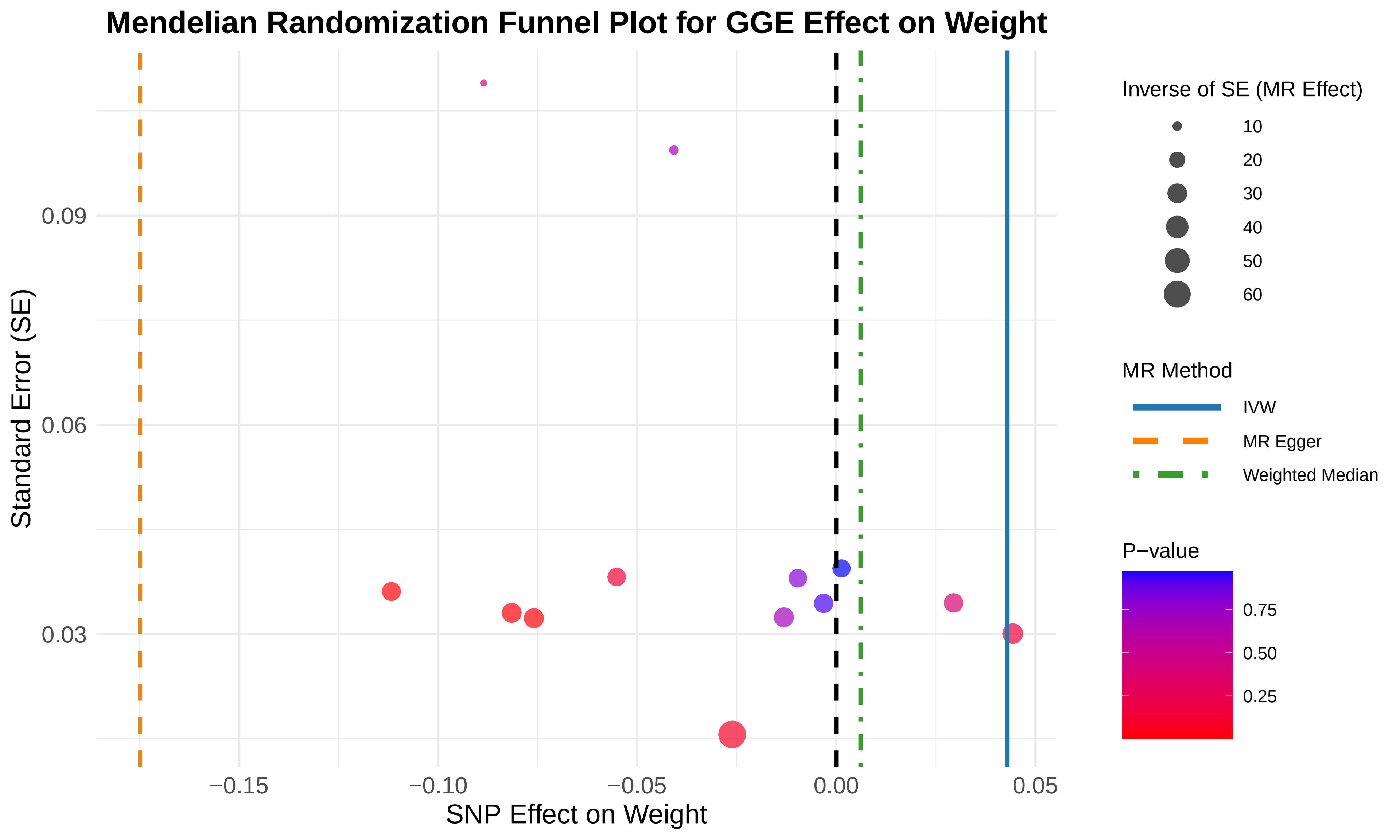
Mendelian Randomization Funnel Plot for GGE Effect on Weight
Standard Error (SE)
0.09
0.06
0.03
−0.15
−0.10
−0.05
0.00
0.05
SNP Effect on Weight
Inverse of SE (MR Effect)
10
20
30
40
50
60
MR Method
IVW
MR Egger
Weighted Median
P−value
0.75
0.50
0.25

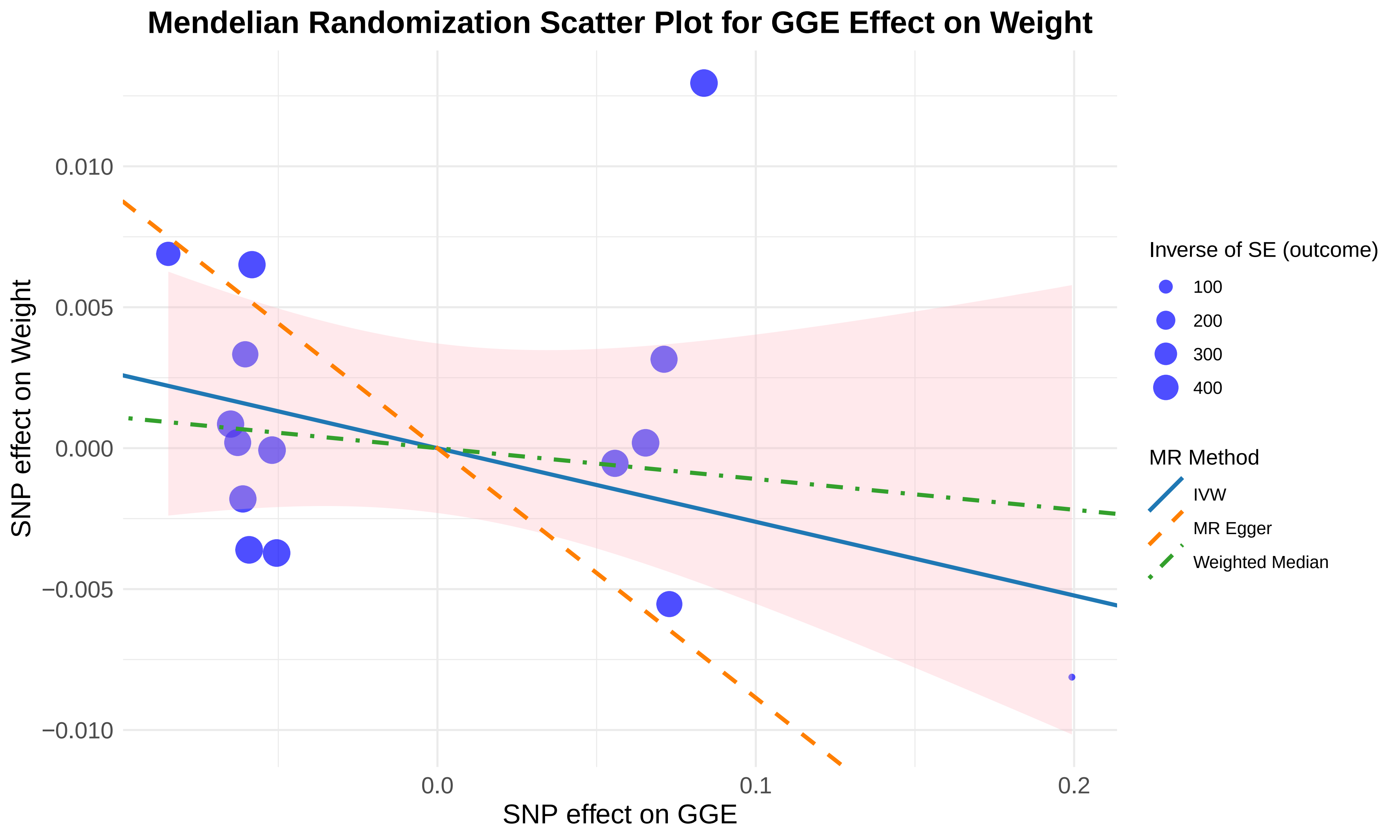
Mendelian Randomization Scatter Plot for GGE Effect on Weight
SNP effect on Weight
SNP effect on GGE
0.010
0.005
0.000
−0.005
−0.010
0.0
0.1
0.2
Inverse of SE (outcome)
100
200
300
400
MR Method
IVW
MR Egger
Weighted Median

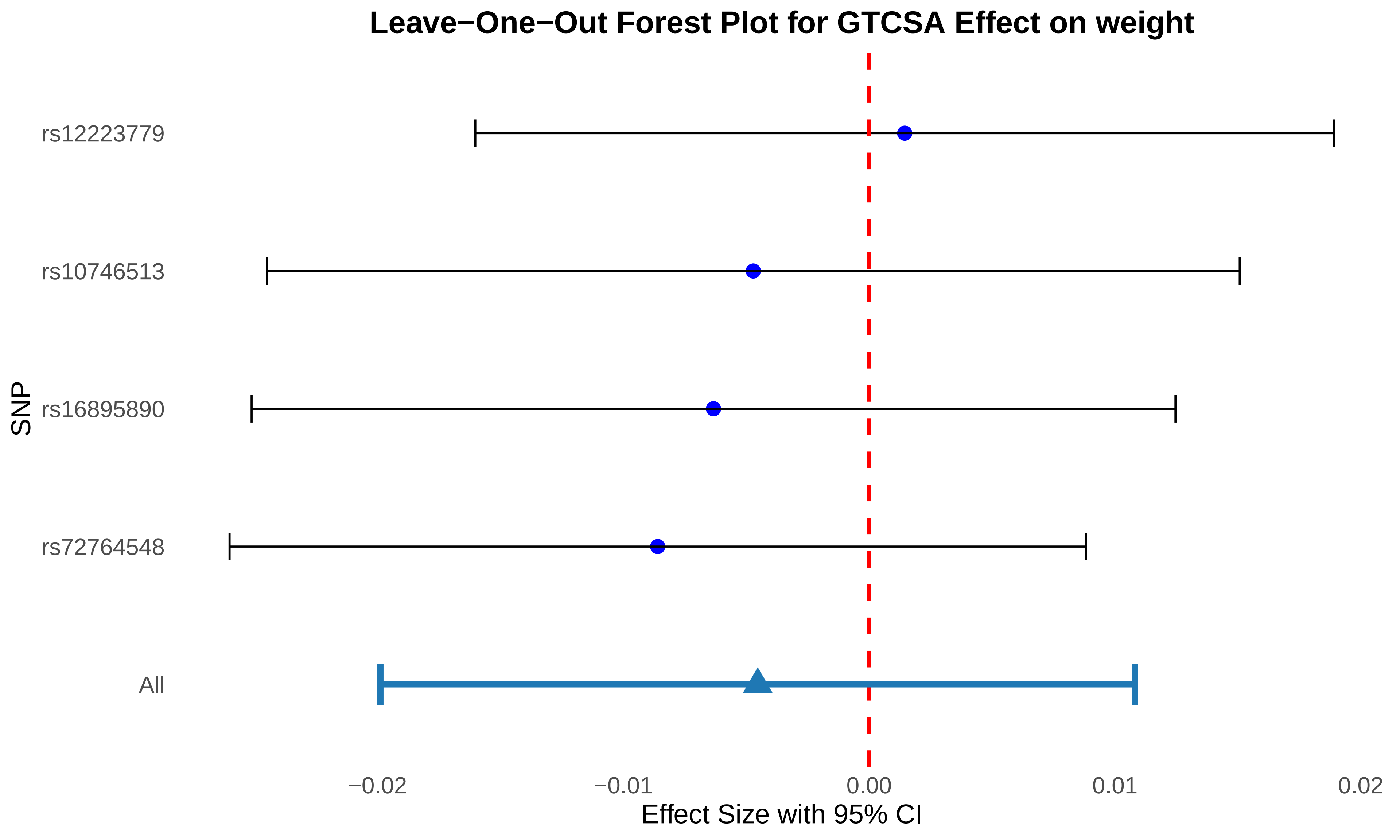
Leave−One−Out Forest Plot for GTCSA Effect on weight
rs12223779
rs10746513
rs16895890
rs72764548
All
SNP
−0.02
−0.01
0.00
0.01
0.02
Effect Size with 95% CI

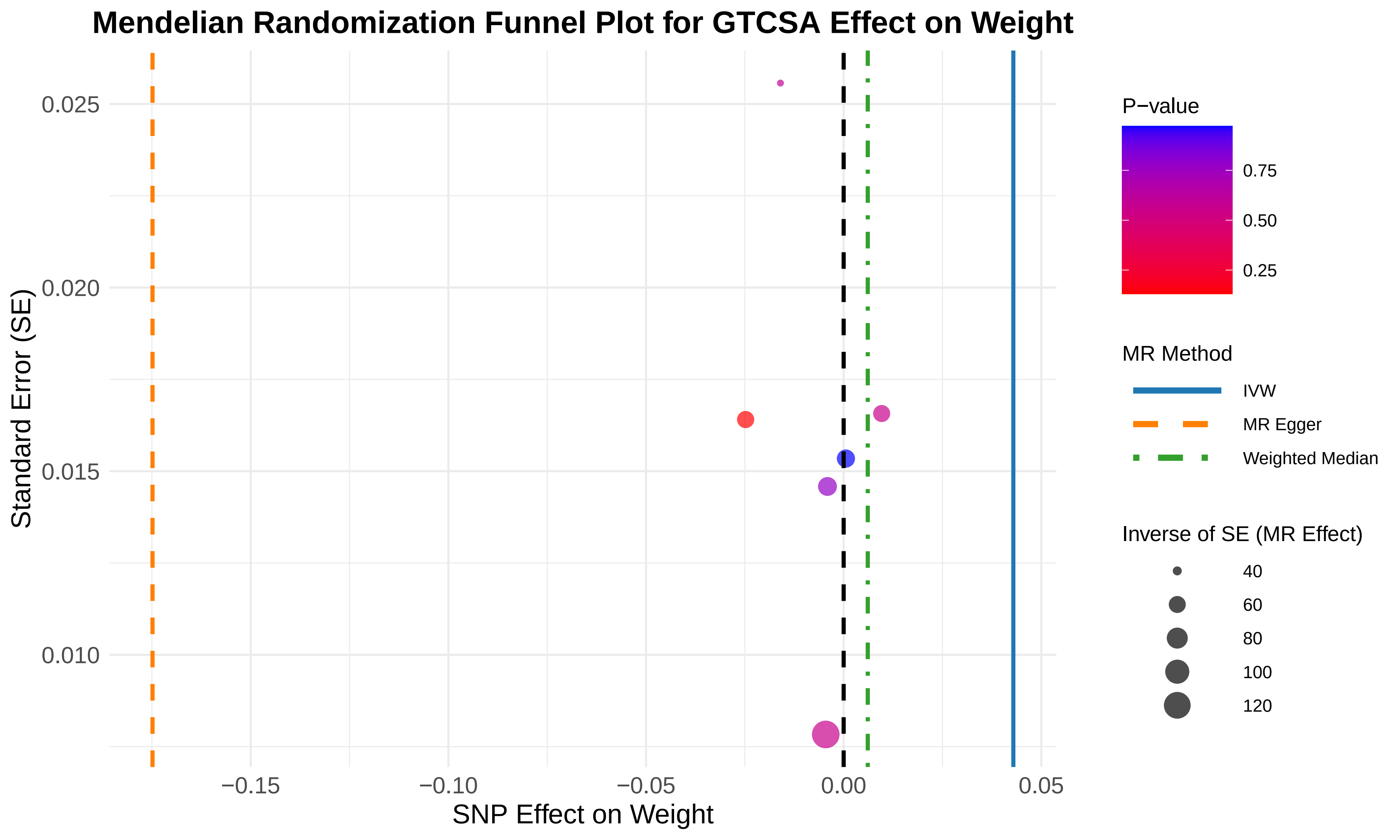
Mendelian Randomization Funnel Plot for GTCSA Effect on Weight
Standard Error (SE)
0.025
0.020
0.015
0.010
−0.15
−0.10
−0.05
0.00
0.05
SNP Effect on Weight
P−value
0.75
0.50
0.25
MR Method
IVW
MR Egger
Weighted Median
Inverse of SE (MR Effect)
40
60
80
100
120

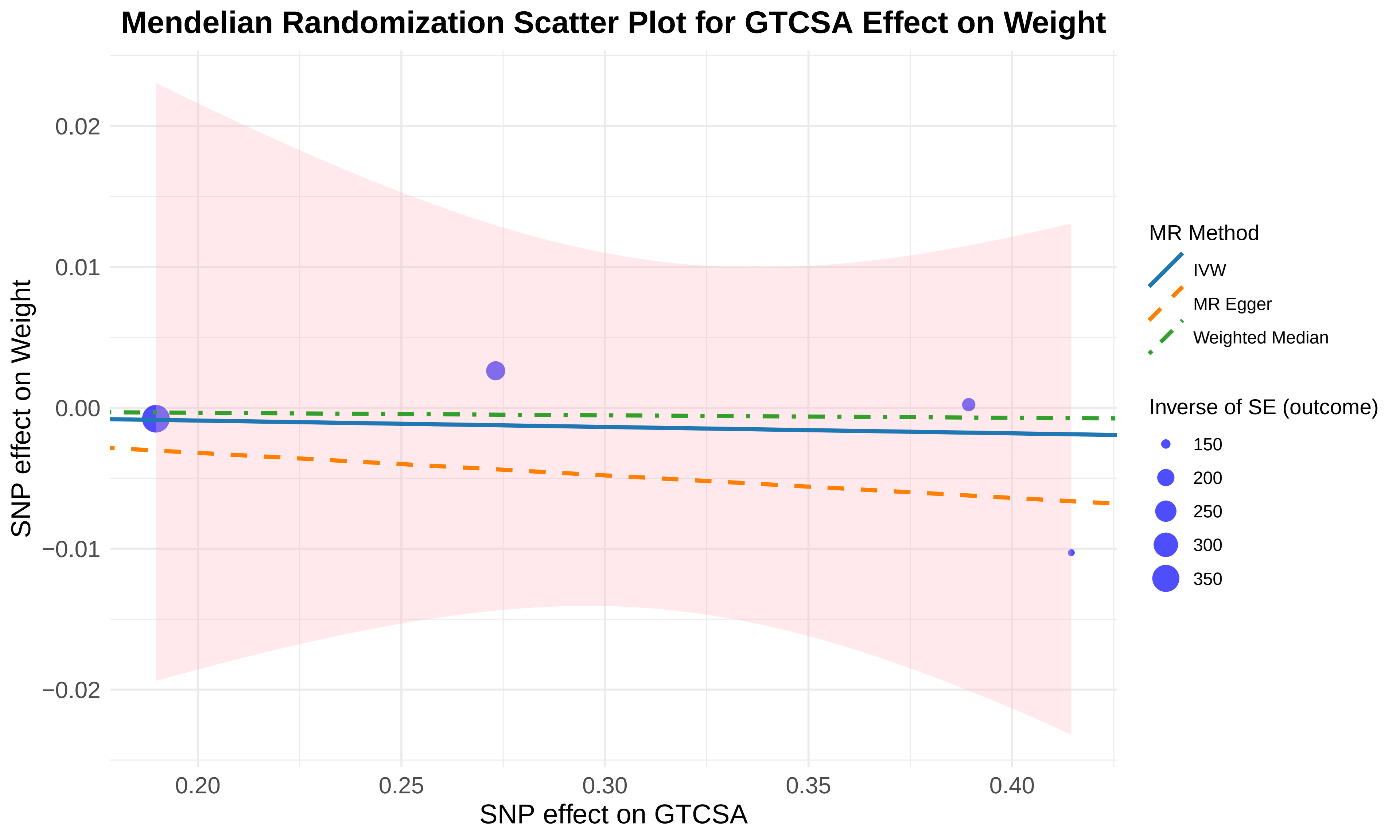
Mendelian Randomization Scatter Plot for GTCSA Effect on Weight
0.02
0.01
0.00
−0.01
−0.02
0.20
0.25
0.30
0.35
0.40
SNP effect on GTCSA
SNP effect on Weight
MR Method
IVW
MR Egger
Weighted Median
Inverse of SE (outcome)
150
200
250
300
350

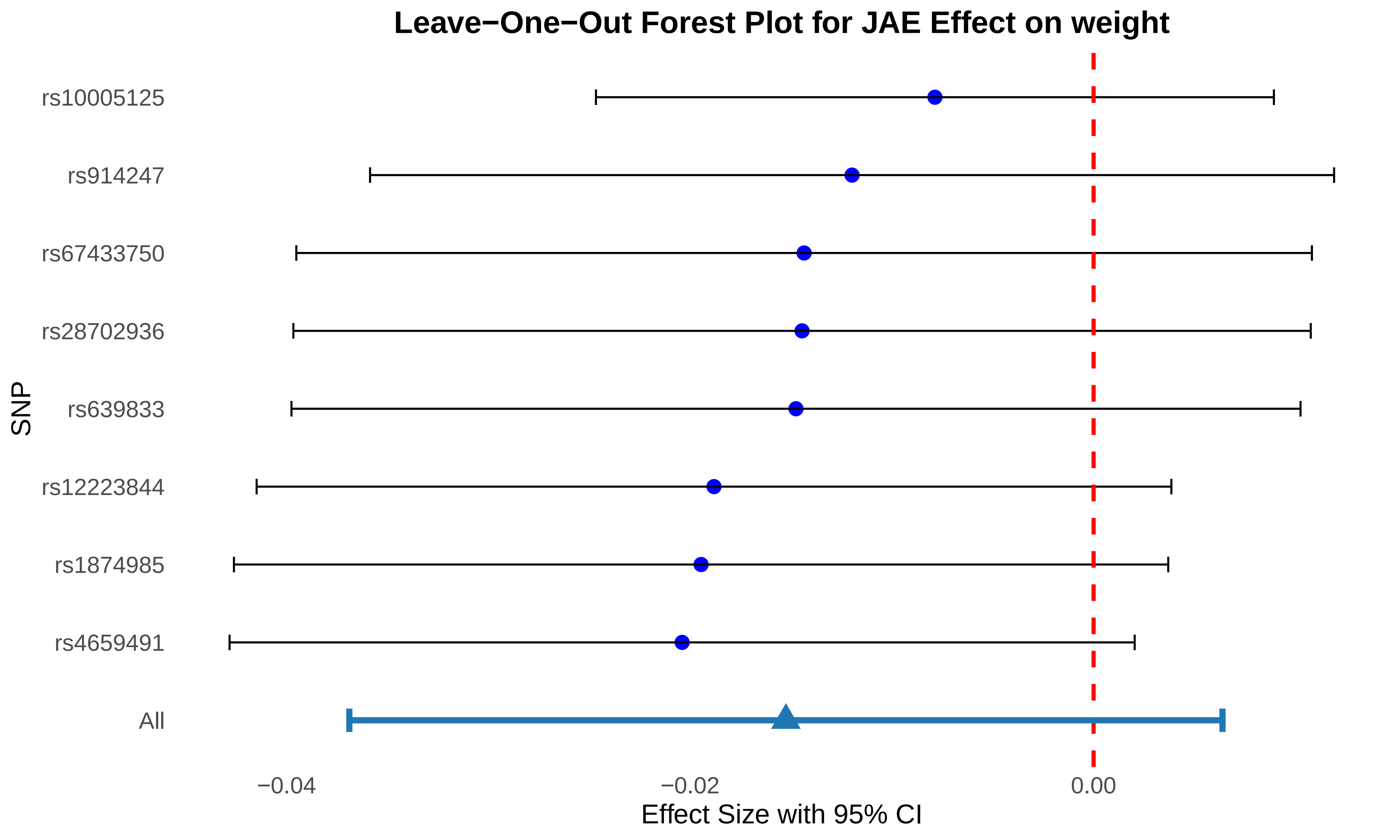

Leave−One−Out Forest Plot for JAE Effect on weight
rs10005125
rs914247
rs67433750
rs28702936
rs639833
rs12223844
rs1874985
rs4659491
All
SNP
−0.04
−0.02
0.00
Effect Size with 95% CI

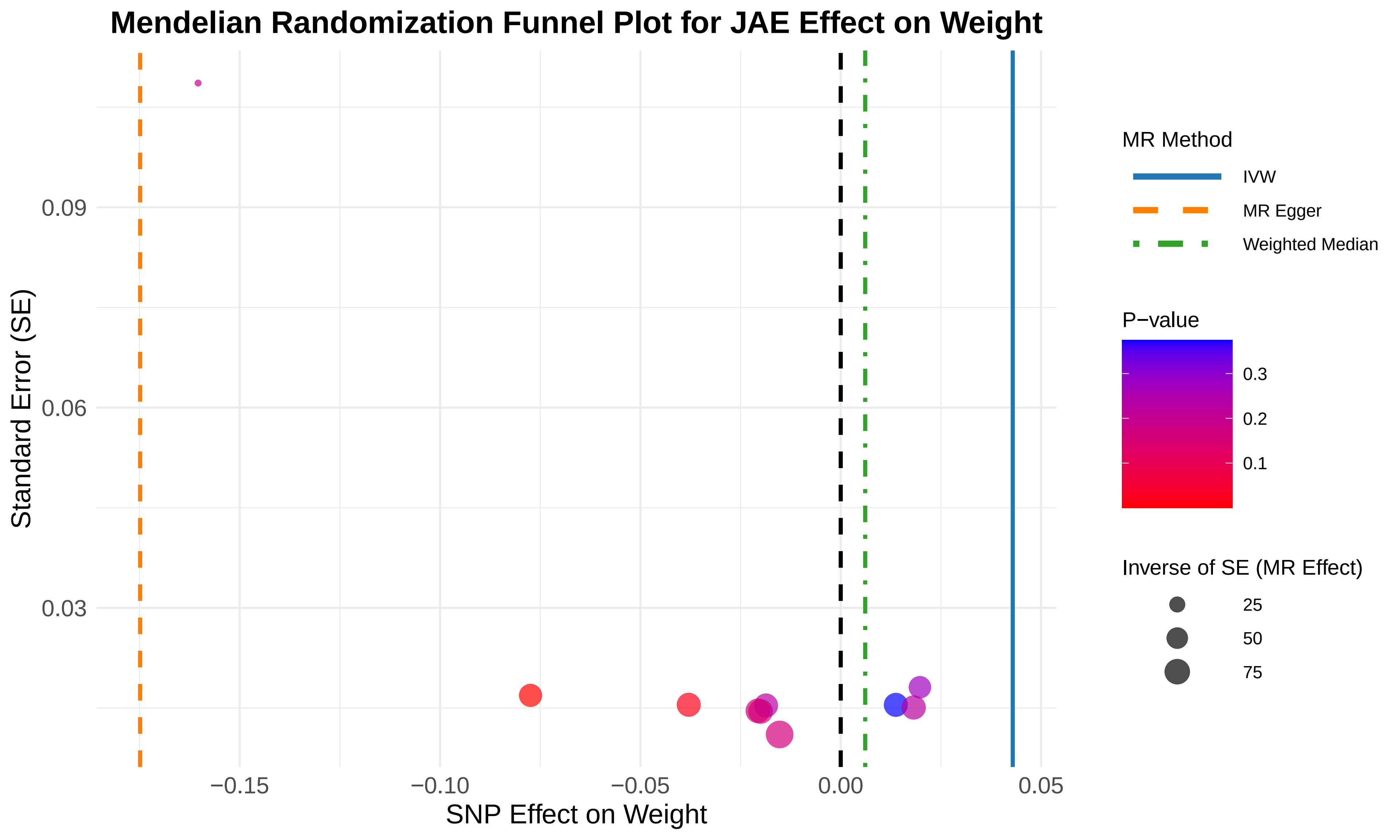

Mendelian Randomization Funnel Plot for JAE Effect on Weight
Standard Error (SE)
0.09
0.06
0.03
−0.15
−0.10
−0.05
0.00
0.05
SNP Effect on Weight
MR Method
IVW
MR Egger
Weighted Median
P−value
0.3
0.2
0.1
Inverse of SE (MR Effect)
25
50
75

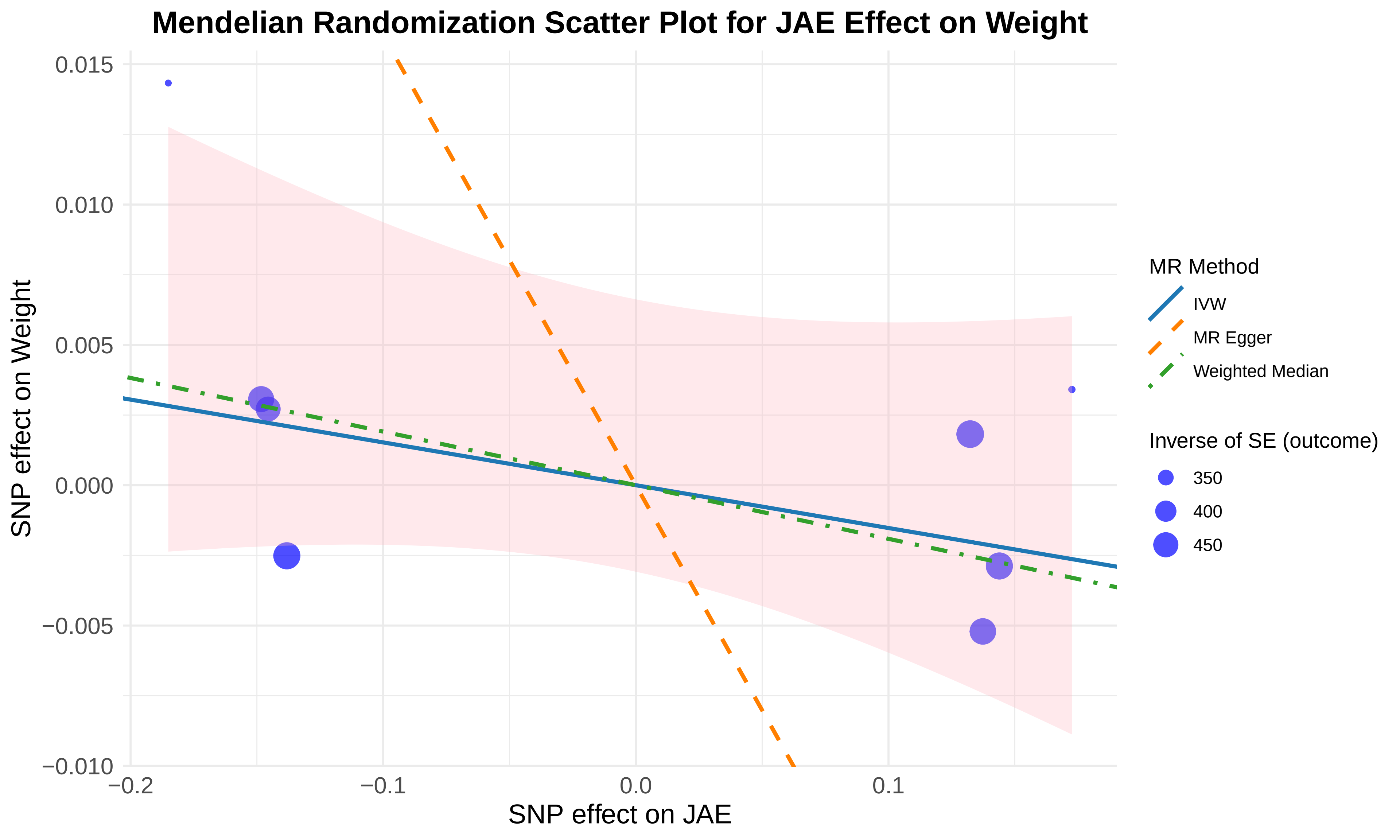

Mendelian Randomization Scatter Plot for JAE Effect on Weight
0.015
0.010
0.005
0.000
−0.005
−0.010
SNP effect on Weight
−0.2
−0.1
0.0
0.1
SNP effect on JAE
MR Method
IVW
MR Egger
Weighted Median
Inverse of SE (outcome)
350
400
450

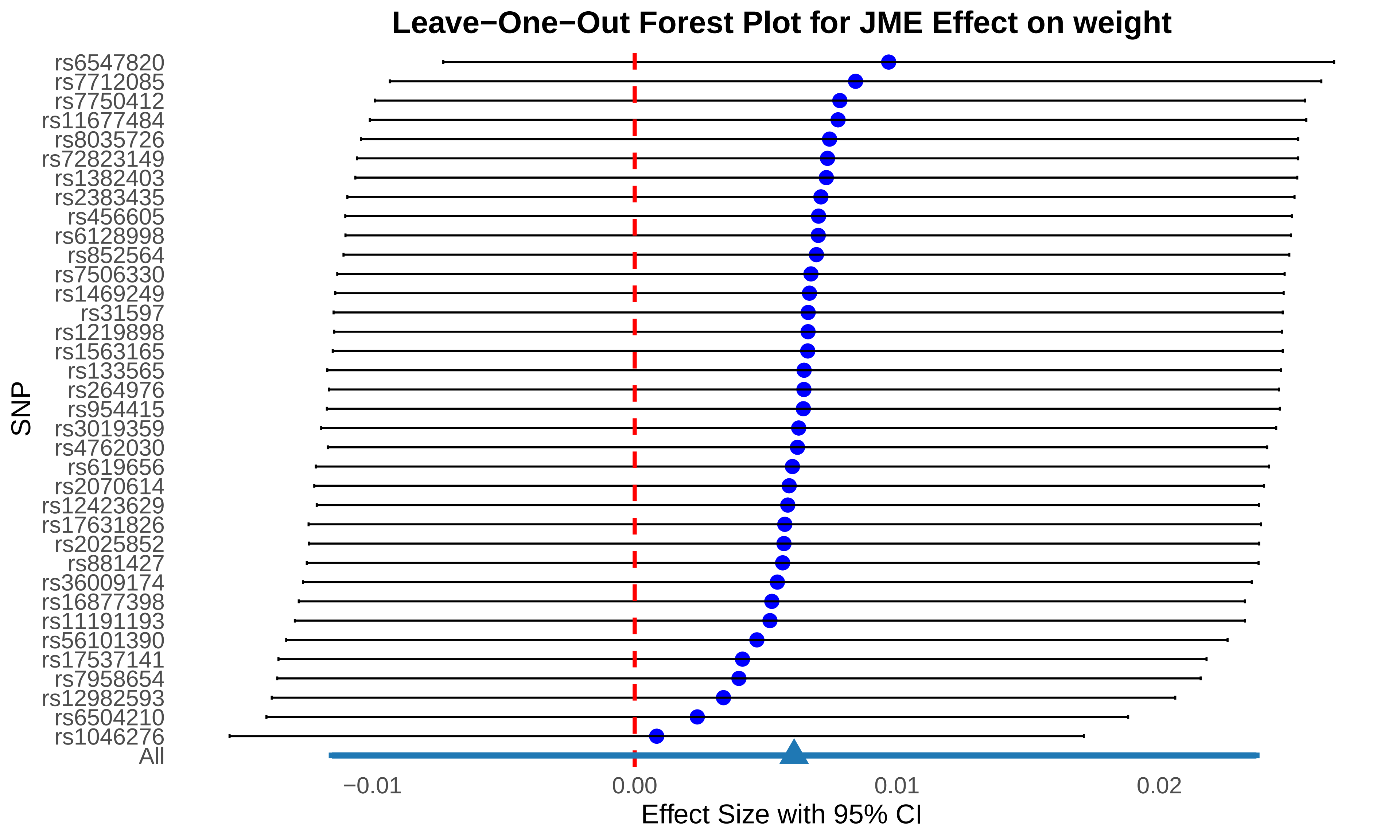
Leave−One−Out Forest Plot for JME Effect on weight
rs6547820
rs7712085
rs7750412
rs11677484
rs8035726
rs72823149
rs1382403
rs2383435
rs456605
rs6128998
rs852564
rs7506330
rs1469249
rs31597
rs1219898
rs1563165
rs133565
rs264976
rs954415
rs3019359
rs4762030
rs619656
rs2070614
rs12423629
rs17631826
rs2025852
rs881427
rs36009174
rs16877398
rs11191193
rs56101390
rs17537141
rs7958654
rs12982593
rs6504210
rs1046276
All
SNP
−0.01
0.00
0.01
0.02
Effect Size with 95% CI

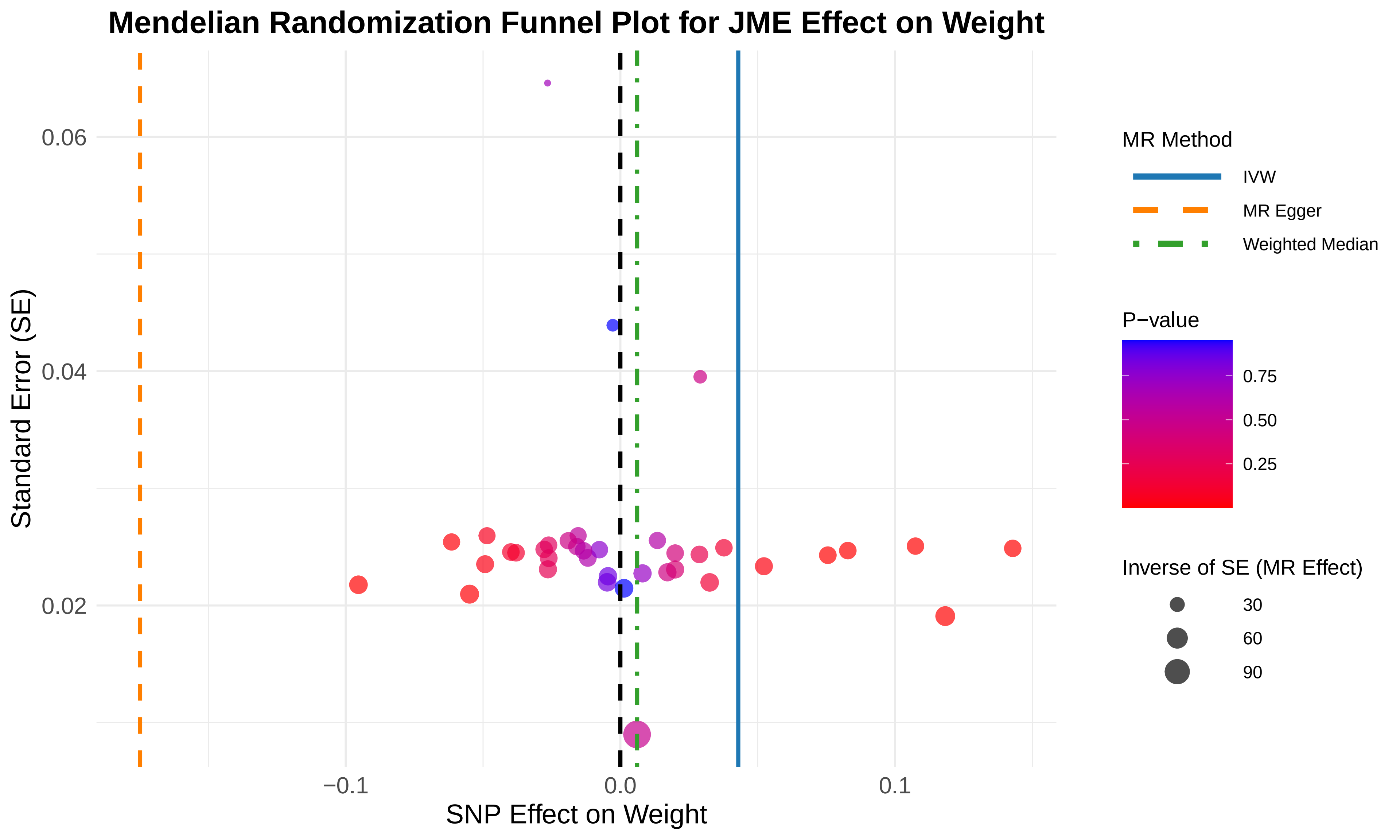
Mendelian Randomization Funnel Plot for JME Effect on Weight
Standard Error (SE)
0.06
0.04
0.02
−0.1
0.0
0.1
SNP Effect on Weight
MR Method
IVW
MR Egger
Weighted Median
P−value
0.75
0.50
0.25
Inverse of SE (MR Effect)
30
60
90

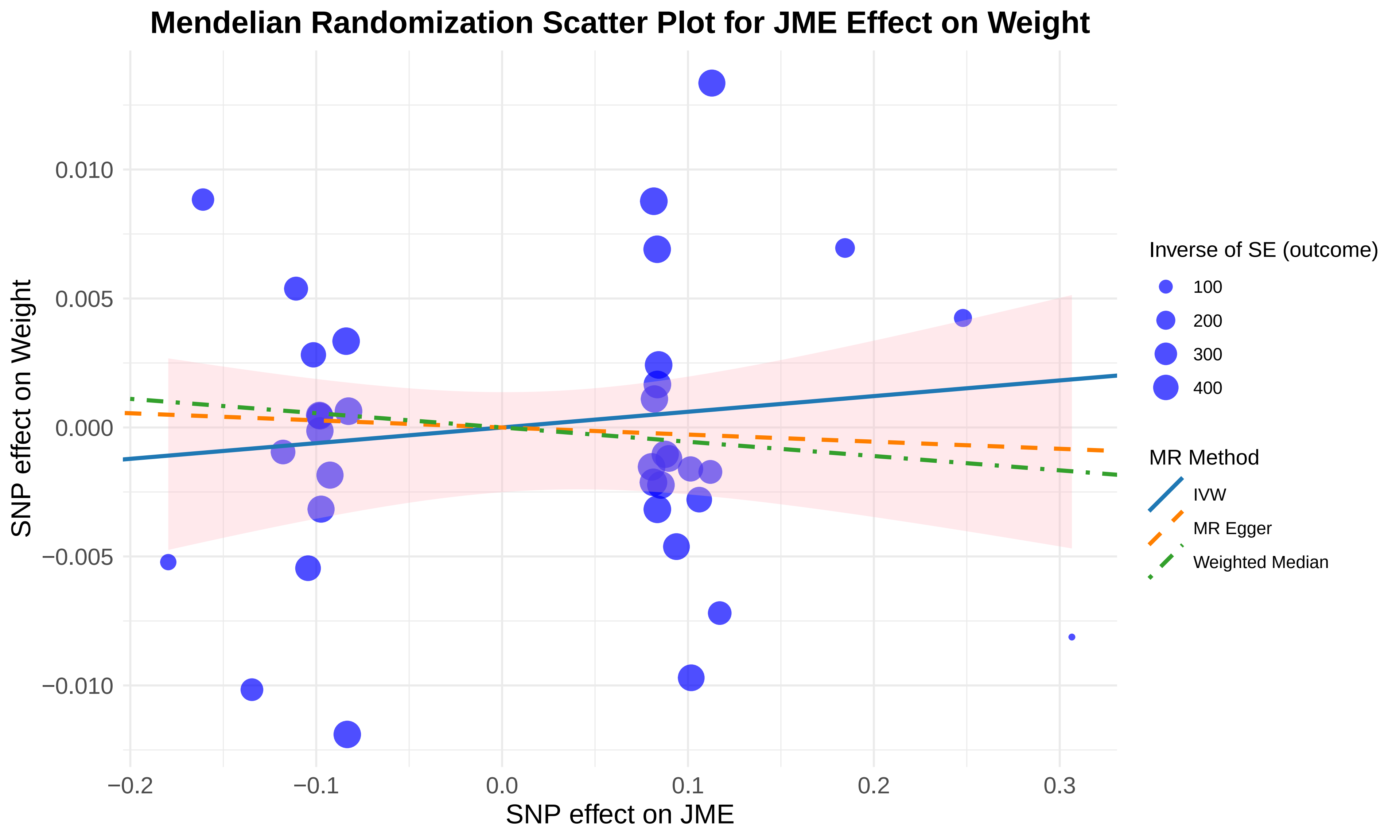
Mendelian Randomization Scatter Plot for JME Effect on Weight
SNP effect on Weight
SNP effect on JME
0.010
0.005
0.000
−0.005
−0.010
−0.2
−0.1
0.0
0.1
0.2
0.3
Inverse of SE (outcome)
100
200
300
400
MR Method
IVW
MR Egger
Weighted Median

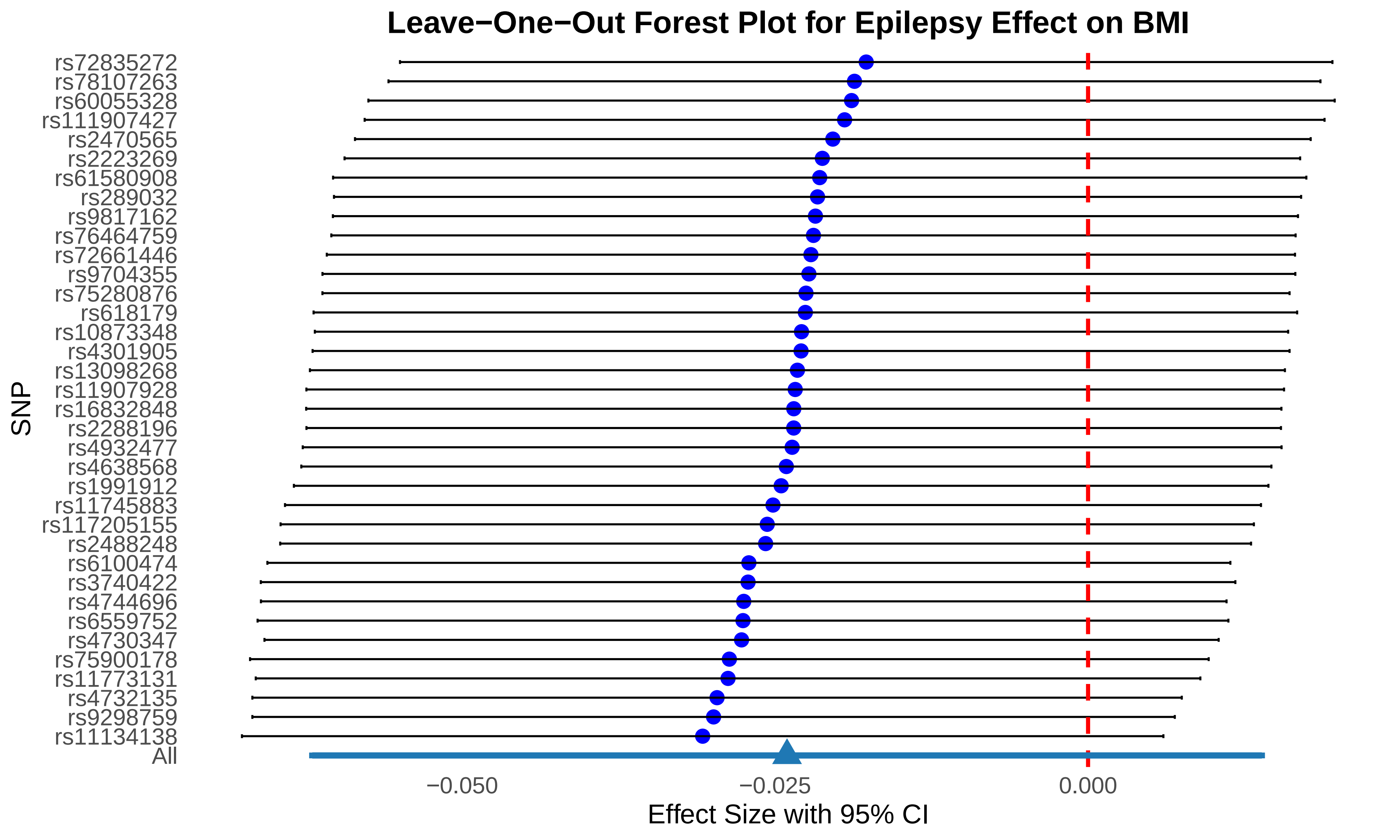
Leave−One−Out Forest Plot for Epilepsy Effect on BMI
SNP
rs72835272
rs78107263
rs60055328
rs111907427
rs2470565
rs2223269
rs61580908
rs289032
rs9817162
rs76464759
rs72661446
rs9704355
rs75280876
rs618179
rs10873348
rs4301905
rs13098268
rs11907928
rs16832848
rs2288196
rs4932477
rs4638568
rs1991912
rs11745883
rs117205155
rs2488248
rs6100474
rs3740422
rs4744696
rs6559752
rs4730347
rs75900178
rs11773131
rs4732135
rs9298759
rs11134138
All
−0.050
−0.025
0.000
Effect Size with 95% CI

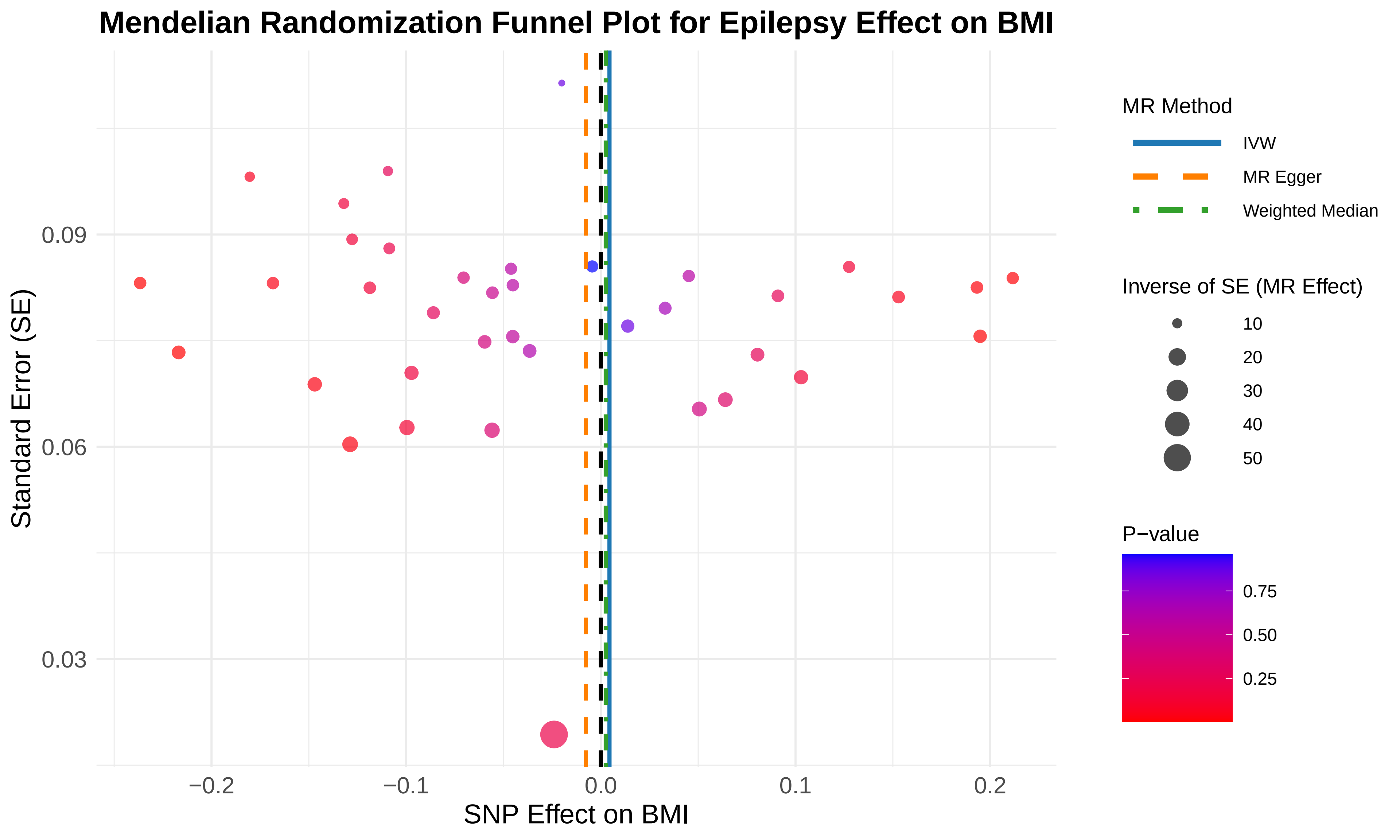
Mendelian Randomization Funnel Plot for Epilepsy Effect on BMI
Standard Error (SE)
0.09
0.06
0.03
−0.2
−0.1
0.0
0.1
0.2
SNP Effect on BMI
MR Method
IVW
MR Egger
Weighted Median
Inverse of SE (MR Effect)
10
20
30
40
50
P−value
0.75
0.50
0.25

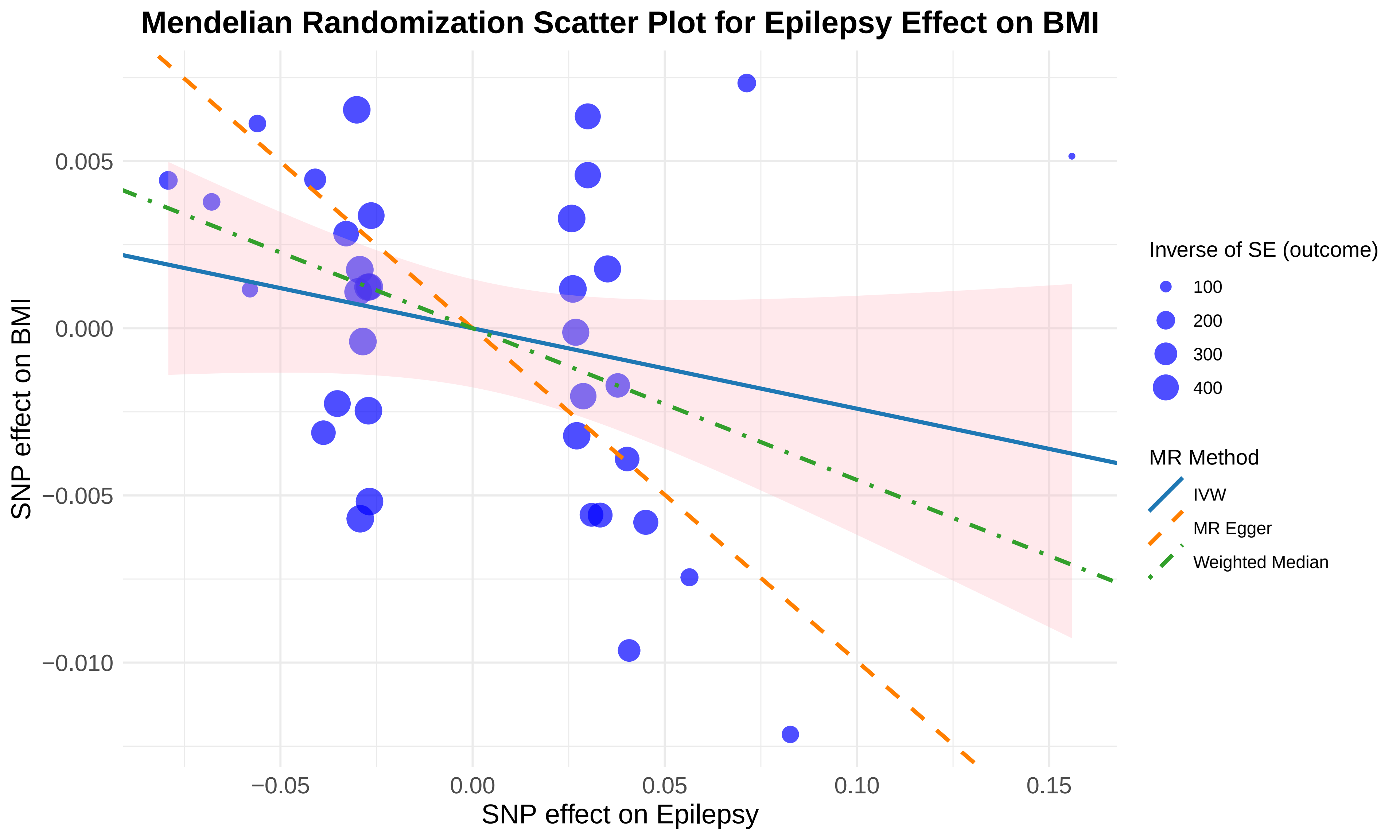

Mendelian Randomization Scatter Plot for Epilepsy Effect on BMI
SNP effect on BMI
0.005
0.000
−0.005
−0.010
SNP effect on Epilepsy
−0.05
0.00
0.05
0.10
0.15
Inverse of SE (outcome)
100
200
300
400
MR Method
IVW
MR Egger
Weighted Median

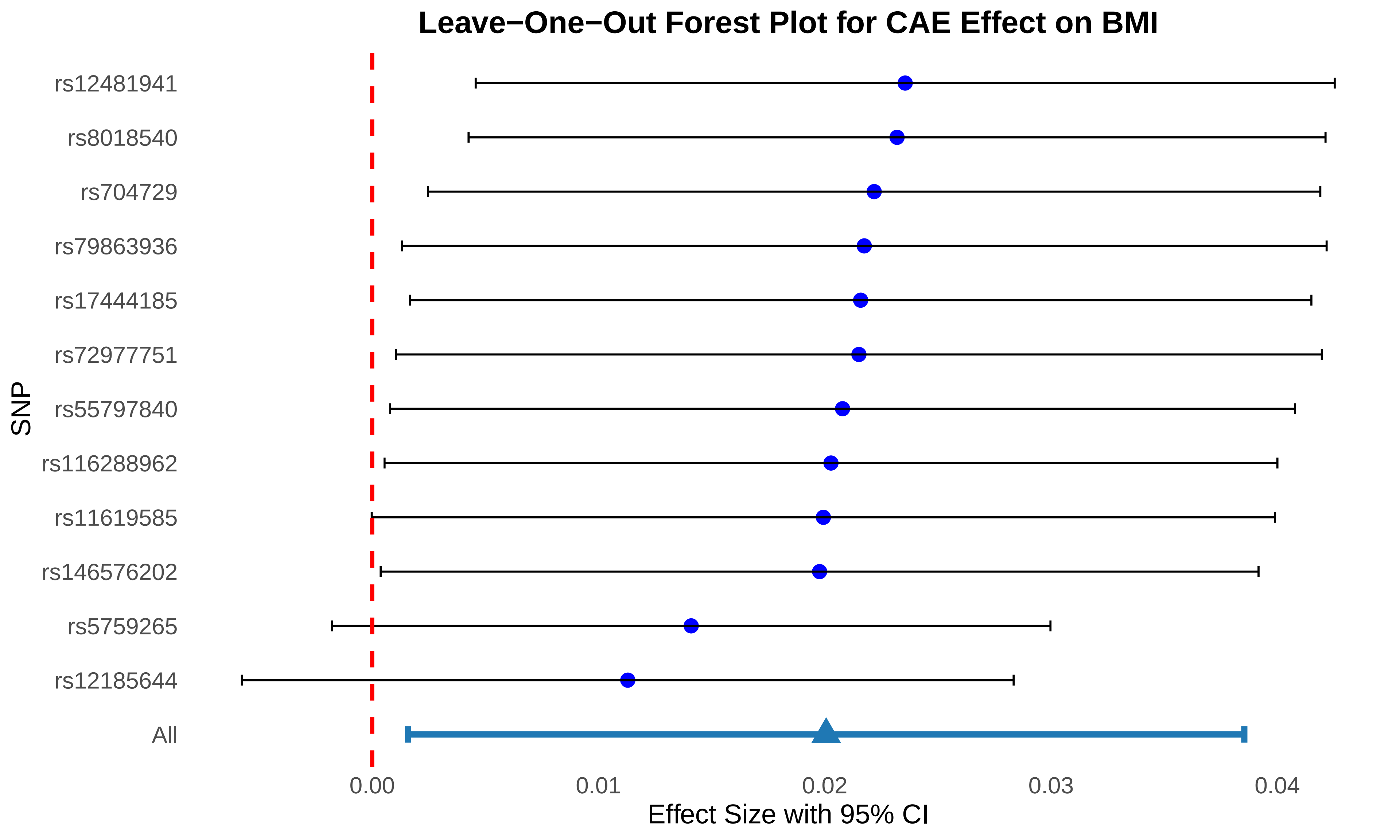

Leave−One−Out Forest Plot for CAE Effect on BMI
SNP
rs12481941
rs8018540
rs704729
rs79863936
rs17444185
rs72977751
rs55797840
rs116288962
rs11619585
rs146576202
rs5759265
rs12185644
All
0.00
0.01
0.02
0.03
0.04
Effect Size with 95% CI

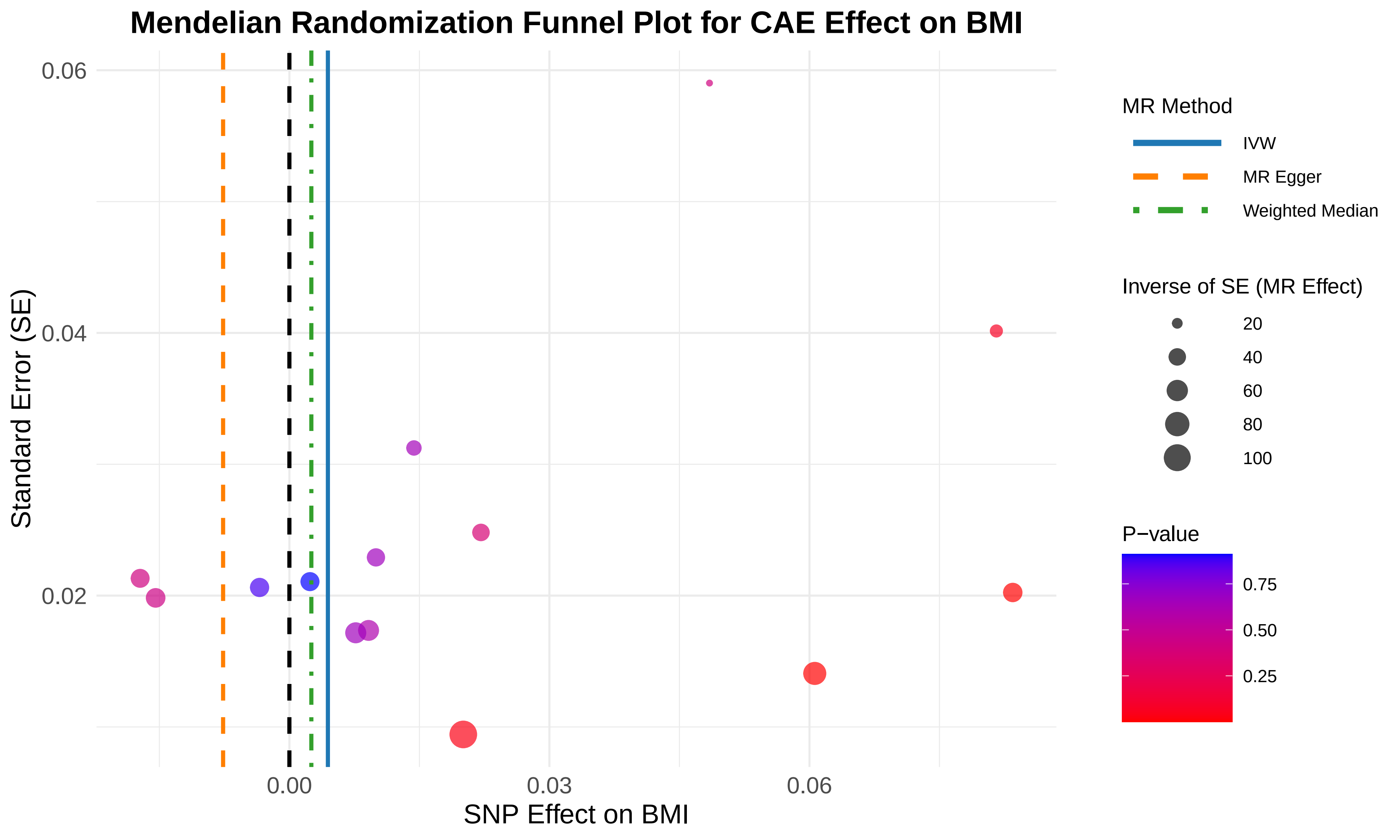

Mendelian Randomization Funnel Plot for CAE Effect on BMI
Standard Error (SE)
0.06
0.04
0.02
0.00
0.03
0.06
SNP Effect on BMI
MR Method
IVW
MR Egger
Weighted Median
Inverse of SE (MR Effect)
20
40
60
80
100
P−value
0.75
0.50
0.25

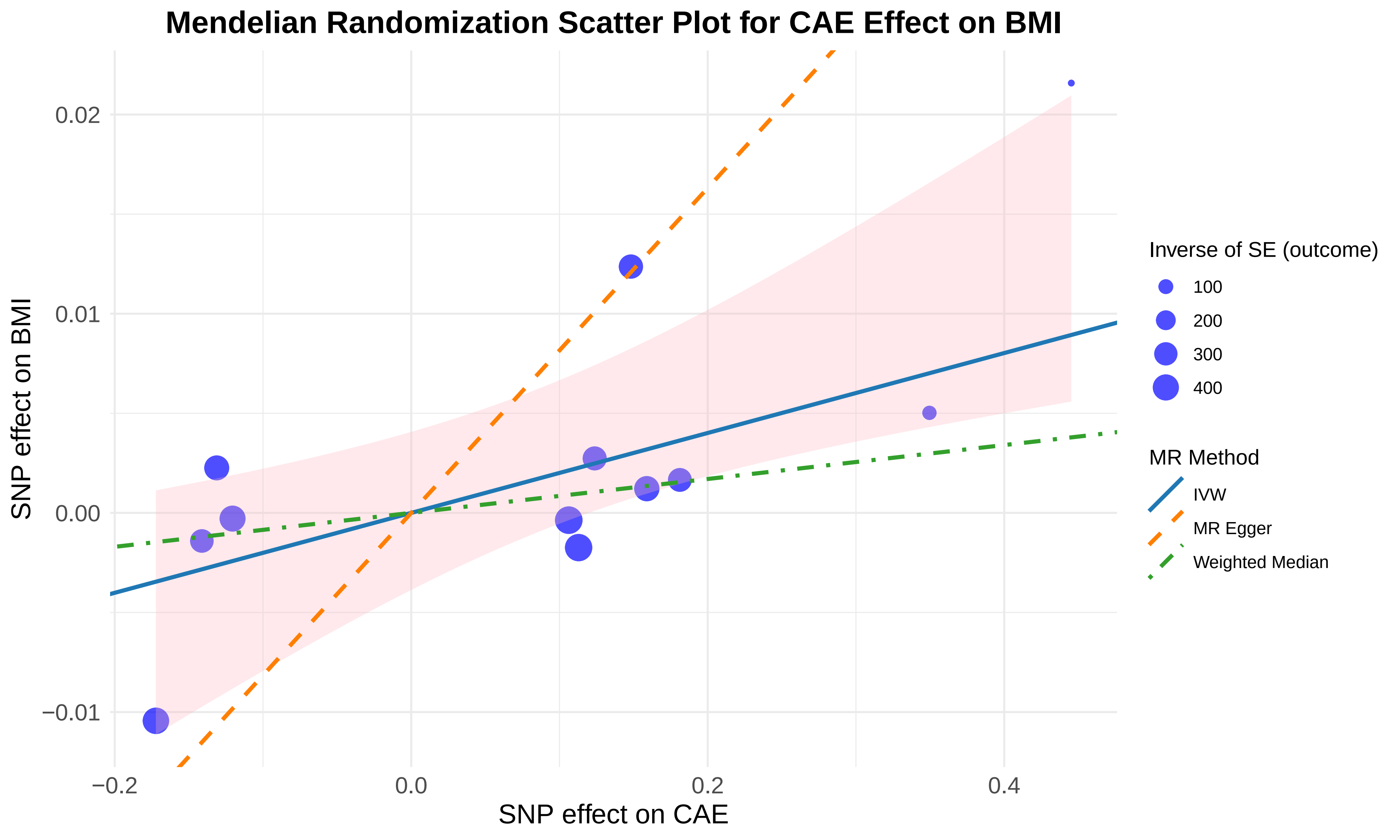

Mendelian Randomization Scatter Plot for CAE Effect on BMI
0.02
0.01
0.00
−0.01
SNP effect on BMI
−0.2
0.0
0.2
0.4
SNP effect on CAE
Inverse of SE (outcome)
100
200
300
400
MR Method
IVW
MR Egger
Weighted Median

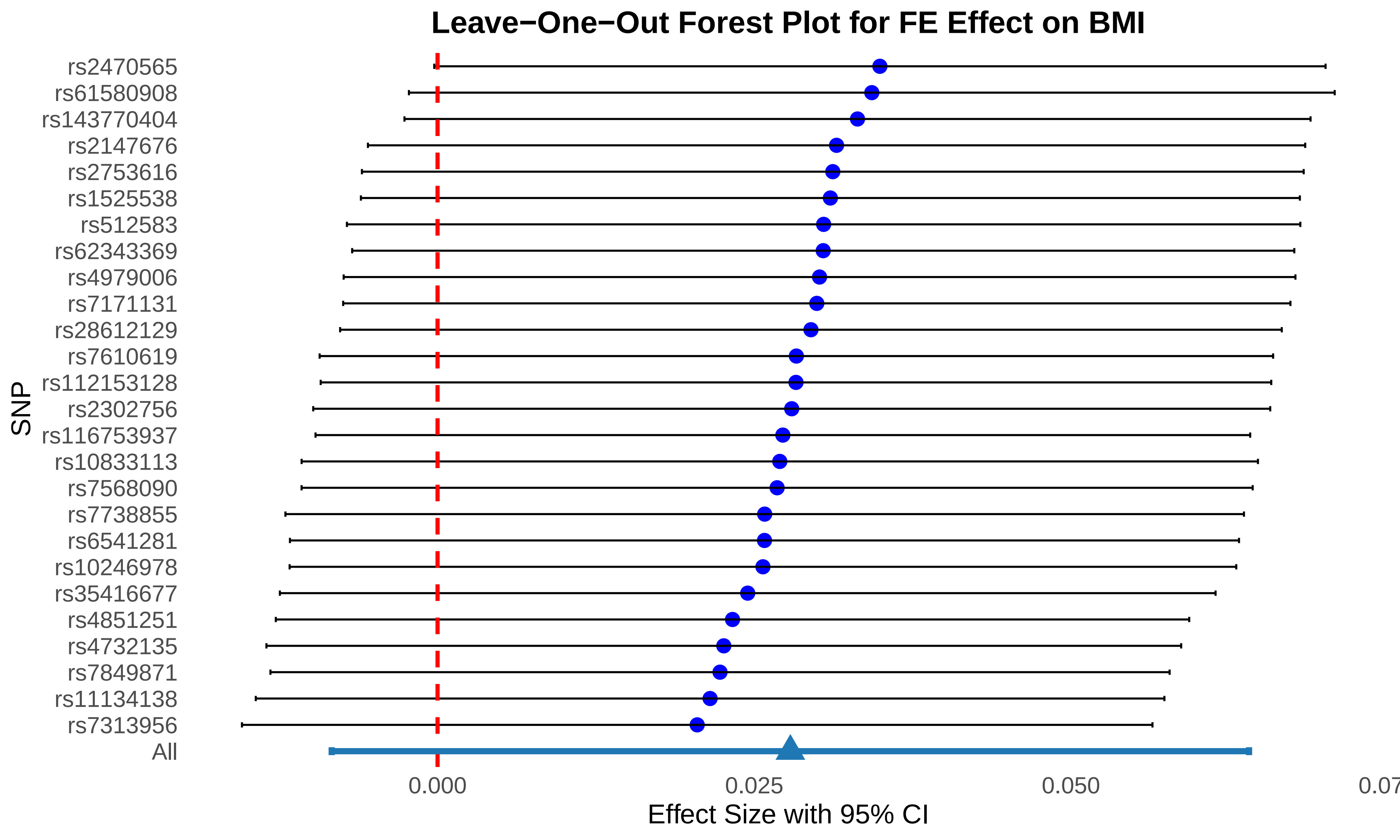

Leave−One−Out Forest Plot for FE Effect on BMI
SNP
rs2470565
rs61580908
rs143770404
rs2147676
rs2753616
rs1525538
rs512583
rs62343369
rs4979006
rs7171131
rs28612129
rs7610619
rs112153128
rs2302756
rs116753937
rs10833113
rs7568090
rs7738855
rs6541281
rs10246978
rs35416677
rs4851251
rs4732135
rs7849871
rs11134138
rs7313956
All
0.000
0.025
0.050
Effect Size with 95% CI

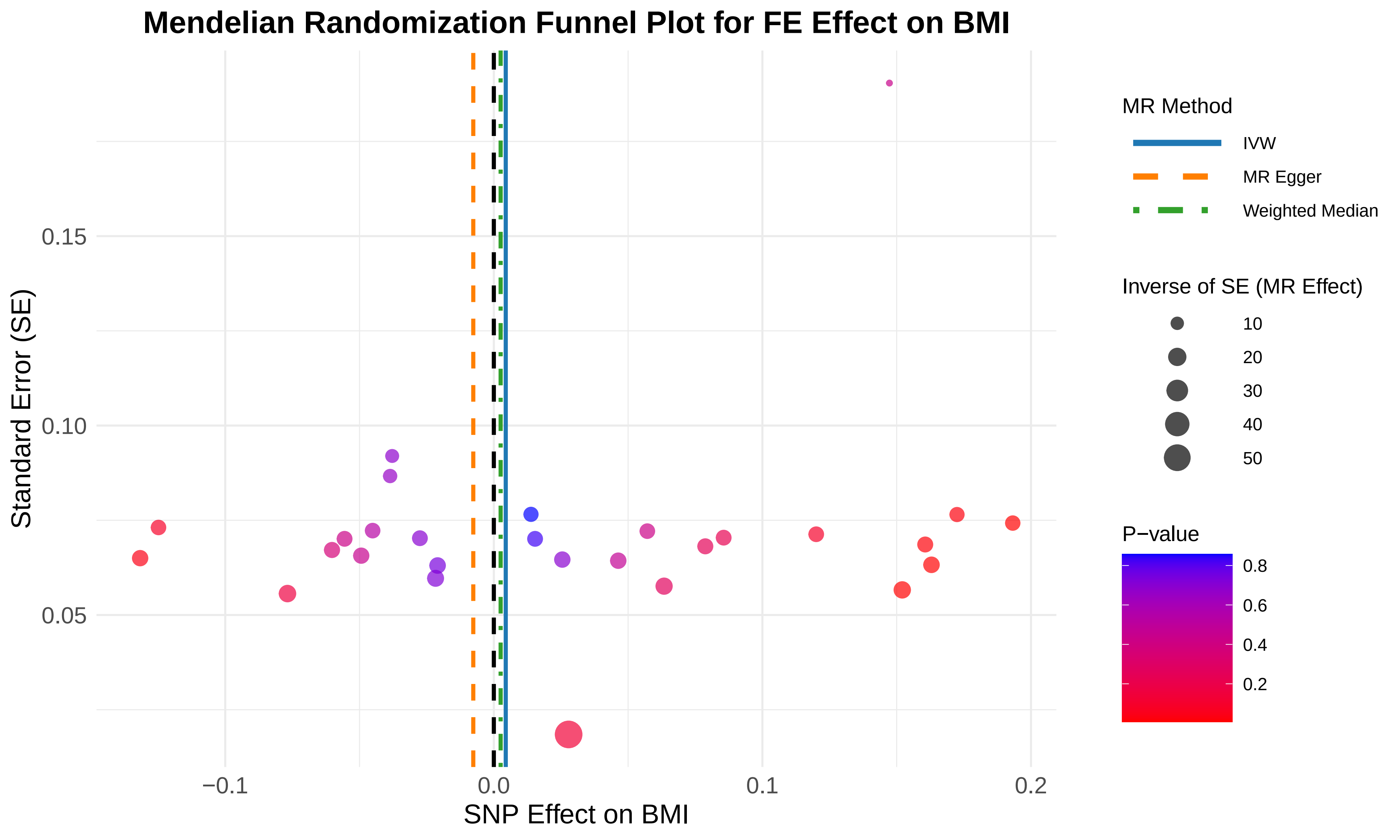
Mendelian Randomization Funnel Plot for FE Effect on BMI
Standard Error (SE)
0.15
0.10
0.05
−0.1
0.0
0.1
0.2
SNP Effect on BMI
MR Method
IVW
MR Egger
Weighted Median
Inverse of SE (MR Effect)
10
20
30
40
50
P−value
0.8
0.6
0.4
0.2

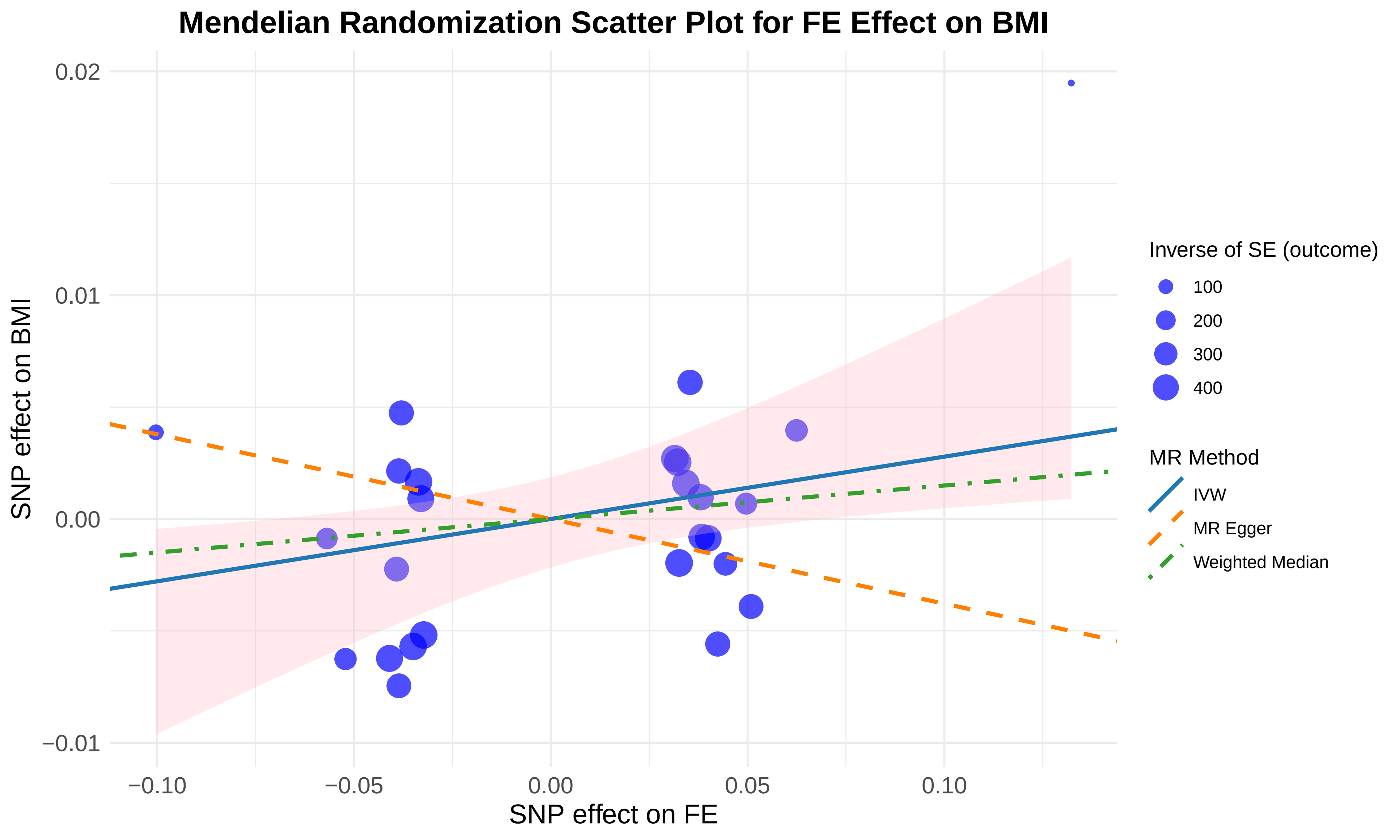
Mendelian Randomization Scatter Plot for FE Effect on BMI
0.02
0.01
0.00
−0.01
SNP effect on BMI
−0.10
−0.05
0.00
0.05
0.10
SNP effect on FE
Inverse of SE (outcome)
100
200
300
400
MR Method
IVW
MR Egger
Weighted Median

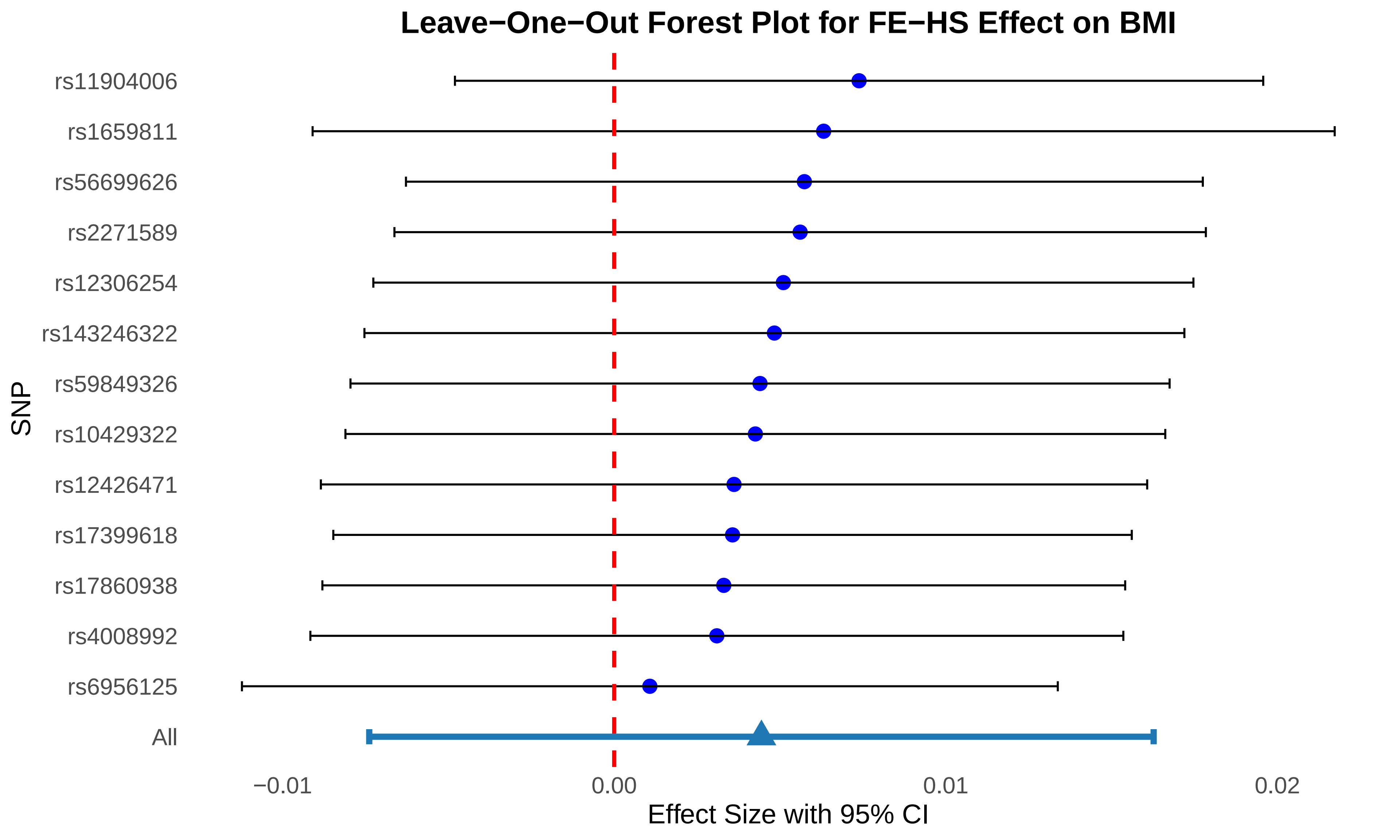
Leave−One−Out Forest Plot for FE−HS Effect on BMI
rs11904006
rs1659811
rs56699626
rs2271589
rs12306254
rs143246322
rs59849326
rs10429322
rs12426471
rs17399618
rs17860938
rs4008992
rs6956125
All
SNP
−0.01
0.00
0.01
0.02
Effect Size with 95% CI

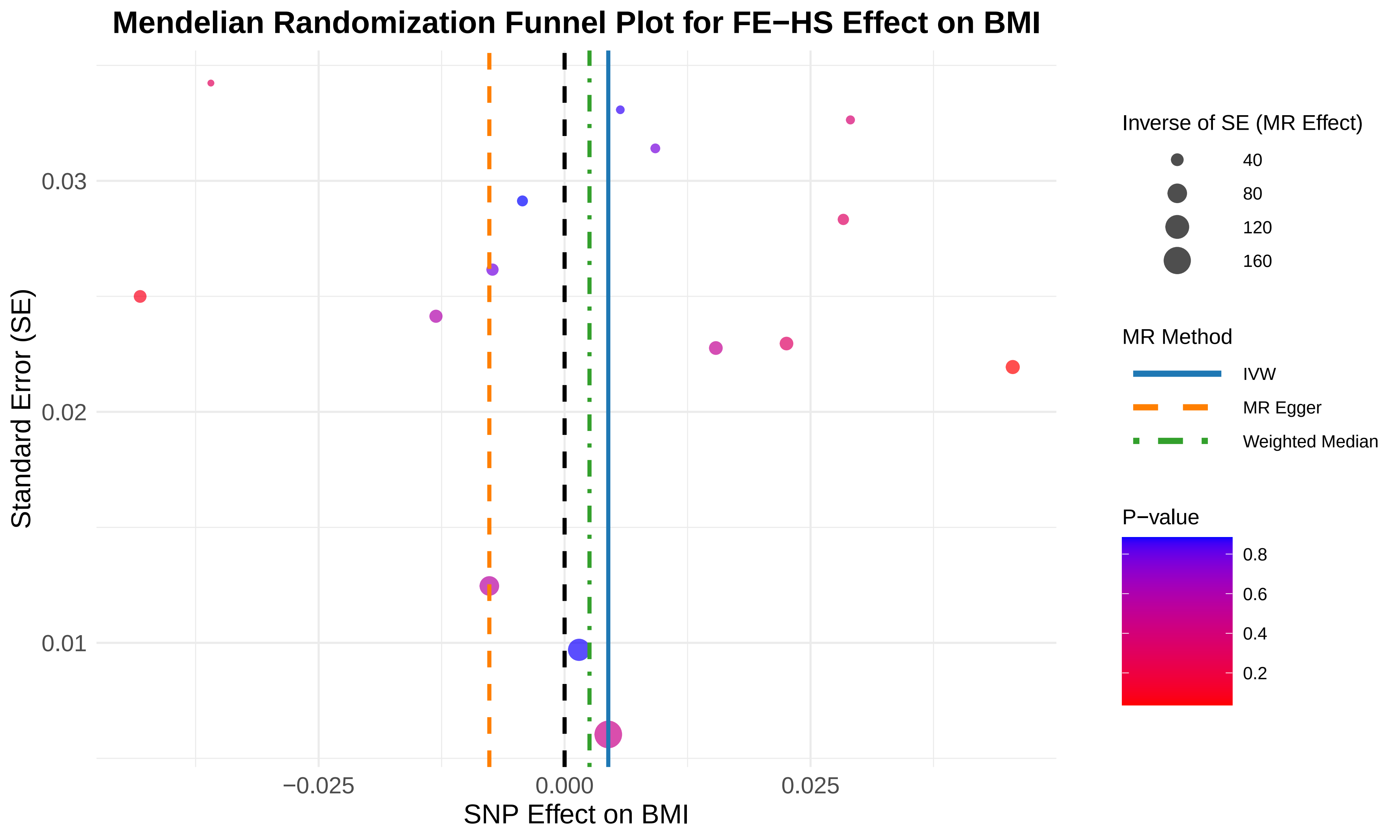
Mendelian Randomization Funnel Plot for FE−HS Effect on BMI
Standard Error (SE)
0.03
0.02
0.01
−0.025
0.000
0.025
SNP Effect on BMI
Inverse of SE (MR Effect)
40
80
120
160
MR Method
IVW
MR Egger
Weighted Median
P−value
0.8
0.6
0.4
0.2

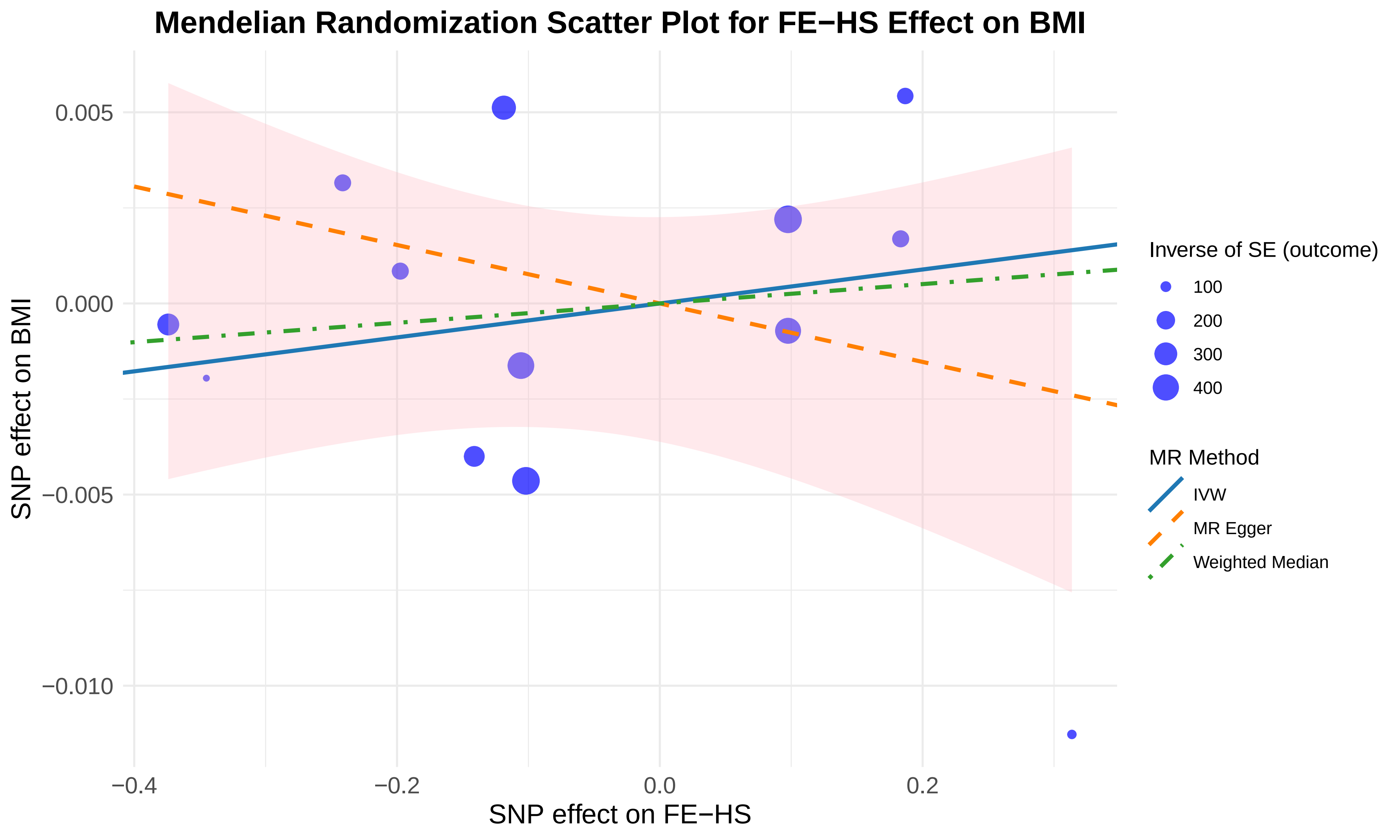
Mendelian Randomization Scatter Plot for FE−HS Effect on BMI
SNP effect on BMI
SNP effect on FE−HS
0.005
0.000
−0.005
−0.010
−0.4
−0.2
0.0
0.2
Inverse of SE (outcome)
100
200
300
400
MR Method
IVW
MR Egger
Weighted Median

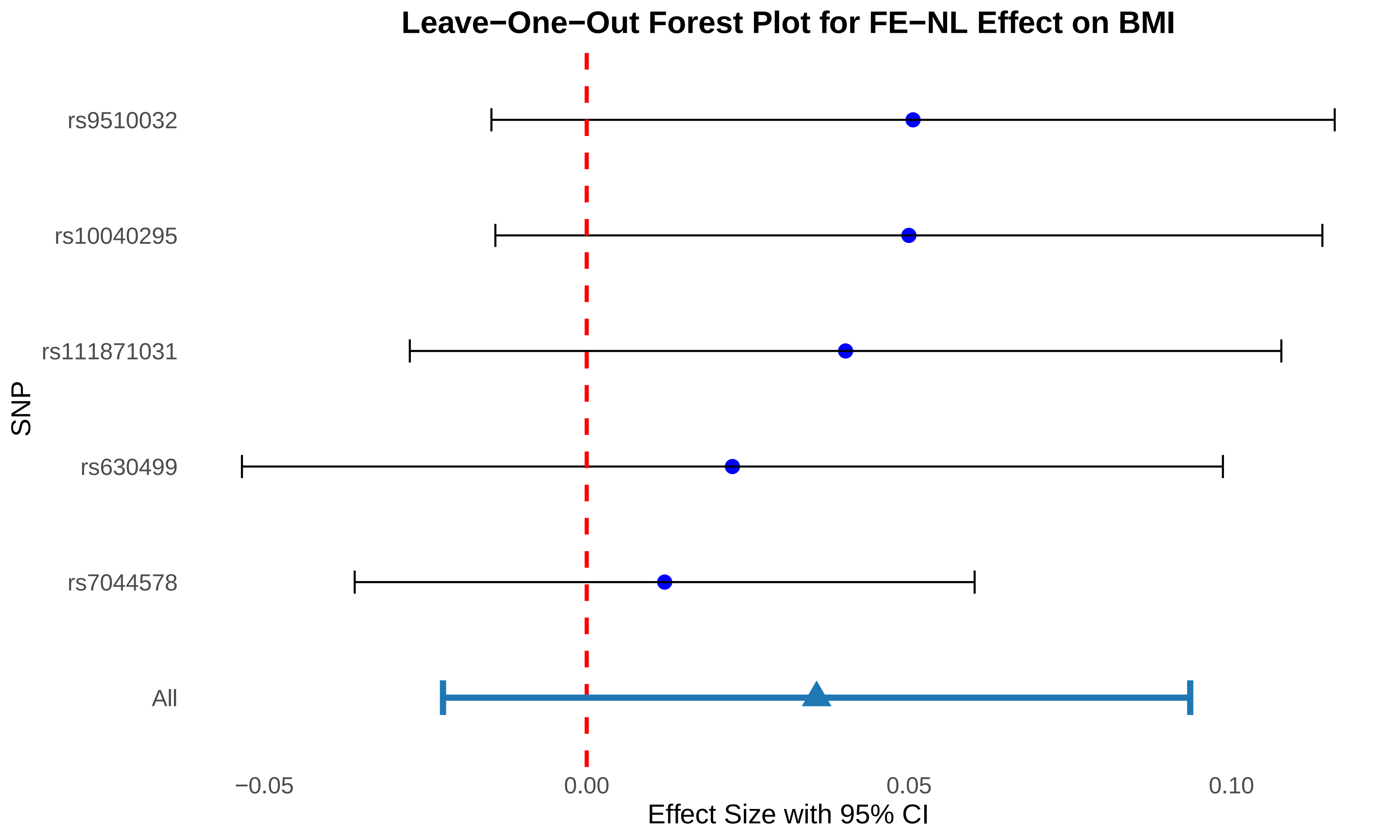
Leave−One−Out Forest Plot for FE−NL Effect on BMI
rs9510032
rs10040295
rs111871031
rs630499
rs7044578
All
SNP
−0.05
0.00
0.05
0.10
Effect Size with 95% CI

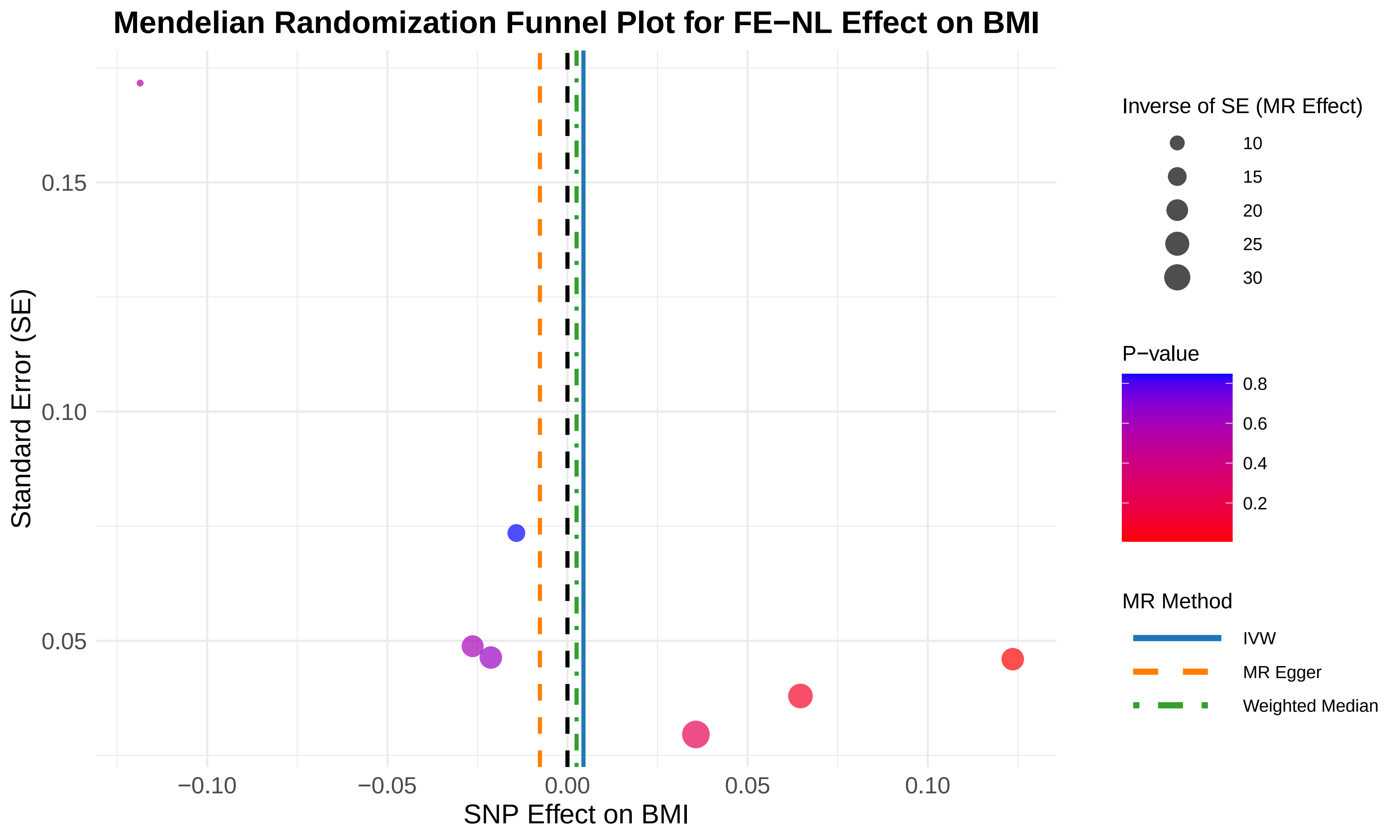
Mendelian Randomization Funnel Plot for FE−NL Effect on BMI
Standard Error (SE)
0.15
0.10
0.05
−0.10
−0.05
0.00
0.05
0.10
SNP Effect on BMI
Inverse of SE (MR Effect)
10
15
20
25
30
P−value
0.8
0.6
0.4
0.2
MR Method
IVW
MR Egger
Weighted Median

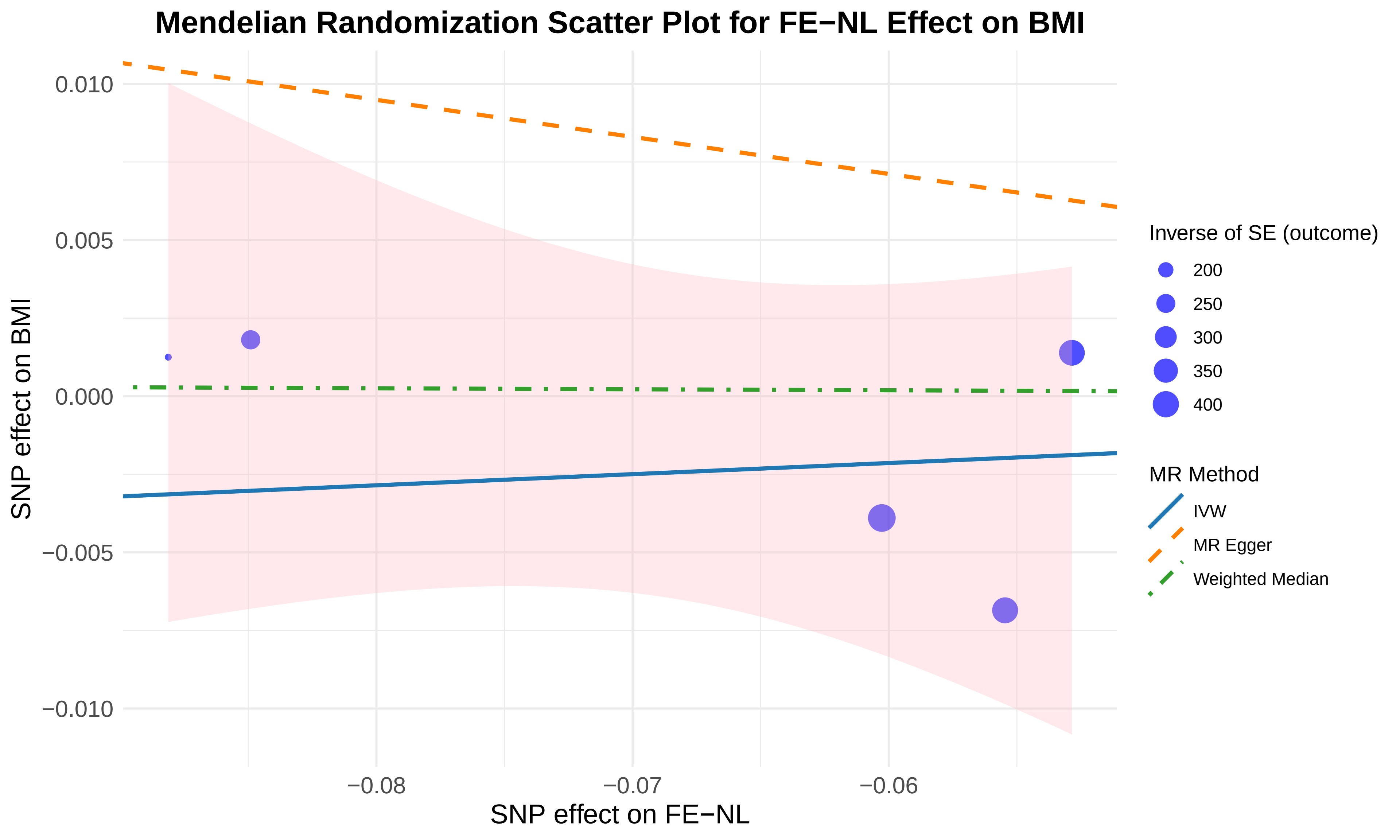
Mendelian Randomization Scatter Plot for FE−NL Effect on BMI
0.010
0.005
0.000
−0.005
−0.010
SNP effect on BMI
−0.08
−0.07
−0.06
SNP effect on FE−NL
Inverse of SE (outcome)
200
250
300
350
400
MR Method
IVW
MR Egger
Weighted Median

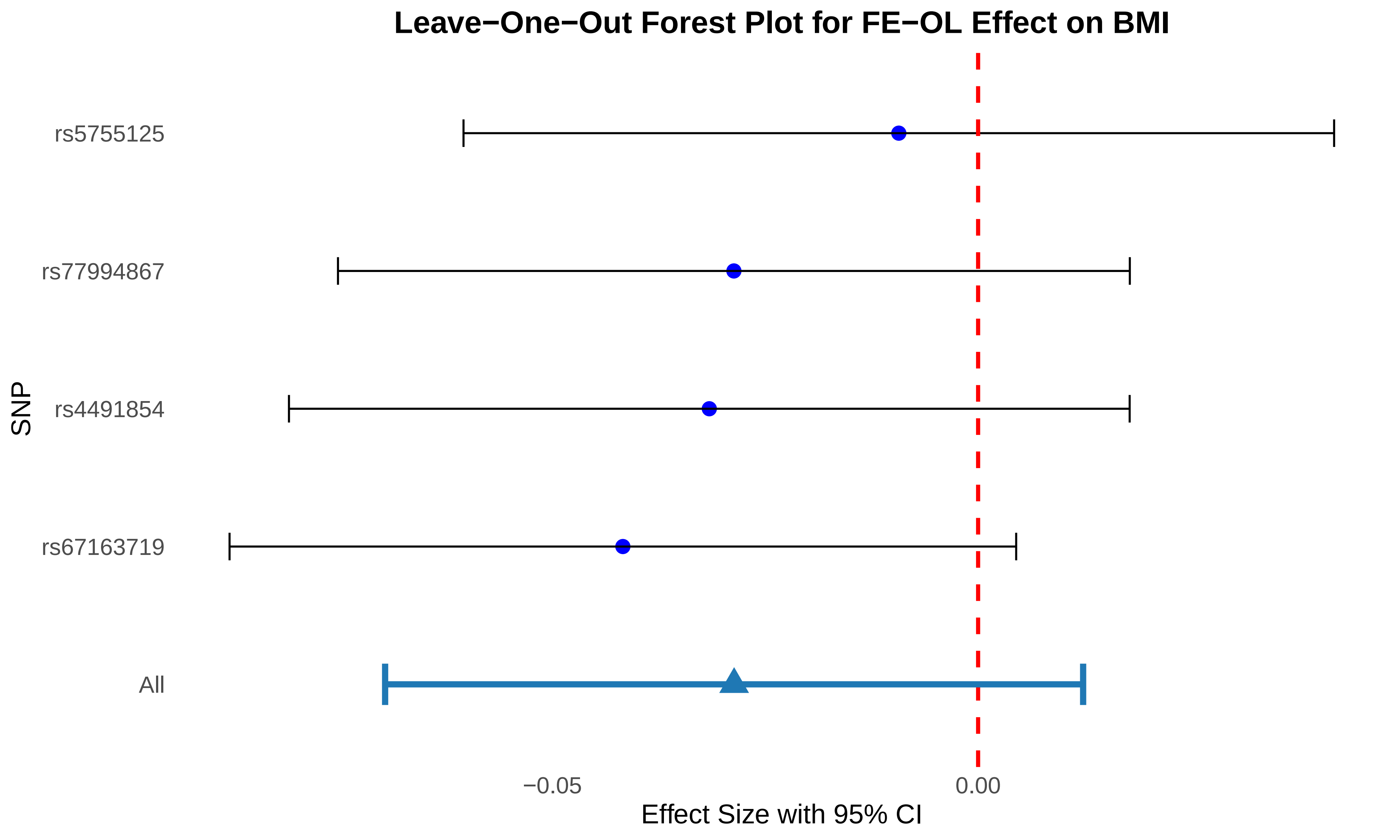
Leave−One−Out Forest Plot for FE−OL Effect on BMI
rs5755125
rs77994867
rs4491854
rs67163719
All
SNP
−0.05
0.00
Effect Size with 95% CI

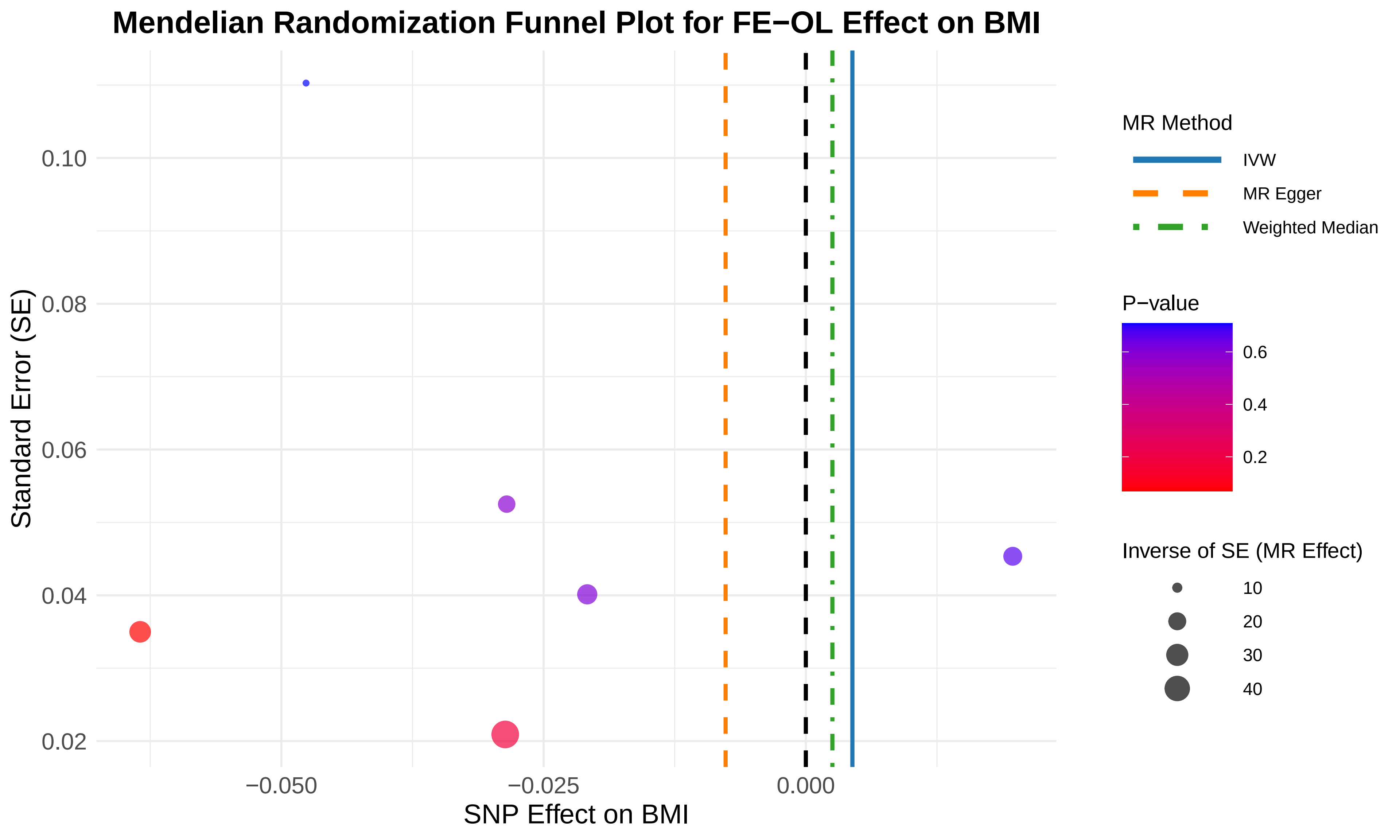
Mendelian Randomization Funnel Plot for FE−OL Effect on BMI
Standard Error (SE)
0.10
0.08
0.06
0.04
0.02
−0.050
−0.025
0.000
SNP Effect on BMI
MR Method
IVW
MR Egger
Weighted Median
P−value
0.6
0.4
0.2
Inverse of SE (MR Effect)
10
20
30
40

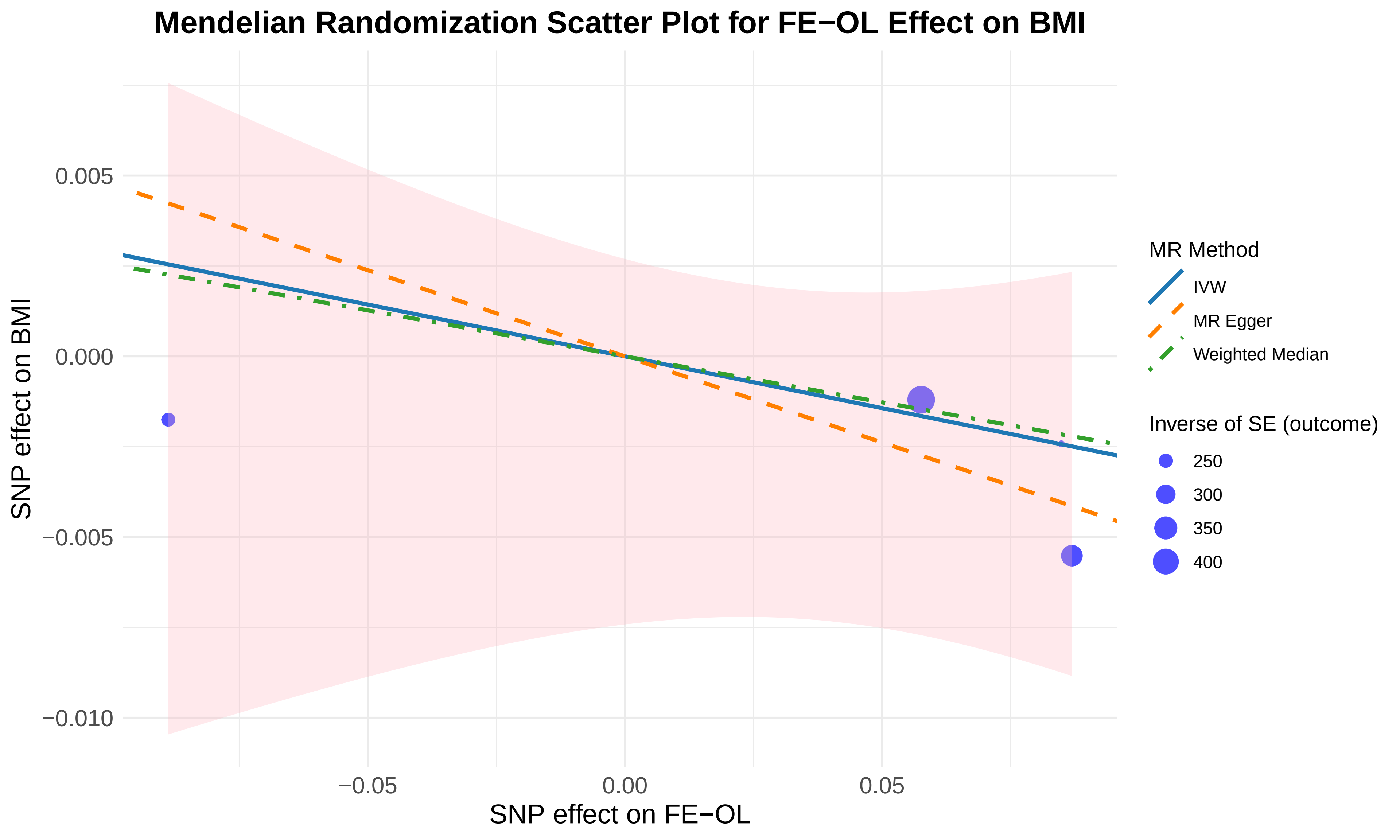
Mendelian Randomization Scatter Plot for FE−OL Effect on BMI
0.005
0.000
−0.005
−0.010
SNP effect on BMI
−0.05
0.00
0.05
SNP effect on FE−OL
MR Method
IVW
MR Egger
Weighted Median
Inverse of SE (outcome)
250
300
350
400

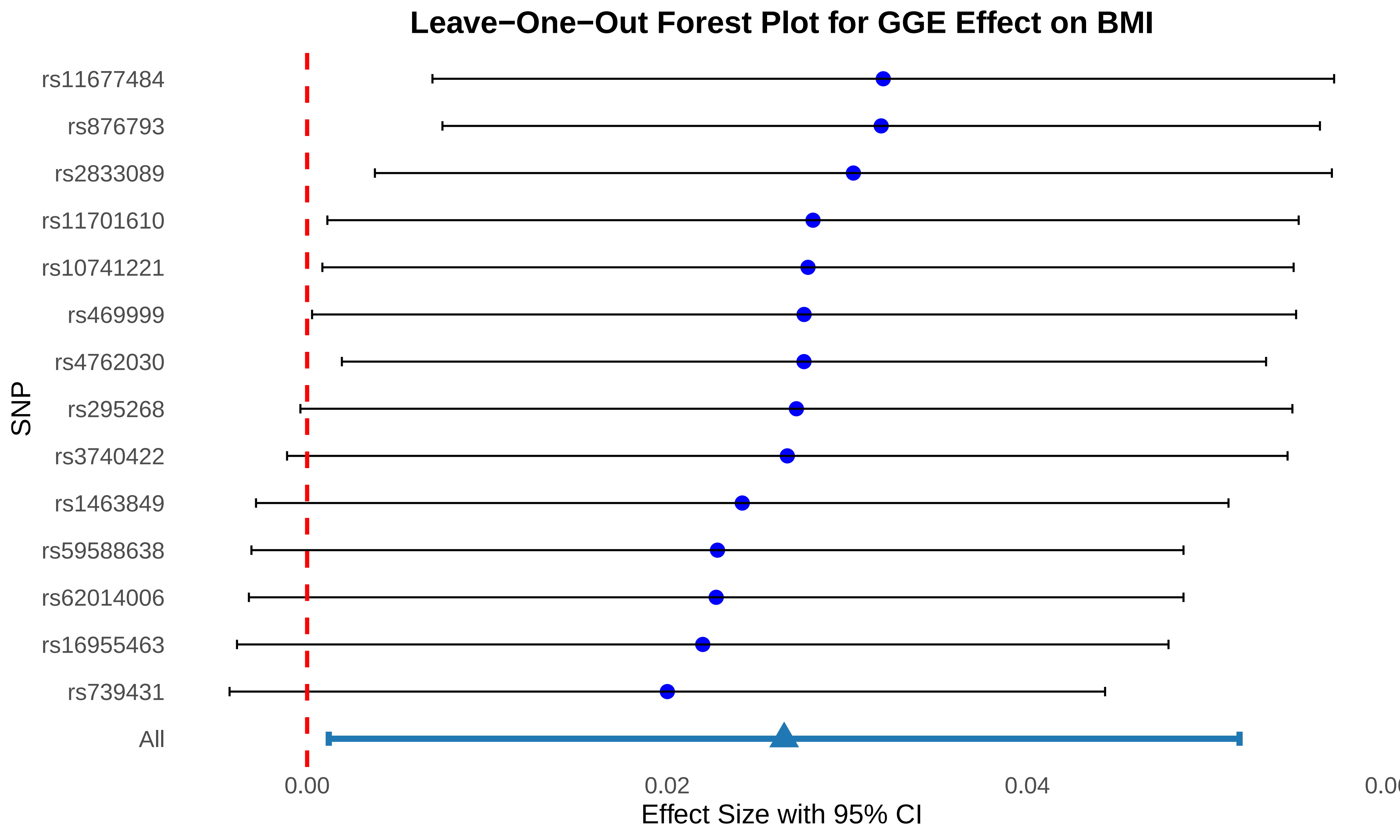

Leave−One−Out Forest Plot for GGE Effect on BMI
rs11677484
rs876793
rs2833089
rs11701610
rs10741221
rs469999
rs4762030
rs295268
rs3740422
rs1463849
rs59588638
rs62014006
rs16955463
rs739431
All
SNP
0.00
0.02
0.04
Effect Size with 95% CI

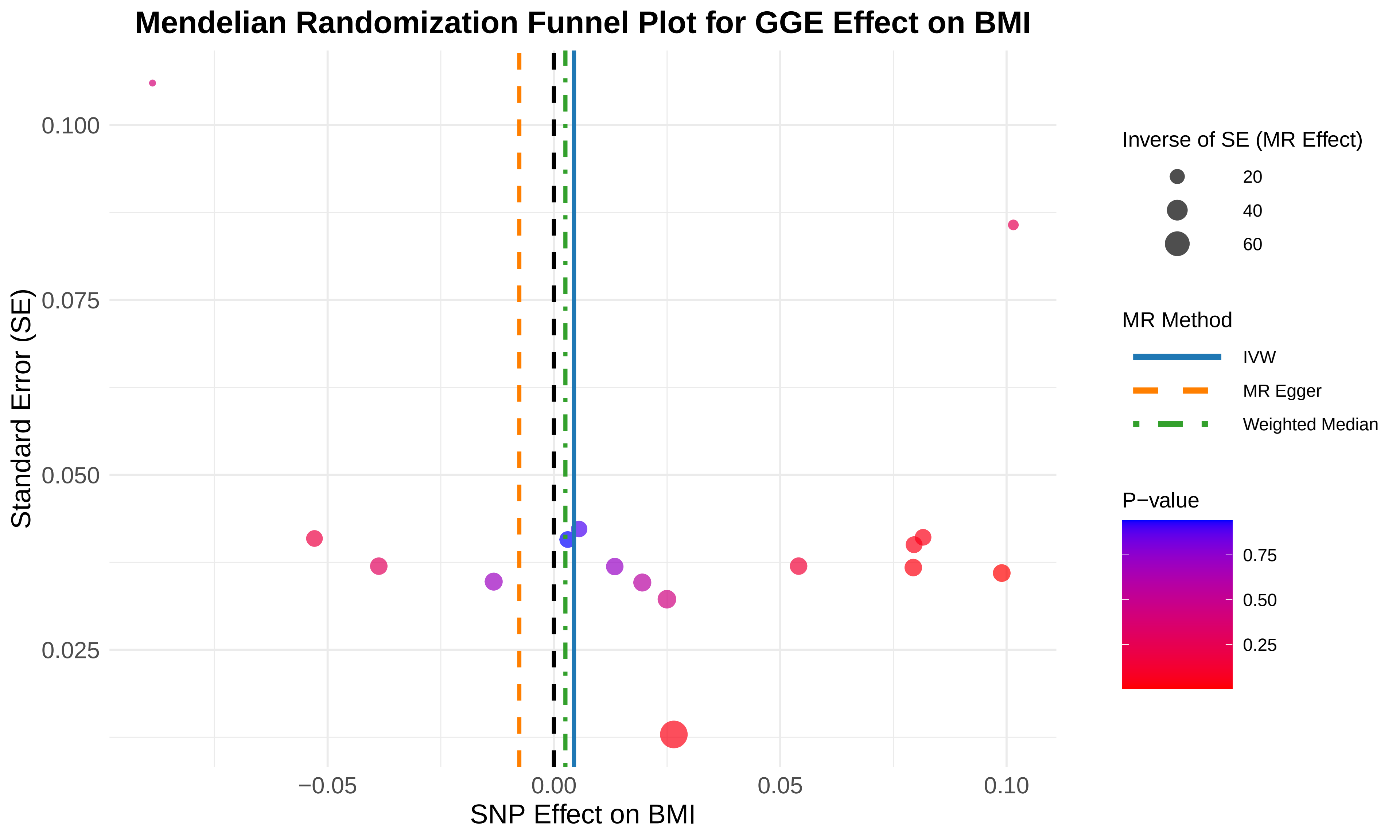

Mendelian Randomization Funnel Plot for GGE Effect on BMI
0.100
0.075
0.050
0.025
Standard Error (SE)
−0.05
0.00
0.05
0.10
SNP Effect on BMI
Inverse of SE (MR Effect)
20
40
60
MR Method
IVW
MR Egger
Weighted Median
P−value
0.75
0.50
0.25

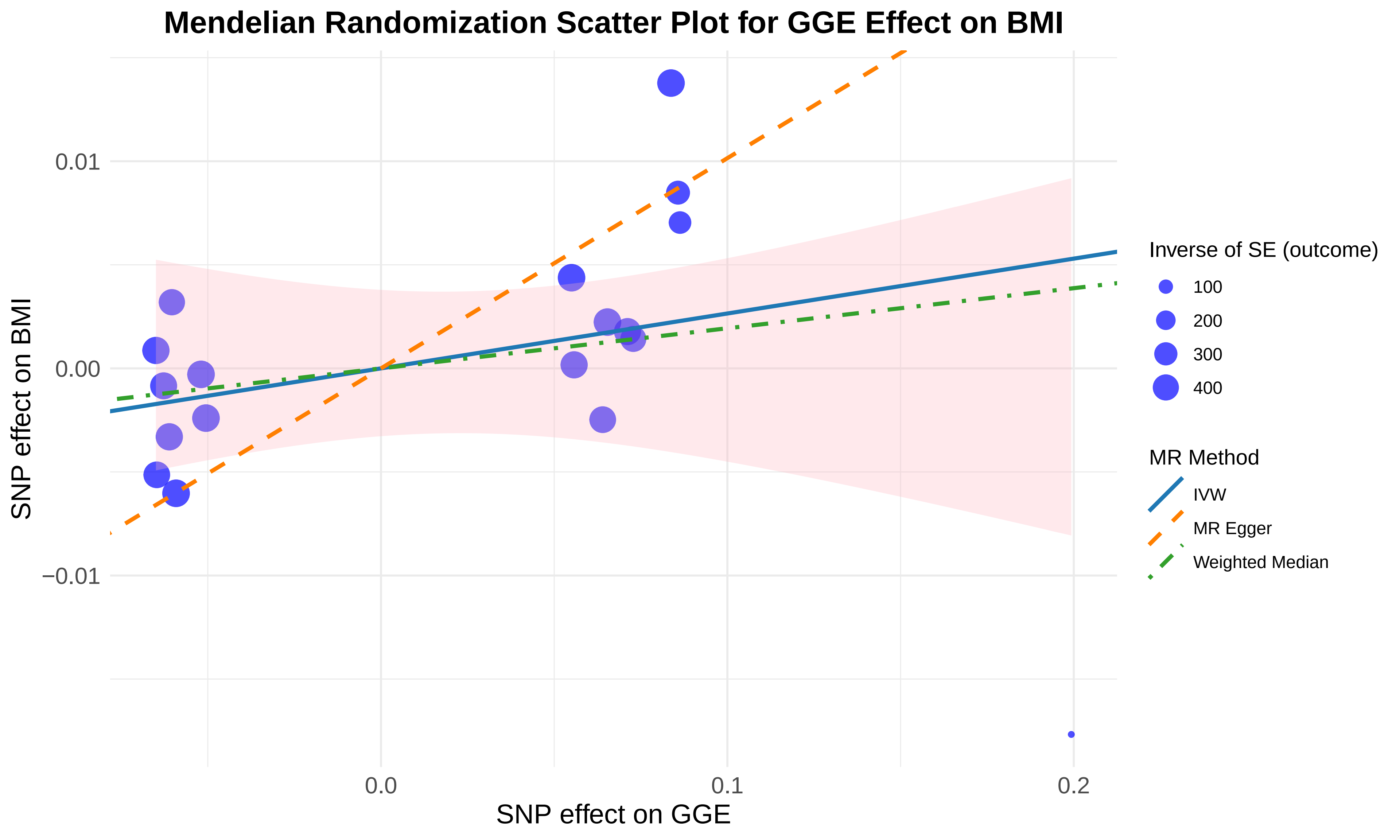
Mendelian Randomization Scatter Plot for GGE Effect on BMI
0.01
0.00
−0.01
SNP effect on BMI
0.0
0.1
0.2
SNP effect on GGE
Inverse of SE (outcome)
100
200
300
400
MR Method
IVW
MR Egger
Weighted Median

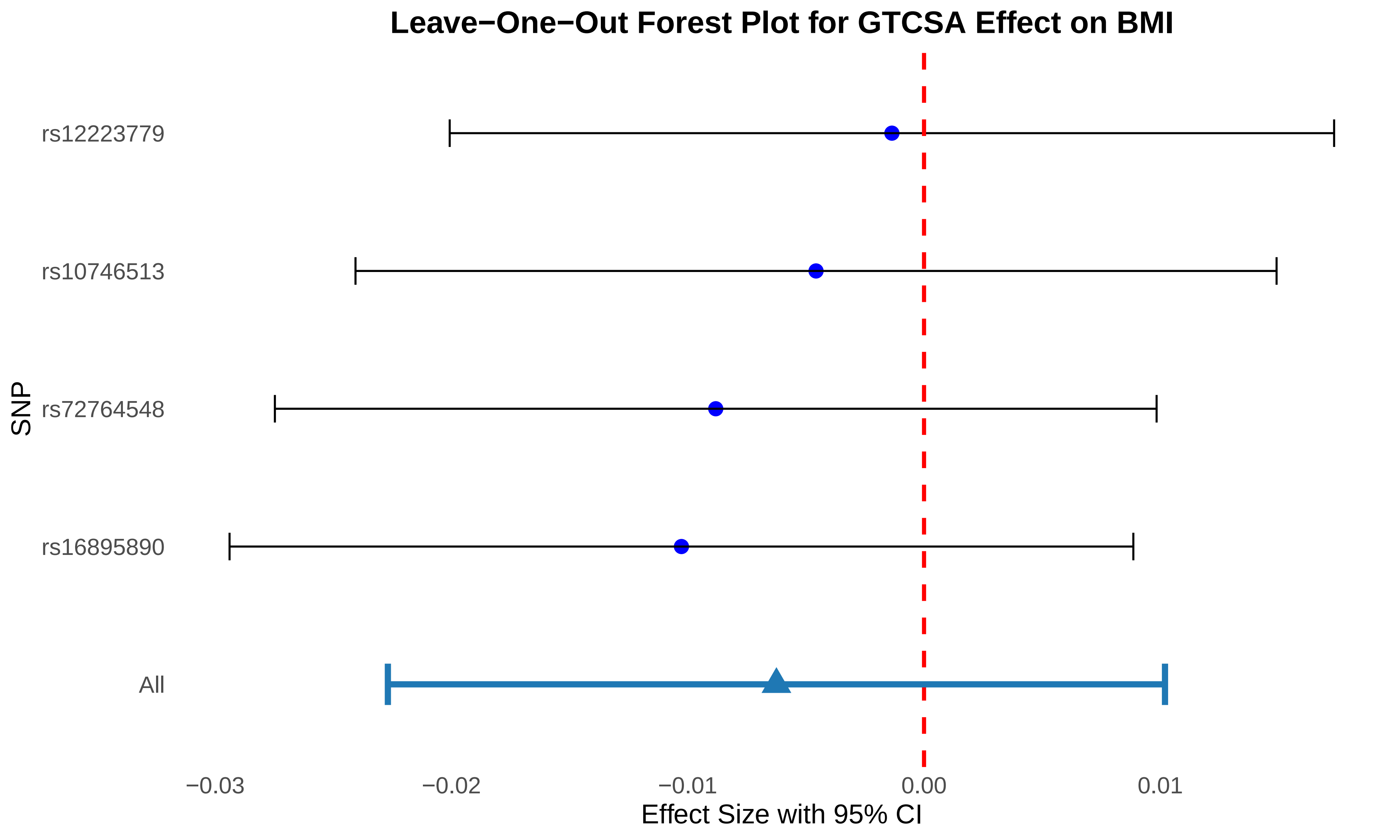

Leave−One−Out Forest Plot for GTCSA Effect on BMI
rs12223779
rs10746513
rs72764548
rs16895890
All
SNP
−0.03
−0.02
−0.01
0.00
0.01
Effect Size with 95% CI

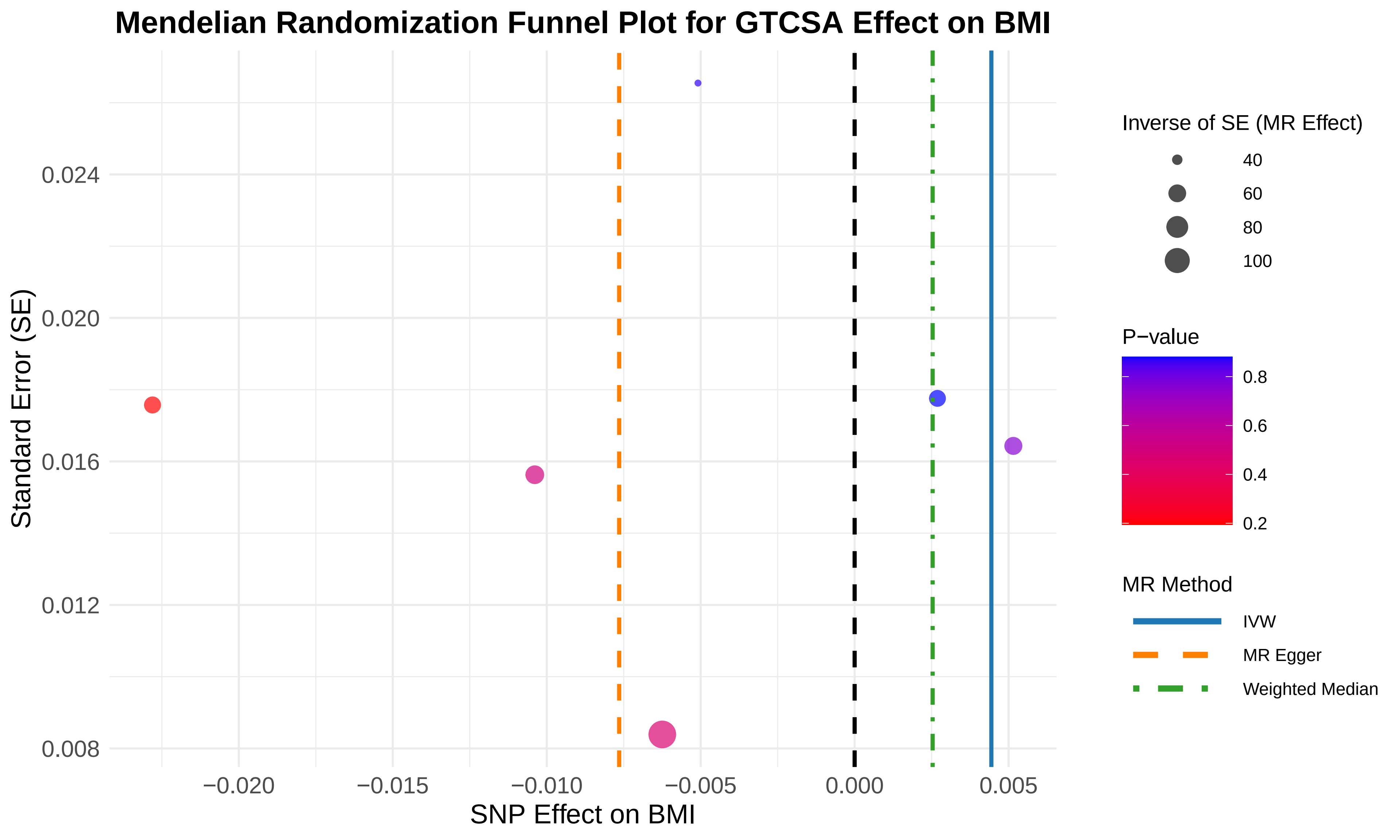
Mendelian Randomization Funnel Plot for GTCSA Effect on BMI
Standard Error (SE)
0.024
0.020
0.016
0.012
0.008
−0.020
−0.015
−0.010
−0.005
0.000
0.005
SNP Effect on BMI
Inverse of SE (MR Effect)
40
60
80
100
P−value
0.8
0.6
0.4
0.2
MR Method
IVW
MR Egger
Weighted Median

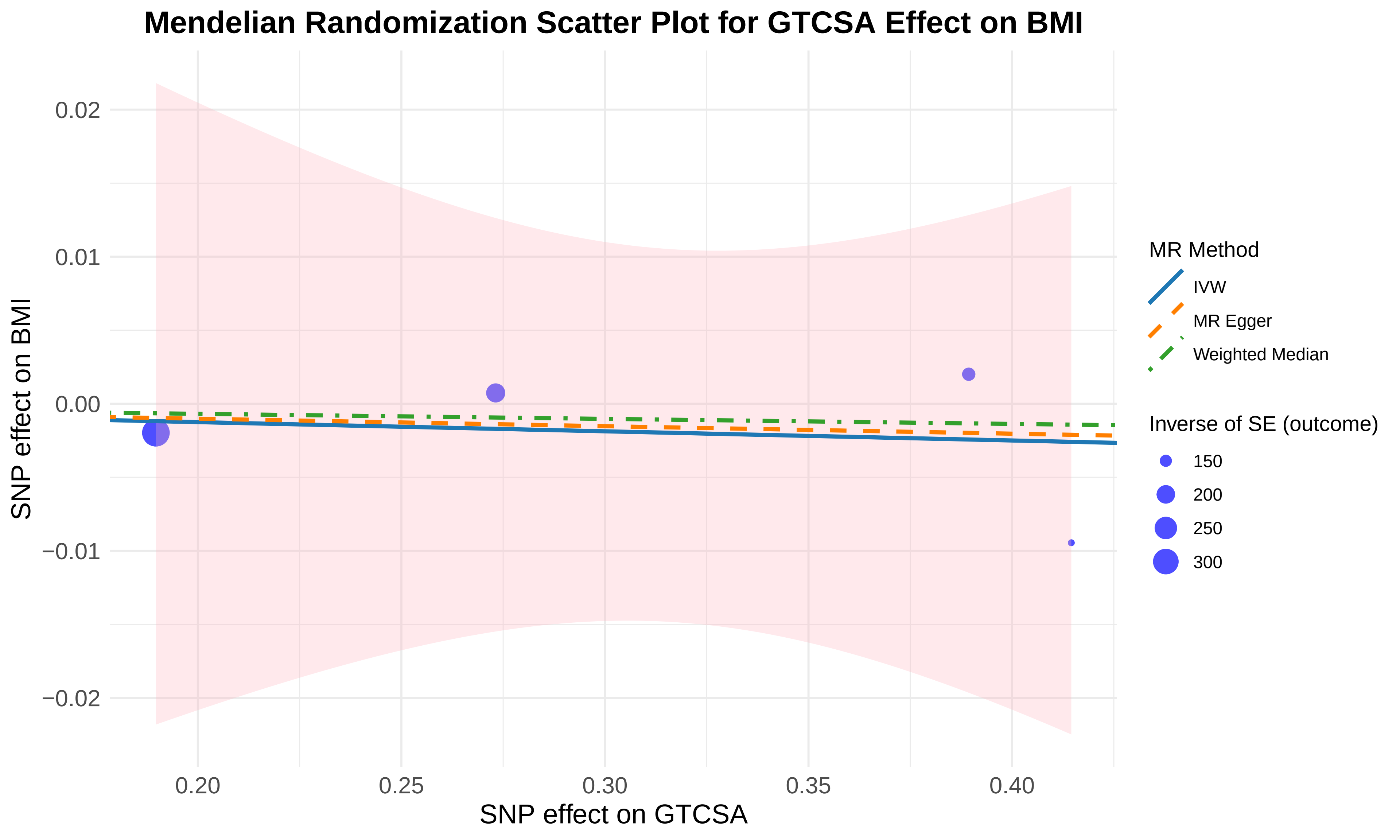
Mendelian Randomization Scatter Plot for GTCSA Effect on BMI
0.02
0.01
0.00
−0.01
−0.02
SNP effect on BMI
0.20
0.25
0.30
0.35
0.40
SNP effect on GTCSA
MR Method
IVW
MR Egger
Weighted Median
Inverse of SE (outcome)
150
200
250
300

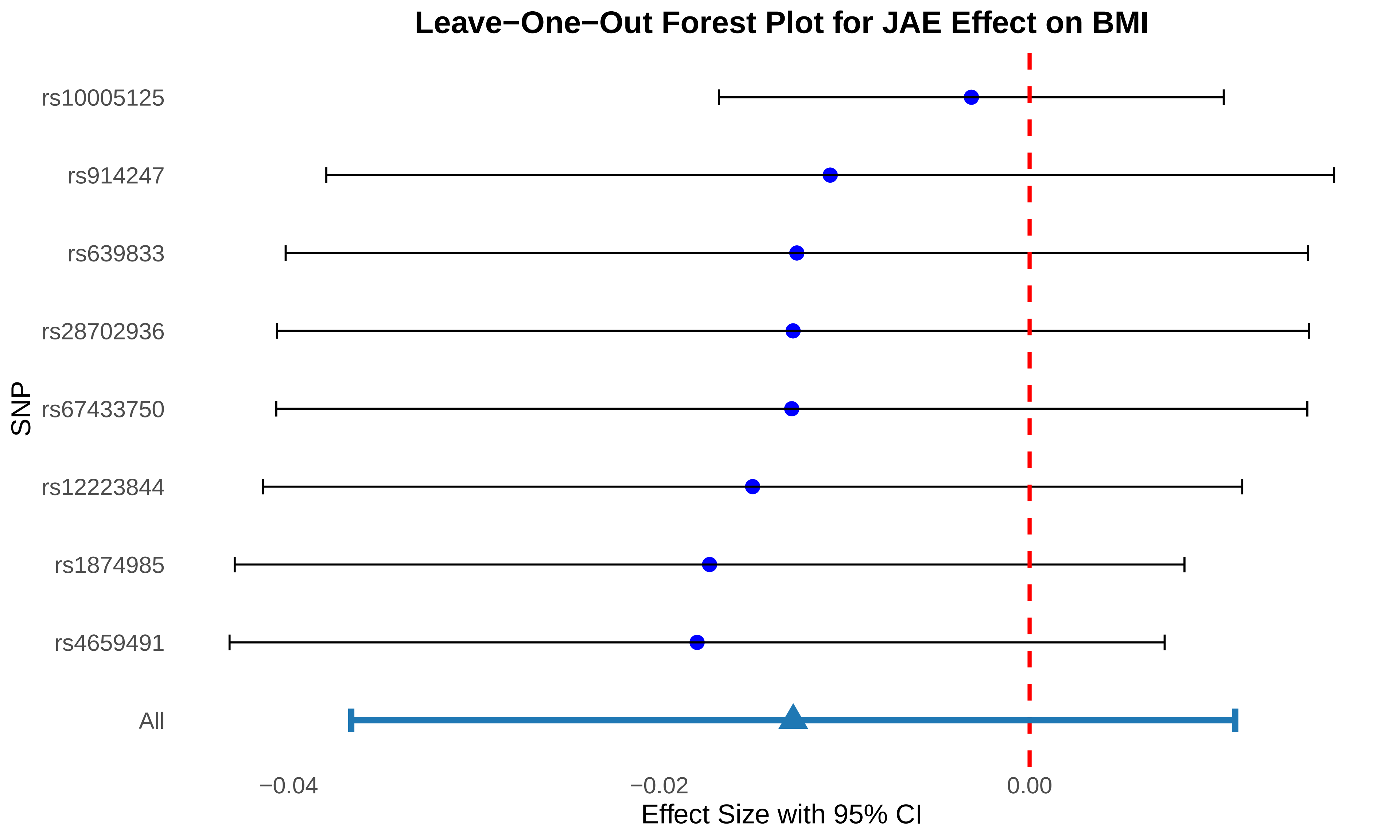

Leave−One−Out Forest Plot for JAE Effect on BMI
SNP
rs10005125
rs914247
rs639833
rs28702936
rs67433750
rs12223844
rs1874985
rs4659491
All
−0.04
−0.02
0.00
Effect Size with 95% CI

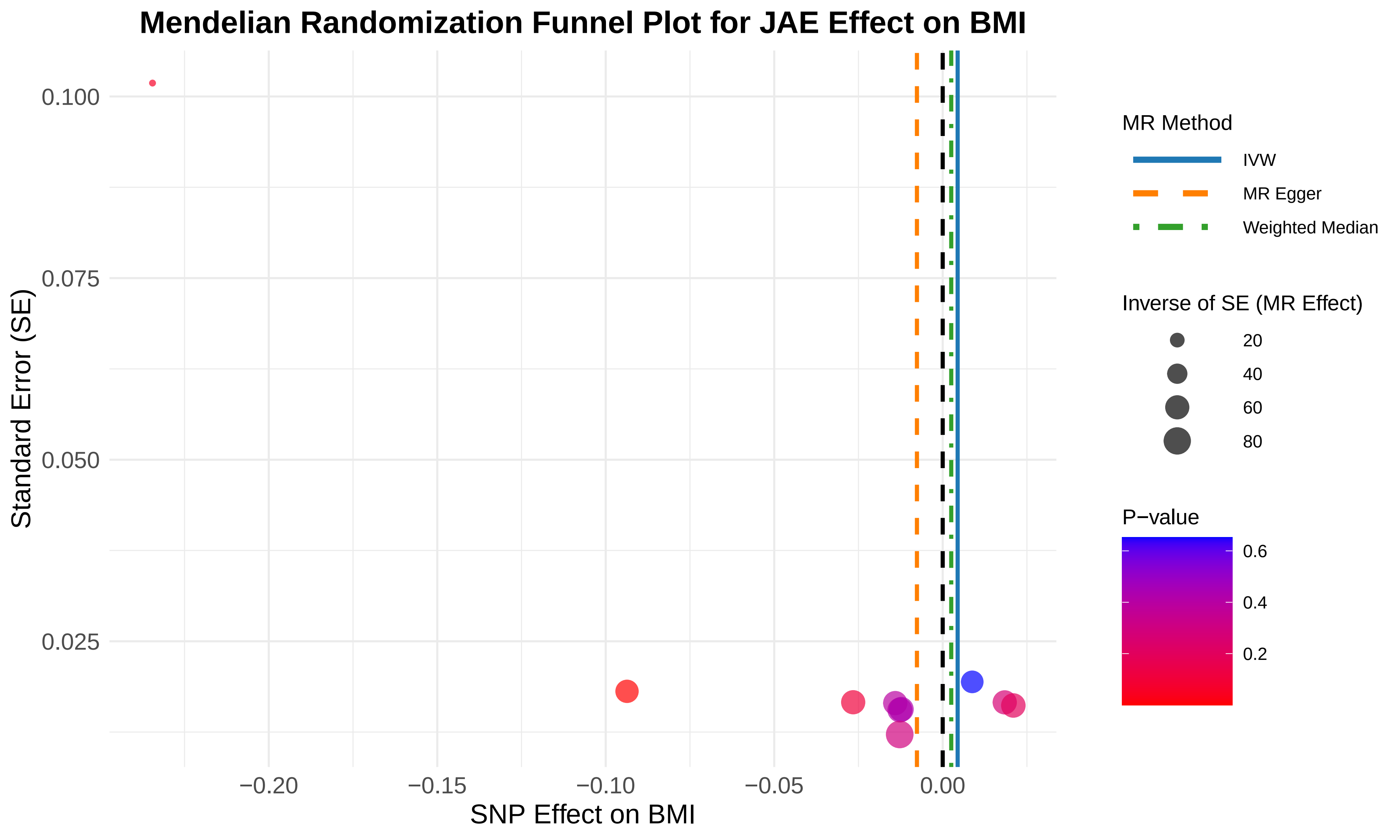

Mendelian Randomization Funnel Plot for JAE Effect on BMI
Standard Error (SE)
0.100
0.075
0.050
0.025
−0.20
−0.15
−0.10
−0.05
0.00
SNP Effect on BMI
MR Method
IVW
MR Egger
Weighted Median
Inverse of SE (MR Effect)
20
40
60
80
P−value
0.6
0.4
0.2

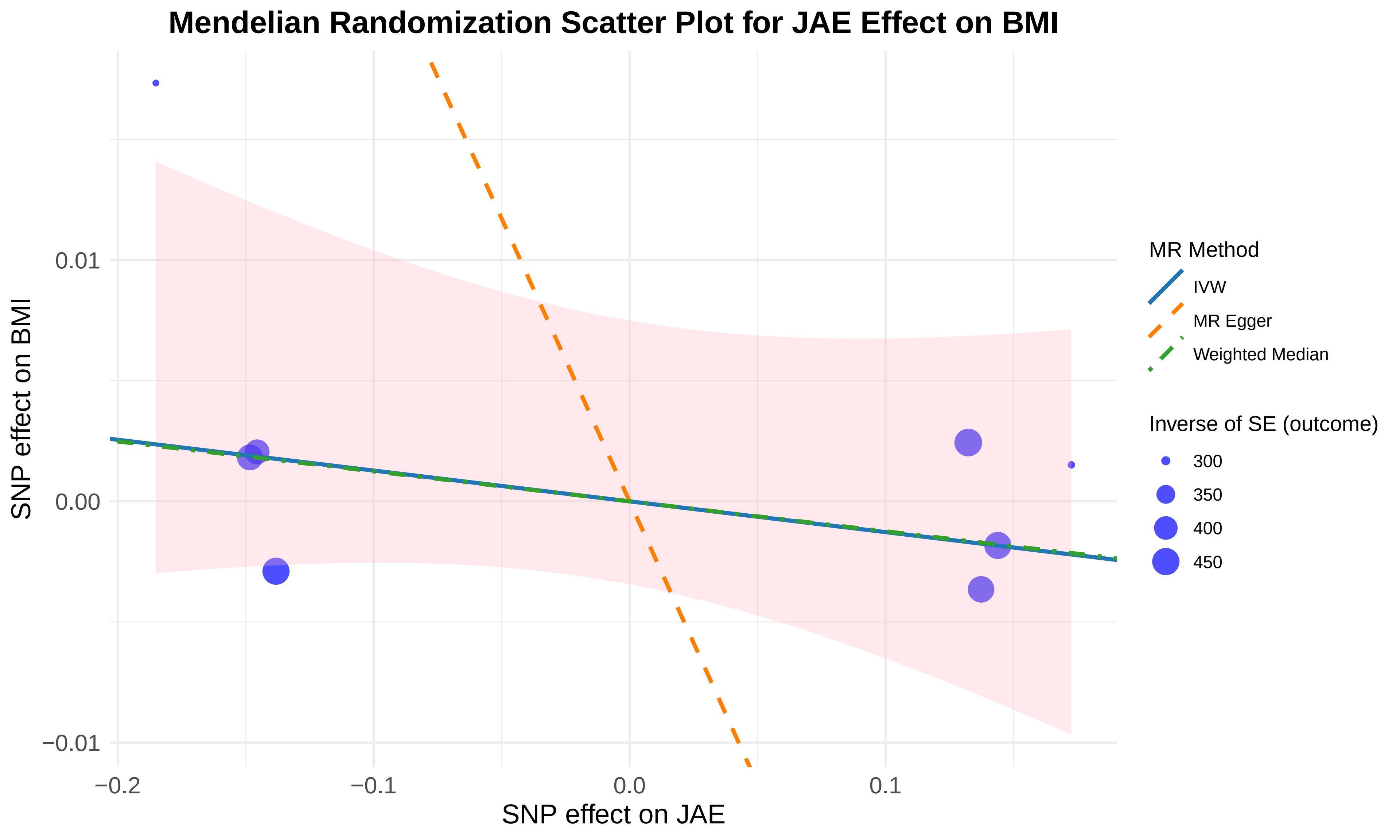
Mendelian Randomization Scatter Plot for JAE Effect on BMI
SNP effect on BMI
SNP effect on JAE
0.01
0.00
−0.01
−0.2
−0.1
0.0
0.1
MR Method
IVW
MR Egger
Weighted Median
Inverse of SE (outcome)
300
350
400
450

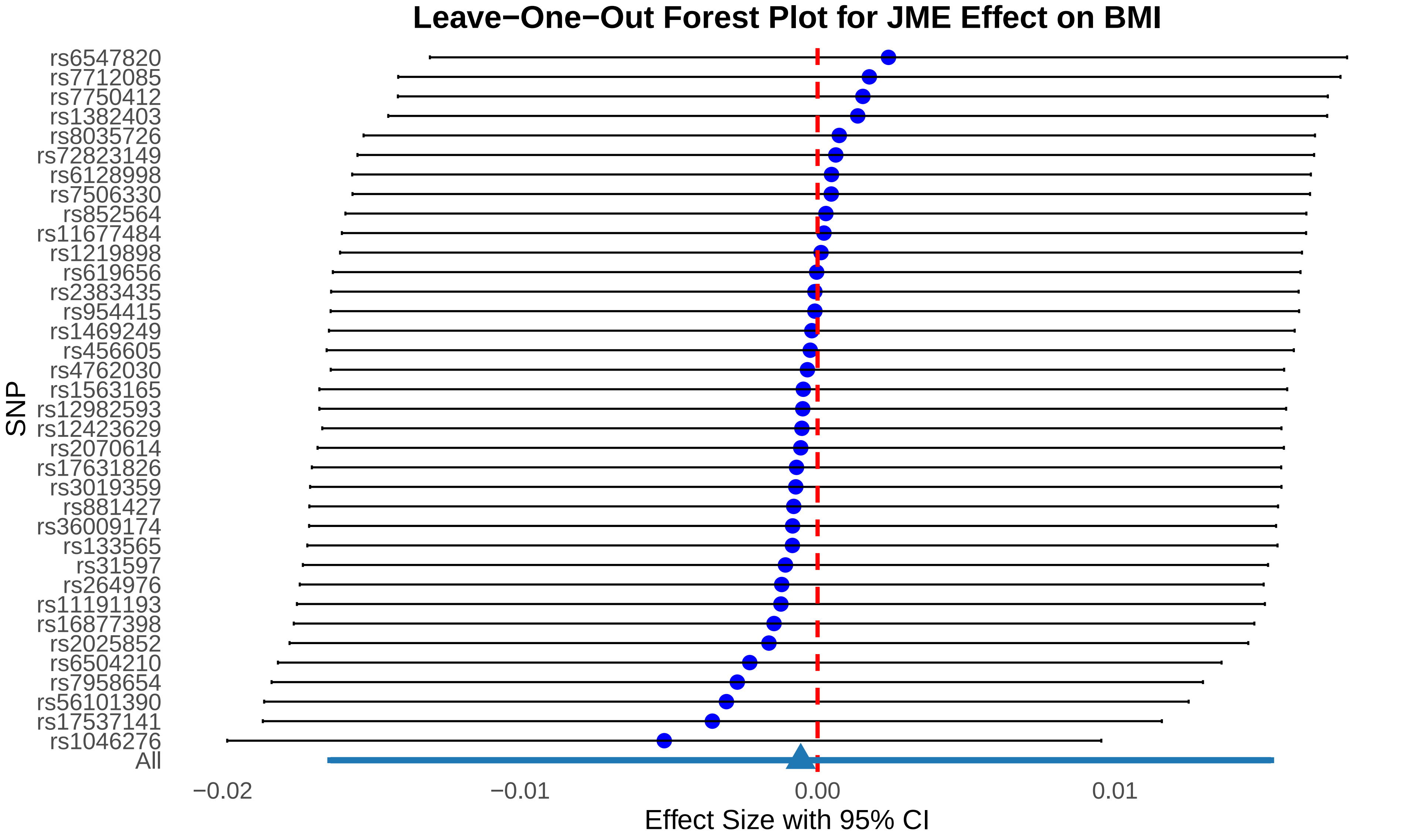
Leave−One−Out Forest Plot for JME Effect on BMI
rs6547820
rs7712085
rs7750412
rs1382403
rs8035726
rs72823149
rs6128998
rs7506330
rs852564
rs11677484
rs1219898
rs619656
rs2383435
rs954415
rs1469249
rs456605
rs4762030
rs1563165
rs12982593
rs12423629
rs2070614
rs17631826
rs3019359
rs881427
rs36009174
rs133565
rs31597
rs264976
rs11191193
rs16877398
rs2025852
rs6504210
rs7958654
rs56101390
rs17537141
rs1046276
All
SNP
−0.02
−0.01
0.00
0.01
Effect Size with 95% CI

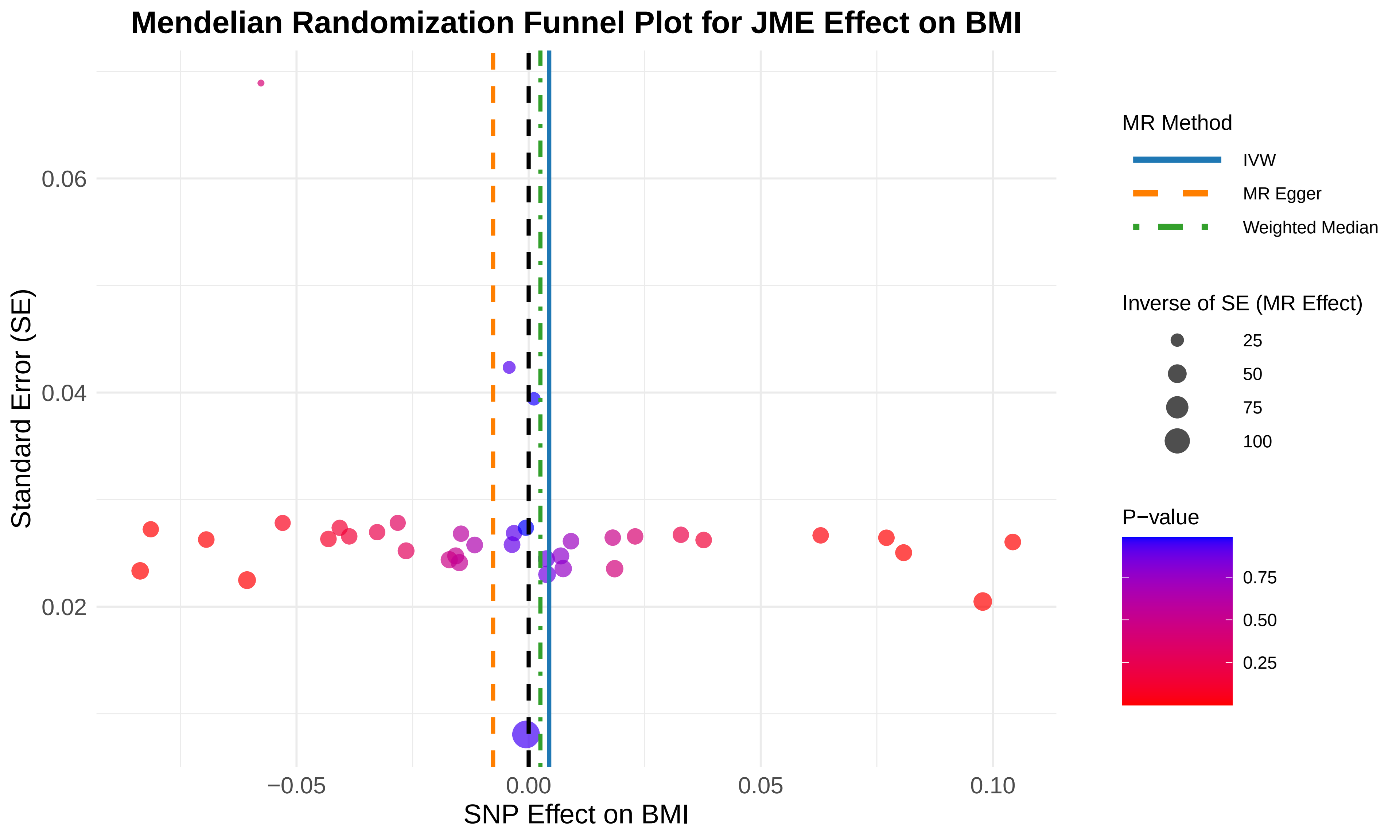

Mendelian Randomization Funnel Plot for JME Effect on BMI
Standard Error (SE)
0.06
0.04
0.02
−0.05
0.00
0.05
0.10
SNP Effect on BMI
MR Method
IVW
MR Egger
Weighted Median
Inverse of SE (MR Effect)
25
50
75
100
P−value
0.75
0.50
0.25

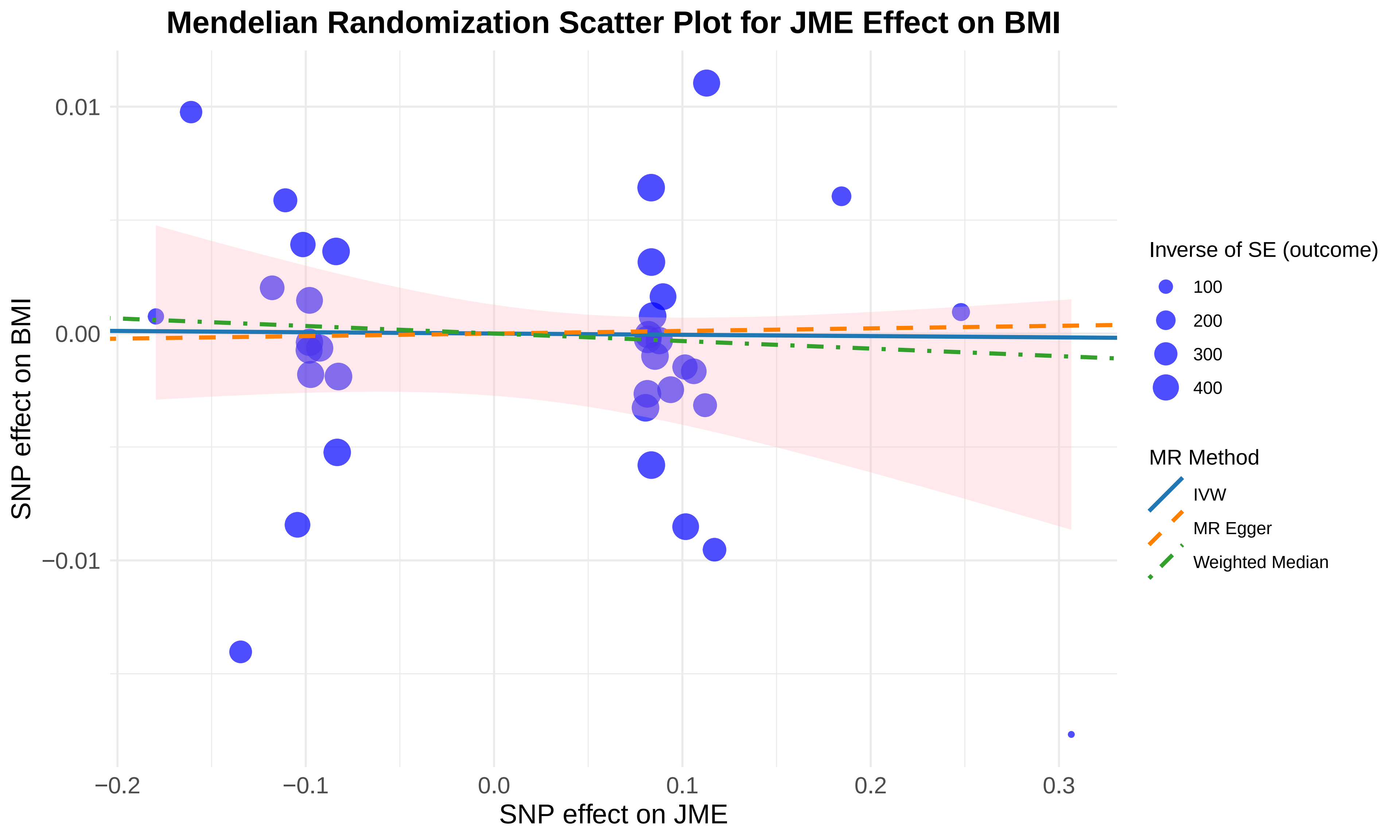
Mendelian Randomization Scatter Plot for JME Effect on BMI
0.01
0.00
−0.01
−0.2
−0.1
0.0
0.1
0.2
0.3
SNP effect on JME
SNP effect on BMI
Inverse of SE (outcome)
100
200
300
400
MR Method
IVW
MR Egger
Weighted Median

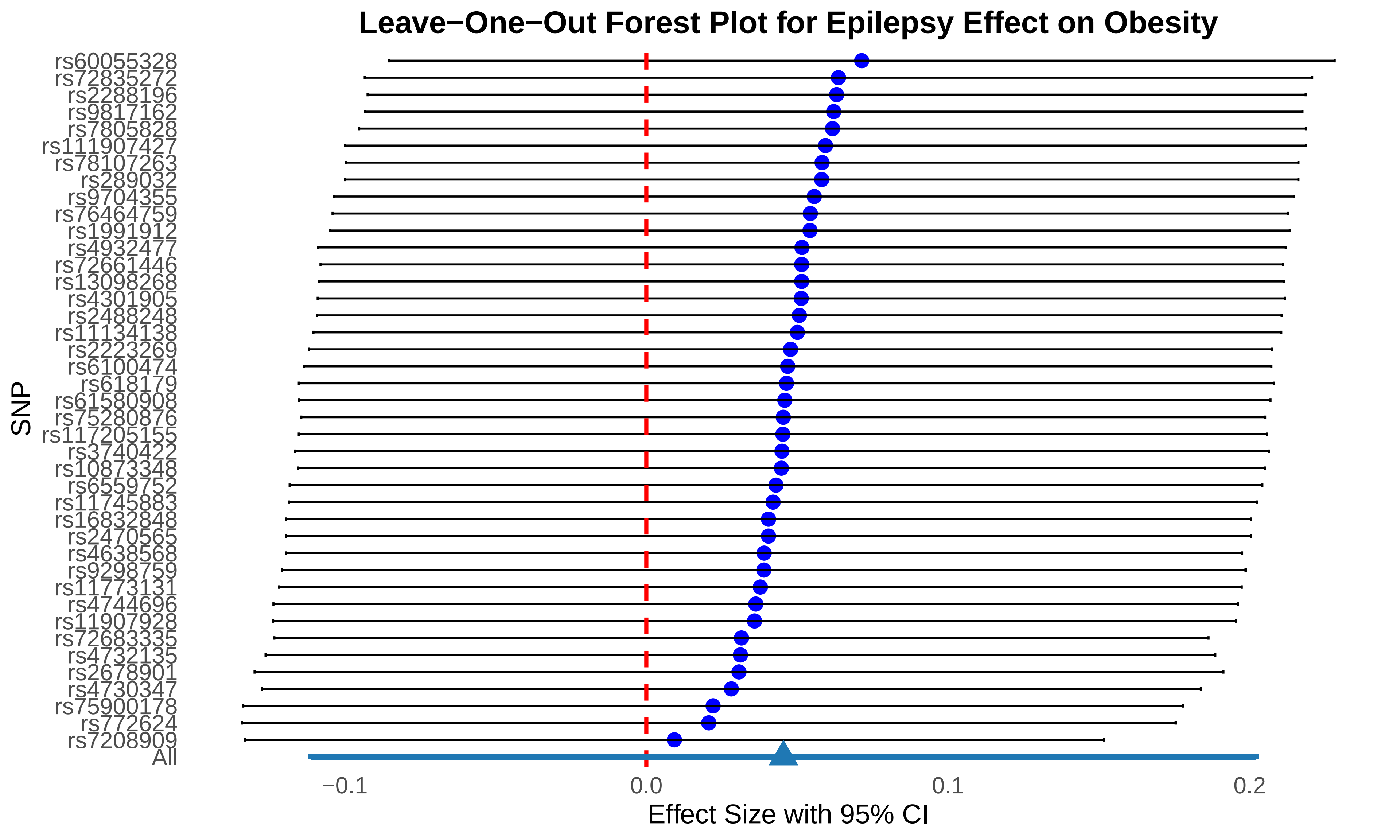
Leave−One−Out Forest Plot for Epilepsy Effect on Obesity
SNP
rs60055328
rs72835272
rs2288196
rs9817162
rs7805828
rs111907427
rs78107263
rs289032
rs9704355
rs76464759
rs1991912
rs4932477
rs72661446
rs13098268
rs4301905
rs2488248
rs11134138
rs2223269
rs6100474
rs618179
rs61580908
rs75280876
rs117205155
rs3740422
rs10873348
rs6559752
rs11745883
rs16832848
rs2470565
rs4638568
rs9298759
rs11773131
rs4744696
rs11907928
rs72683335
rs4732135
rs2678901
rs4730347
rs75900178
rs772624
rs7208909
All
−0.1
0.0
0.1
0.2
Effect Size with 95% CI

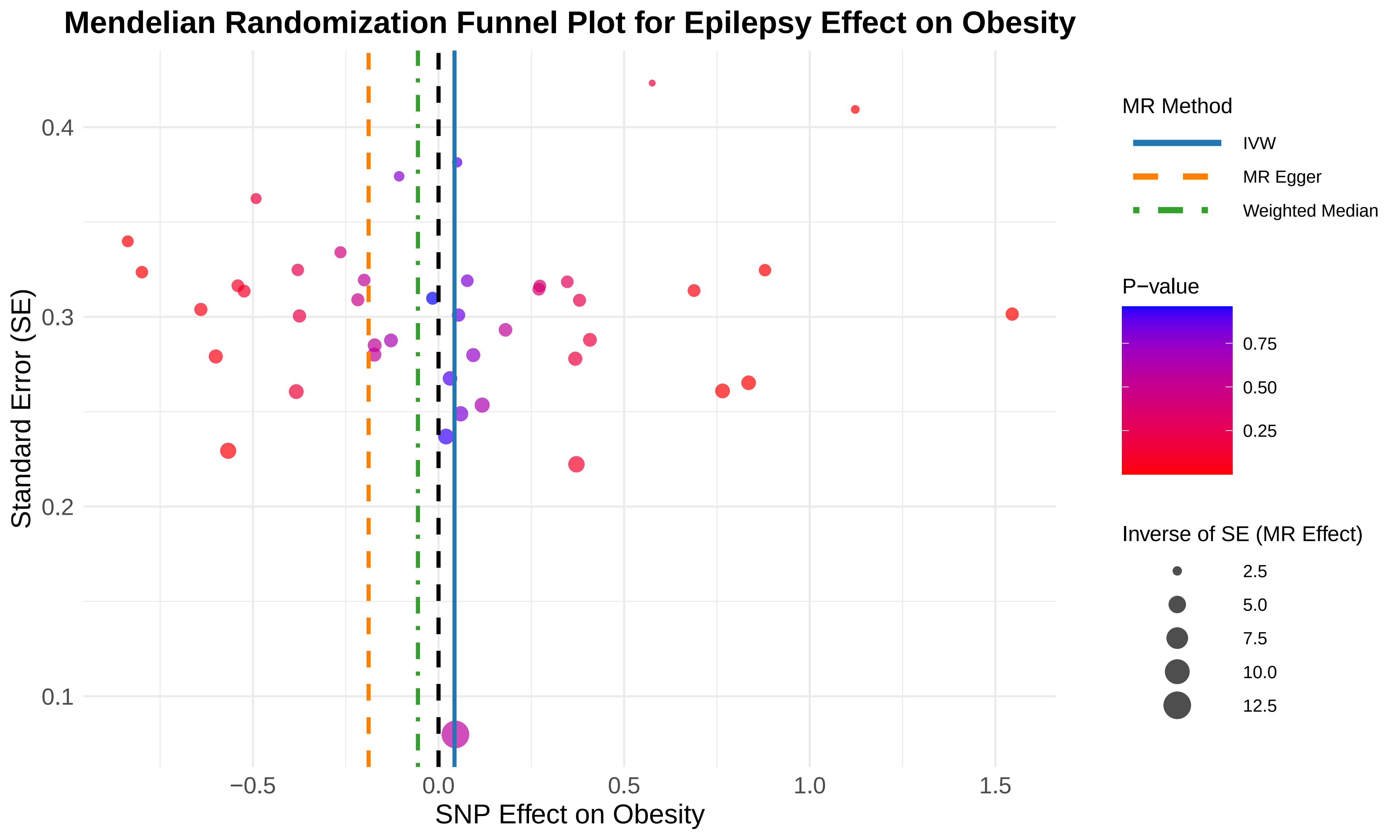
Mendelian Randomization Funnel Plot for Epilepsy Effect on Obesity
Standard Error (SE)
0.4
0.3
0.2
0.1
−0.5
0.0
0.5
1.0
1.5
SNP Effect on Obesity
MR Method
IVW
MR Egger
Weighted Median
P−value
0.75
0.50
0.25
Inverse of SE (MR Effect)
2.5
5.0
7.5
10.0
12.5

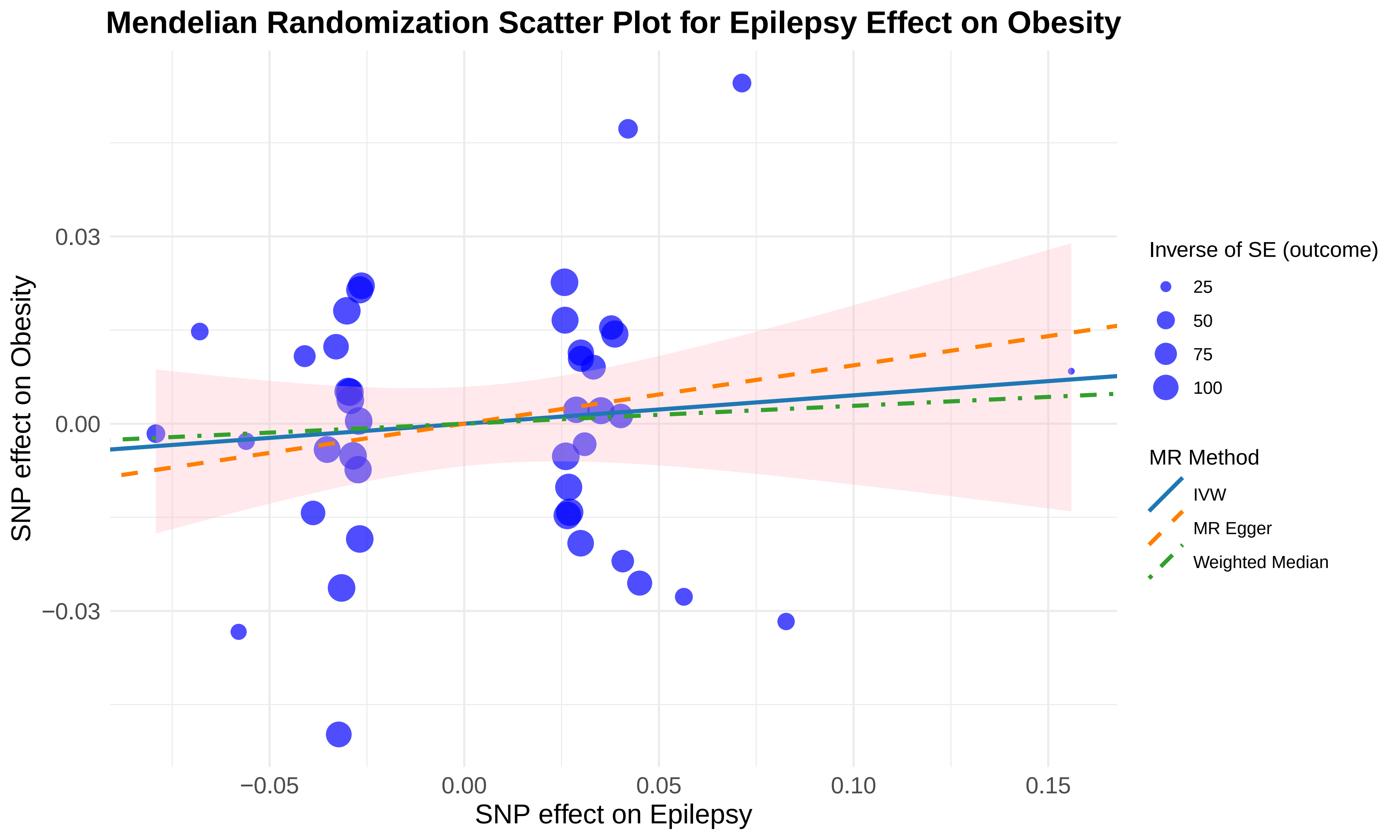

Mendelian Randomization Scatter Plot for Epilepsy Effect on Obesity
SNP effect on Obesity
SNP effect on Epilepsy
0.03
0.00
−0.03
−0.05
0.00
0.05
0.10
0.15
Inverse of SE (outcome)
25
50
75
100
MR Method
IVW
MR Egger
Weighted Median

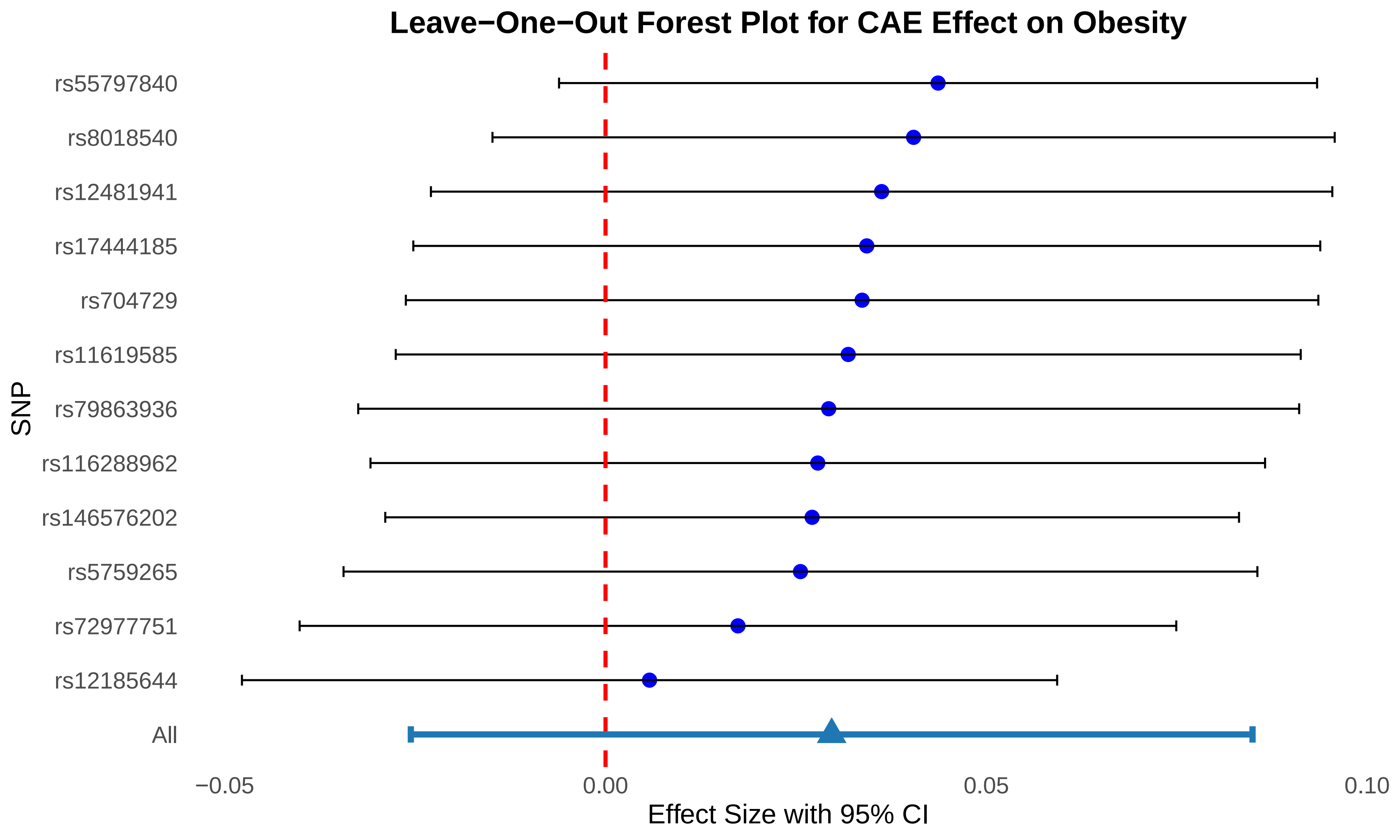

Leave−One−Out Forest Plot for CAE Effect on Obesity
SNP
rs55797840
rs8018540
rs12481941
rs17444185
rs704729
rs11619585
rs79863936
rs116288962
rs146576202
rs5759265
rs72977751
rs12185644
All
−0.05
0.00
0.05
0.10
Effect Size with 95% CI

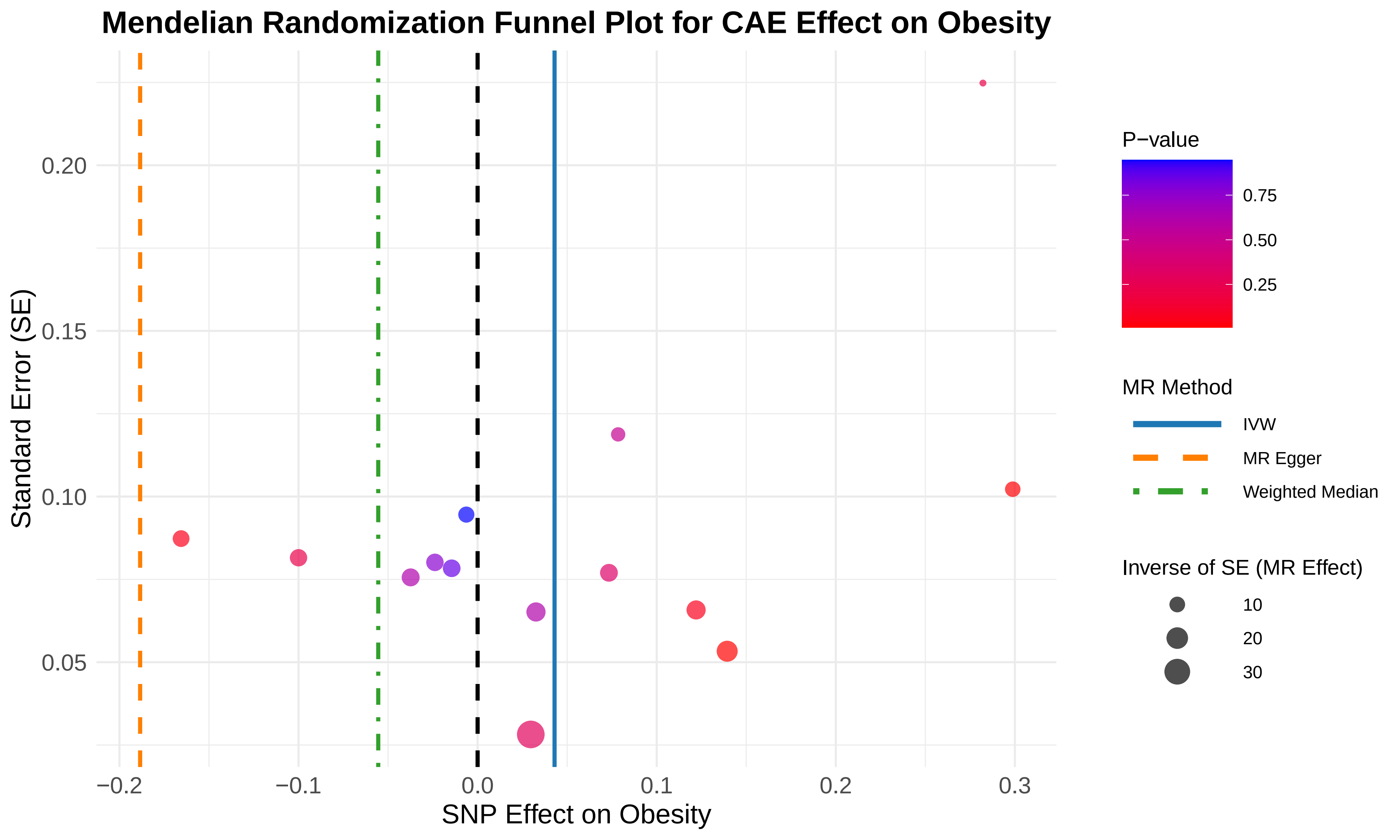
Mendelian Randomization Funnel Plot for CAE Effect on Obesity
Standard Error (SE)
0.20
0.15
0.10
0.05
−0.2
−0.1
0.0
0.1
0.2
0.3
SNP Effect on Obesity
P−value
0.75
0.50
0.25
MR Method
IVW
MR Egger
Weighted Median
Inverse of SE (MR Effect)
10
20
30

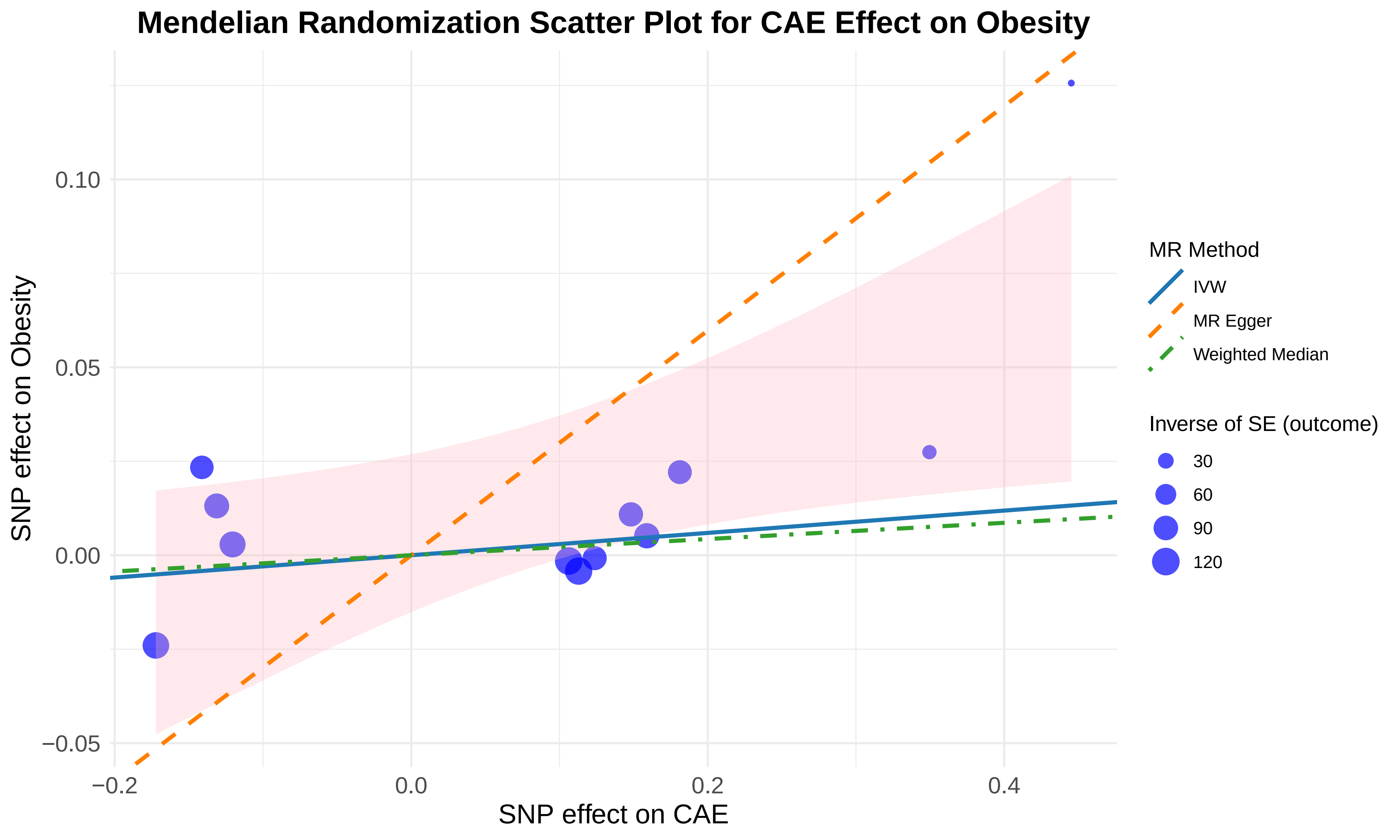
Mendelian Randomization Scatter Plot for CAE Effect on Obesity
SNP effect on Obesity
0.10
0.05
0.00
−0.05
−0.2
0.0
0.2
0.4
SNP effect on CAE
MR Method
IVW
MR Egger
Weighted Median
Inverse of SE (outcome)
30
60
90
120

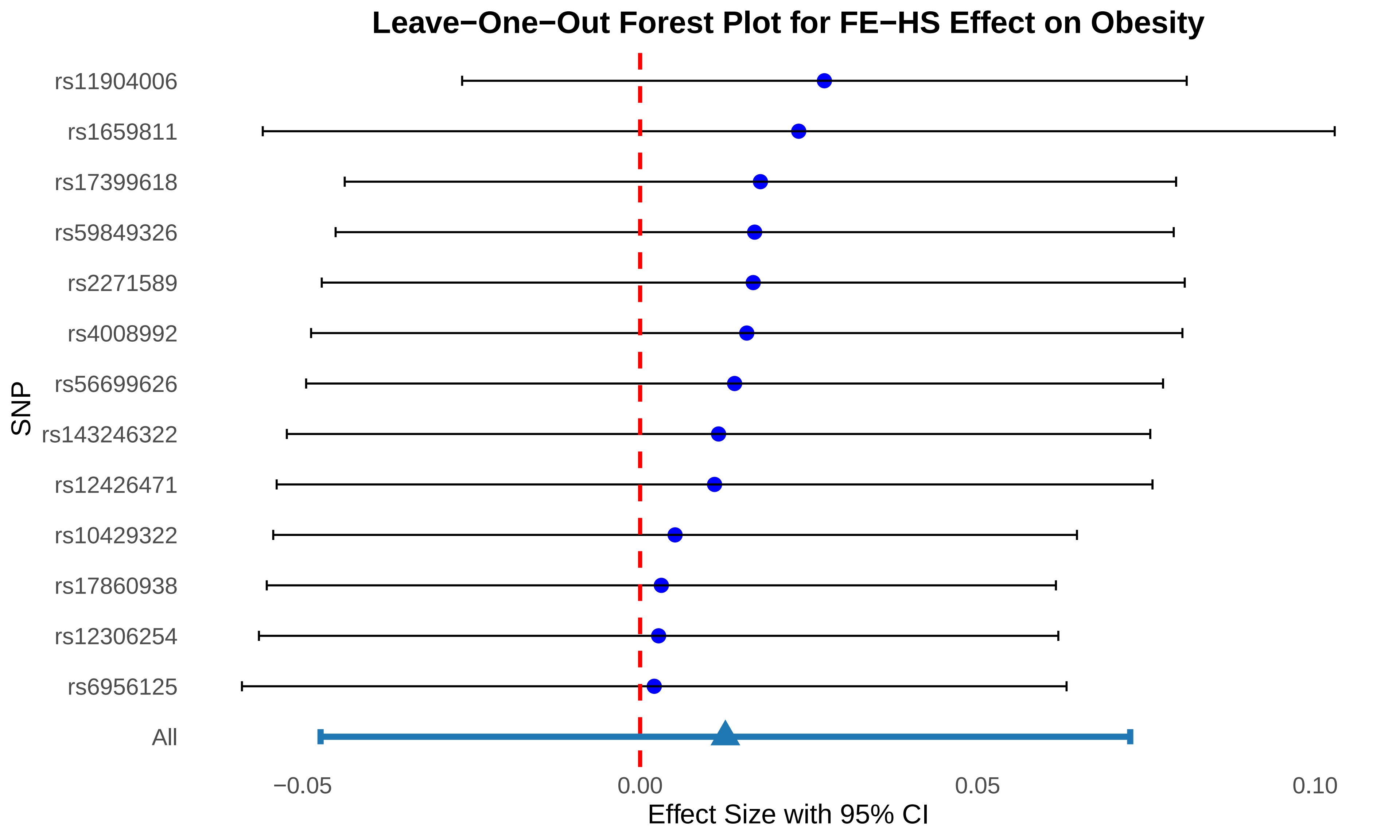

Leave−One−Out Forest Plot for FE−HS Effect on Obesity
rs11904006
rs1659811
rs17399618
rs59849326
rs2271589
rs4008992
rs56699626
rs143246322
rs12426471
rs10429322
rs17860938
rs12306254
rs6956125
All
SNP
−0.05
0.00
0.05
0.10
Effect Size with 95% CI

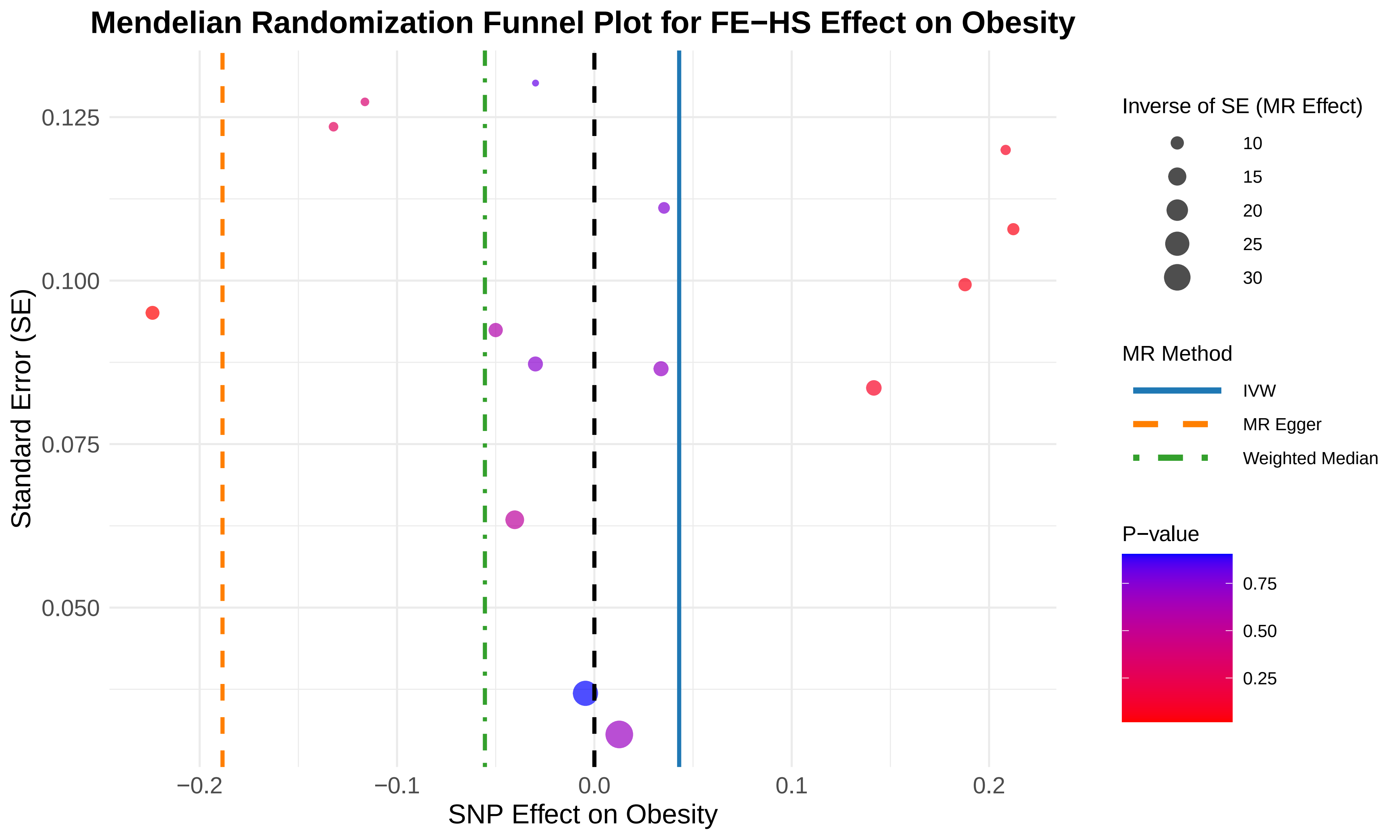
Mendelian Randomization Funnel Plot for FE−HS Effect on Obesity
Standard Error (SE)
0.125
0.100
0.075
0.050
−0.2
−0.1
0.0
0.1
0.2
SNP Effect on Obesity
Inverse of SE (MR Effect)
10
15
20
25
30
MR Method
IVW
MR Egger
Weighted Median
P−value
0.75
0.50
0.25

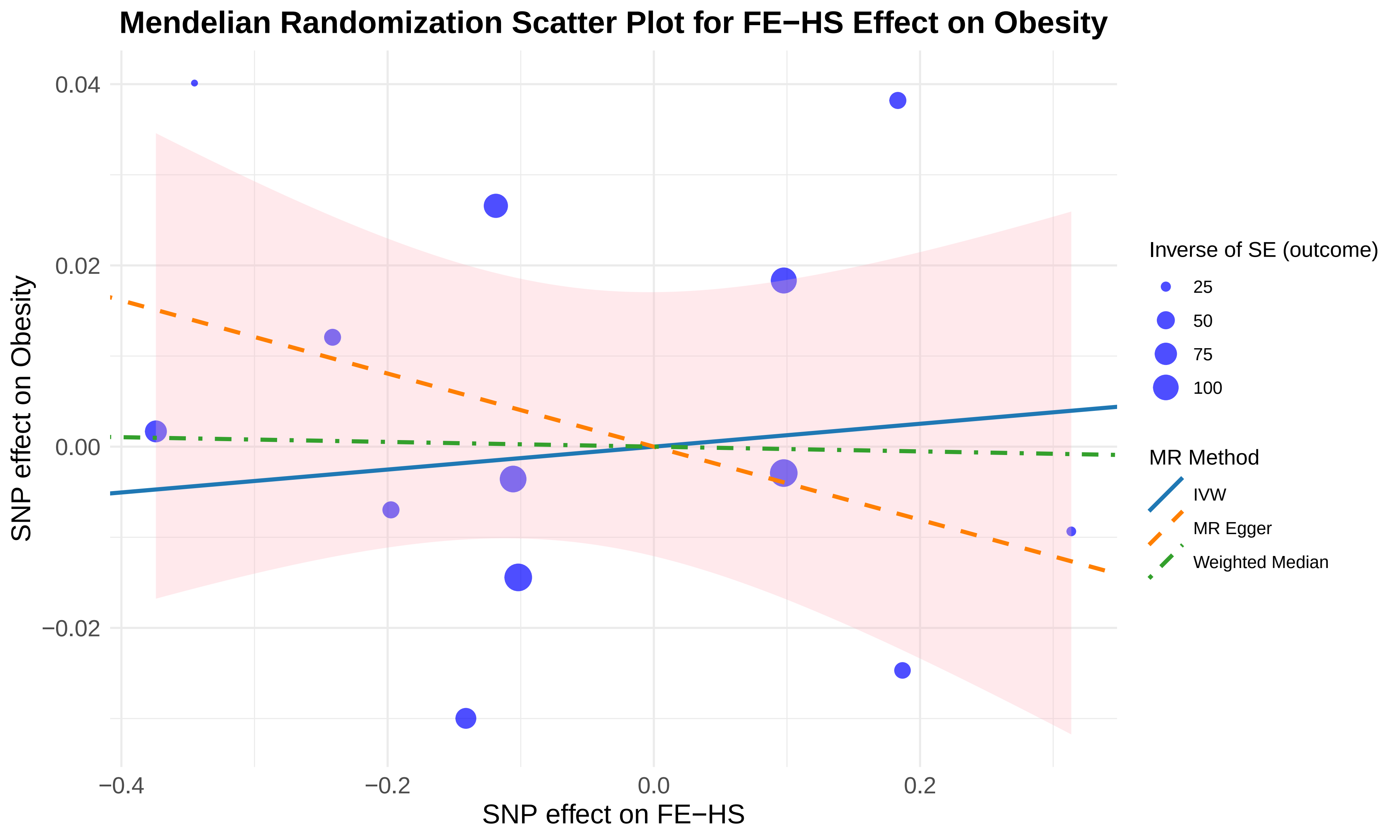
Mendelian Randomization Scatter Plot for FE−HS Effect on Obesity
0.04
0.02
0.00
−0.02
SNP effect on Obesity
−0.4
−0.2
0.0
0.2
SNP effect on FE−HS
Inverse of SE (outcome)
25
50
75
100
MR Method
IVW
MR Egger
Weighted Median

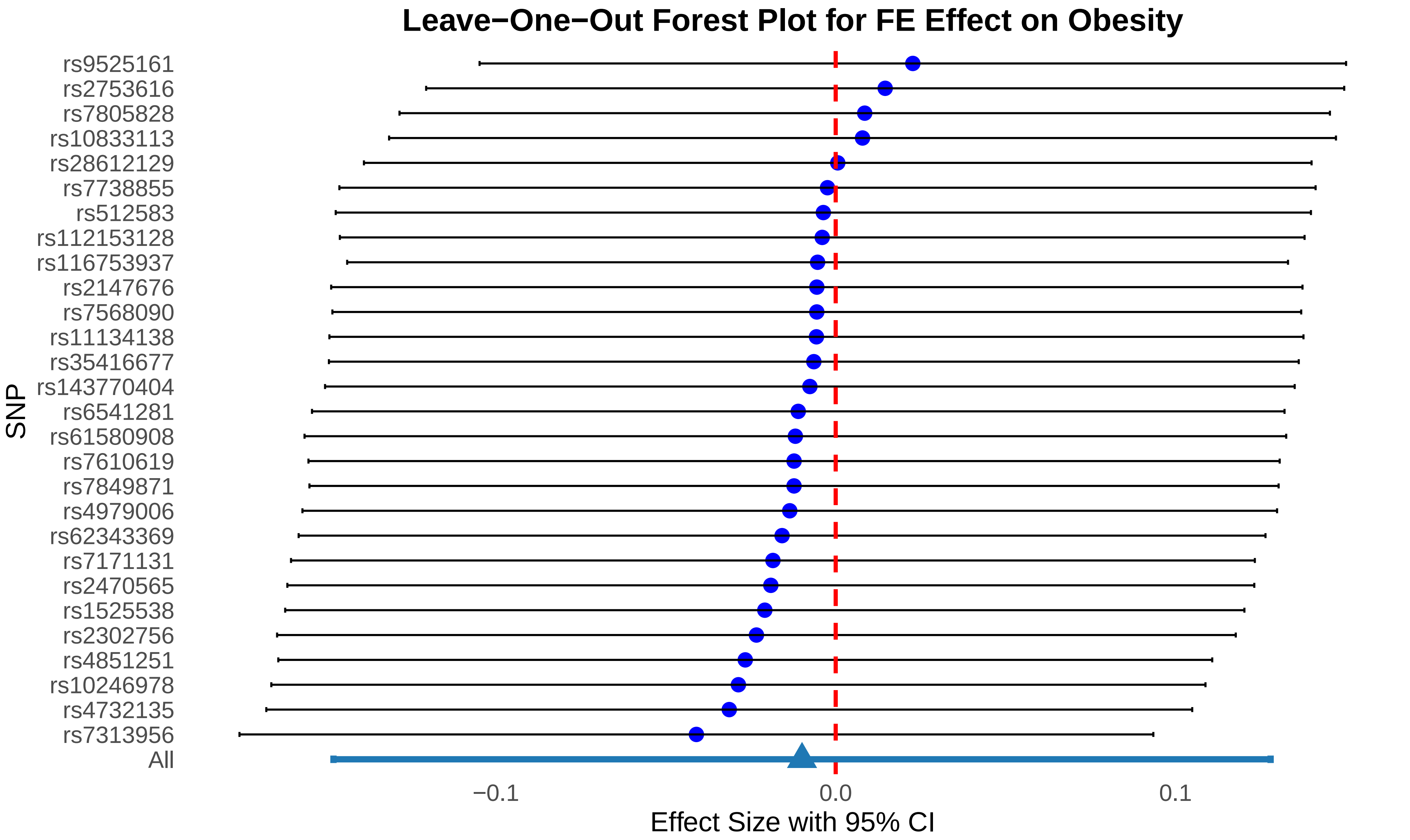
Leave−One−Out Forest Plot for FE Effect on Obesity
rs9525161
rs2753616
rs7805828
rs10833113
rs28612129
rs7738855
rs512583
rs112153128
rs116753937
rs2147676
rs7568090
rs11134138
rs35416677
rs143770404
rs6541281
rs61580908
rs7610619
rs7849871
rs4979006
rs62343369
rs7171131
rs2470565
rs1525538
rs2302756
rs4851251
rs10246978
rs4732135
rs7313956
All
SNP
−0.1
0.0
0.1
Effect Size with 95% CI

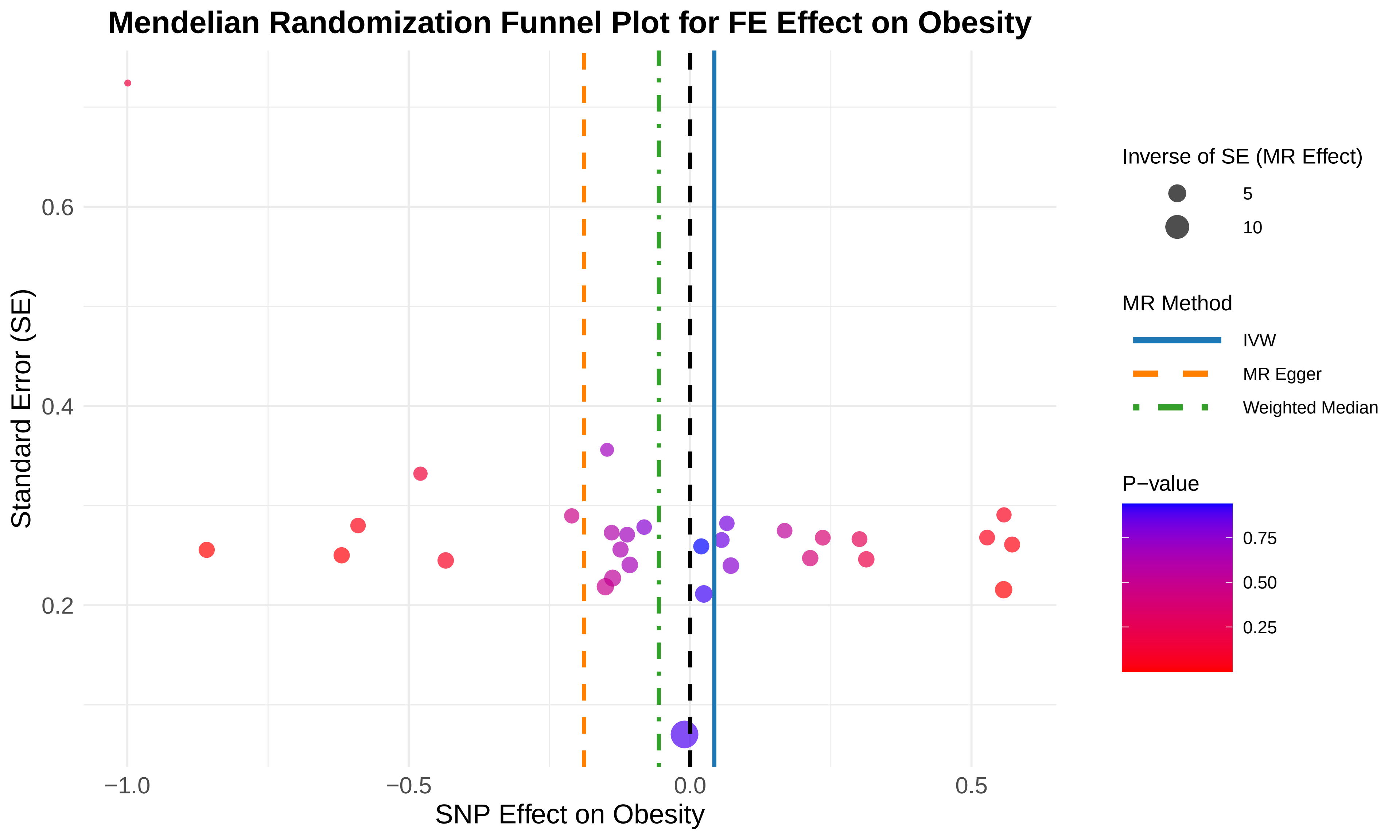
Mendelian Randomization Funnel Plot for FE Effect on Obesity
Standard Error (SE)
0.6
0.4
0.2
−1.0
−0.5
0.0
0.5
SNP Effect on Obesity
Inverse of SE (MR Effect)
5
10
MR Method
IVW
MR Egger
Weighted Median
P−value
0.75
0.50
0.25

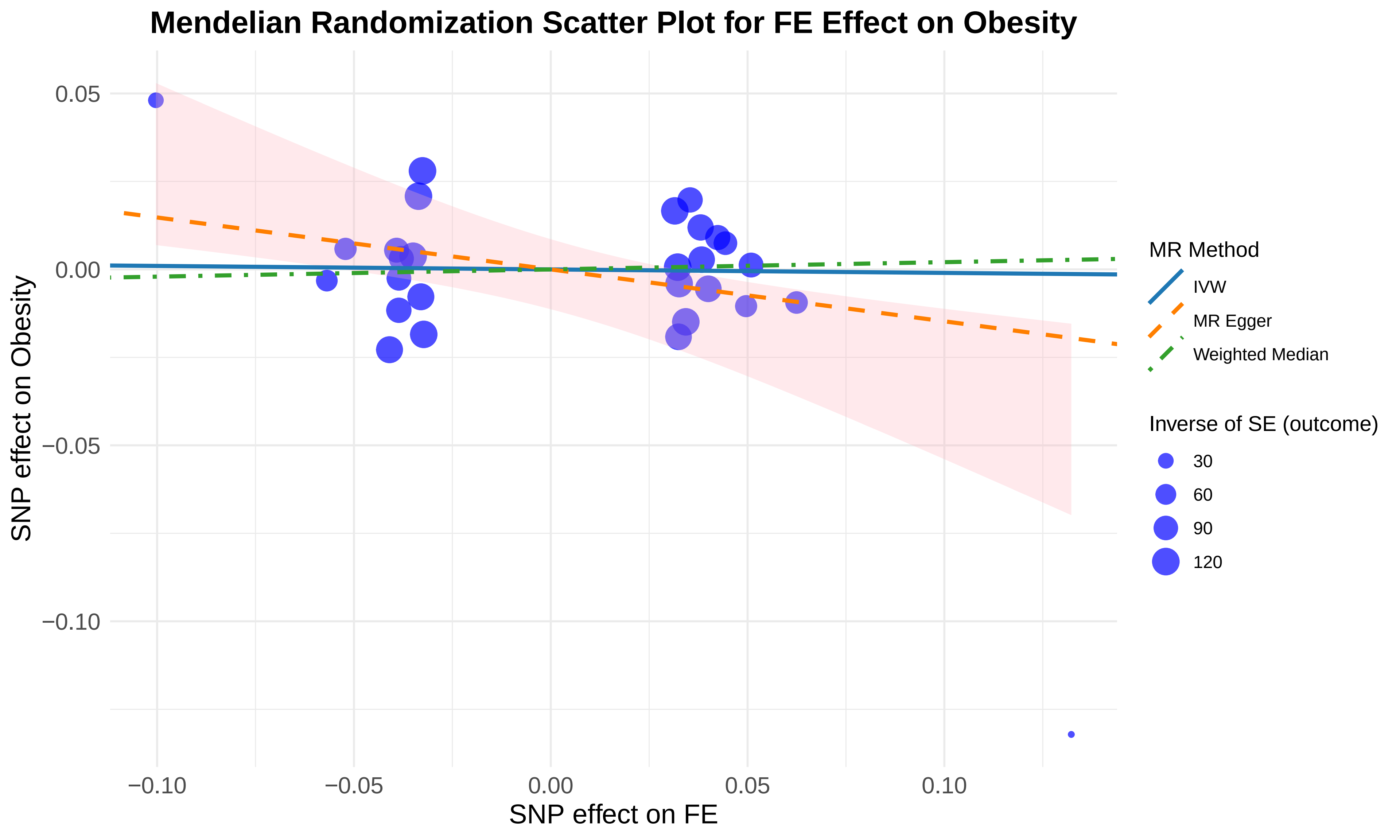

Mendelian Randomization Scatter Plot for FE Effect on Obesity
0.05
0.00
−0.05
−0.10
SNP effect on Obesity
−0.10
−0.05
0.00
0.05
0.10
SNP effect on FE
MR Method
IVW
MR Egger
Weighted Median
Inverse of SE (outcome)
30
60
90
120

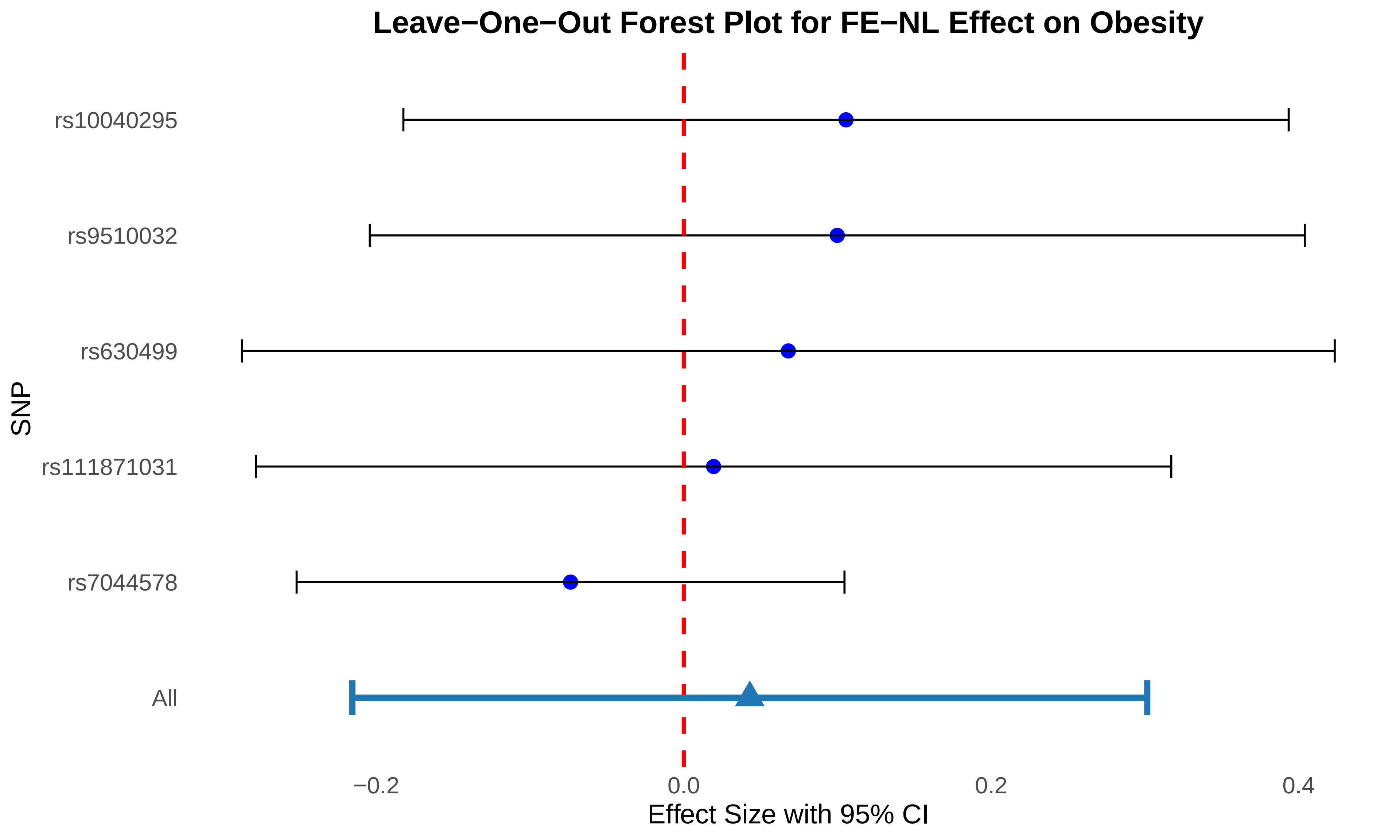

Leave−One−Out Forest Plot for FE−NL Effect on Obesity
rs10040295
rs9510032
rs630499
rs111871031
rs7044578
All
SNP
−0.2
0.0
0.2
0.4
Effect Size with 95% CI

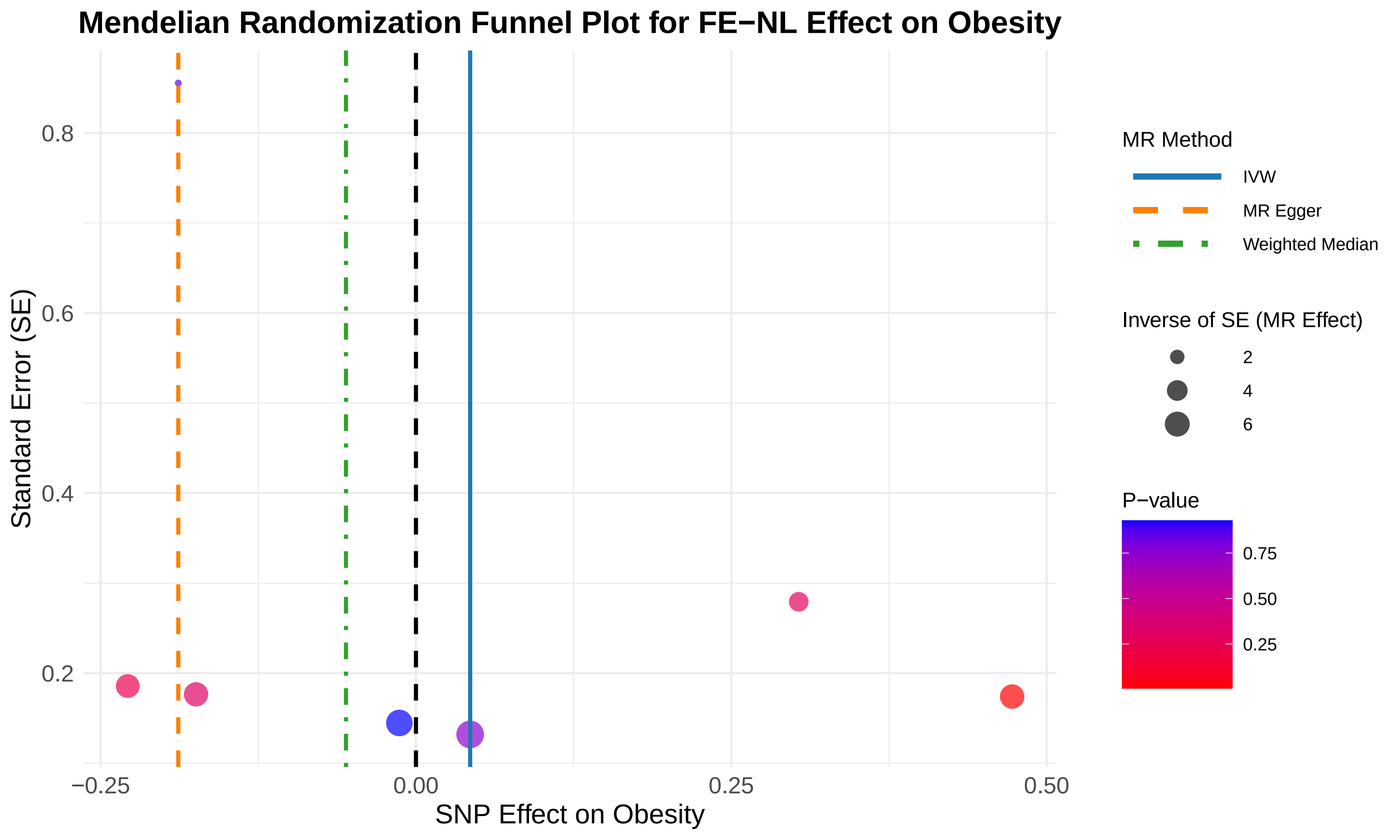
Mendelian Randomization Funnel Plot for FE−NL Effect on Obesity
Standard Error (SE)
0.8
0.6
0.4
0.2
−0.25
0.00
0.25
0.50
SNP Effect on Obesity
MR Method
IVW
MR Egger
Weighted Median
Inverse of SE (MR Effect)
2
4
6
P−value
0.75
0.50
0.25

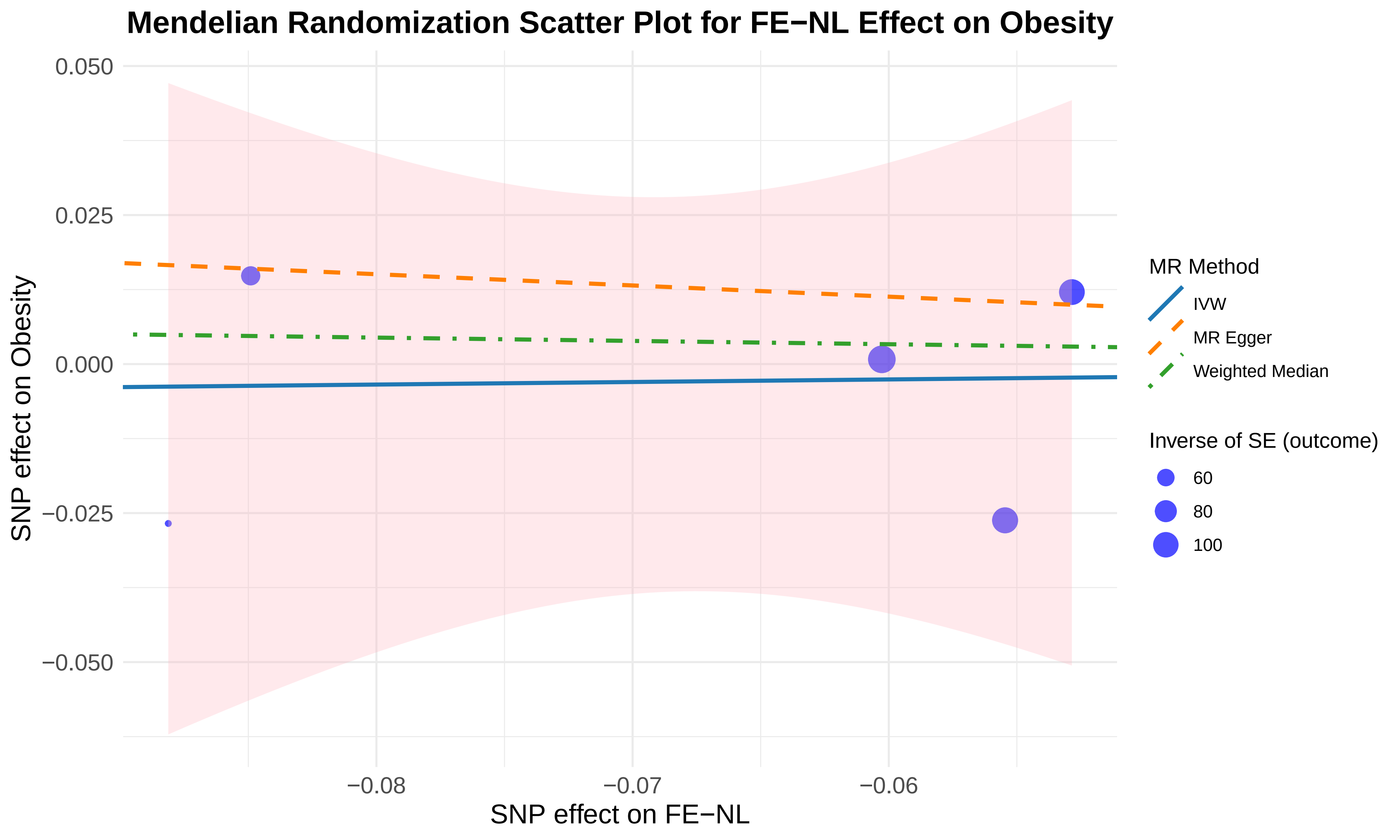
Mendelian Randomization Scatter Plot for FE−NL Effect on Obesity
0.050
0.025
0.000
−0.025
−0.050
SNP effect on Obesity
−0.08
−0.07
−0.06
SNP effect on FE−NL
MR Method
IVW
MR Egger
Weighted Median
Inverse of SE (outcome)
60
80
100

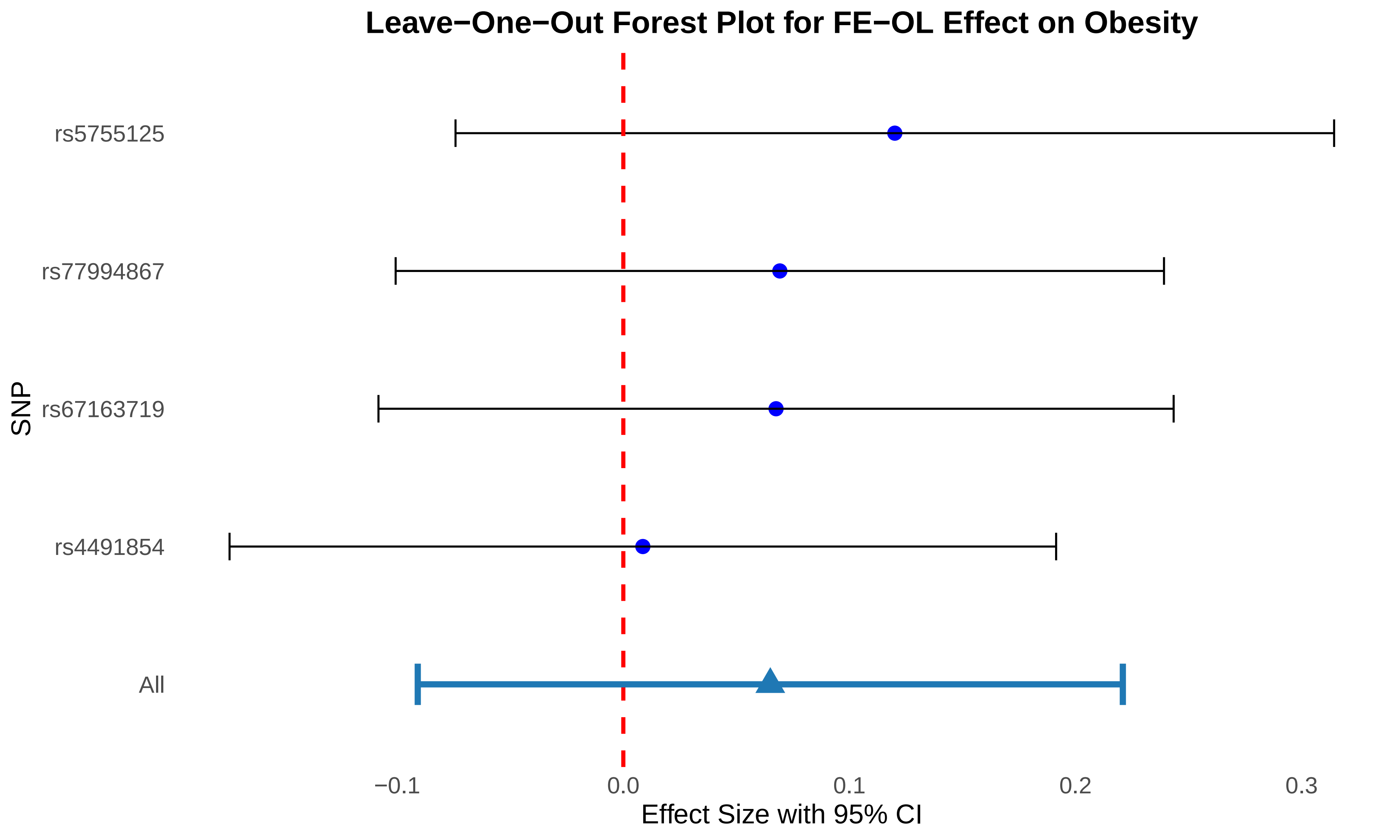

Leave−One−Out Forest Plot for FE−OL Effect on Obesity
SNP
rs5755125
rs77994867
rs67163719
rs4491854
All
−0.1
0.0
0.1
0.2
0.3
Effect Size with 95% CI

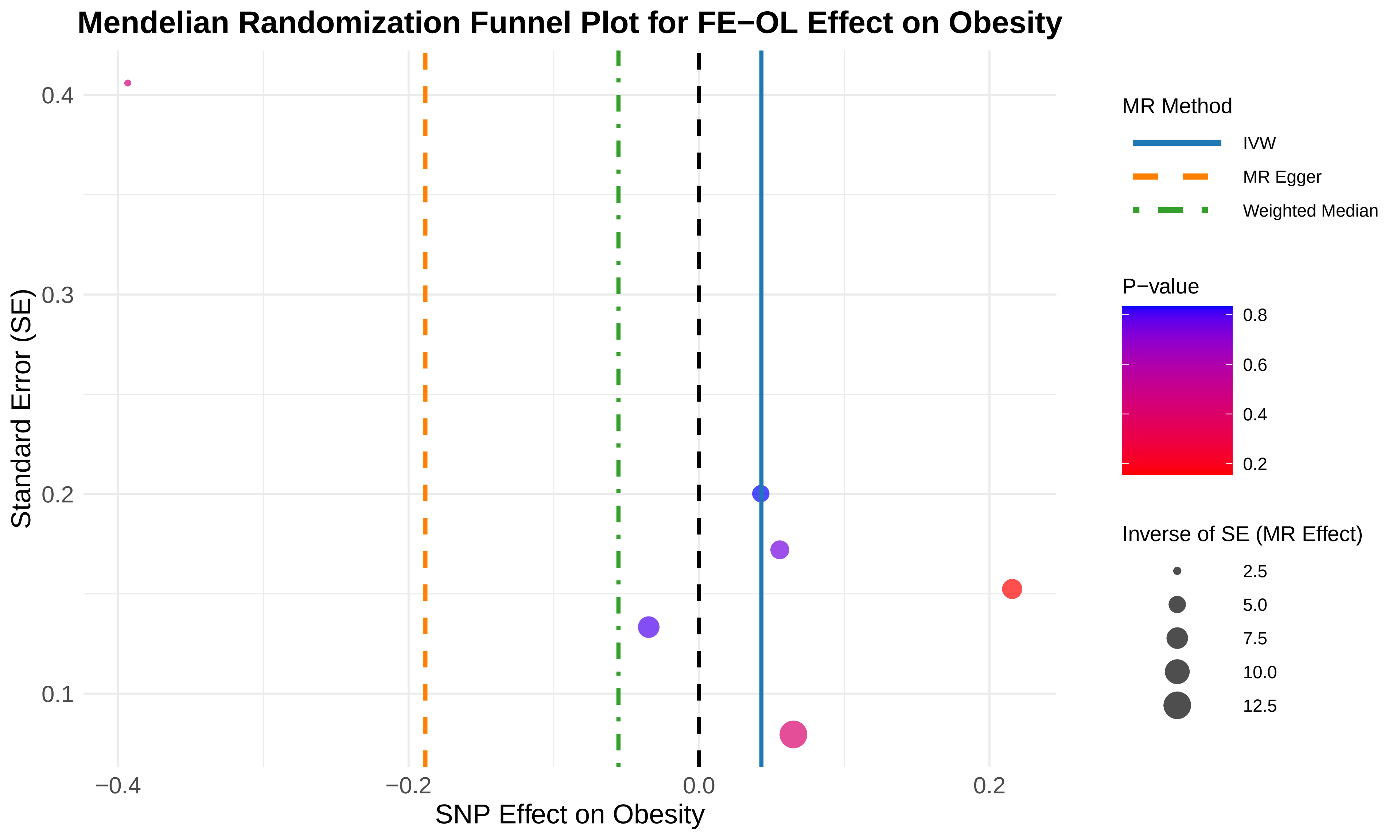
Mendelian Randomization Funnel Plot for FE−OL Effect on Obesity
Standard Error (SE)
0.4
0.3
0.2
0.1
−0.4
−0.2
0.0
0.2
SNP Effect on Obesity
MR Method
IVW
MR Egger
Weighted Median
P−value
0.8
0.6
0.4
0.2
Inverse of SE (MR Effect)
2.5
5.0
7.5
10.0
12.5

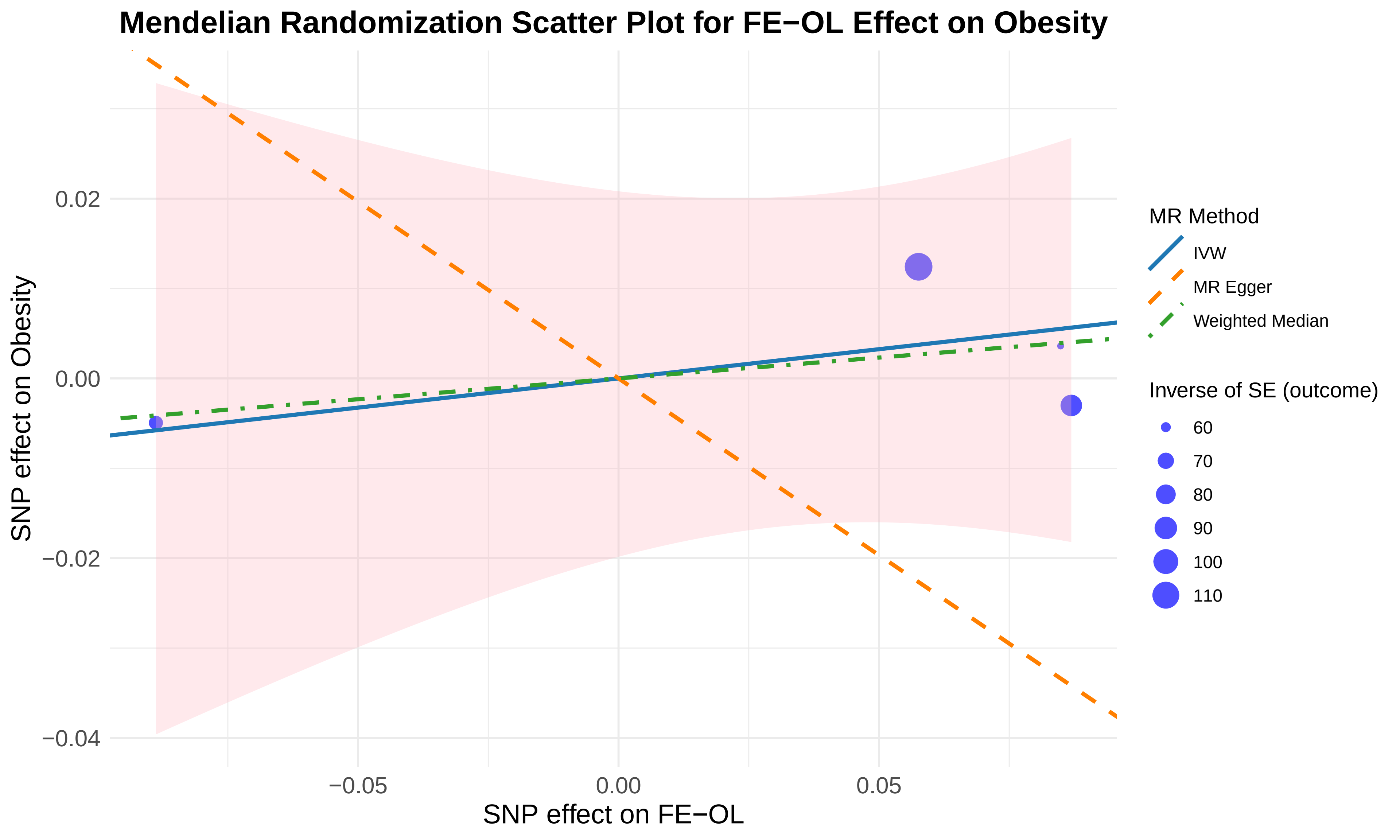

Mendelian Randomization Scatter Plot for FE−OL Effect on Obesity
SNP effect on Obesity
0.02
0.00
−0.02
−0.04
−0.05
0.00
0.05
SNP effect on FE−OL
MR Method
IVW
MR Egger
Weighted Median
Inverse of SE (outcome)
60
70
80
90
100
110

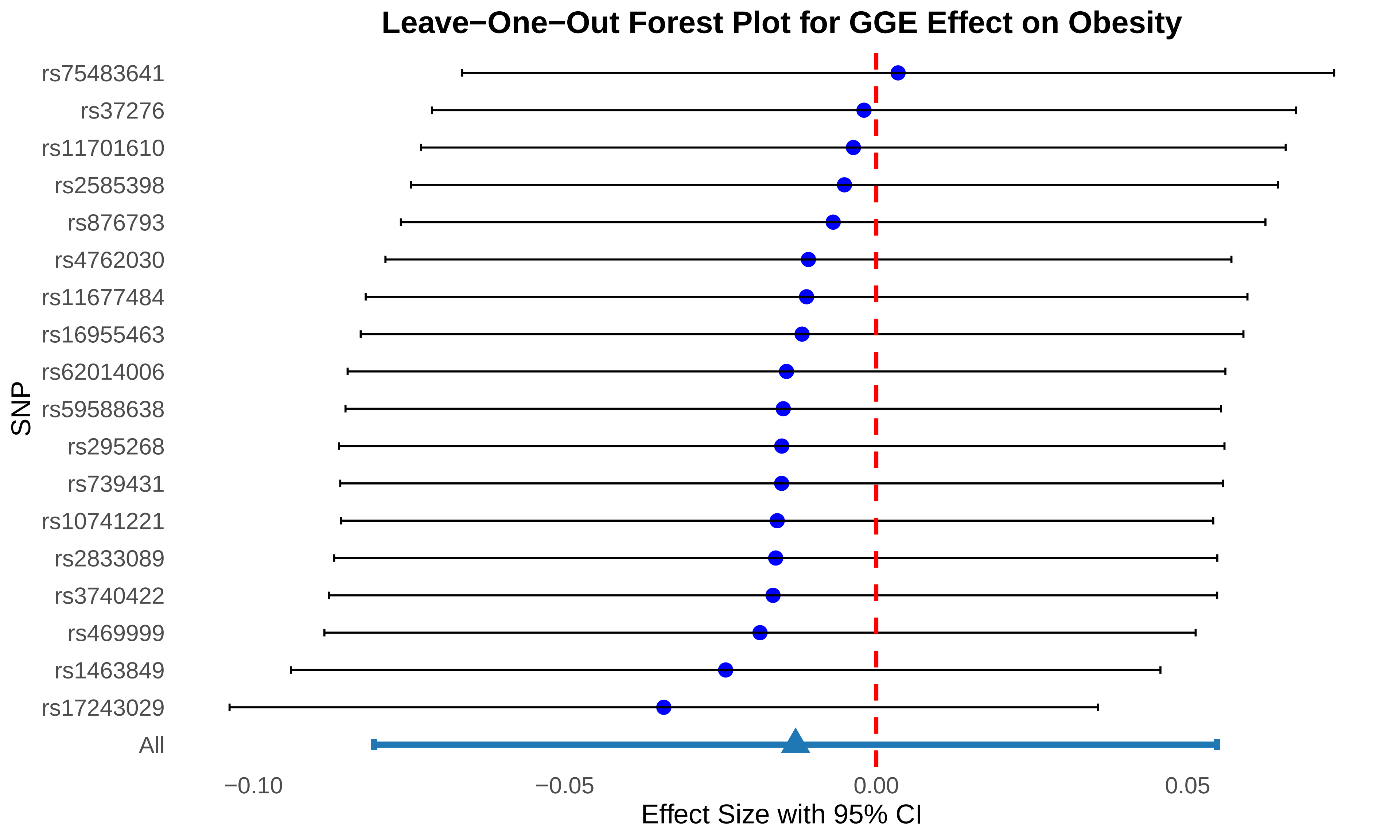

Leave−One−Out Forest Plot for GGE Effect on Obesity
rs75483641
rs37276
rs11701610
rs2585398
rs876793
rs4762030
rs11677484
rs16955463
rs62014006
rs59588638
rs295268
rs739431
rs10741221
rs2833089
rs3740422
rs469999
rs1463849
rs17243029
All
SNP
−0.10
−0.05
0.00
0.05
Effect Size with 95% CI

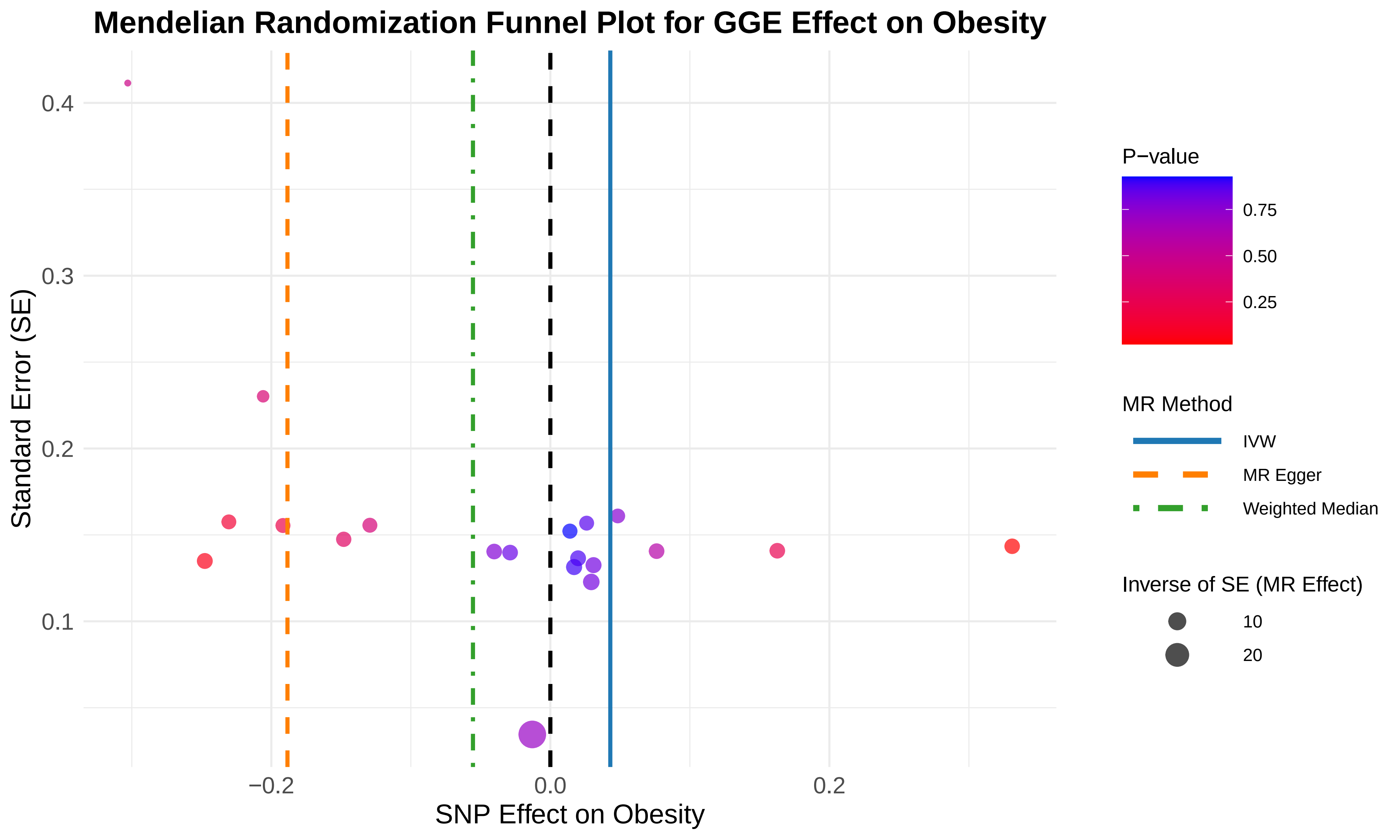
Mendelian Randomization Funnel Plot for GGE Effect on Obesity
Standard Error (SE)
0.4
0.3
0.2
0.1
−0.2
0.0
0.2
SNP Effect on Obesity
P−value
0.75
0.50
0.25
MR Method
IVW
MR Egger
Weighted Median
Inverse of SE (MR Effect)
10
20

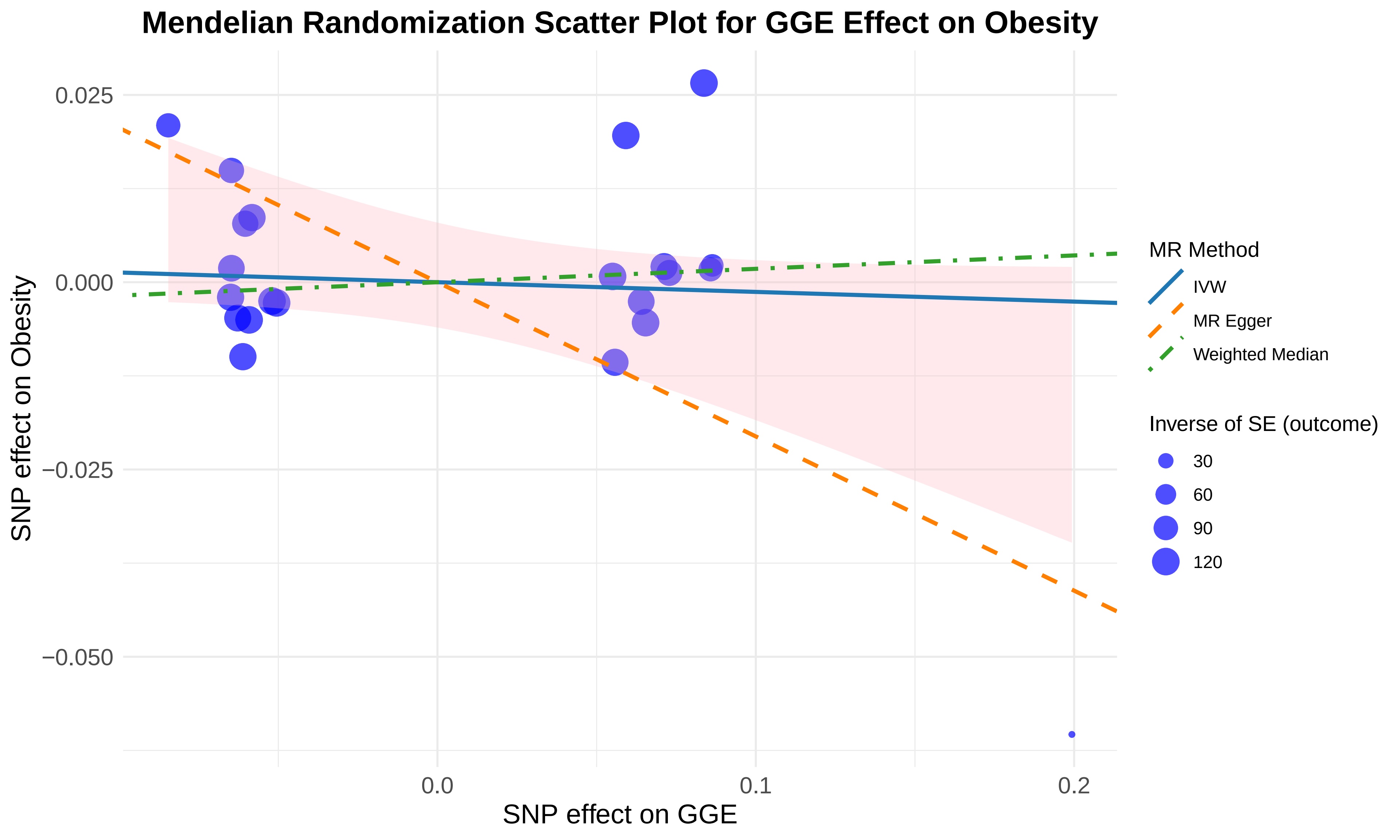

Mendelian Randomization Scatter Plot for GGE Effect on Obesity
0.025
0.000
−0.025
−0.050
SNP effect on Obesity
0.0
0.1
0.2
SNP effect on GGE
MR Method
IVW
MR Egger
Weighted Median
Inverse of SE (outcome)
30
60
90
120

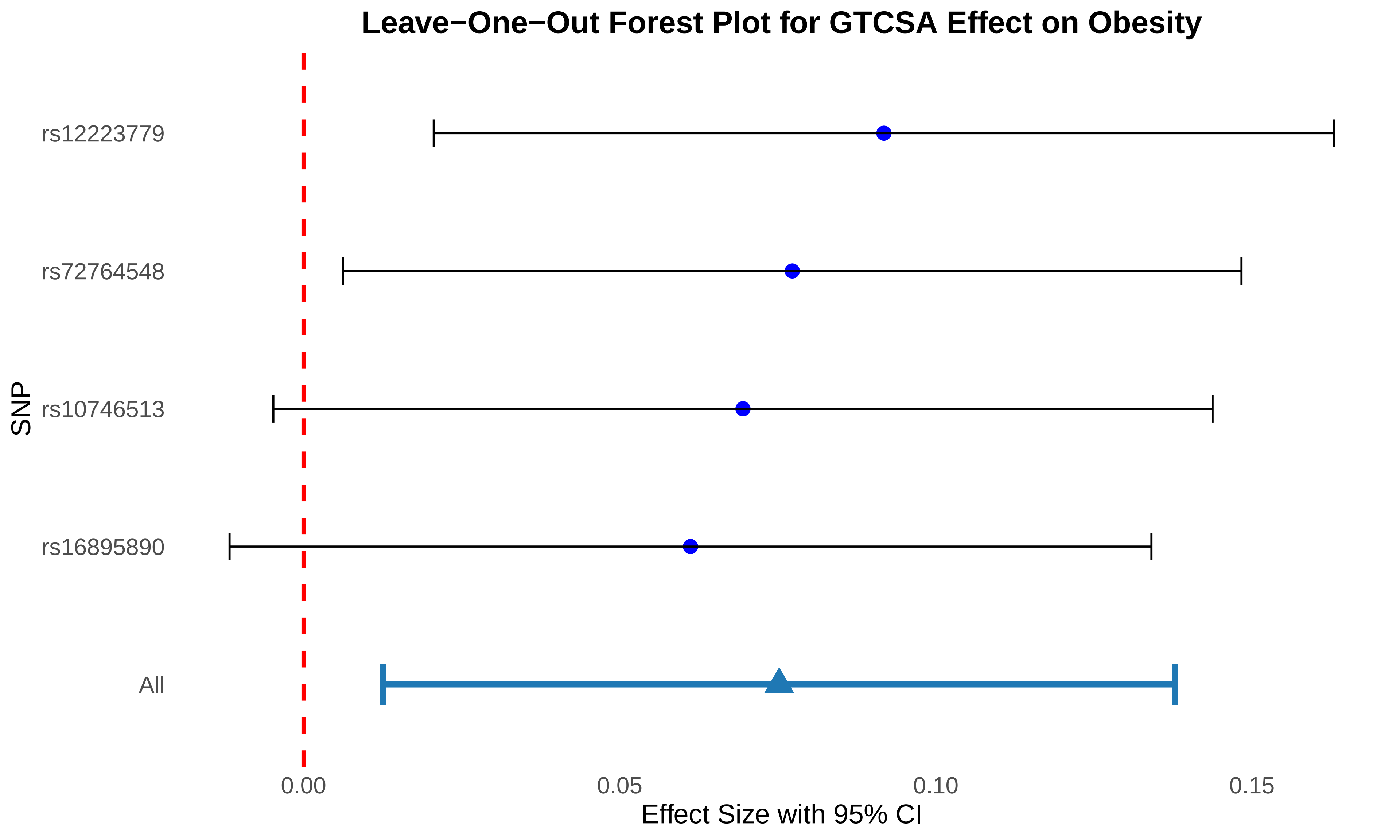
Leave−One−Out Forest Plot for GTCSA Effect on Obesity
SNP
rs12223779
rs72764548
rs10746513
rs16895890
All
0.00
0.05
0.10
0.15
Effect Size with 95% CI

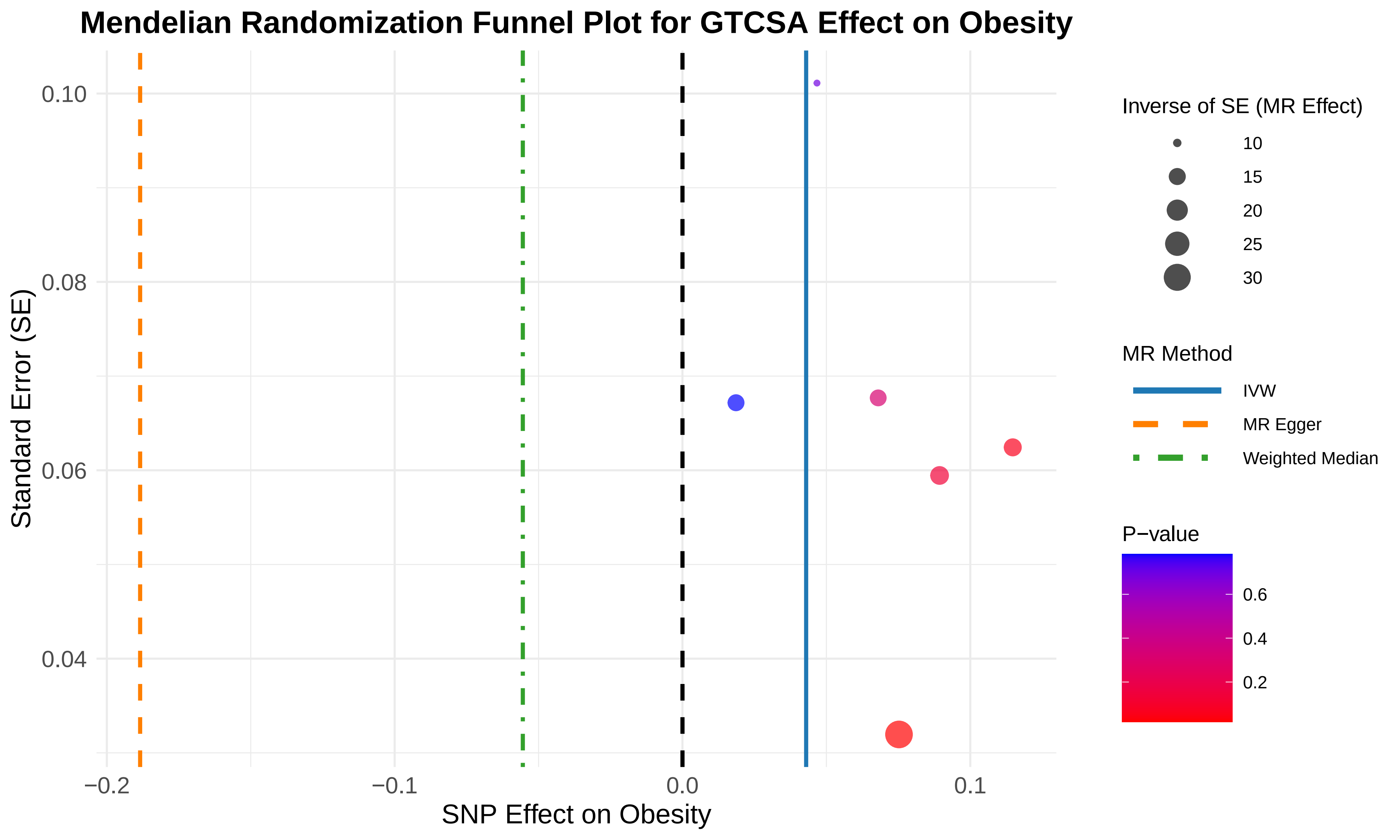
Mendelian Randomization Funnel Plot for GTCSA Effect on Obesity
Standard Error (SE)
0.10
0.08
0.06
0.04
−0.2
−0.1
0.0
0.1
SNP Effect on Obesity
Inverse of SE (MR Effect)
10
15
20
25
30
MR Method
IVW
MR Egger
Weighted Median
P−value
0.6
0.4
0.2

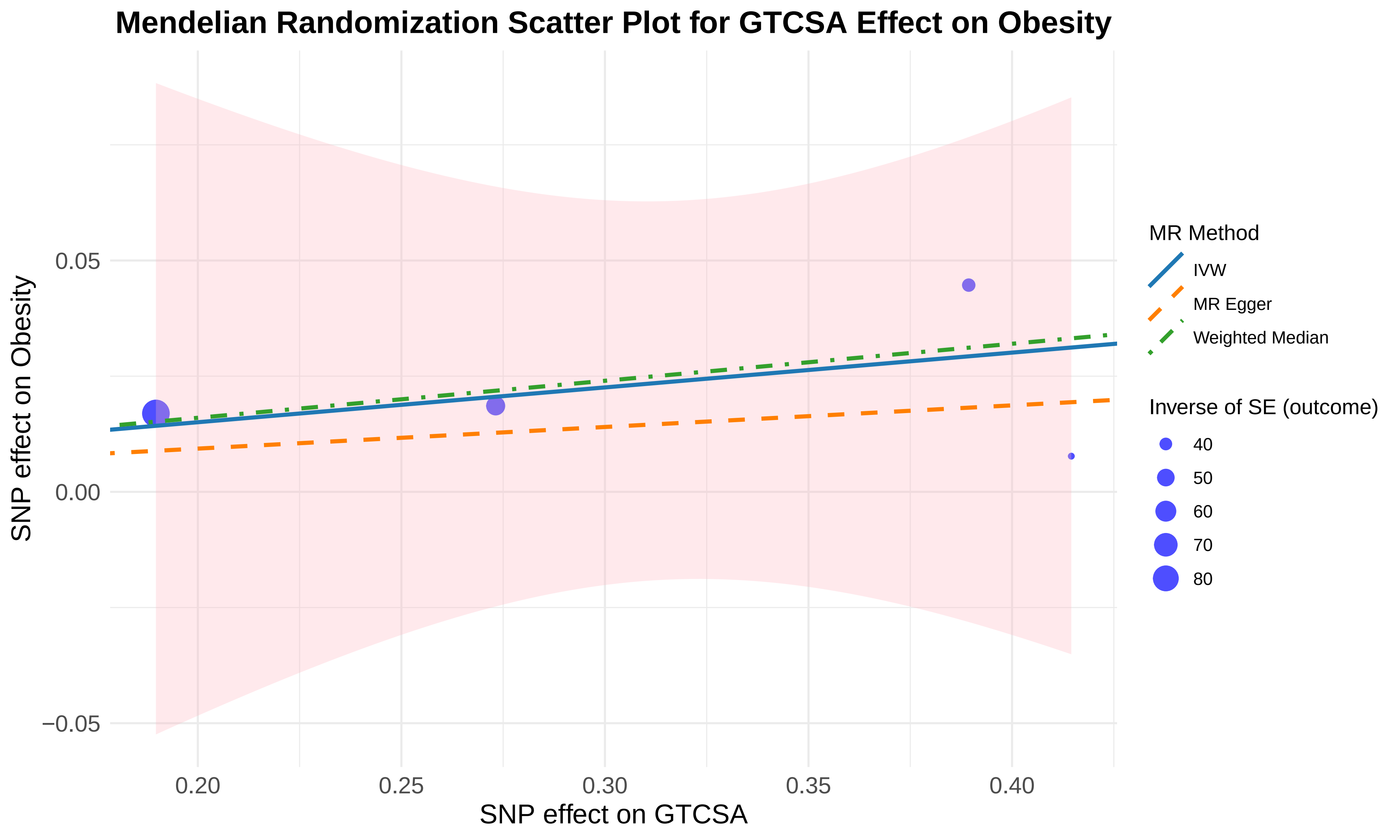

Mendelian Randomization Scatter Plot for GTCSA Effect on Obesity
SNP effect on Obesity
0.05
0.00
−0.05
0.20
0.25
0.30
0.35
0.40
SNP effect on GTCSA
MR Method
IVW
MR Egger
Weighted Median
Inverse of SE (outcome)
40
50
60
70
80

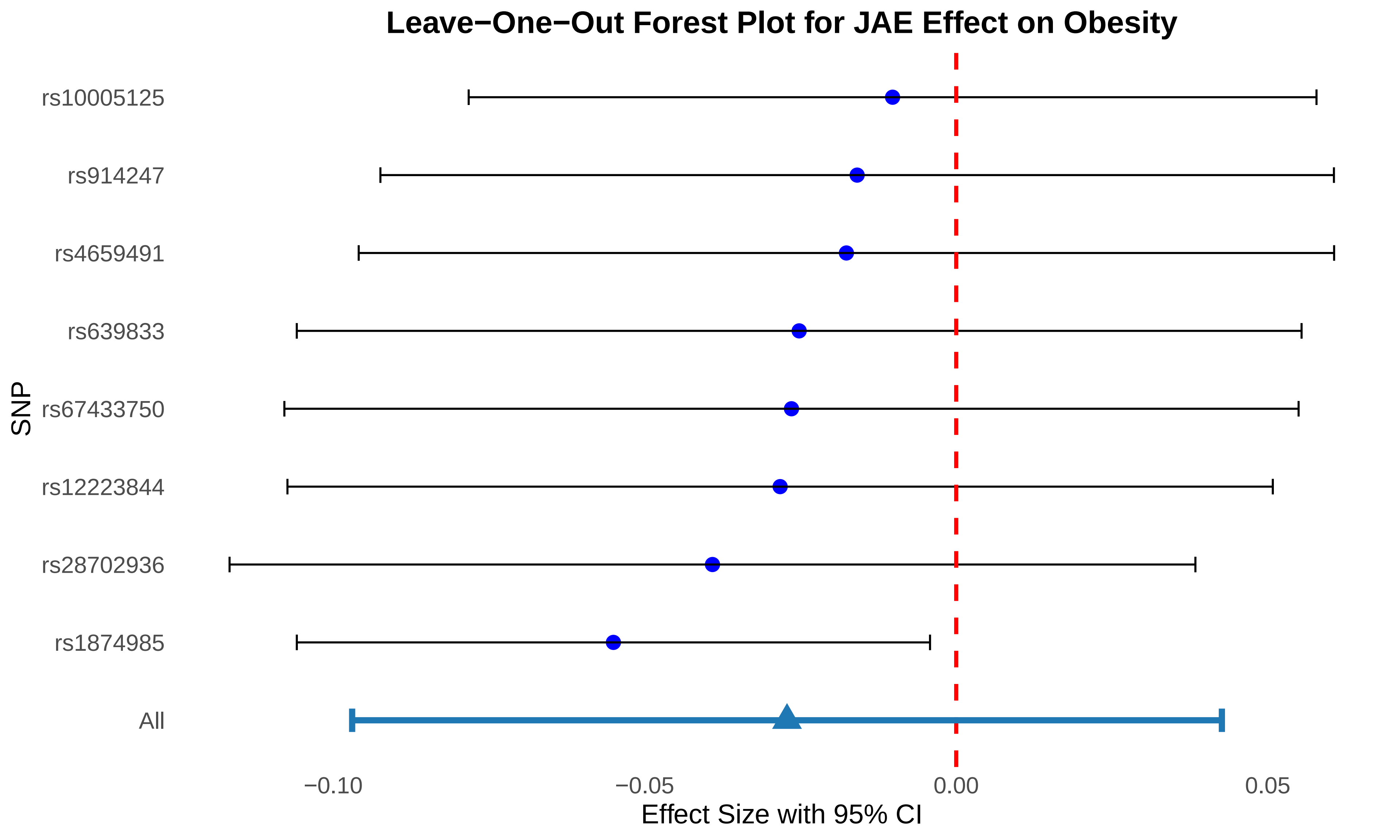

Leave−One−Out Forest Plot for JAE Effect on Obesity
SNP
rs10005125
rs914247
rs4659491
rs639833
rs67433750
rs12223844
rs28702936
rs1874985
All
−0.10
−0.05
0.00
0.05
Effect Size with 95% CI

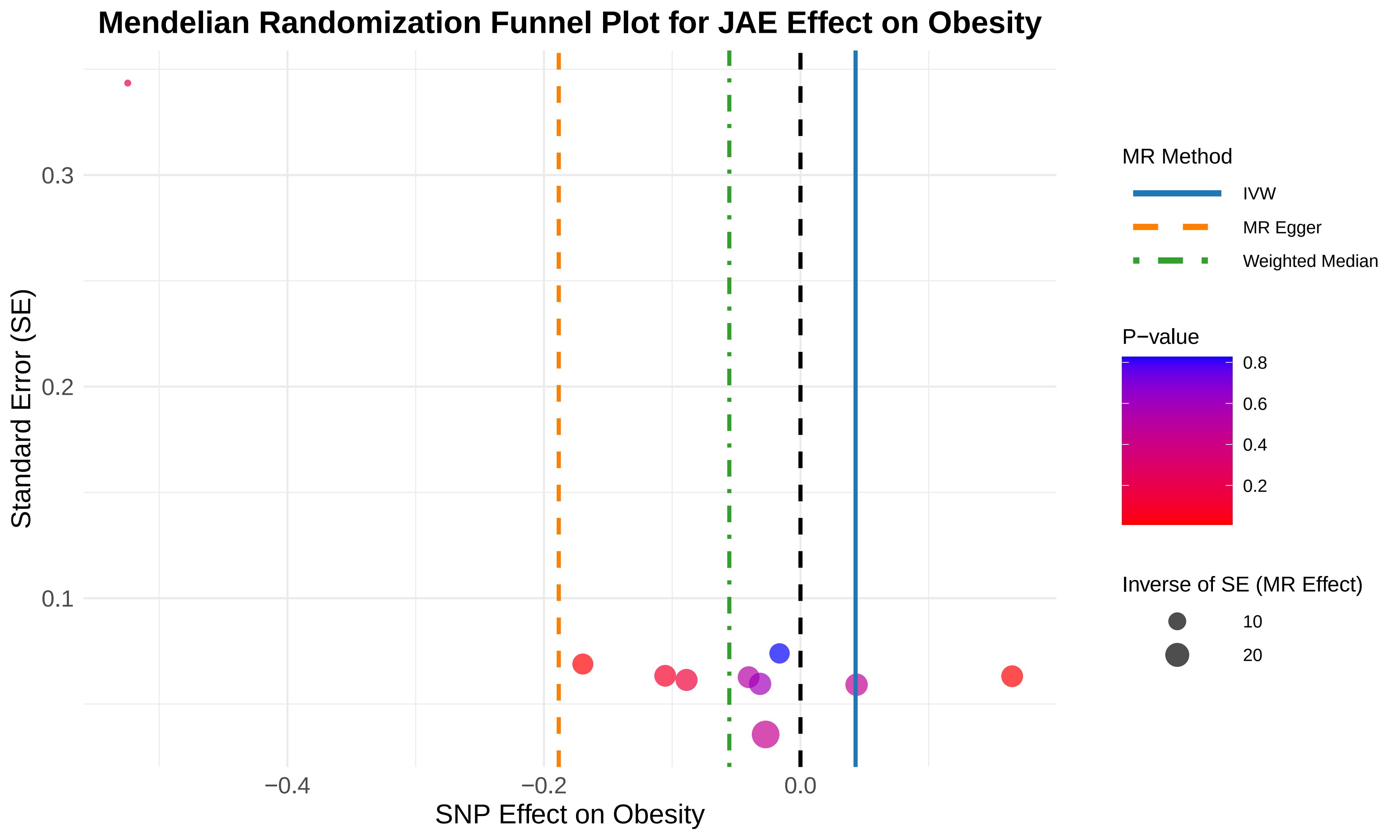

Mendelian Randomization Funnel Plot for JAE Effect on Obesity
Standard Error (SE)
0.3
0.2
0.1
−0.4
−0.2
0.0
SNP Effect on Obesity
MR Method
IVW
MR Egger
Weighted Median
P−value
0.8
0.6
0.4
0.2
Inverse of SE (MR Effect)
10
20

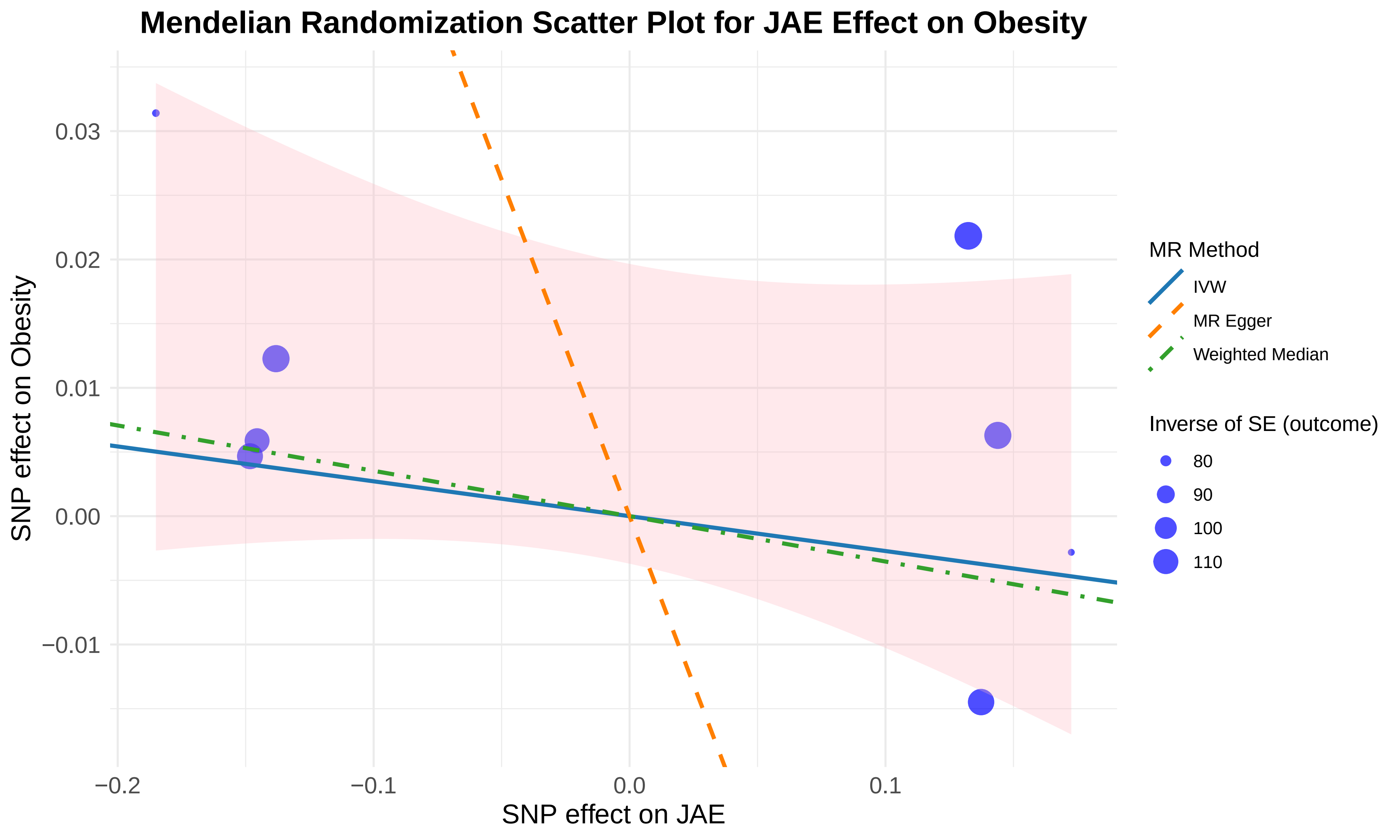

Mendelian Randomization Scatter Plot for JAE Effect on Obesity
SNP effect on Obesity
SNP effect on JAE
0.03
0.02
0.01
0.00
−0.01
−0.2
−0.1
0.0
0.1
MR Method
IVW
MR Egger
Weighted Median
Inverse of SE (outcome)
80
90
100
110

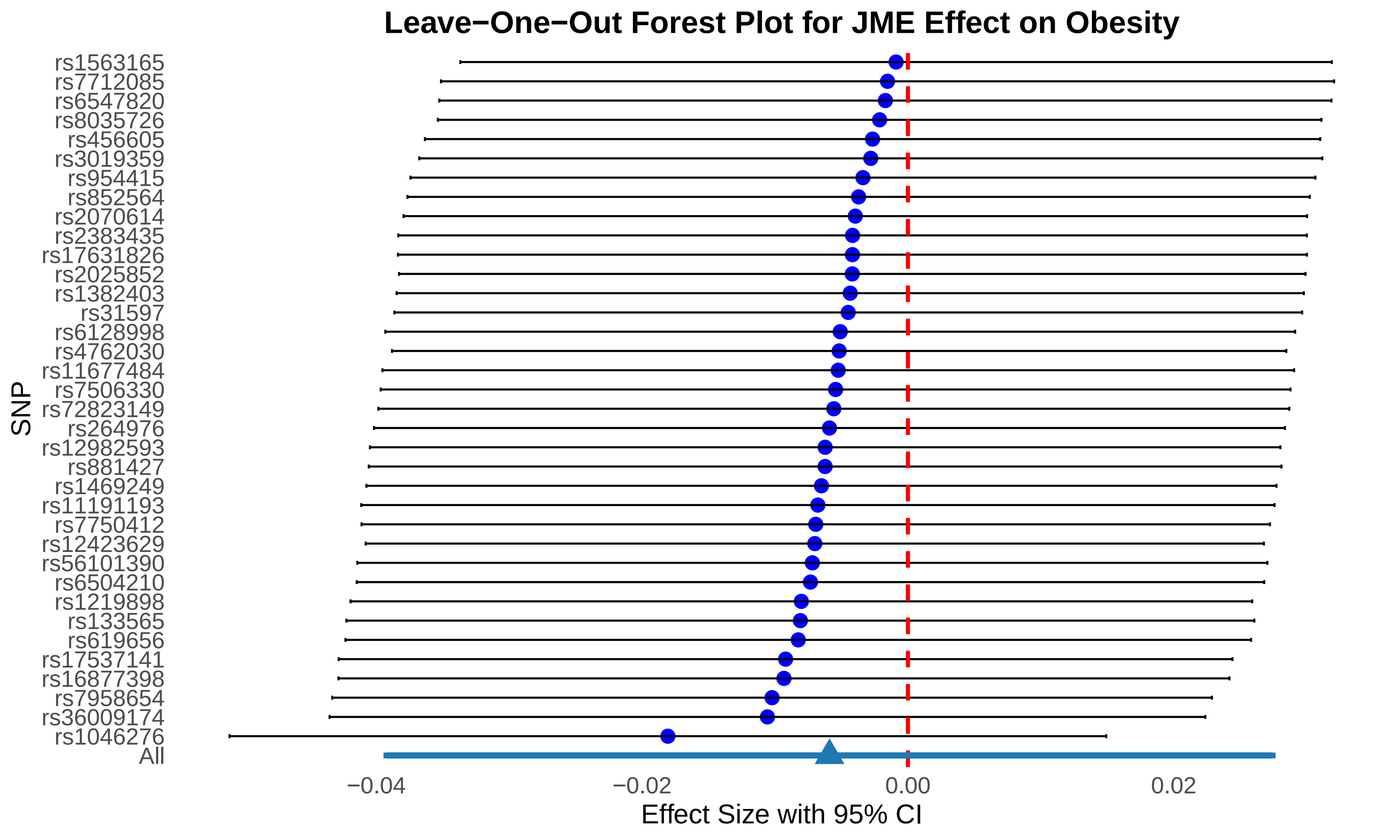
Leave−One−Out Forest Plot for JME Effect on Obesity
rs1563165
rs7712085
rs6547820
rs8035726
rs456605
rs3019359
rs954415
rs852564
rs2070614
rs2383435
rs17631826
rs2025852
rs1382403
rs31597
rs6128998
rs4762030
rs11677484
rs7506330
rs72823149
rs264976
rs12982593
rs881427
rs1469249
rs11191193
rs7750412
rs12423629
rs56101390
rs6504210
rs1219898
rs133565
rs619656
rs17537141
rs16877398
rs7958654
rs36009174
rs1046276
All
SNP
−0.04
−0.02
0.00
0.02
Effect Size with 95% CI

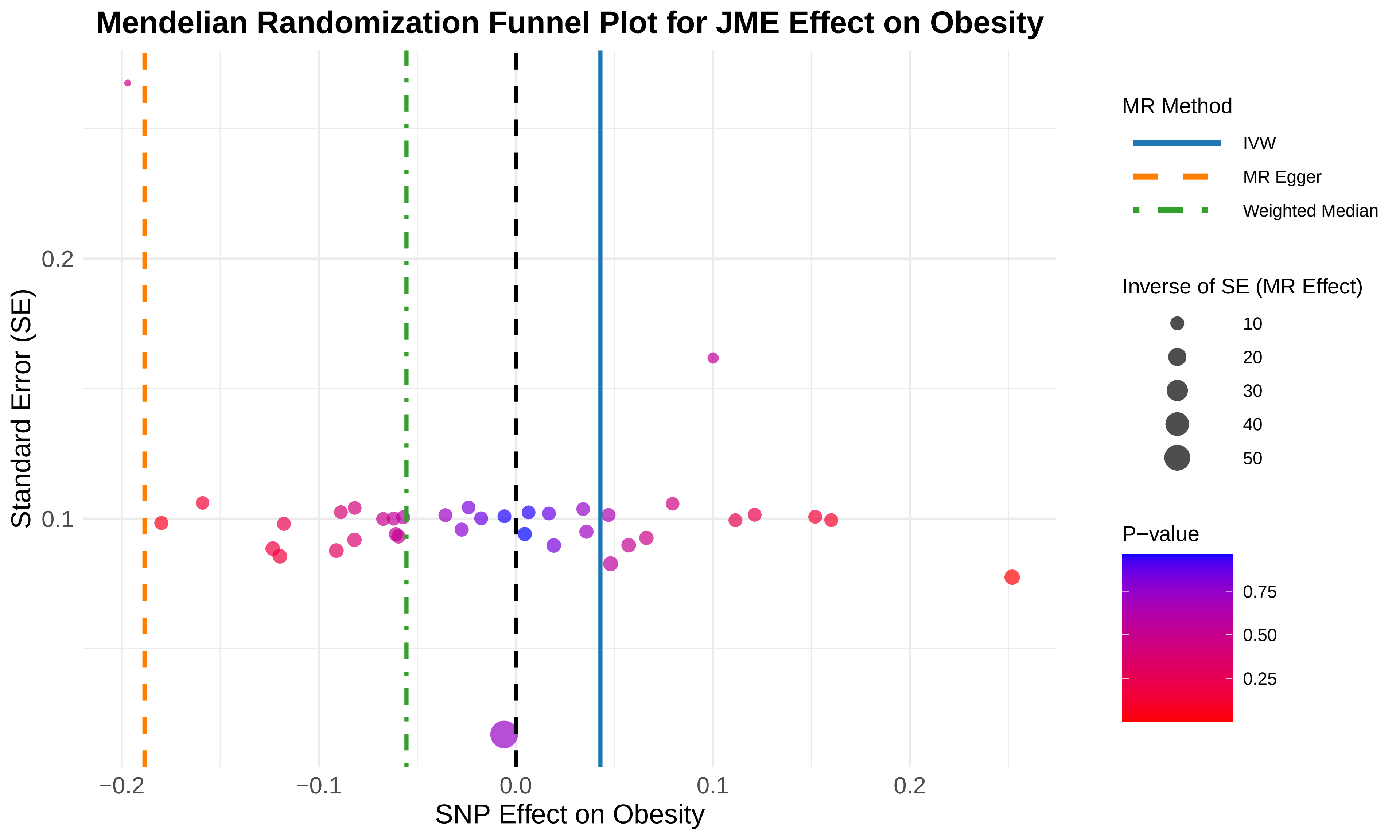
Mendelian Randomization Funnel Plot for JME Effect on Obesity
Standard Error (SE)
0.2
0.1
−0.2
−0.1
0.0
0.1
0.2
SNP Effect on Obesity
MR Method
IVW
MR Egger
Weighted Median
Inverse of SE (MR Effect)
10
20
30
40
50
P−value
0.75
0.50
0.25

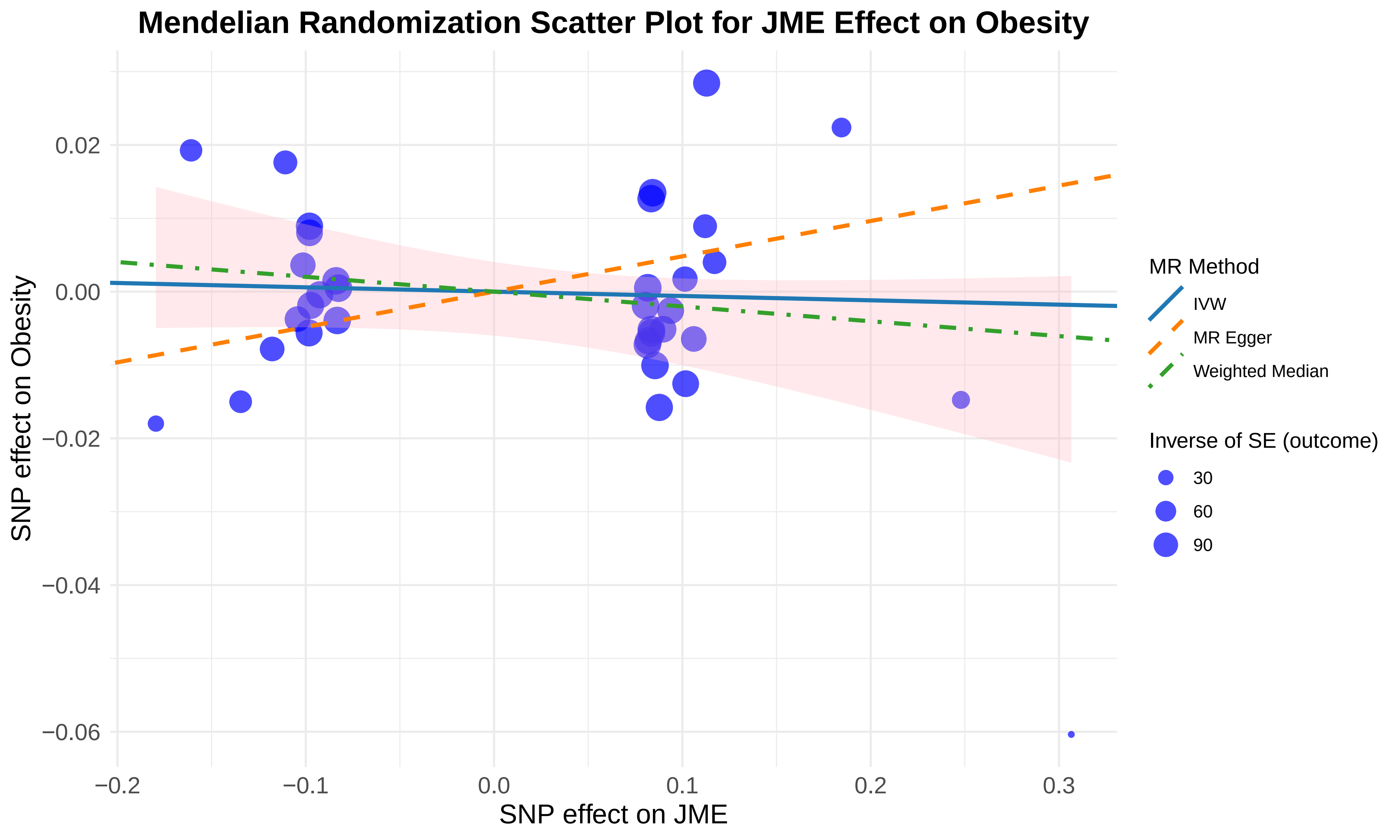

Mendelian Randomization Scatter Plot for JME Effect on Obesity
SNP effect on Obesity
SNP effect on JME
0.02
0.00
−0.02
−0.04
−0.06
−0.2
−0.1
0.0
0.1
0.2
0.3
MR Method
IVW
MR Egger
Weighted Median
Inverse of SE (outcome)
30
60
90

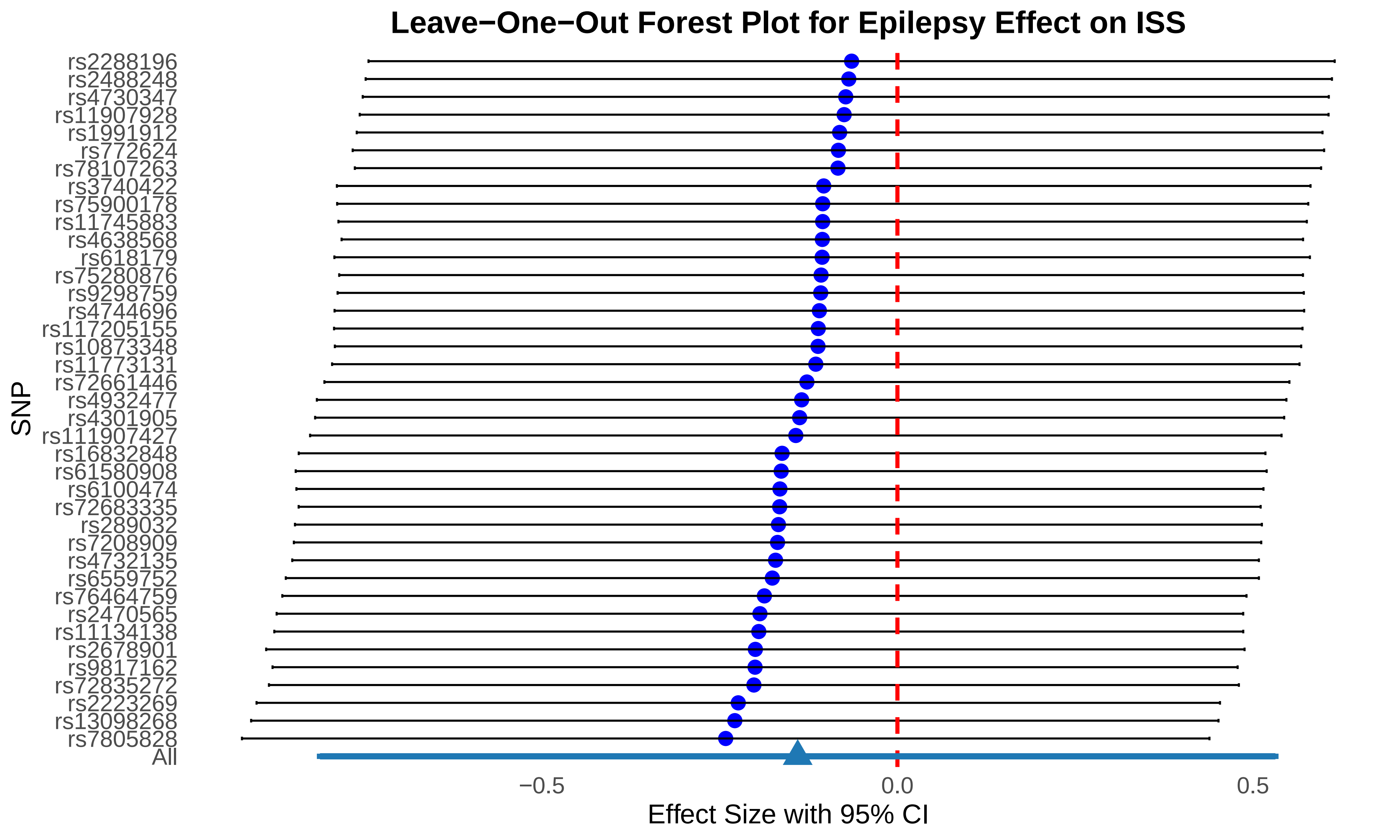
Leave−One−Out Forest Plot for Epilepsy Effect on ISS
rs2288196
rs2488248
rs4730347
rs11907928
rs1991912
rs772624
rs78107263
rs3740422
rs75900178
rs11745883
rs4638568
rs618179
rs75280876
rs9298759
rs4744696
rs117205155
rs10873348
rs11773131
rs72661446
rs4932477
rs4301905
rs111907427
rs16832848
rs61580908
rs6100474
rs72683335
rs289032
rs7208909
rs4732135
rs6559752
rs76464759
rs2470565
rs11134138
rs2678901
rs9817162
rs72835272
rs2223269
rs13098268
rs7805828
All
SNP
−0.5
0.0
0.5
Effect Size with 95% CI

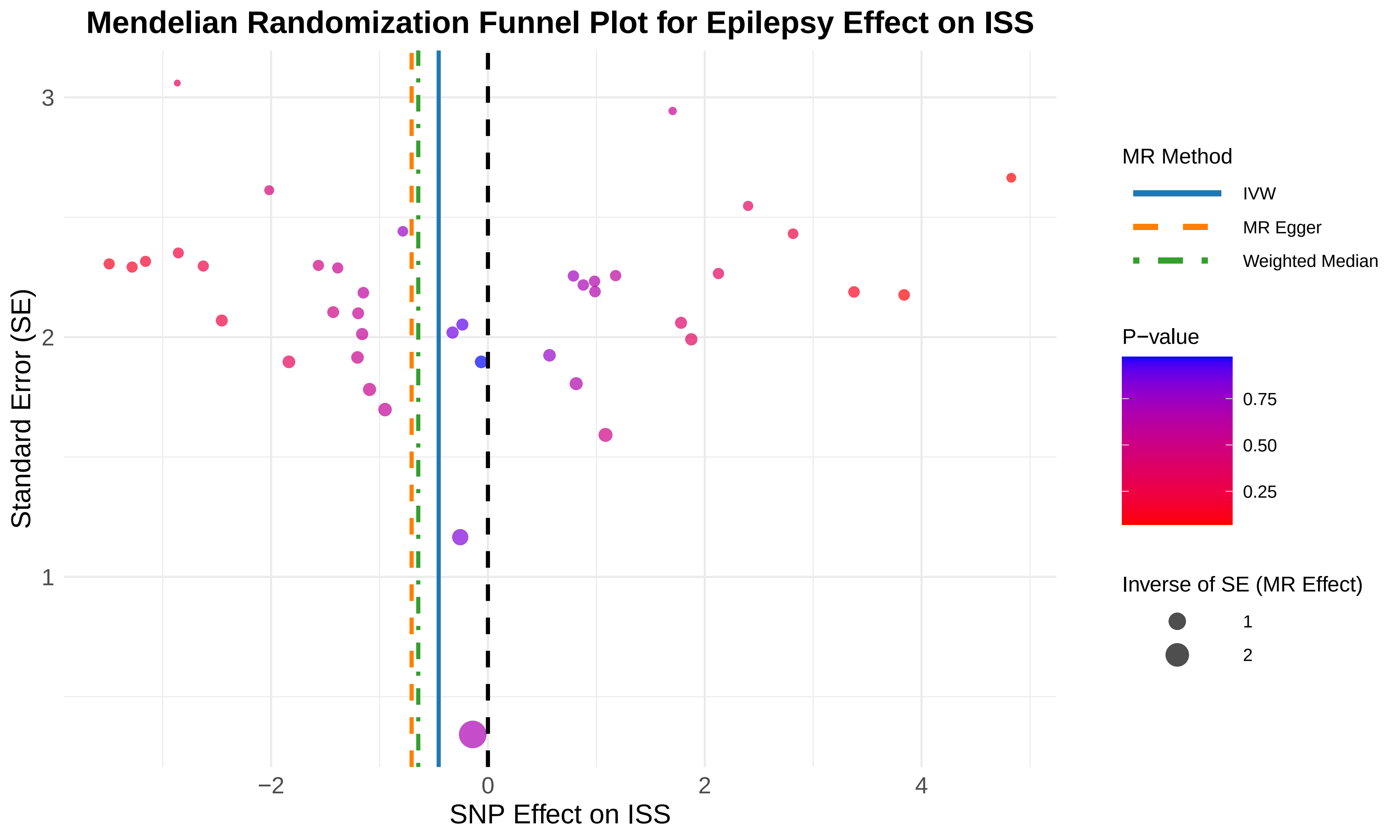

Mendelian Randomization Funnel Plot for Epilepsy Effect on ISS
Standard Error (SE)
3
2
1
−2
0
2
4
SNP Effect on ISS
MR Method
IVW
MR Egger
Weighted Median
P−value
0.75
0.50
0.25
Inverse of SE (MR Effect)
1
2

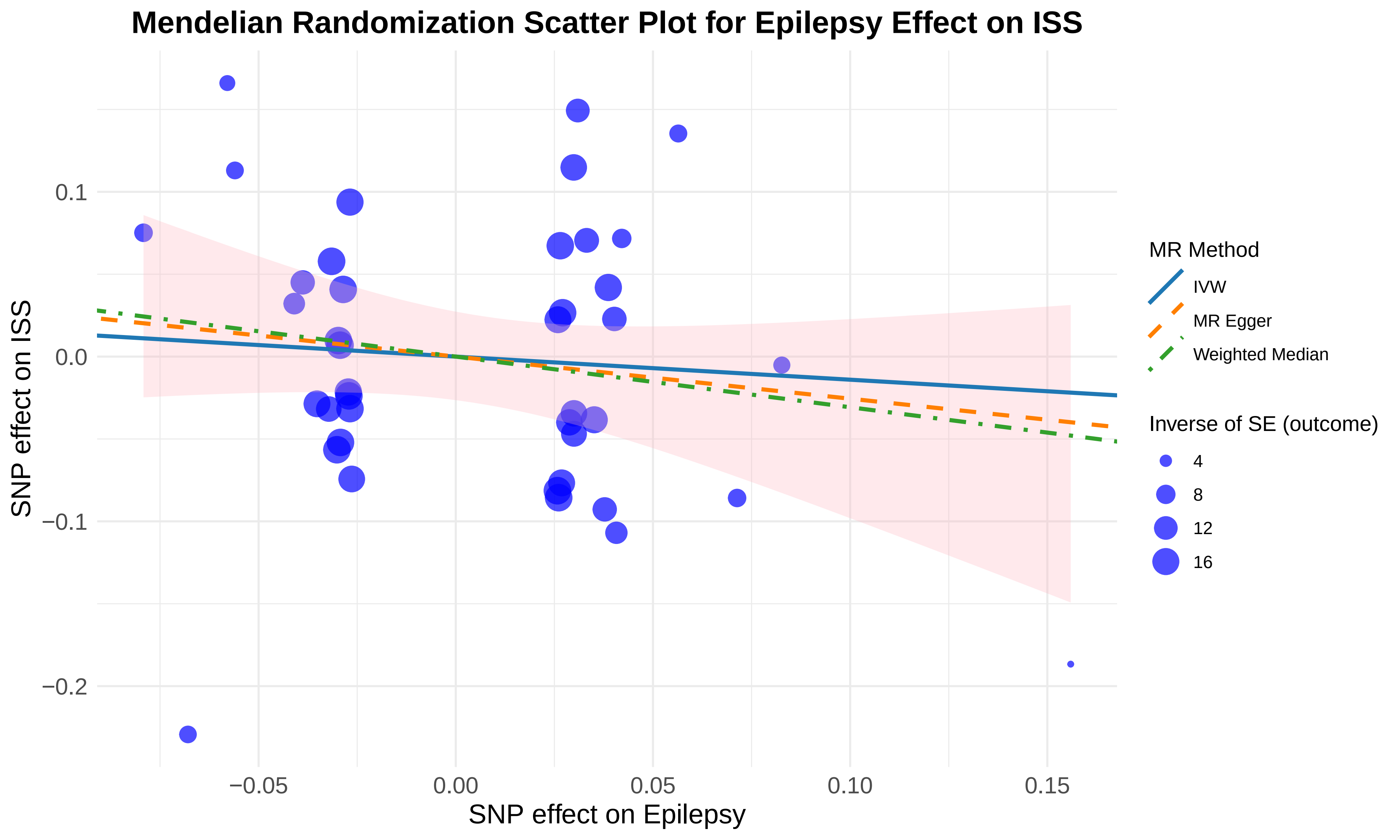
Mendelian Randomization Scatter Plot for Epilepsy Effect on ISS
SNP effect on ISS
SNP effect on Epilepsy
0.1
0.0
−0.1
−0.2
−0.05
0.00
0.05
0.10
0.15
MR Method
IVW
MR Egger
Weighted Median
Inverse of SE (outcome)
4
8
12
16

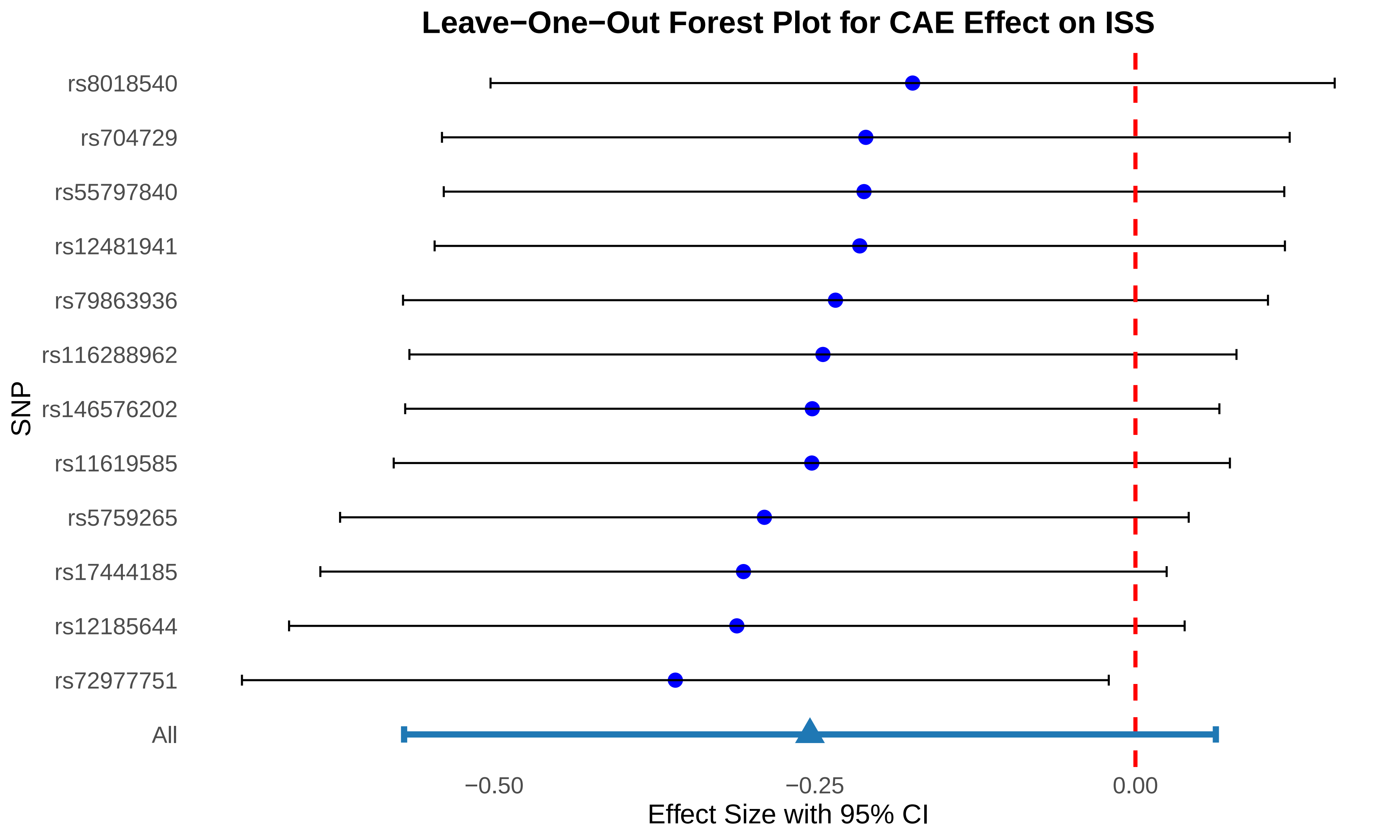

Leave−One−Out Forest Plot for CAE Effect on ISS
rs8018540
rs704729
rs55797840
rs12481941
rs79863936
rs116288962
rs146576202
rs11619585
rs5759265
rs17444185
rs12185644
rs72977751
All
SNP
−0.50
−0.25
0.00
Effect Size with 95% CI

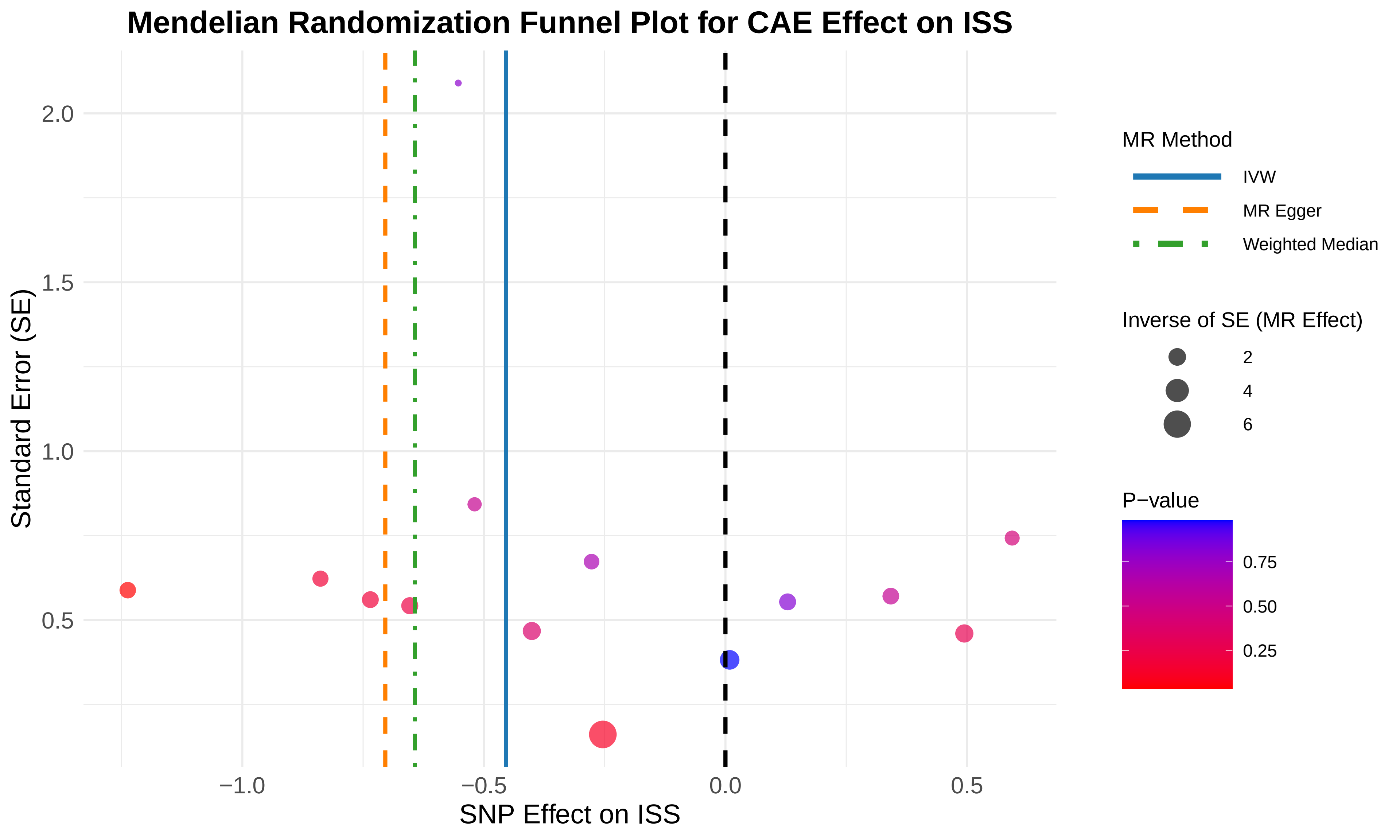
Mendelian Randomization Funnel Plot for CAE Effect on ISS
Standard Error (SE)
2.0
1.5
1.0
0.5
−1.0
−0.5
0.0
0.5
SNP Effect on ISS
MR Method
IVW
MR Egger
Weighted Median
Inverse of SE (MR Effect)
2
4
6
P−value
0.75
0.50
0.25

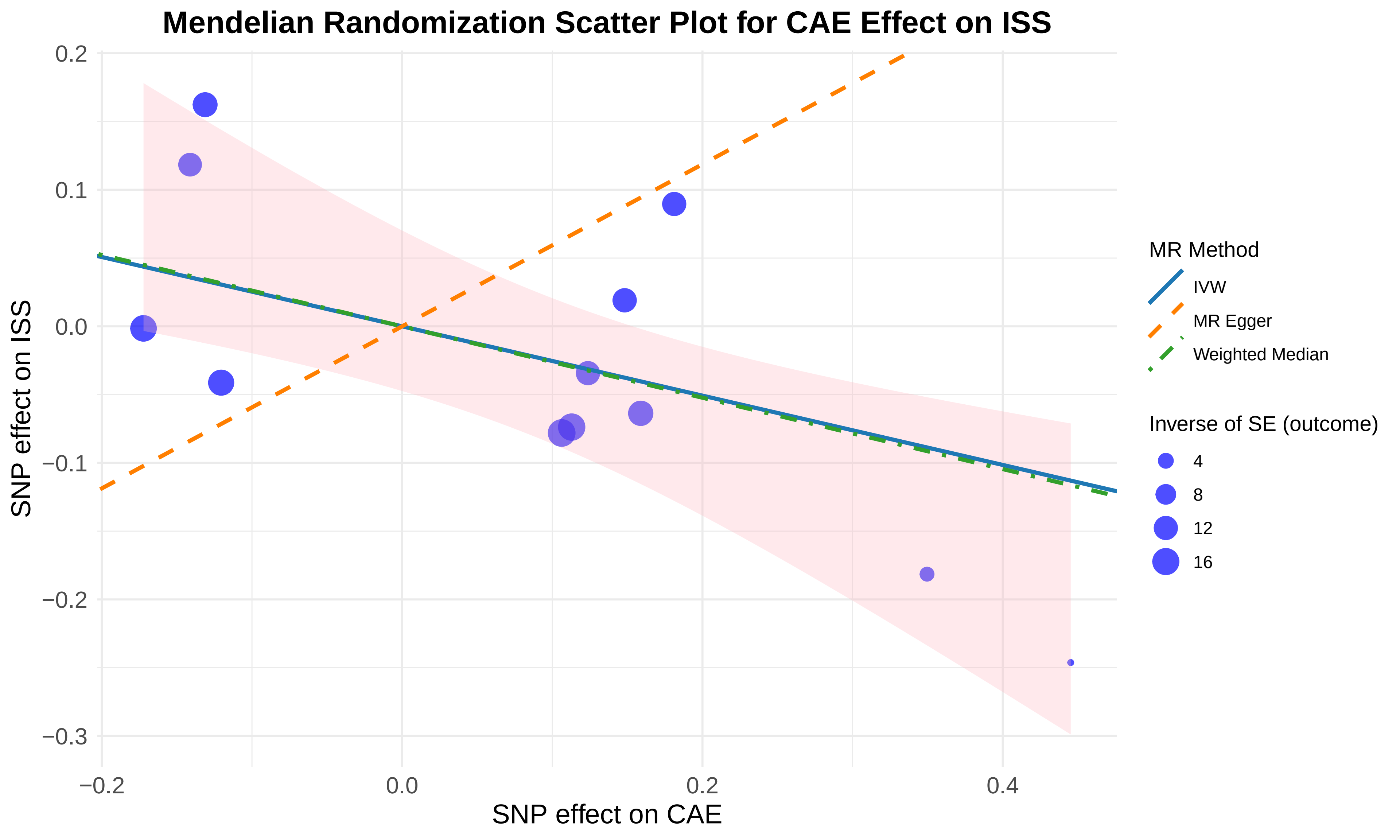

Mendelian Randomization Scatter Plot for CAE Effect on ISS
0.2
0.1
0.0
−0.1
−0.2
−0.3
SNP effect on ISS
−0.2
0.0
0.2
0.4
SNP effect on CAE
MR Method
IVW
MR Egger
Weighted Median
Inverse of SE (outcome)
4
8
12
16

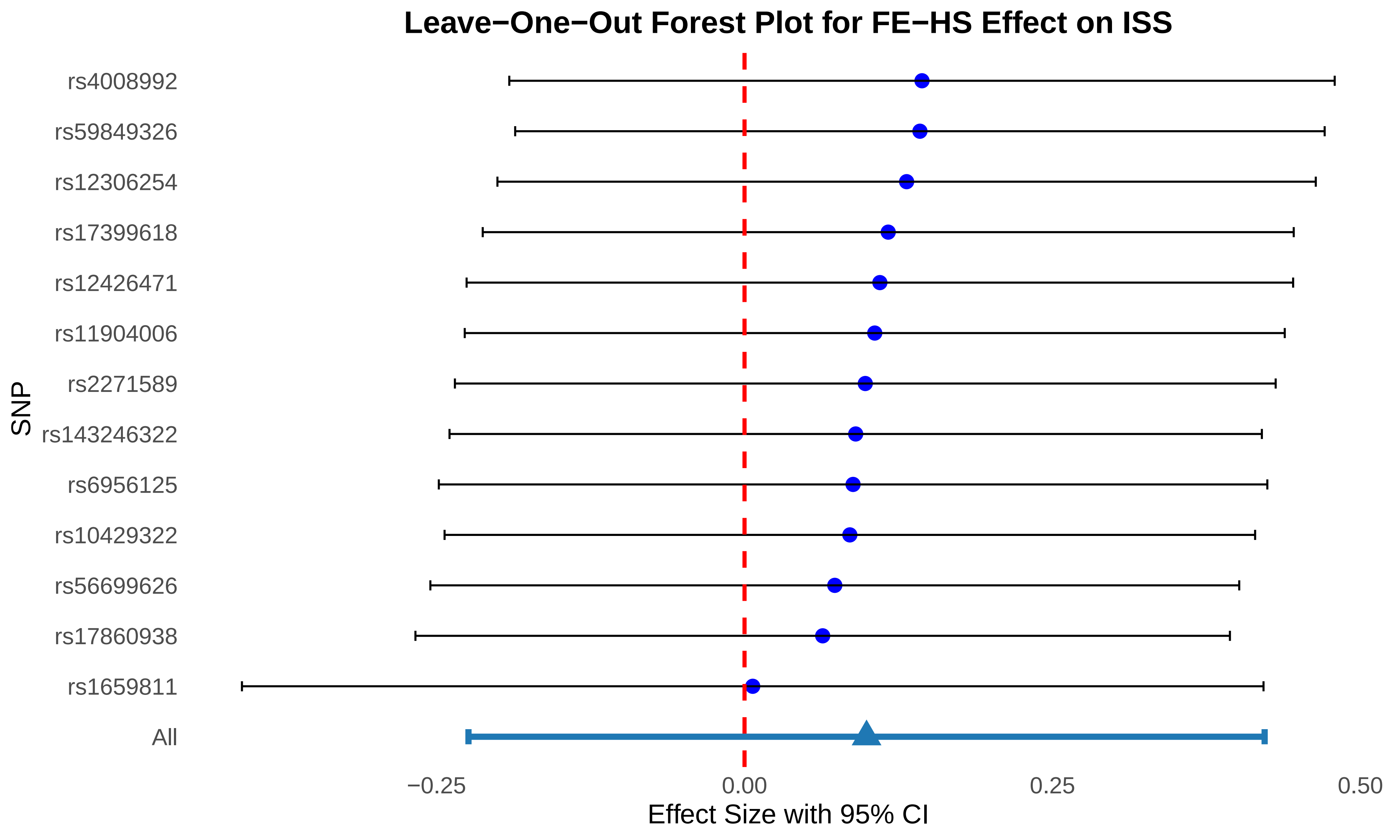

Leave−One−Out Forest Plot for FE−HS Effect on ISS
rs4008992
rs59849326
rs12306254
rs17399618
rs12426471
rs11904006
rs2271589
rs143246322
rs6956125
rs10429322
rs56699626
rs17860938
rs1659811
All
SNP
−0.25
0.00
0.25
0.50
Effect Size with 95% CI

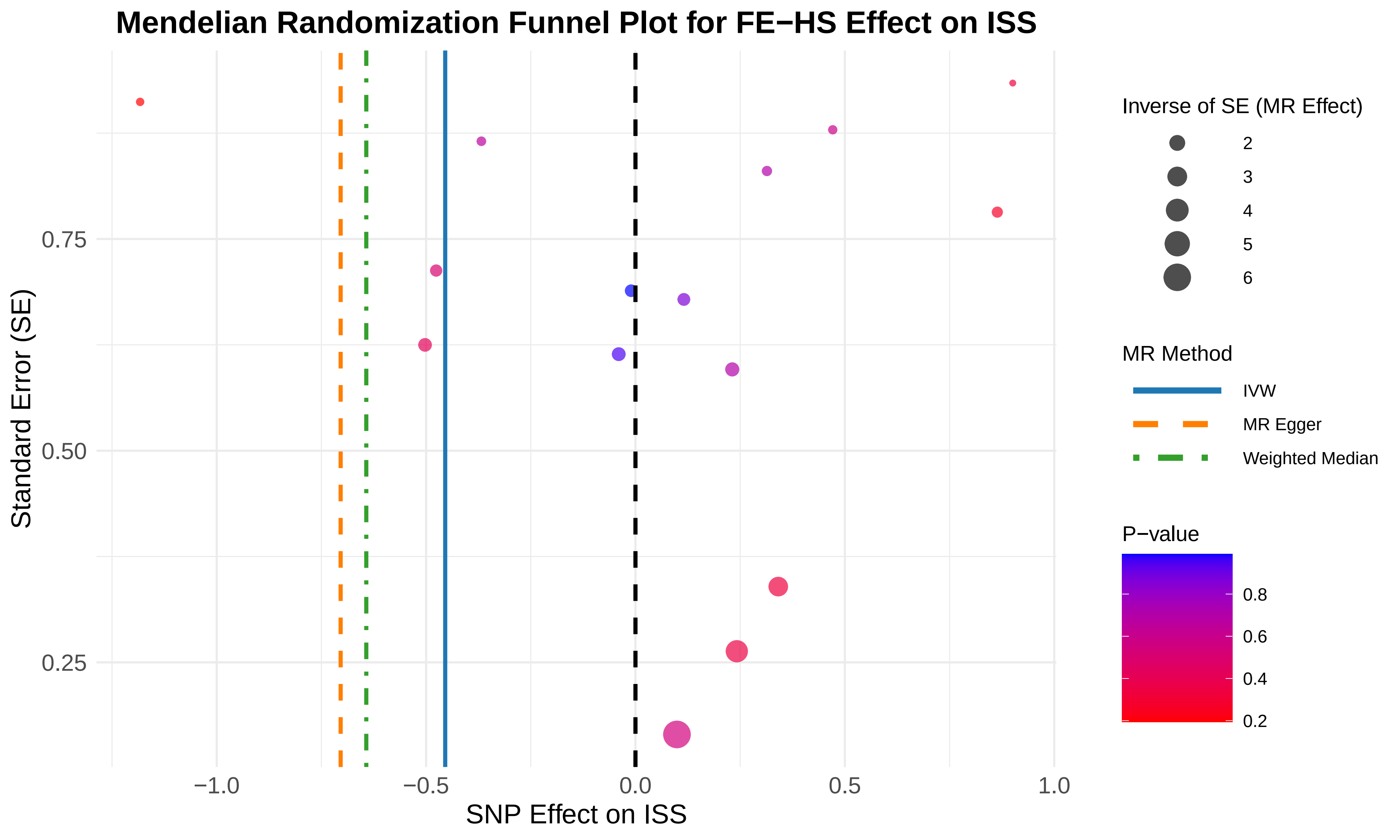

Mendelian Randomization Funnel Plot for FE−HS Effect on ISS
Standard Error (SE)
0.75
0.50
0.25
−1.0
−0.5
0.0
0.5
1.0
SNP Effect on ISS
Inverse of SE (MR Effect)
2
3
4
5
6
MR Method
IVW
MR Egger
Weighted Median
P−value
0.8
0.6
0.4
0.2

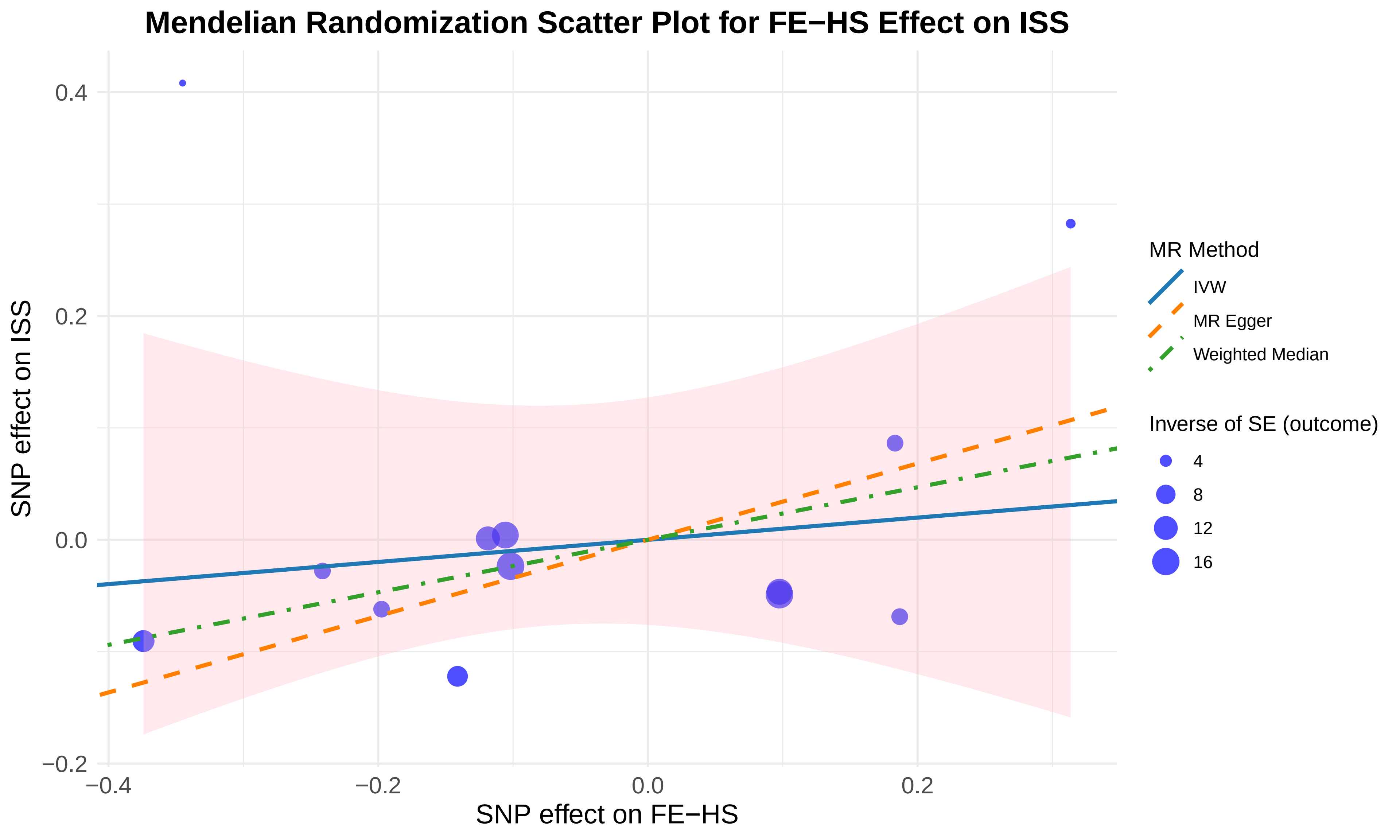
Mendelian Randomization Scatter Plot for FE−HS Effect on ISS
0.4
0.2
0.0
−0.2
−0.4
−0.2
0.0
0.2
SNP effect on ISS
SNP effect on FE−HS
MR Method
IVW
MR Egger
Weighted Median
Inverse of SE (outcome)
4
8
12
16

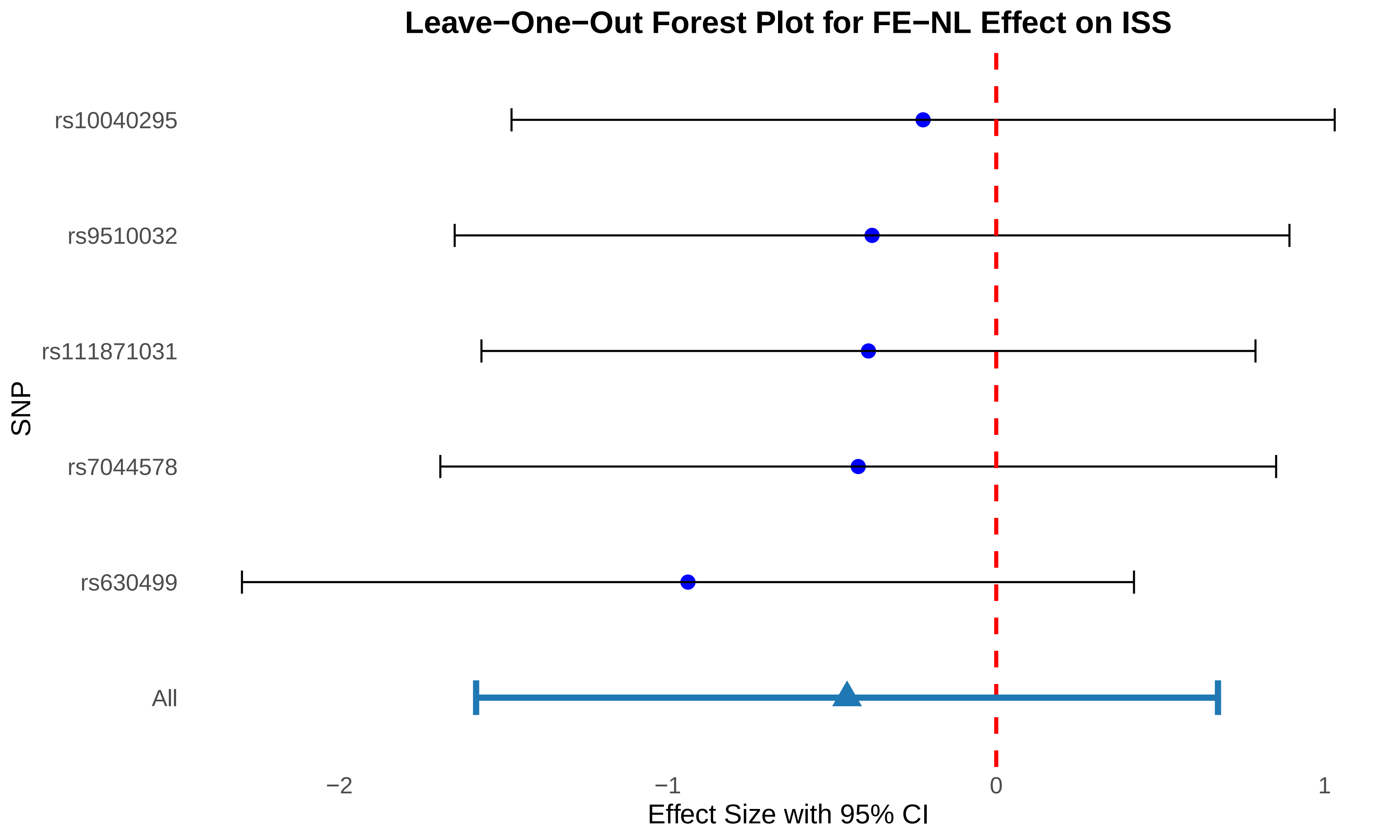
Leave−One−Out Forest Plot for FE−NL Effect on ISS
rs10040295
rs9510032
rs111871031
rs7044578
rs630499
All
SNP
−2
−1
0
1
Effect Size with 95% CI

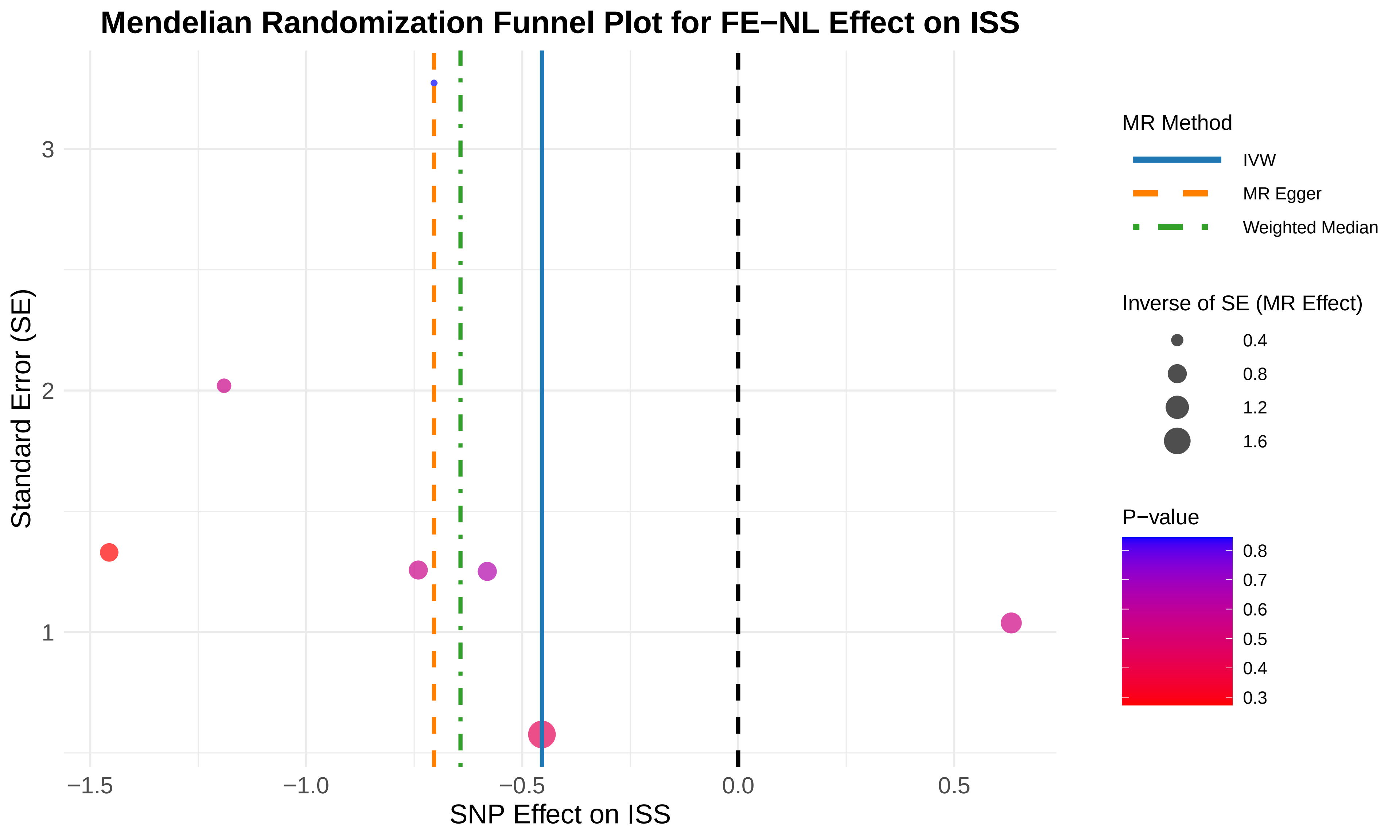

Mendelian Randomization Funnel Plot for FE−NL Effect on ISS
Standard Error (SE)
3
2
1
−1.5
−1.0
−0.5
0.0
0.5
SNP Effect on ISS
MR Method
IVW
MR Egger
Weighted Median
Inverse of SE (MR Effect)
0.4
0.8
1.2
1.6
P−value
0.8
0.7
0.6
0.5
0.4
0.3

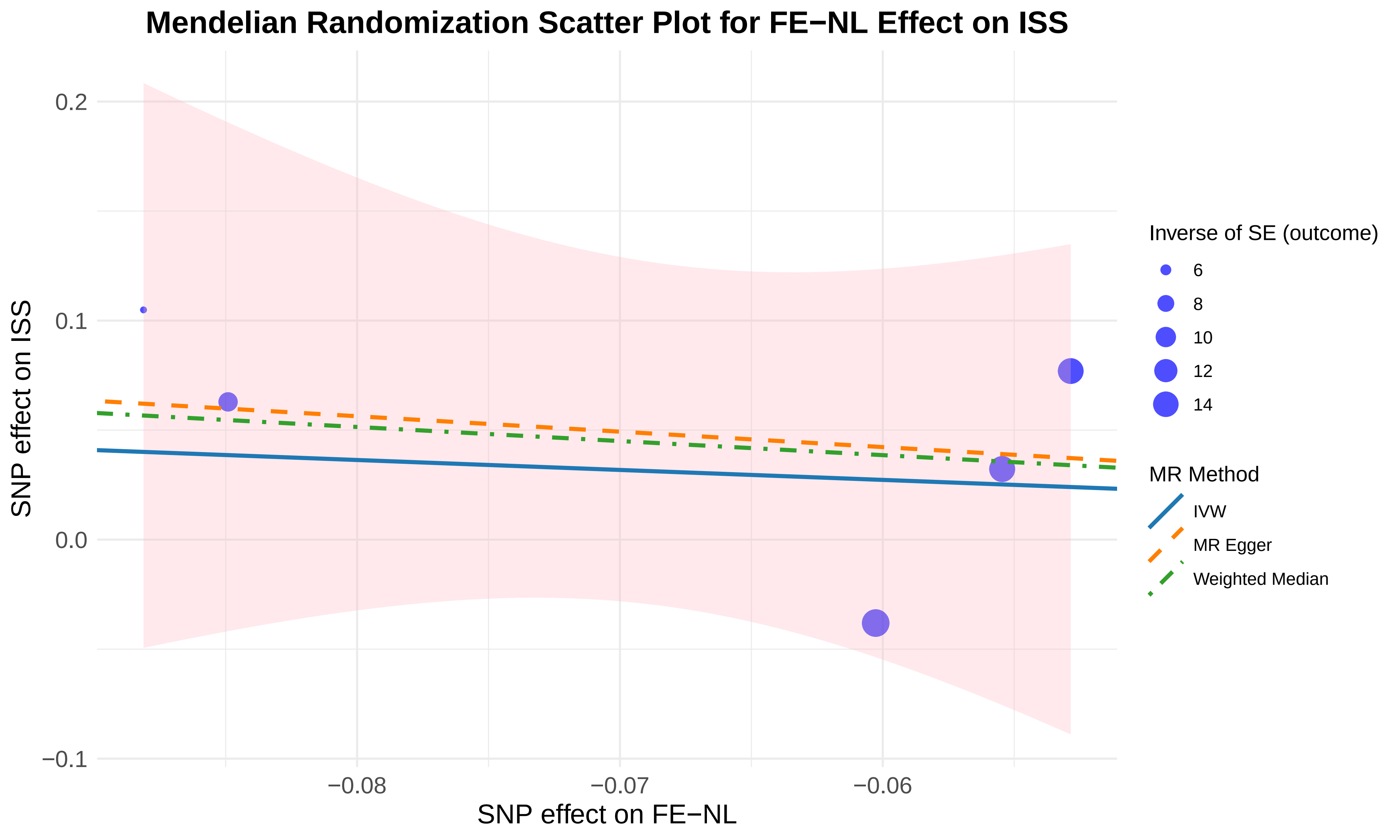

Mendelian Randomization Scatter Plot for FE−NL Effect on ISS
SNP effect on ISS
SNP effect on FE−NL
0.2
0.1
0.0
−0.1
−0.08
−0.07
−0.06
Inverse of SE (outcome)
6
8
10
12
14
MR Method
IVW
MR Egger
Weighted Median

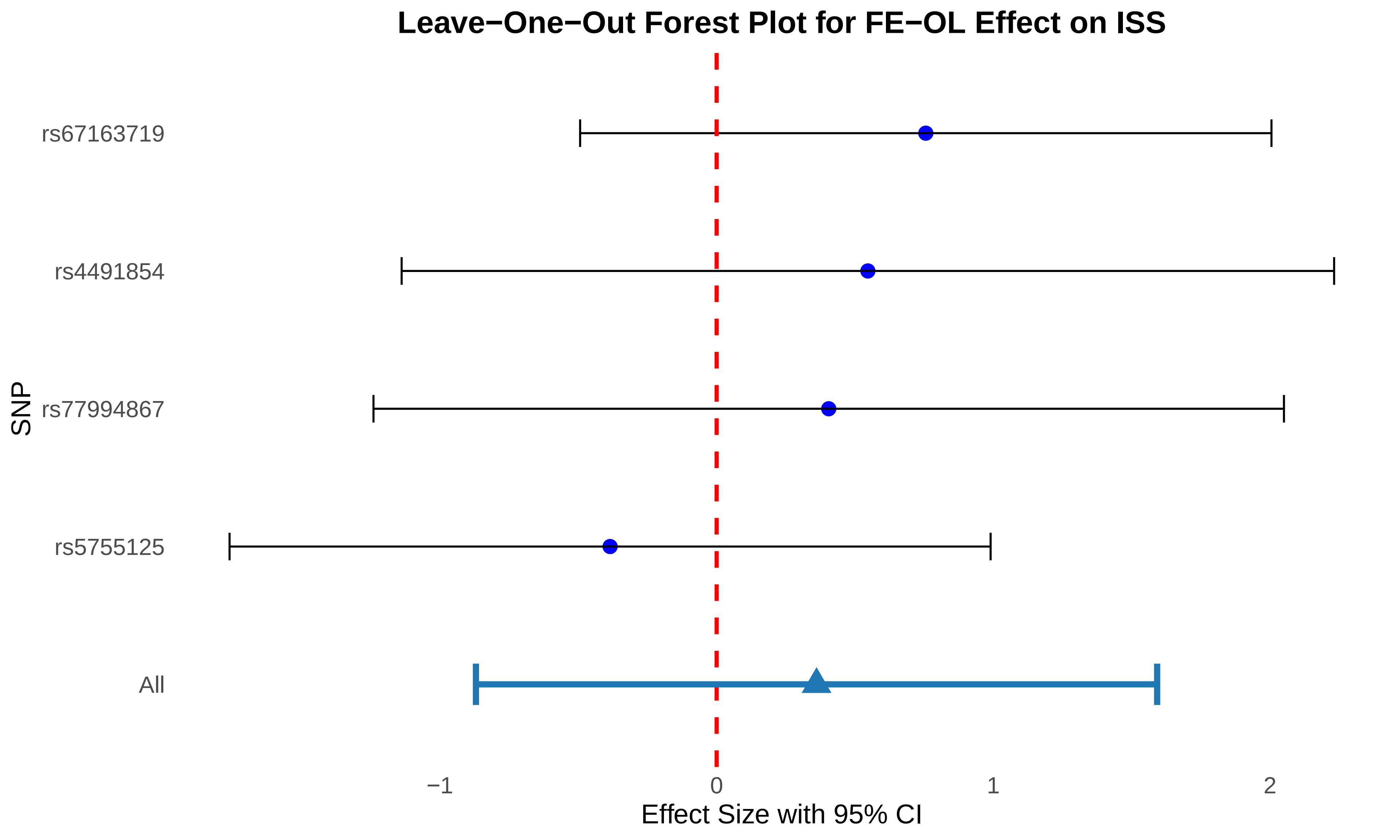
Leave−One−Out Forest Plot for FE−OL Effect on ISS
rs67163719
rs4491854
rs77994867
rs5755125
All
SNP
−1
0
1
2
Effect Size with 95% CI

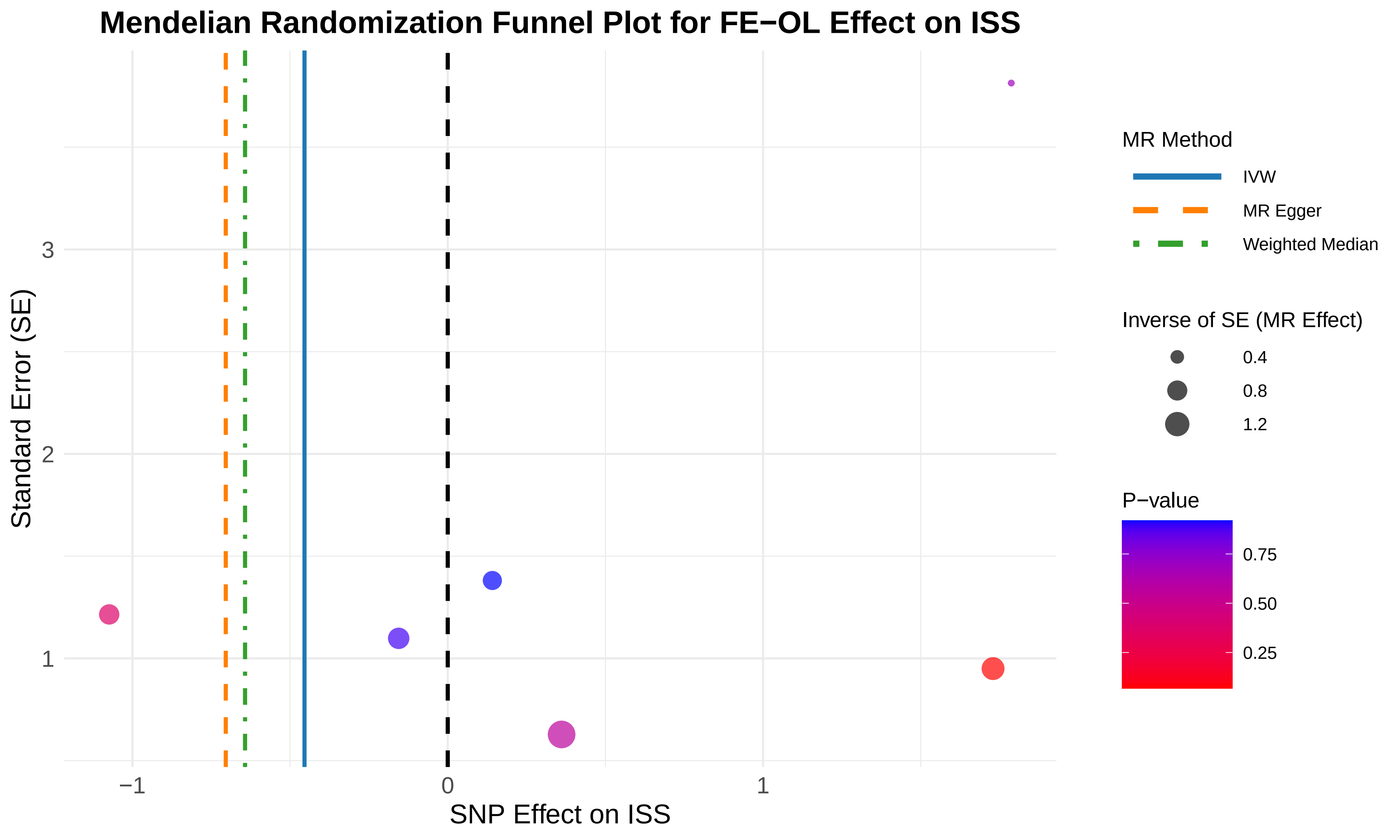

Mendelian Randomization Funnel Plot for FE−OL Effect on ISS
Standard Error (SE)
3
2
1
−1
0
1
SNP Effect on ISS
MR Method
IVW
MR Egger
Weighted Median
Inverse of SE (MR Effect)
0.4
0.8
1.2
P−value
0.75
0.50
0.25

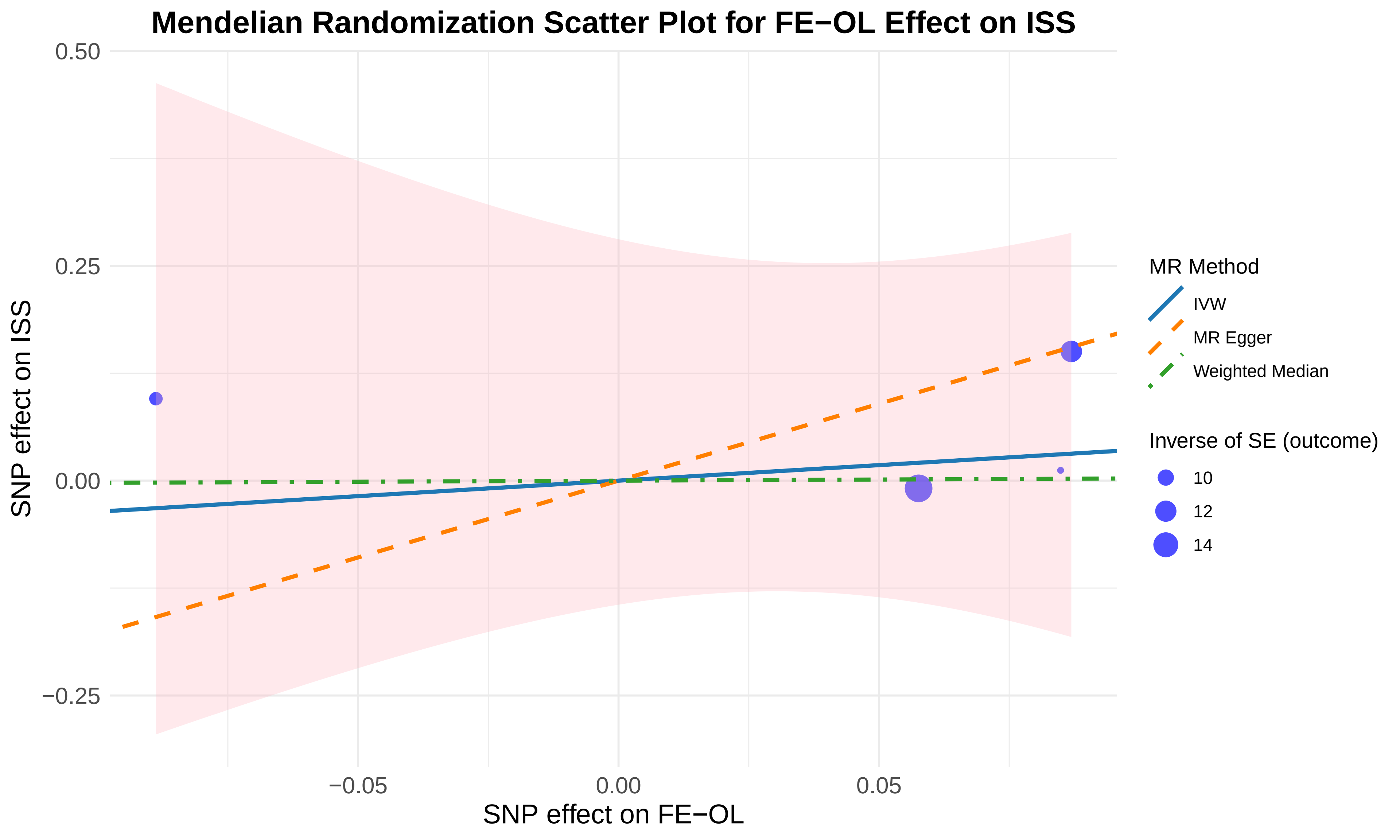
Mendelian Randomization Scatter Plot for FE−OL Effect on ISS
0.50
0.25
0.00
−0.25
SNP effect on ISS
−0.05
0.00
0.05
SNP effect on FE−OL
MR Method
IVW
MR Egger
Weighted Median
Inverse of SE (outcome)
10
12
14

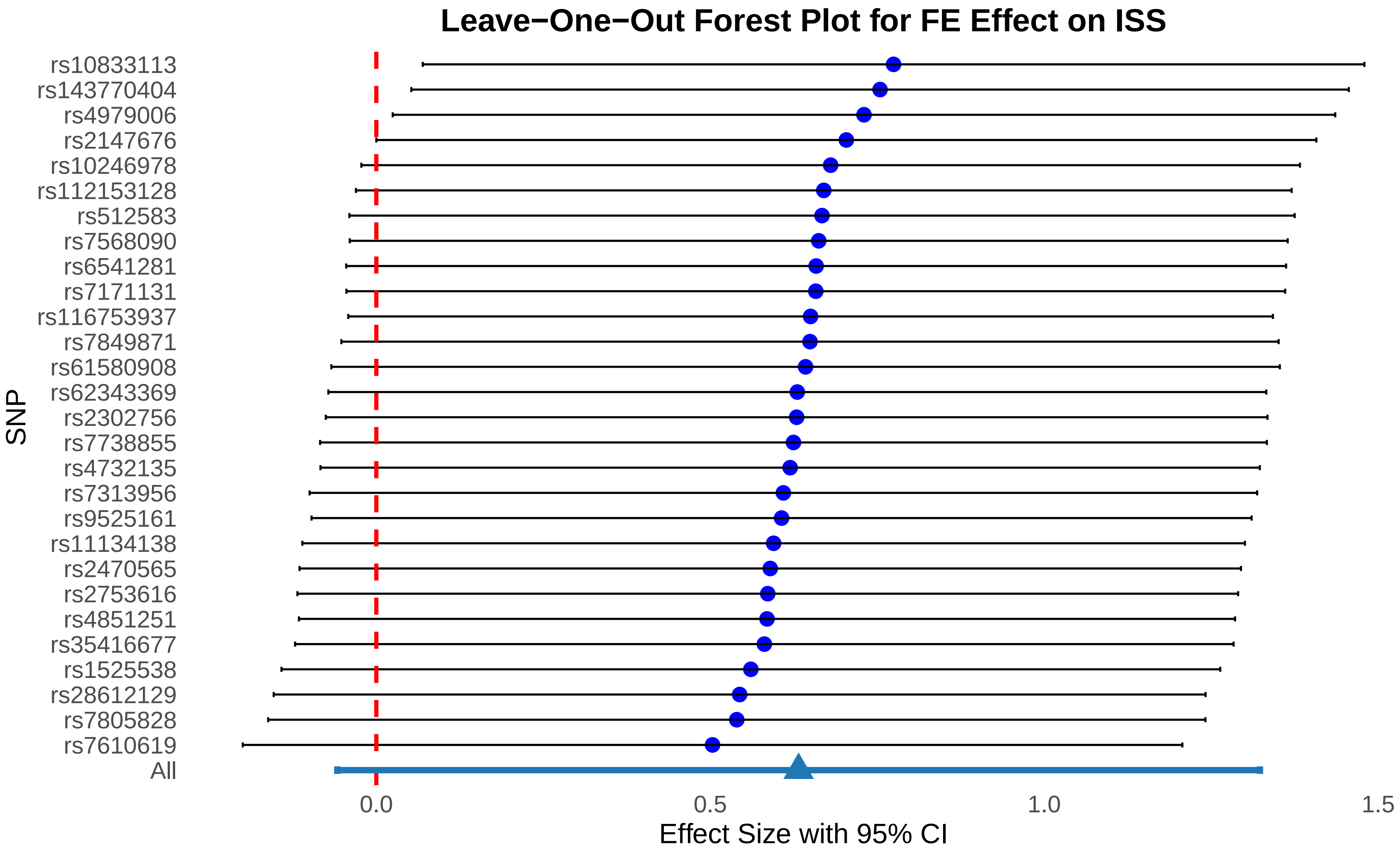
Leave−One−Out Forest Plot for FE Effect on ISS
SNP
rs10833113
rs143770404
rs4979006
rs2147676
rs10246978
rs112153128
rs512583
rs7568090
rs6541281
rs7171131
rs116753937
rs7849871
rs61580908
rs62343369
rs2302756
rs7738855
rs4732135
rs7313956
rs9525161
rs11134138
rs2470565
rs2753616
rs4851251
rs35416677
rs1525538
rs28612129
rs7805828
rs7610619
All
0.0
0.5
1.0
1.5
Effect Size with 95% CI

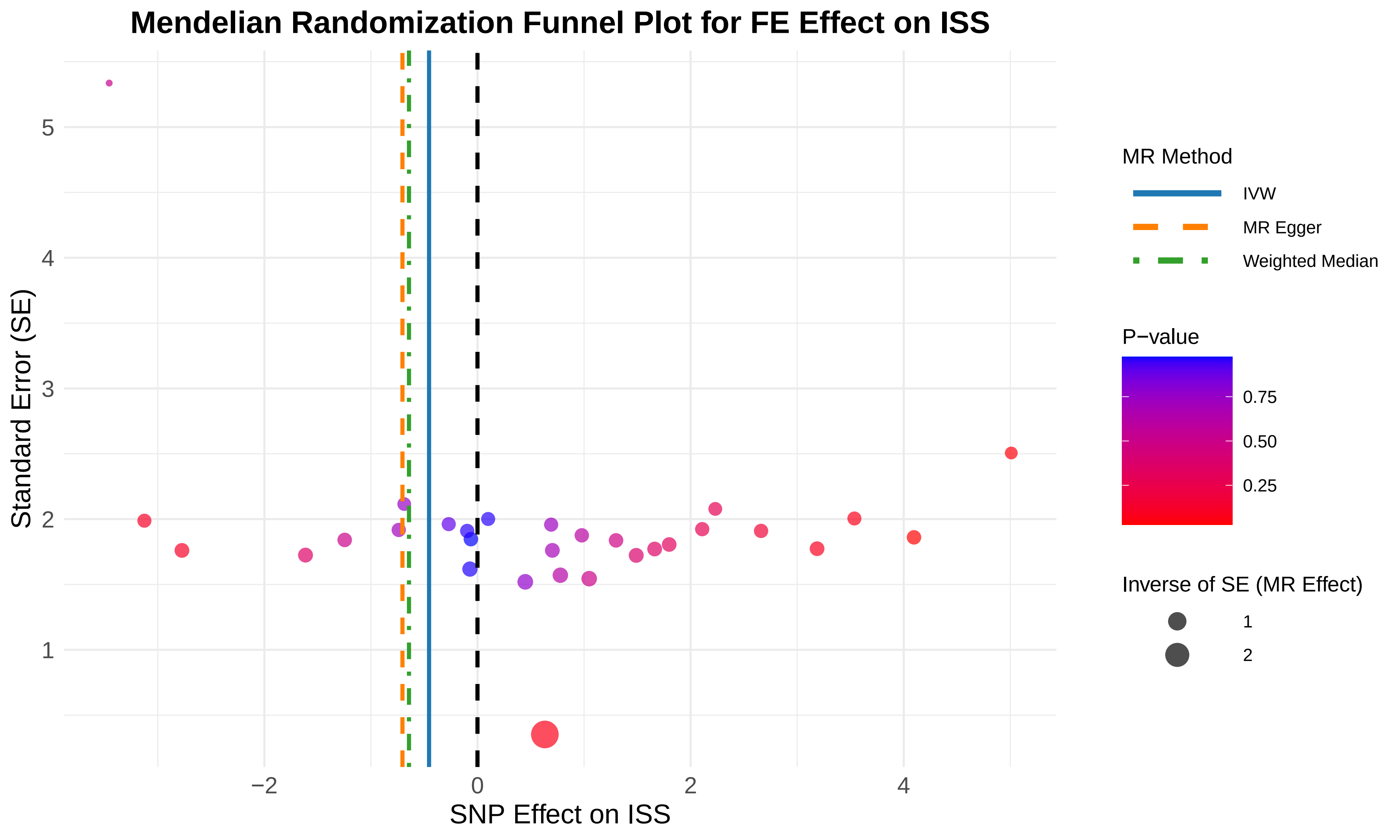
Mendelian Randomization Funnel Plot for FE Effect on ISS
Standard Error (SE)
SNP Effect on ISS
5
4
3
2
1
−2
0
2
4
MR Method
IVW
MR Egger
Weighted Median
P−value
0.75
0.50
0.25
Inverse of SE (MR Effect)
1
2

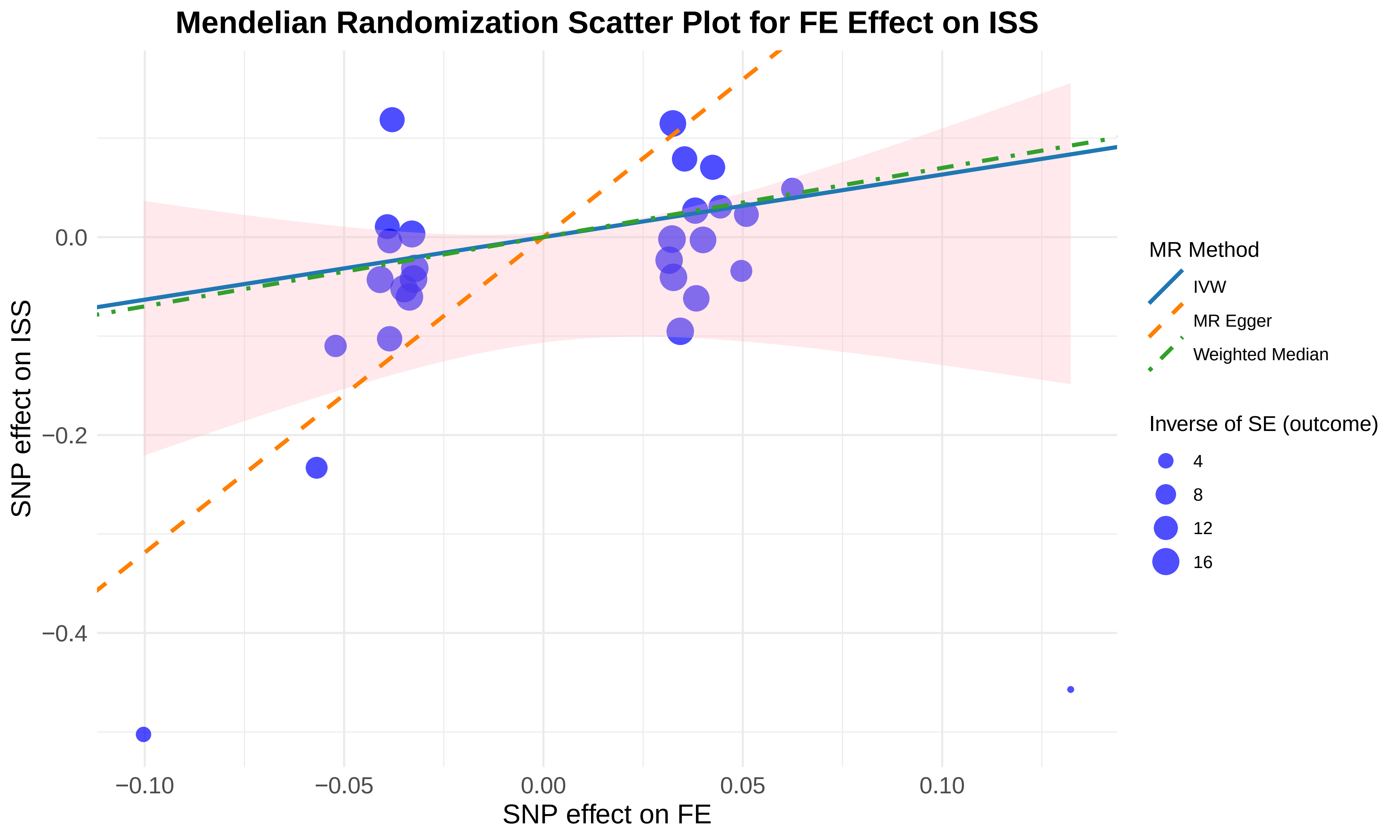

Mendelian Randomization Scatter Plot for FE Effect on ISS
SNP effect on ISS
SNP effect on FE
0.0
−0.2
−0.4
−0.10
−0.05
0.00
0.05
0.10
MR Method
IVW
MR Egger
Weighted Median
Inverse of SE (outcome)
4
8
12
16

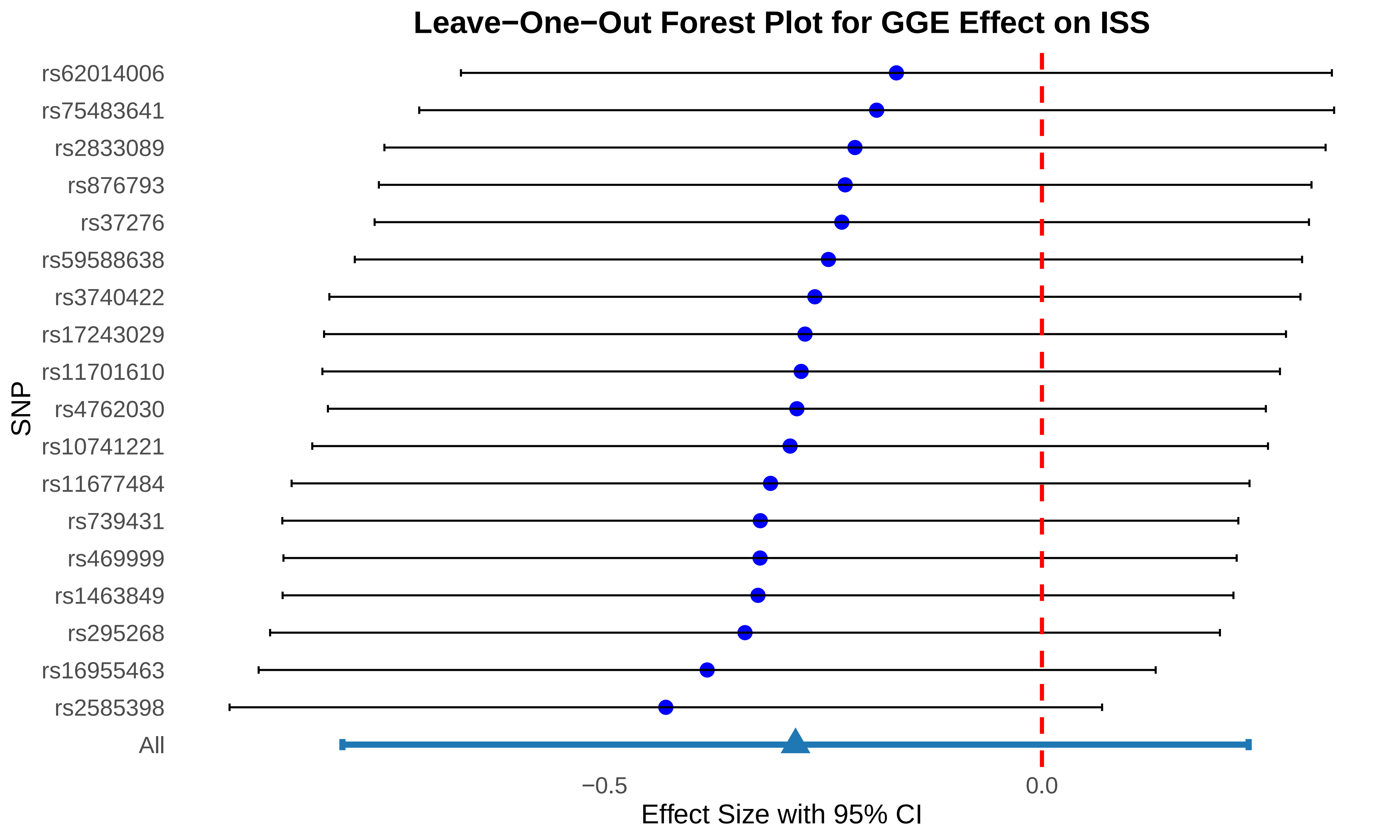

Leave−One−Out Forest Plot for GGE Effect on ISS
rs62014006
rs75483641
rs2833089
rs876793
rs37276
rs59588638
rs3740422
rs17243029
rs11701610
rs4762030
rs10741221
rs11677484
rs739431
rs469999
rs1463849
rs295268
rs16955463
rs2585398
All
SNP
−0.5
0.0
Effect Size with 95% CI

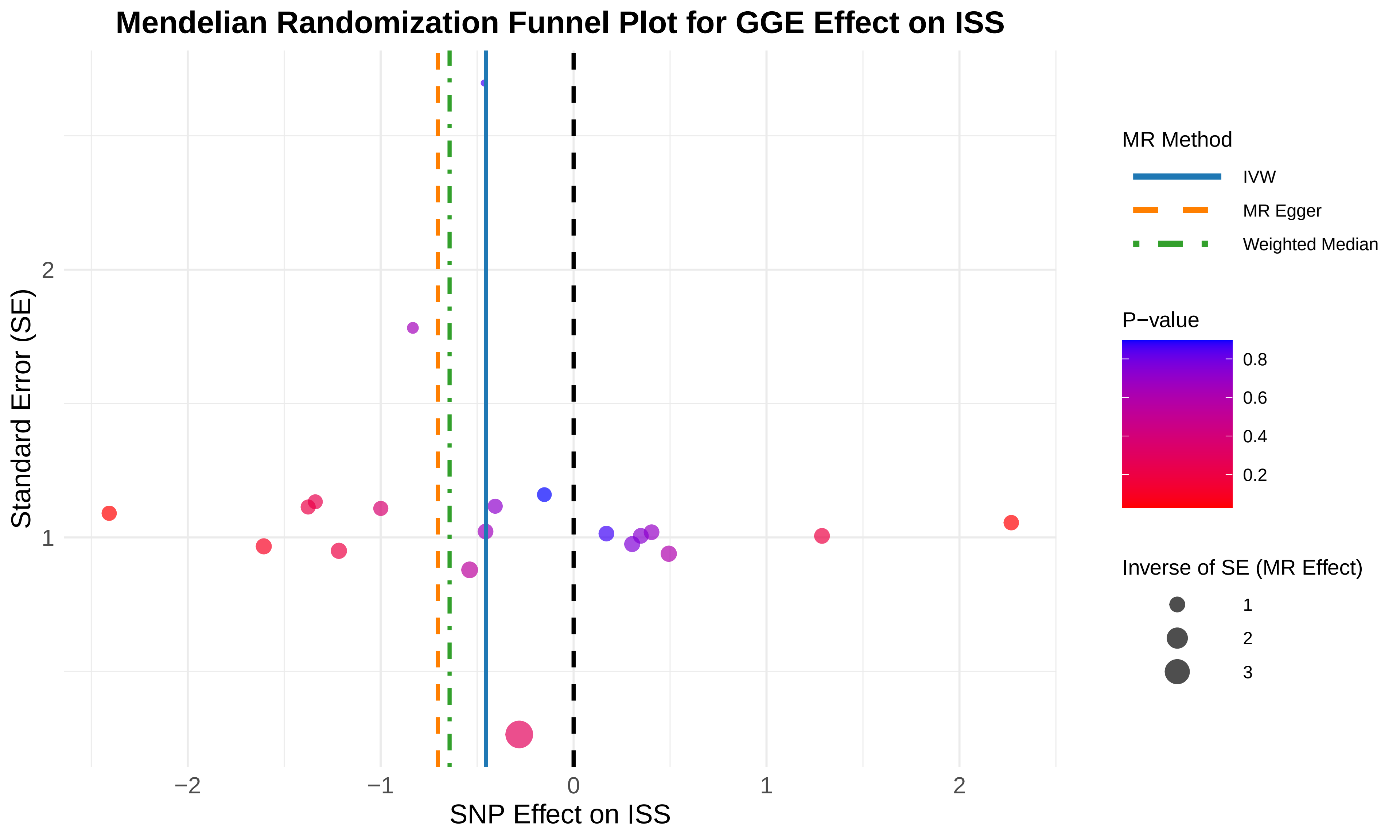
Mendelian Randomization Funnel Plot for GGE Effect on ISS
Standard Error (SE)
2
1
−2
−1
0
1
2
SNP Effect on ISS
MR Method
IVW
MR Egger
Weighted Median
P−value
0.8
0.6
0.4
0.2
Inverse of SE (MR Effect)
1
2
3

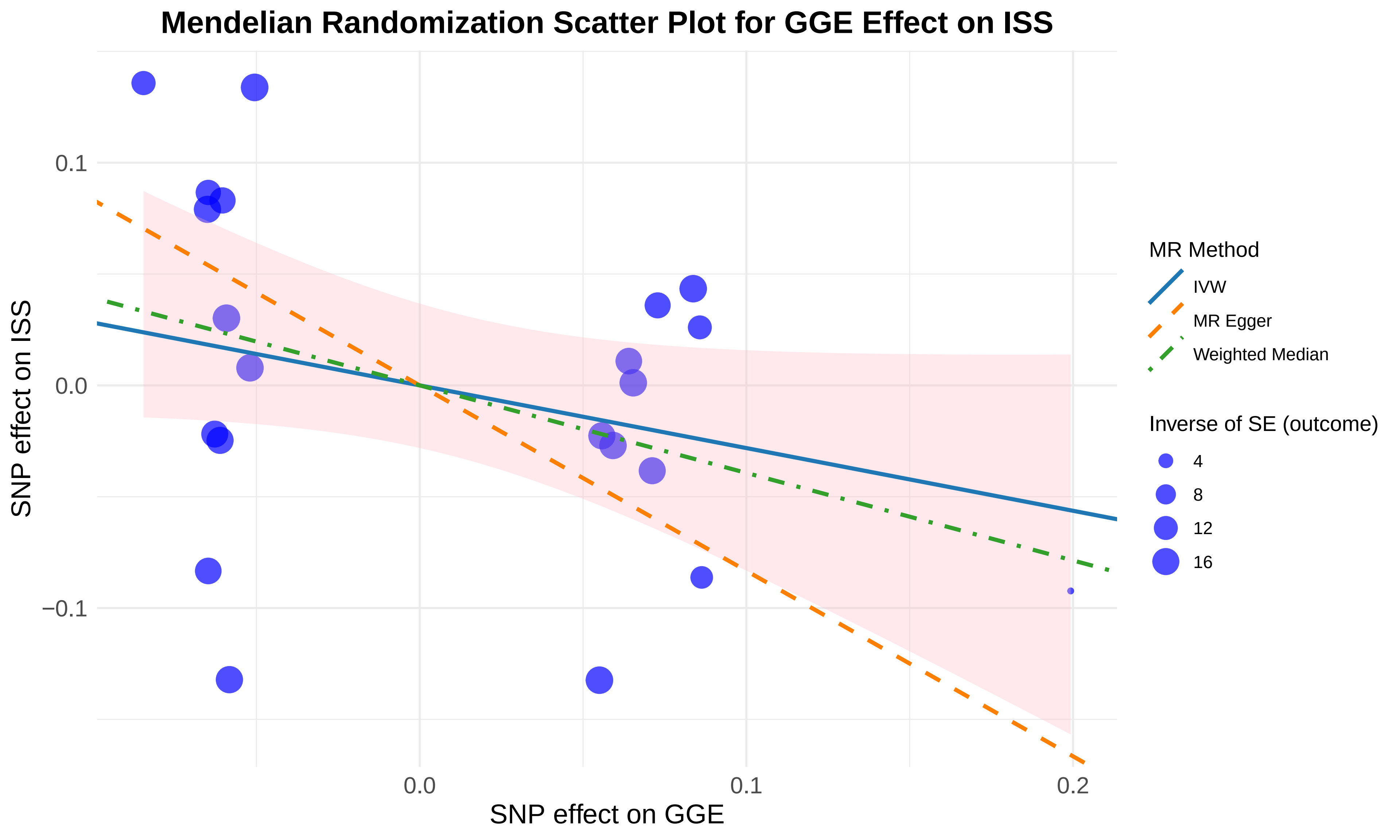

Mendelian Randomization Scatter Plot for GGE Effect on ISS
SNP effect on ISS
0.1
0.0
−0.1
0.0
0.1
0.2
SNP effect on GGE
MR Method
IVW
MR Egger
Weighted Median
Inverse of SE (outcome)
4
8
12
16

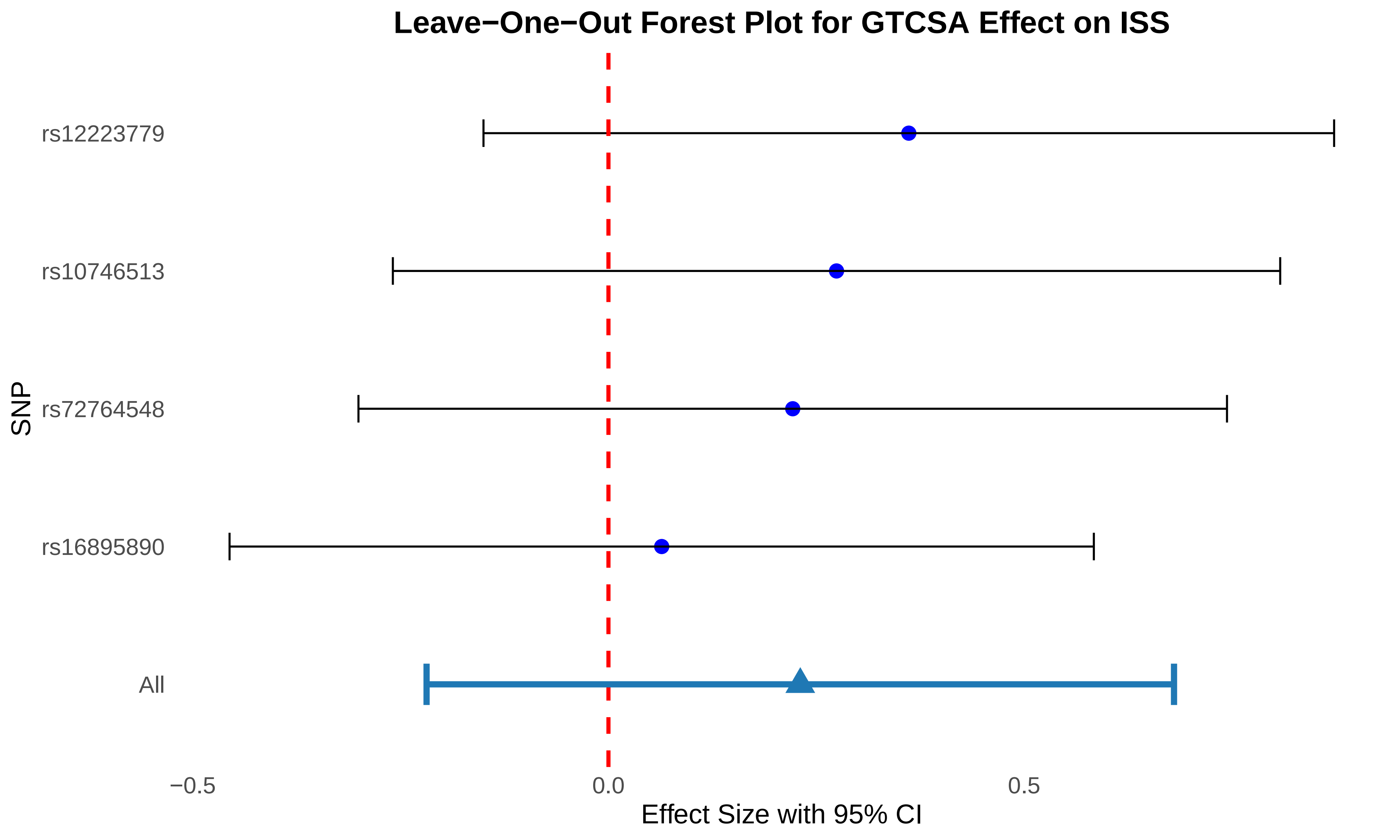
Leave−One−Out Forest Plot for GTCSA Effect on ISS
rs12223779
rs10746513
rs72764548
rs16895890
All
SNP
−0.5
0.0
0.5
Effect Size with 95% CI

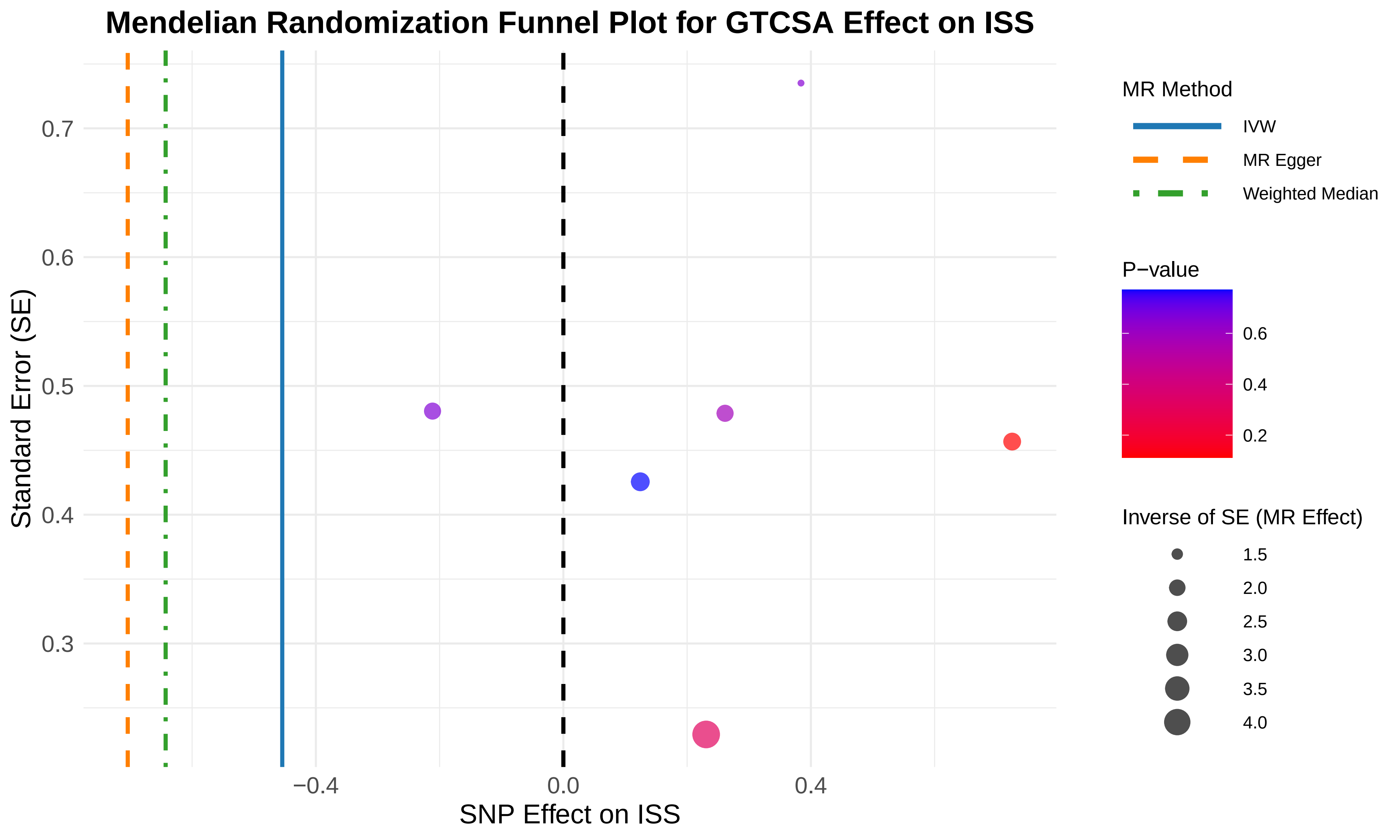

Mendelian Randomization Funnel Plot for GTCSA Effect on ISS
Standard Error (SE)
0.7
0.6
0.5
0.4
0.3
−0.4
0.0
0.4
SNP Effect on ISS
MR Method
IVW
MR Egger
Weighted Median
P−value
0.6
0.4
0.2
Inverse of SE (MR Effect)
1.5
2.0
2.5
3.0
3.5
4.0

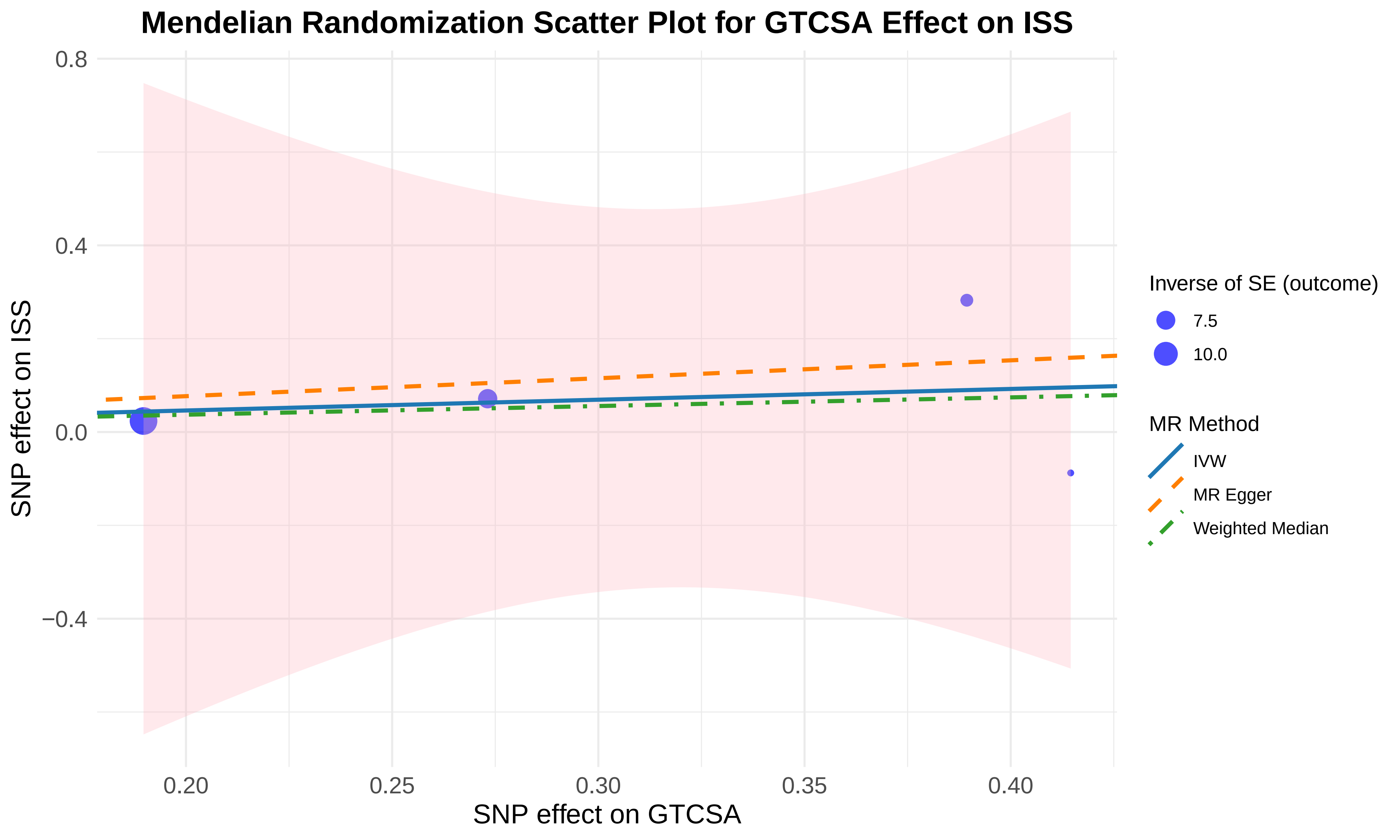
Mendelian Randomization Scatter Plot for GTCSA Effect on ISS
0.8
0.4
0.0
−0.4
SNP effect on ISS
0.20
0.25
0.30
0.35
0.40
SNP effect on GTCSA
Inverse of SE (outcome)
7.5
10.0
MR Method
IVW
MR Egger
Weighted Median

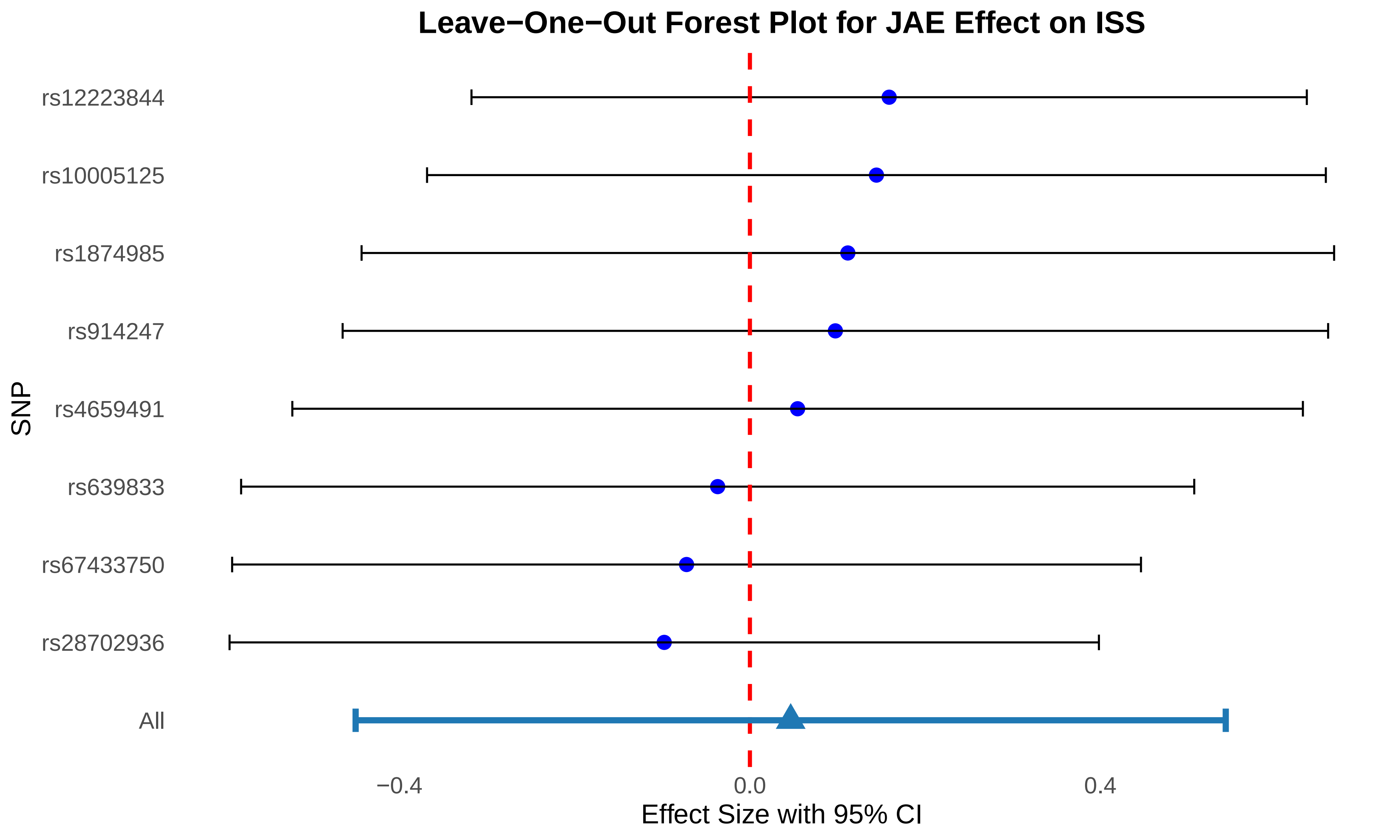
Leave−One−Out Forest Plot for JAE Effect on ISS
SNP
rs12223844
rs10005125
rs1874985
rs914247
rs4659491
rs639833
rs67433750
rs28702936
All
−0.4
0.0
0.4
Effect Size with 95% CI

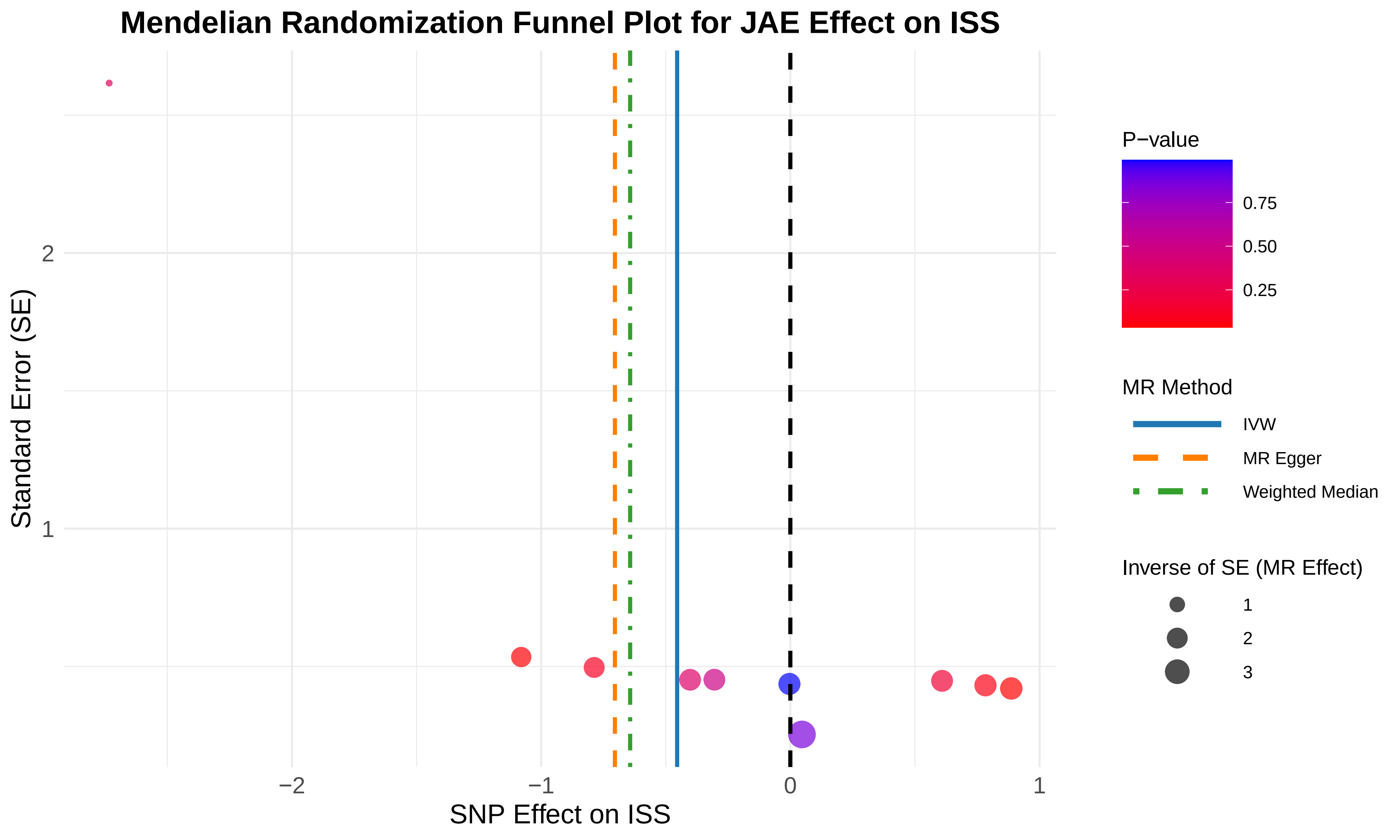
Mendelian Randomization Funnel Plot for JAE Effect on ISS
Standard Error (SE)
SNP Effect on ISS
2
1
−2
−1
0
1
P−value
0.75
0.50
0.25
MR Method
IVW
MR Egger
Weighted Median
Inverse of SE (MR Effect)
1
2
3

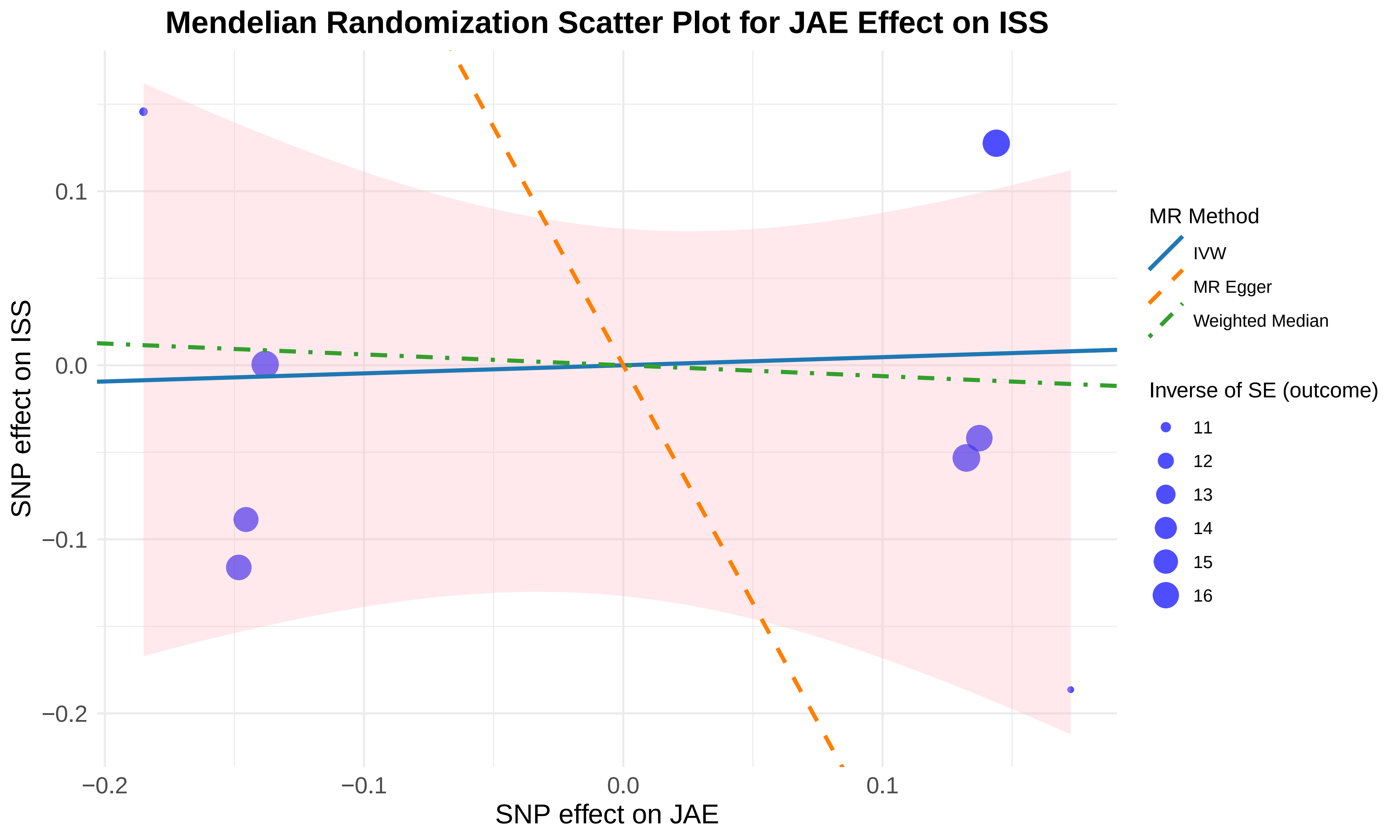

Mendelian Randomization Scatter Plot for JAE Effect on ISS
SNP effect on ISS
0.1
0.0
−0.1
−0.2
−0.2
−0.1
0.0
0.1
SNP effect on JAE
MR Method
IVW
MR Egger
Weighted Median
Inverse of SE (outcome)
11
12
13
14
15
16

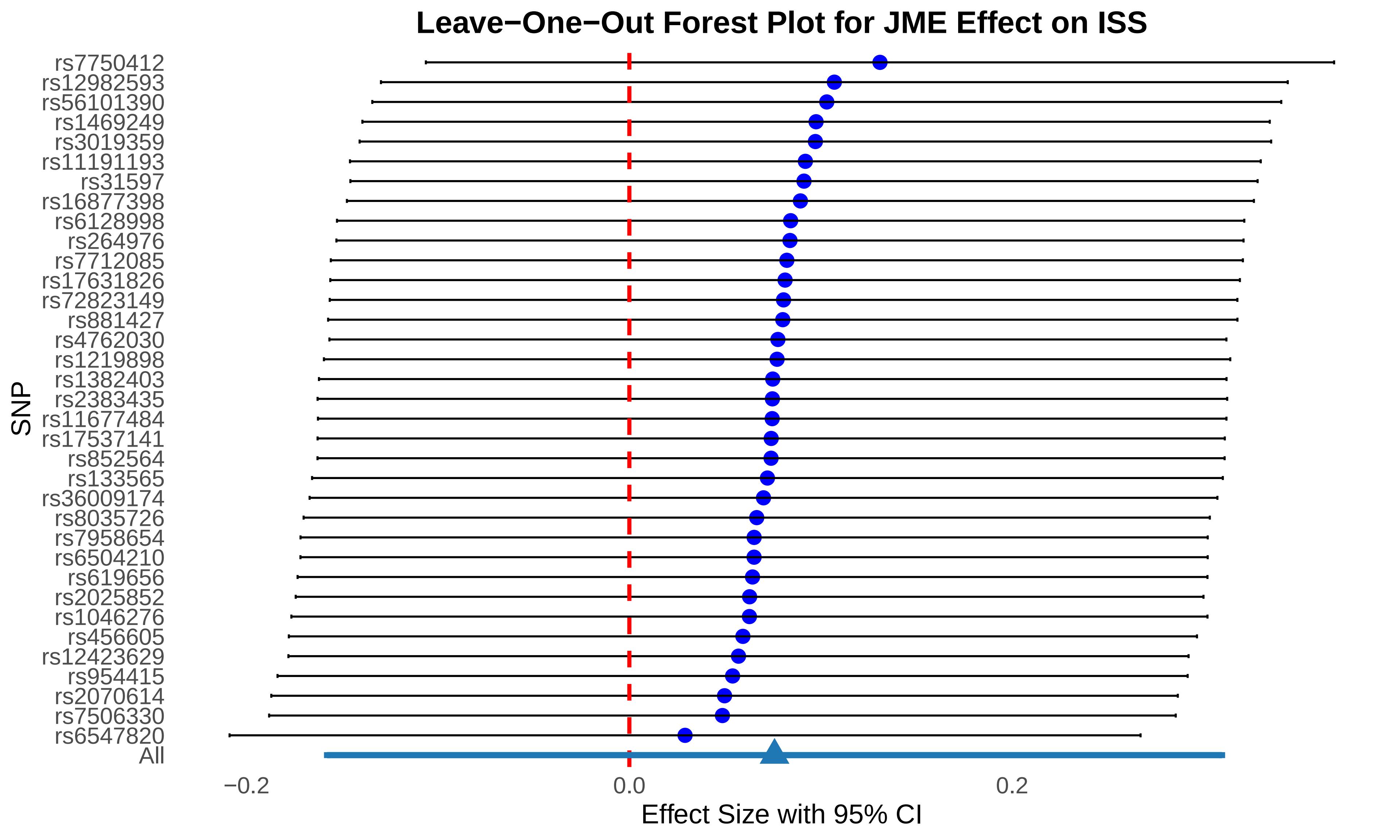

Leave−One−Out Forest Plot for JME Effect on ISS
SNP
rs7750412
rs12982593
rs56101390
rs1469249
rs3019359
rs11191193
rs31597
rs16877398
rs6128998
rs264976
rs7712085
rs17631826
rs72823149
rs881427
rs4762030
rs1219898
rs1382403
rs2383435
rs11677484
rs17537141
rs852564
rs133565
rs36009174
rs8035726
rs7958654
rs6504210
rs619656
rs2025852
rs1046276
rs456605
rs12423629
rs954415
rs2070614
rs7506330
rs6547820
All
−0.2
0.0
0.2
Effect Size with 95% CI

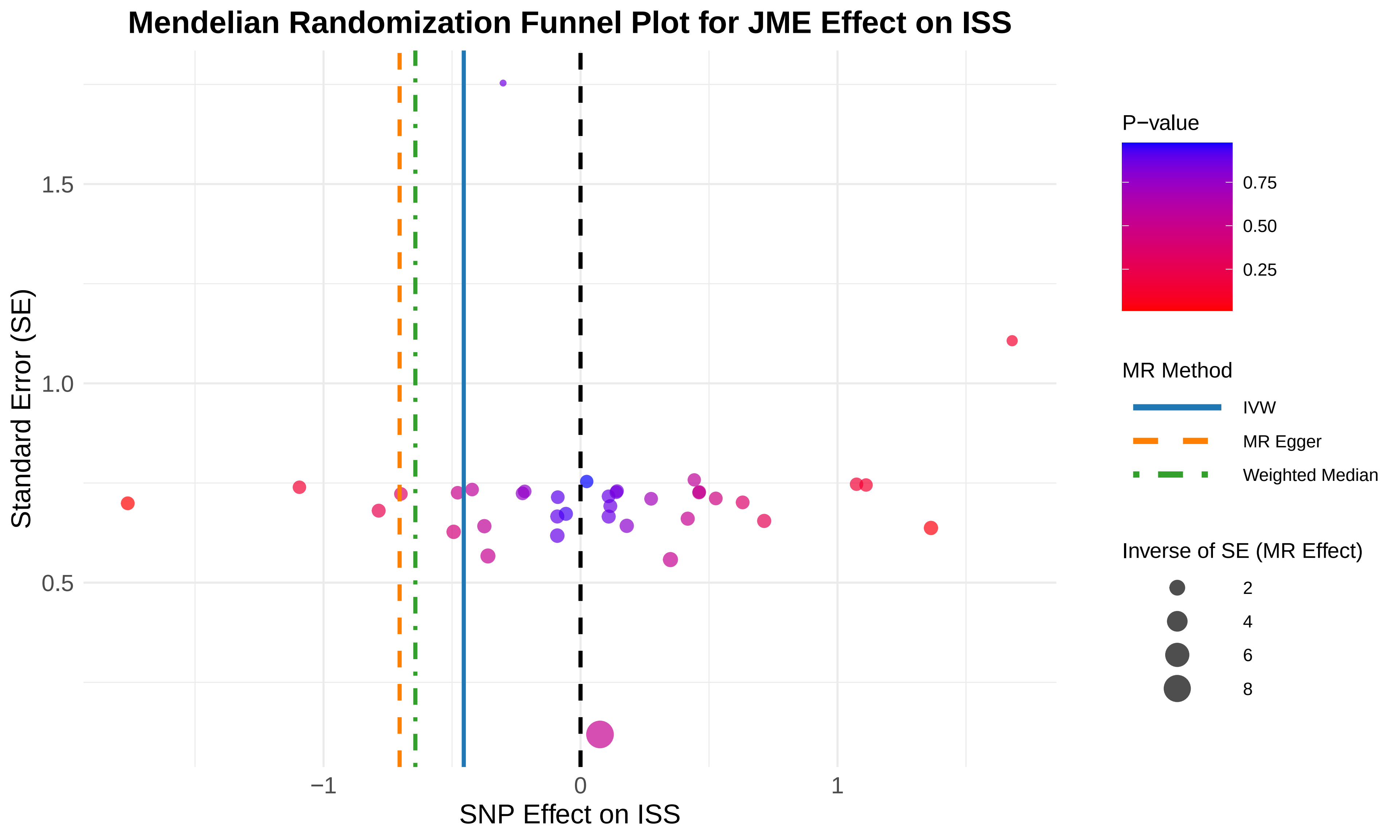

Mendelian Randomization Funnel Plot for JME Effect on ISS
Standard Error (SE)
1.5
1.0
0.5
−1
0
1
SNP Effect on ISS
P−value
0.75
0.50
0.25
MR Method
IVW
MR Egger
Weighted Median
Inverse of SE (MR Effect)
2
4
6
8

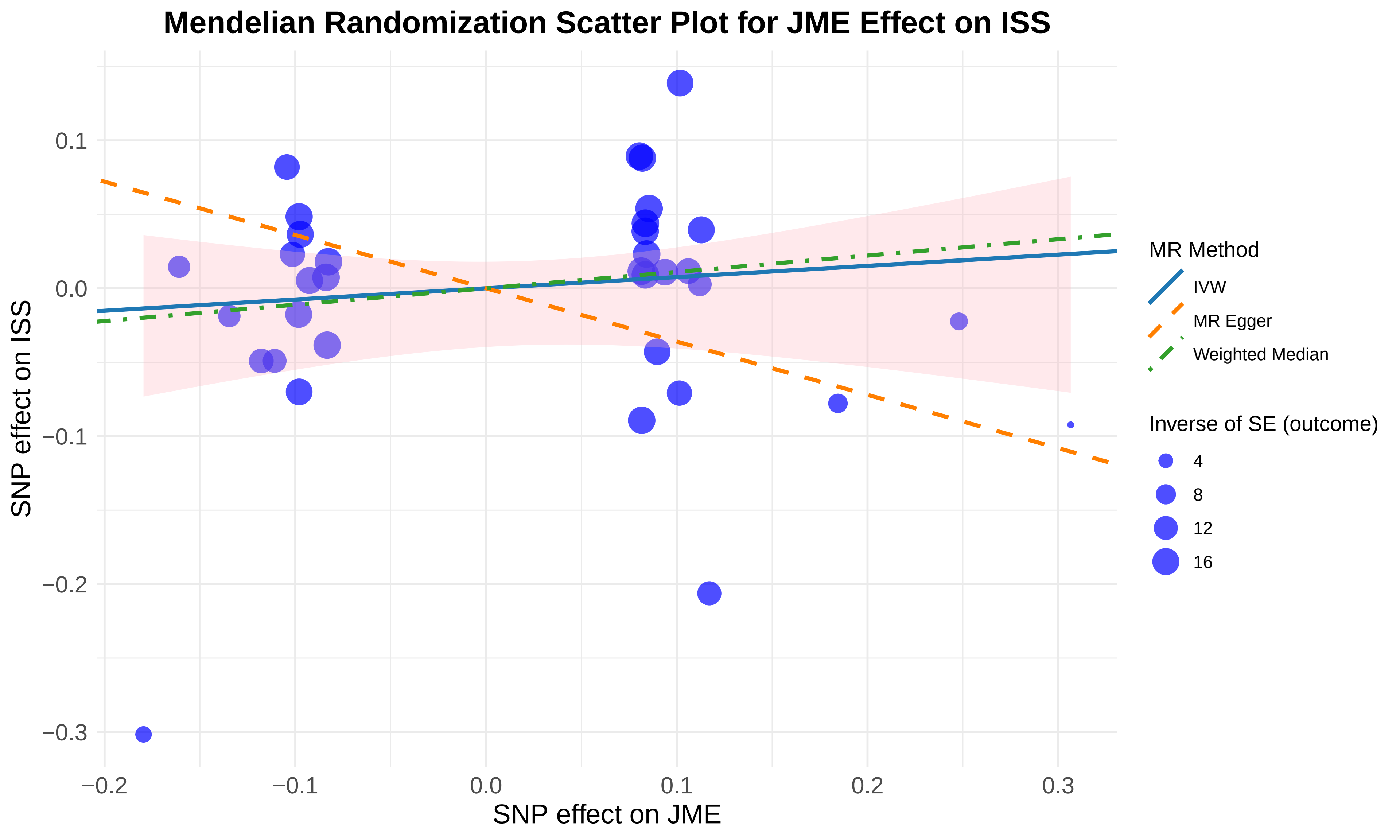
Mendelian Randomization Scatter Plot for JME Effect on ISS
SNP effect on ISS
SNP effect on JME
0.1
0.0
−0.1
−0.2
−0.3
−0.2
−0.1
0.0
0.1
0.2
0.3
MR Method
IVW
MR Egger
Weighted Median
Inverse of SE (outcome)
4
8
12
16

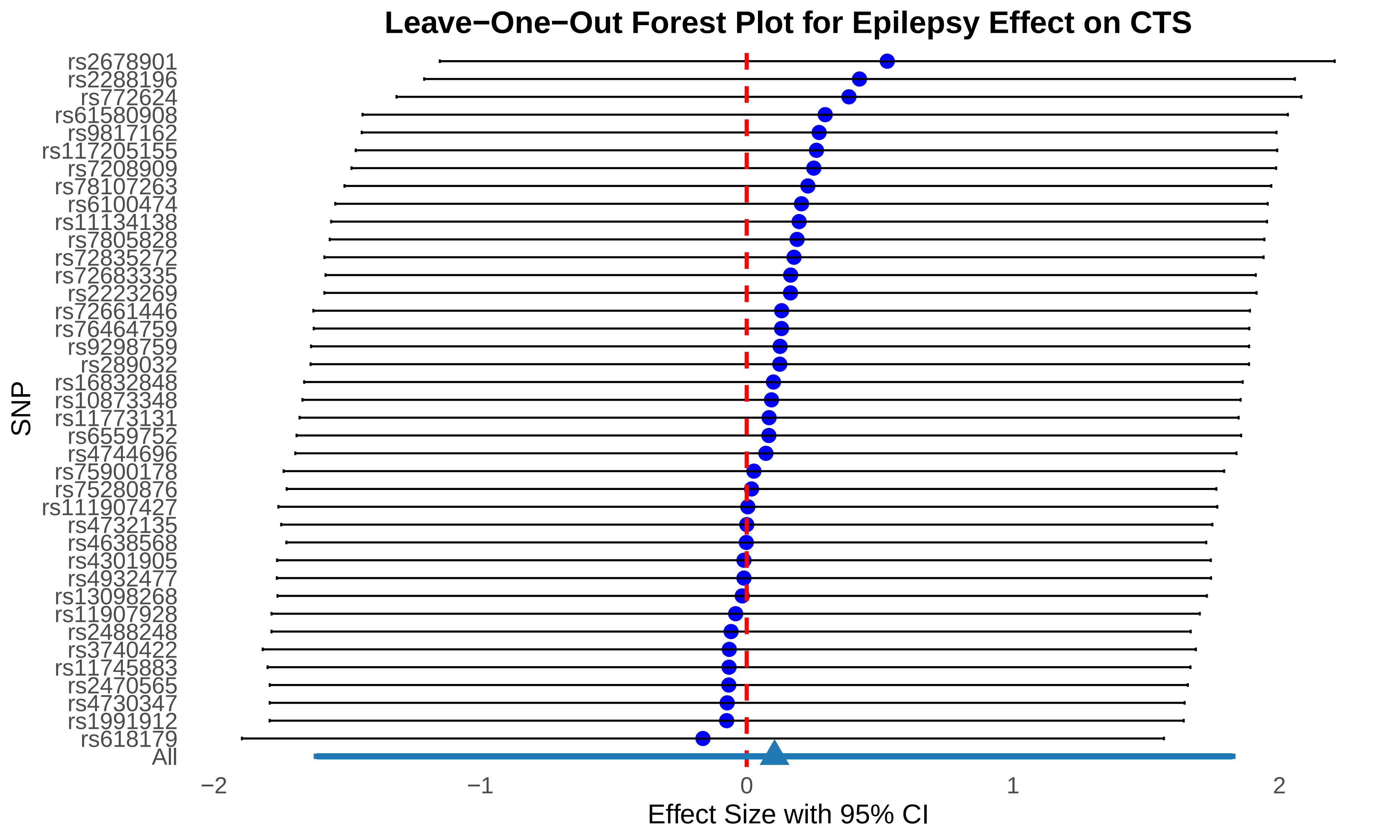

Leave−One−Out Forest Plot for Epilepsy Effect on CTS
SNP
rs2678901
rs2288196
rs772624
rs61580908
rs9817162
rs117205155
rs7208909
rs78107263
rs6100474
rs11134138
rs7805828
rs72835272
rs72683335
rs2223269
rs72661446
rs76464759
rs9298759
rs289032
rs16832848
rs10873348
rs11773131
rs6559752
rs4744696
rs75900178
rs75280876
rs111907427
rs4732135
rs4638568
rs4301905
rs4932477
rs13098268
rs11907928
rs2488248
rs3740422
rs11745883
rs2470565
rs4730347
rs1991912
rs618179
All
−2
−1
0
1
2
Effect Size with 95% CI

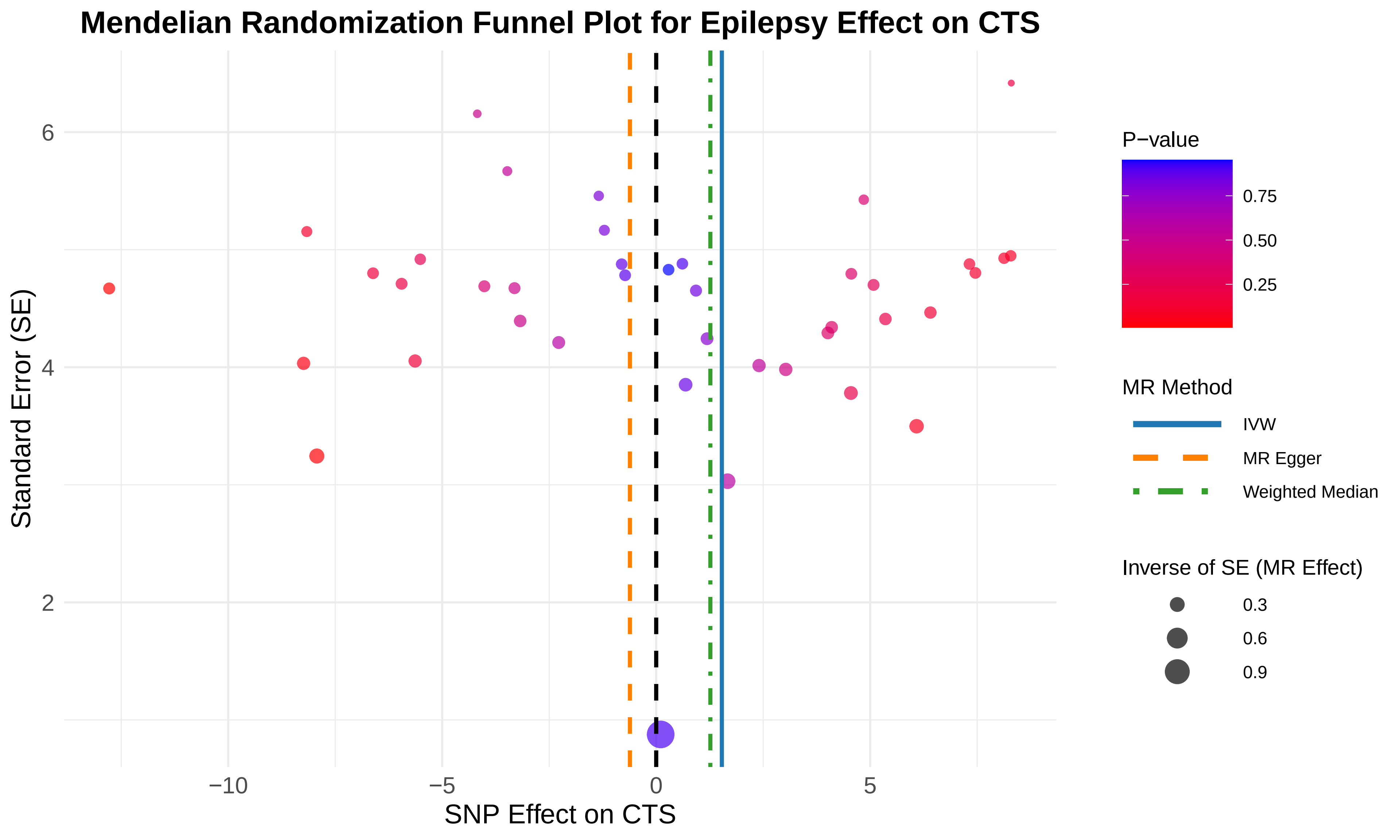
Mendelian Randomization Funnel Plot for Epilepsy Effect on CTS
Standard Error (SE)
SNP Effect on CTS
6
4
2
−10
−5
0
5
P−value
0.75
0.50
0.25
MR Method
IVW
MR Egger
Weighted Median
Inverse of SE (MR Effect)
0.3
0.6
0.9

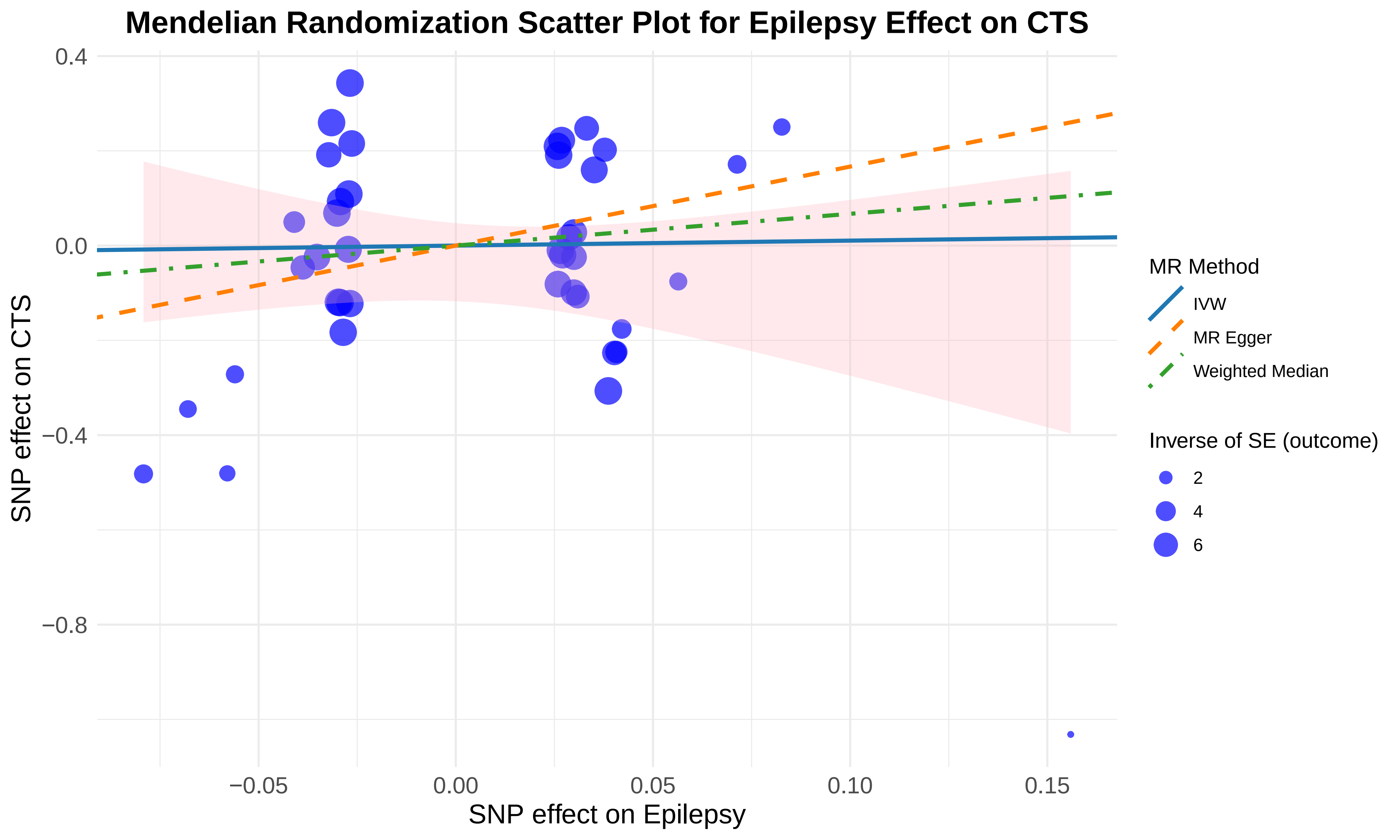

Mendelian Randomization Scatter Plot for Epilepsy Effect on CTS
0.4
0.0
−0.4
−0.8
−0.05
0.00
0.05
0.10
0.15
SNP effect on CTS
SNP effect on Epilepsy
MR Method
IVW
MR Egger
Weighted Median
Inverse of SE (outcome)
2
4
6

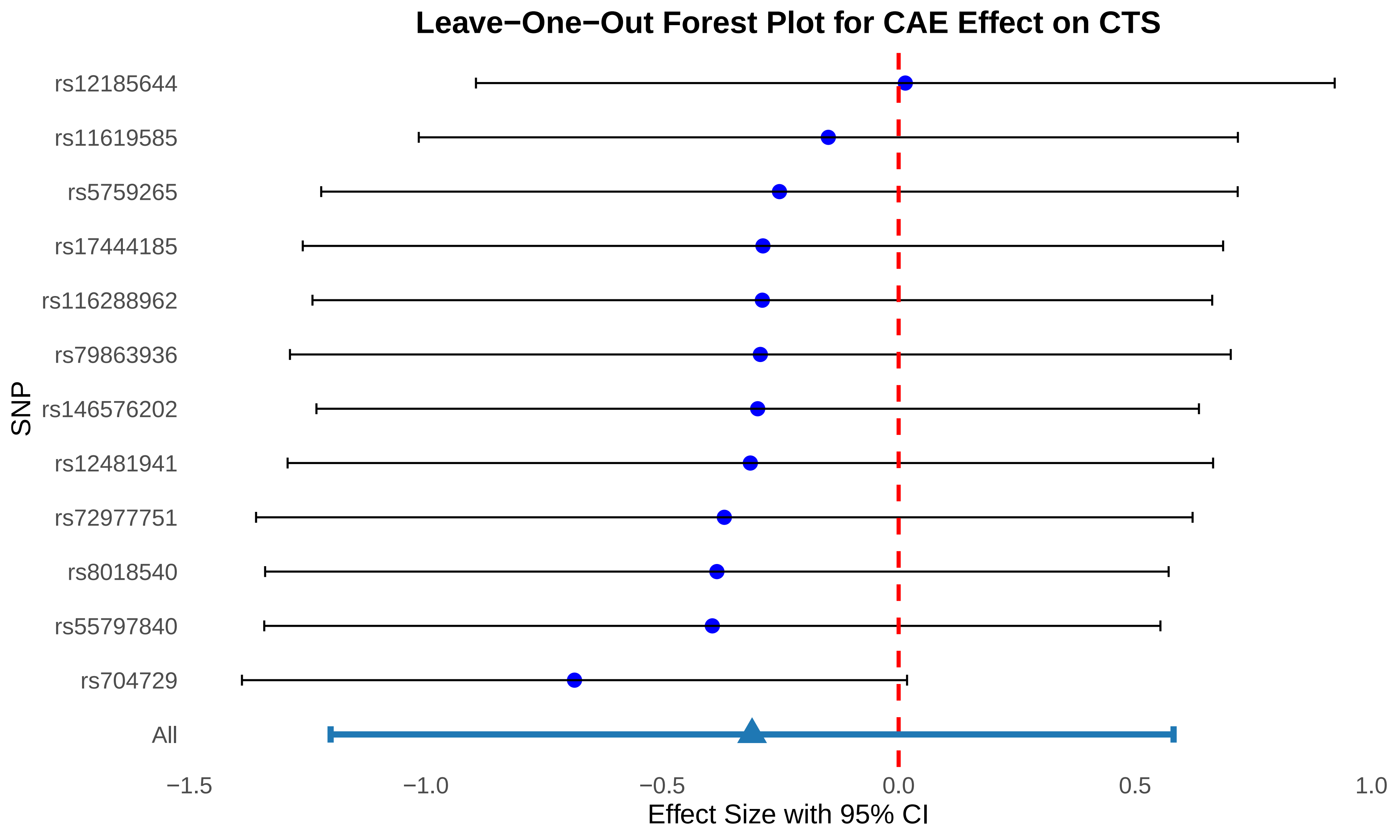
Leave−One−Out Forest Plot for CAE Effect on CTS
SNP
rs12185644
rs11619585
rs5759265
rs17444185
rs116288962
rs79863936
rs146576202
rs12481941
rs72977751
rs8018540
rs55797840
rs704729
All
−1.5
−1.0
−0.5
0.0
0.5
1.0
Effect Size with 95% CI

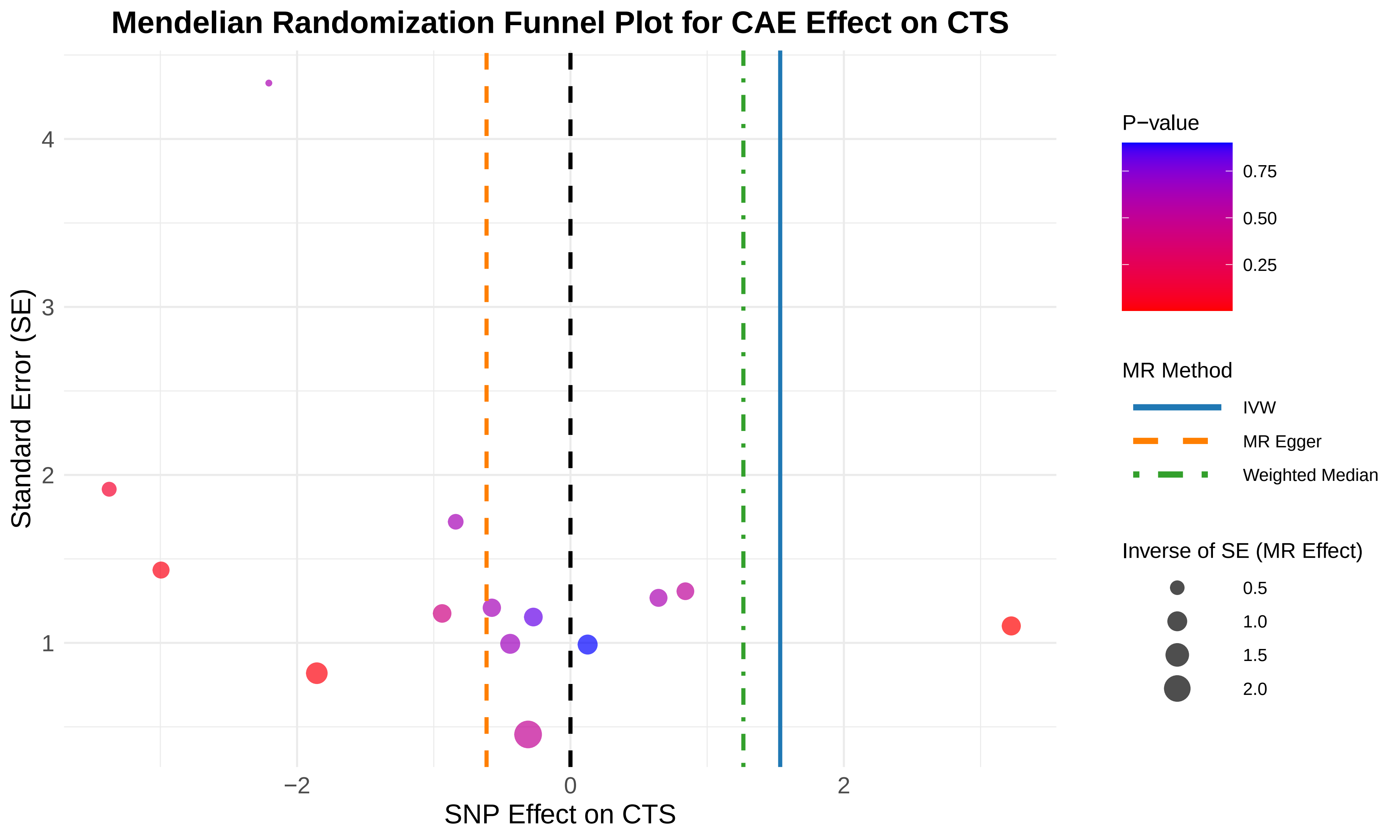

Mendelian Randomization Funnel Plot for CAE Effect on CTS
Standard Error (SE)
SNP Effect on CTS
4
3
2
1
−2
0
2
P−value
0.75
0.50
0.25
MR Method
IVW
MR Egger
Weighted Median
Inverse of SE (MR Effect)
0.5
1.0
1.5
2.0

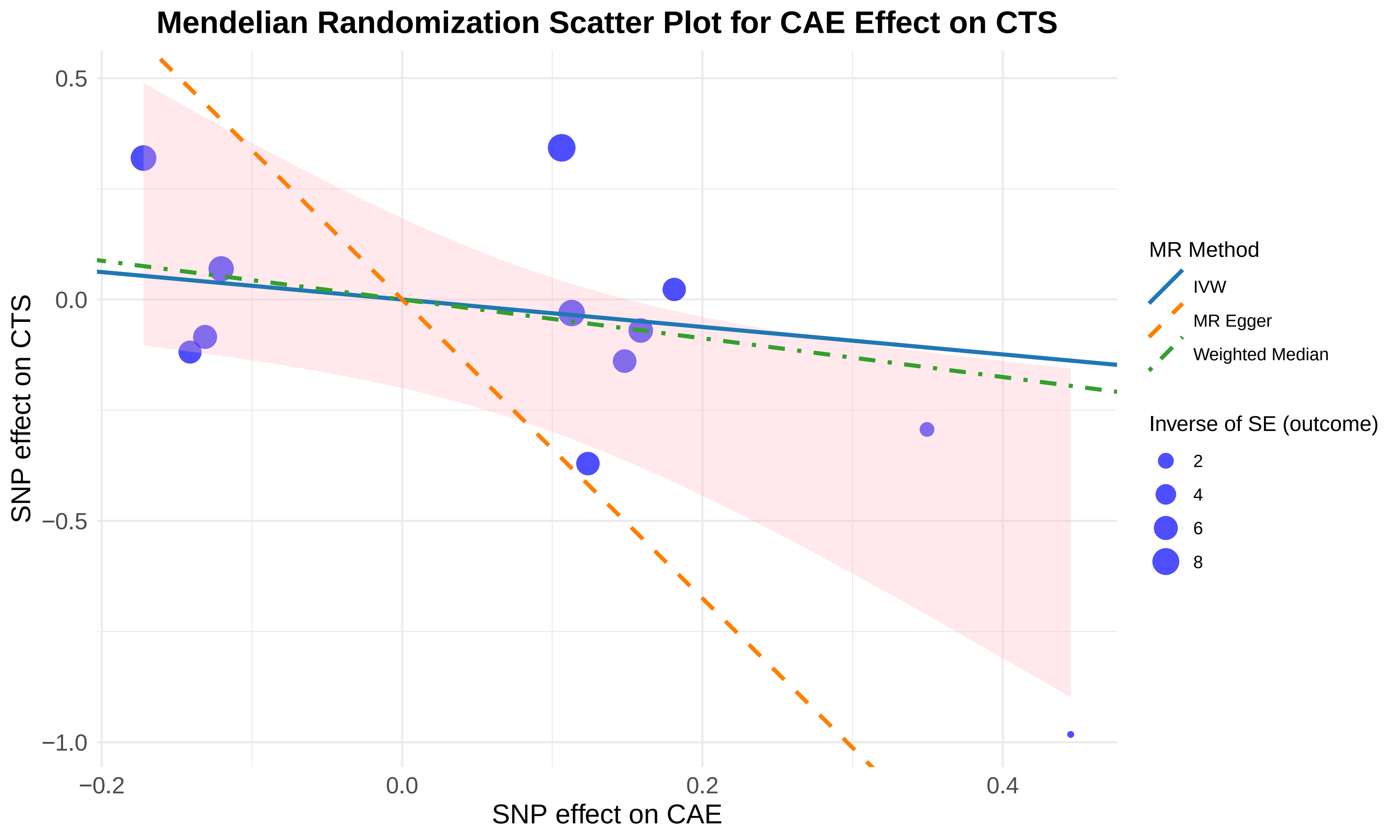

Mendelian Randomization Scatter Plot for CAE Effect on CTS
0.5
0.0
−0.5
−1.0
SNP effect on CTS
−0.2
0.0
0.2
0.4
SNP effect on CAE
MR Method
IVW
MR Egger
Weighted Median
Inverse of SE (outcome)
2
4
6
8

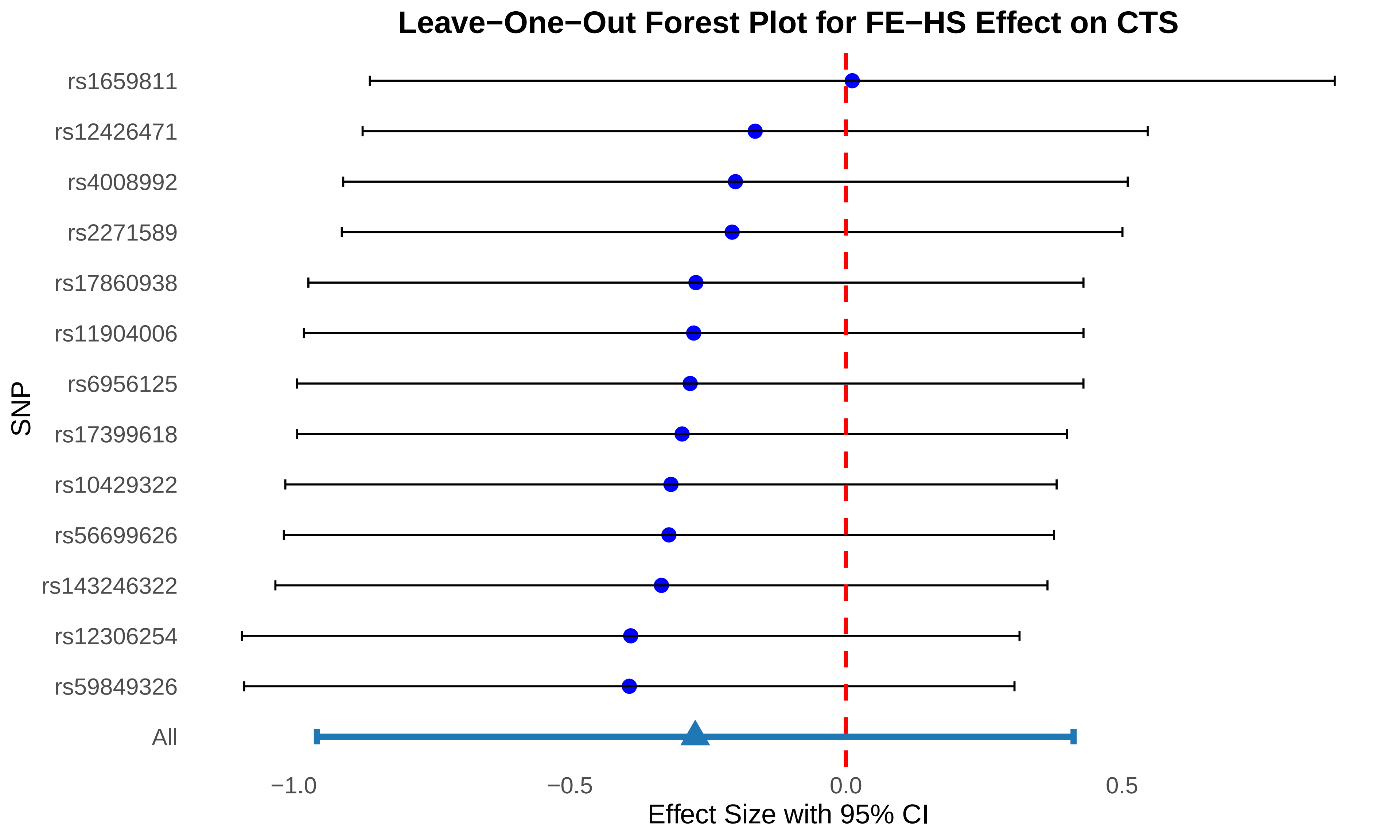

Leave−One−Out Forest Plot for FE−HS Effect on CTS
SNP
rs1659811
rs12426471
rs4008992
rs2271589
rs17860938
rs11904006
rs6956125
rs17399618
rs10429322
rs56699626
rs143246322
rs12306254
rs59849326
All
−1.0
−0.5
0.0
0.5
Effect Size with 95% CI

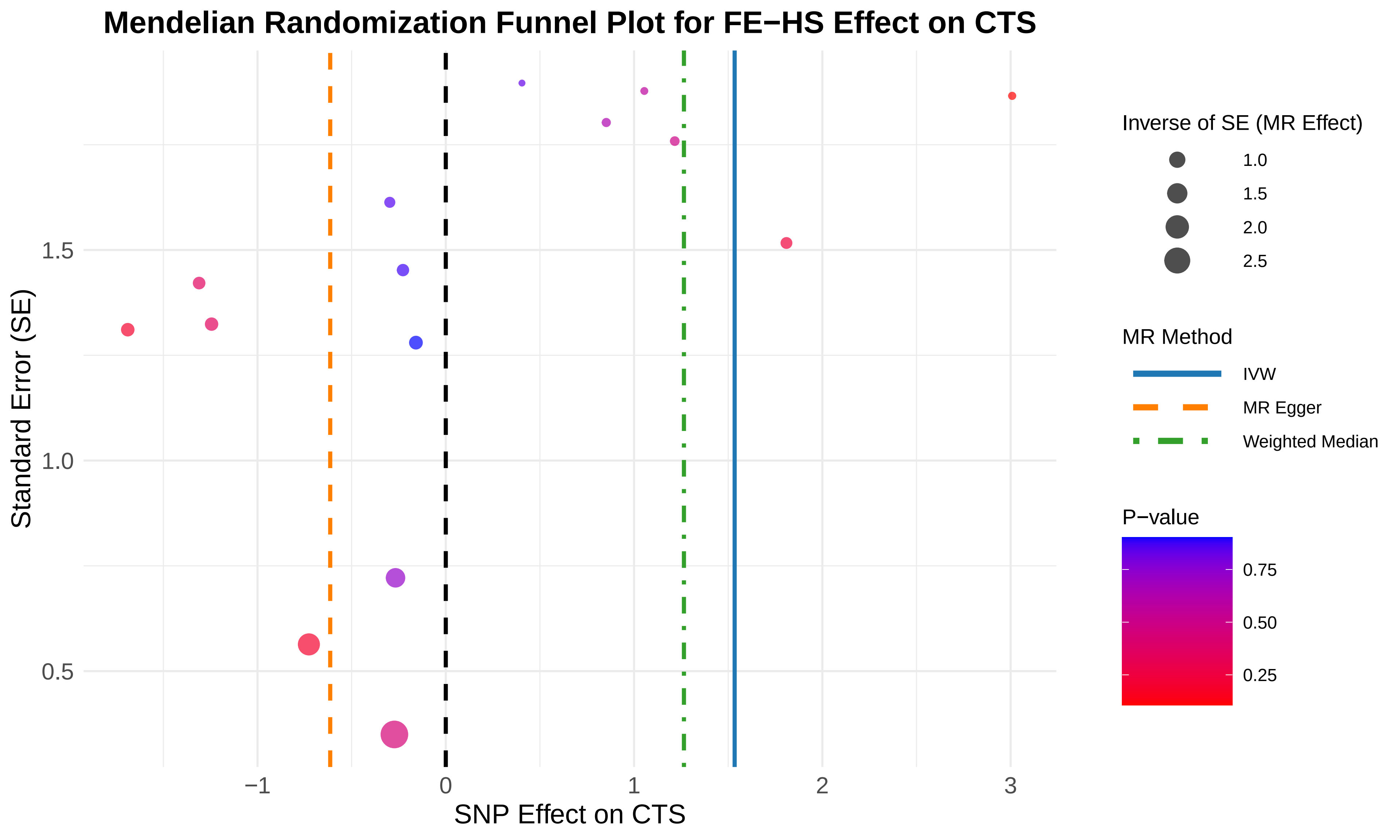

Mendelian Randomization Funnel Plot for FE−HS Effect on CTS
Standard Error (SE)
1.5
1.0
0.5
−1
0
1
2
3
SNP Effect on CTS
Inverse of SE (MR Effect)
1.0
1.5
2.0
2.5
MR Method
IVW
MR Egger
Weighted Median
P−value
0.75
0.50
0.25

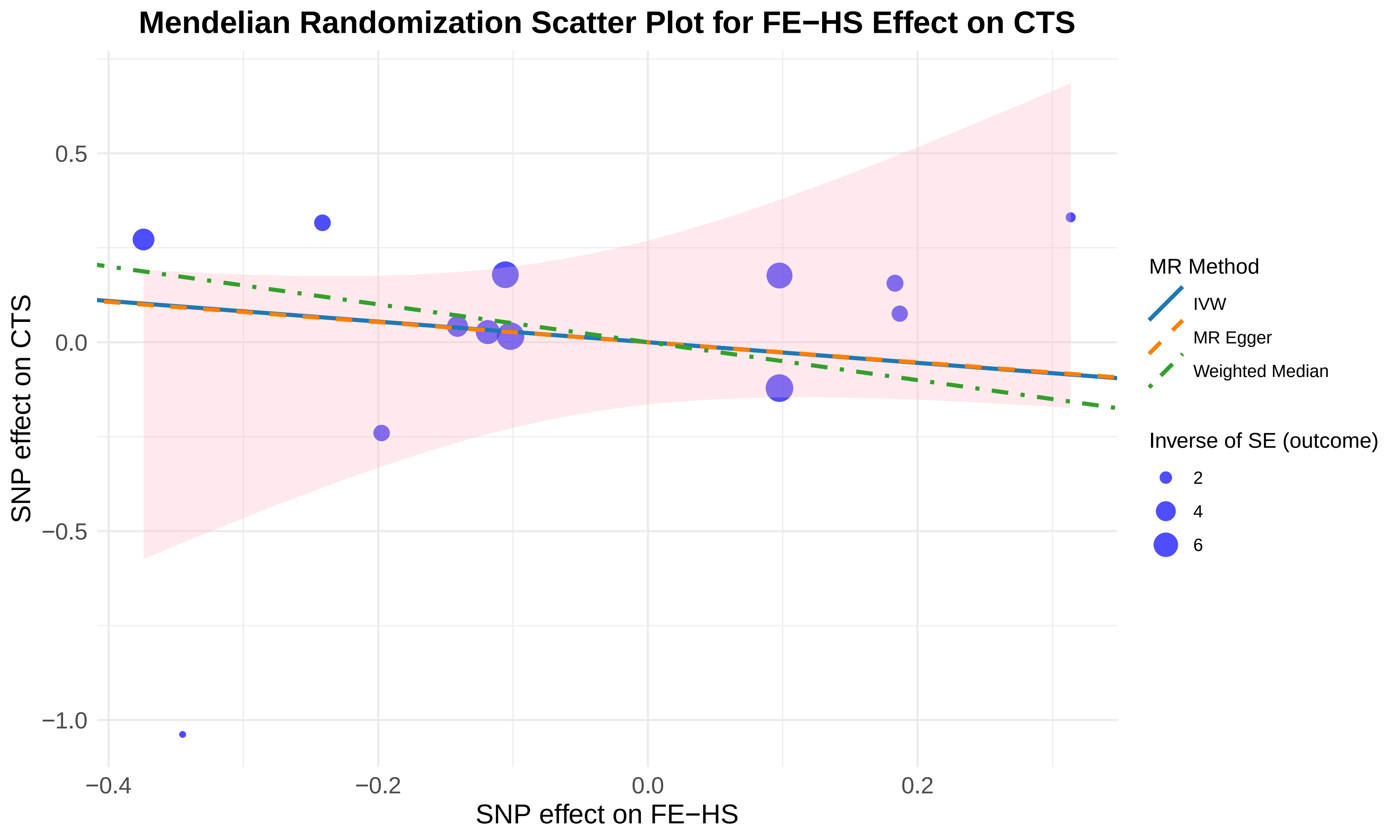
Mendelian Randomization Scatter Plot for FE−HS Effect on CTS
SNP effect on CTS
0.5
0.0
−0.5
−1.0
−0.4
−0.2
0.0
0.2
SNP effect on FE−HS
MR Method
IVW
MR Egger
Weighted Median
Inverse of SE (outcome)
2
4
6

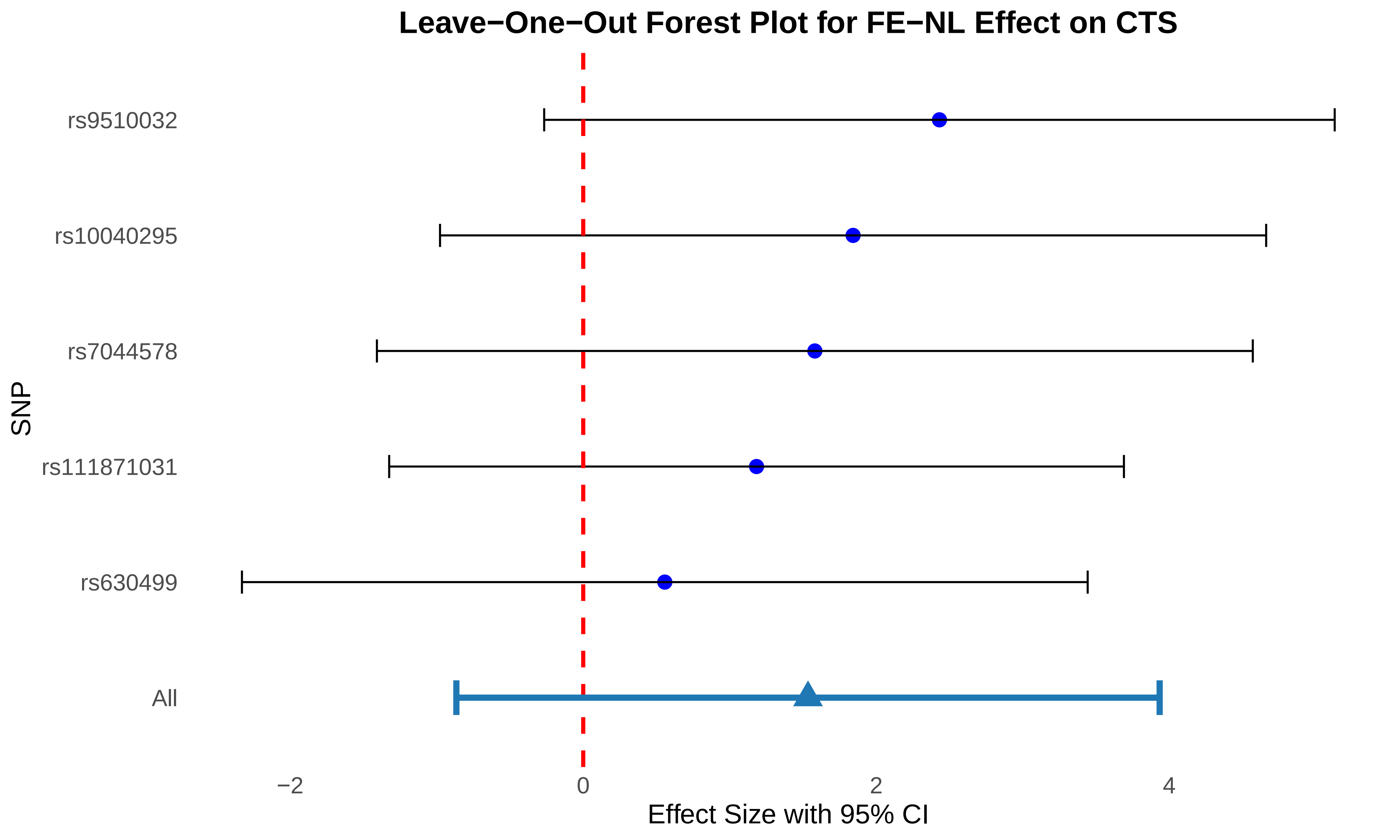

Leave−One−Out Forest Plot for FE−NL Effect on CTS
rs9510032
rs10040295
rs7044578
rs111871031
rs630499
All
SNP
−2
0
2
4
Effect Size with 95% CI

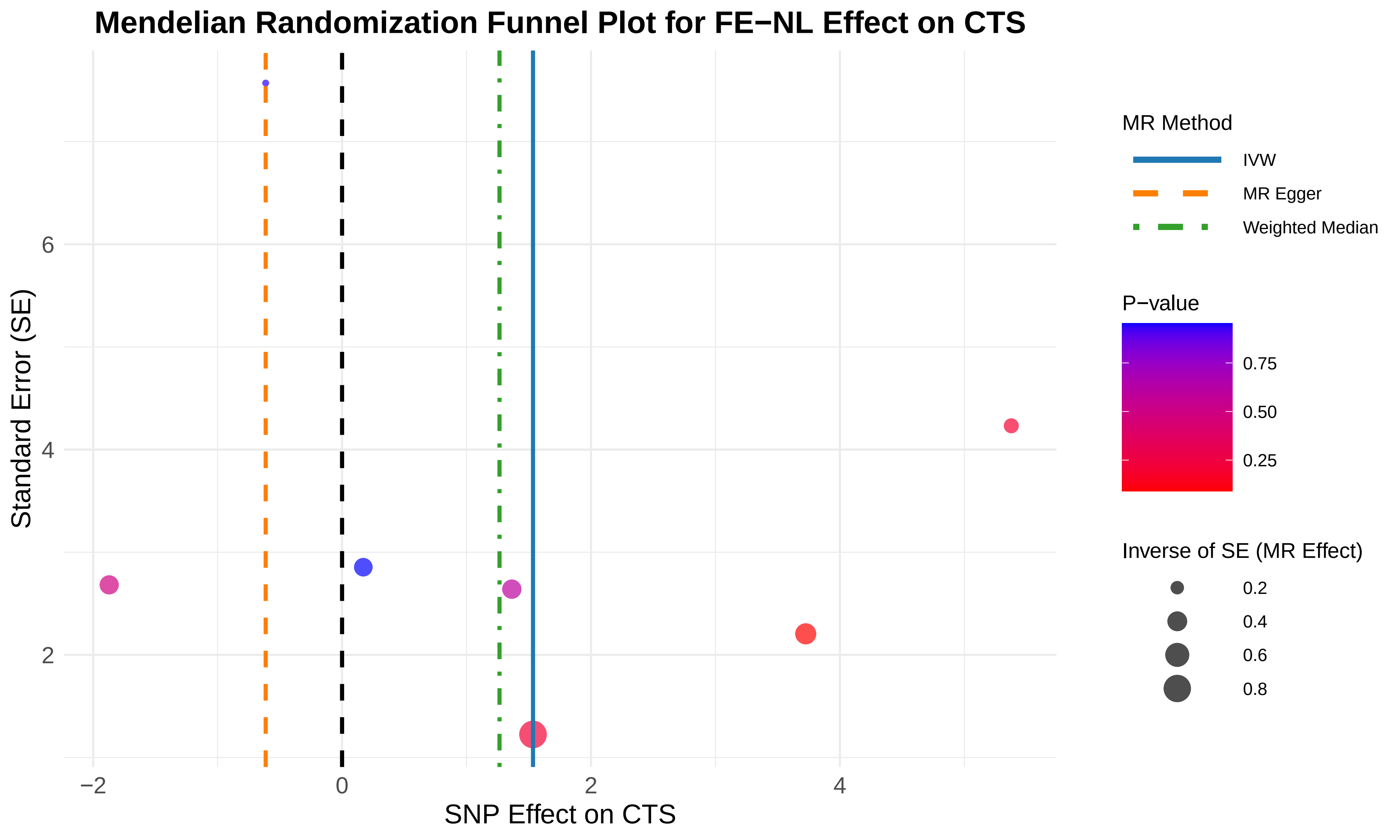
Mendelian Randomization Funnel Plot for FE−NL Effect on CTS
Standard Error (SE)
6
4
2
−2
0
2
4
SNP Effect on CTS
MR Method
IVW
MR Egger
Weighted Median
P−value
0.75
0.50
0.25
Inverse of SE (MR Effect)
0.2
0.4
0.6
0.8

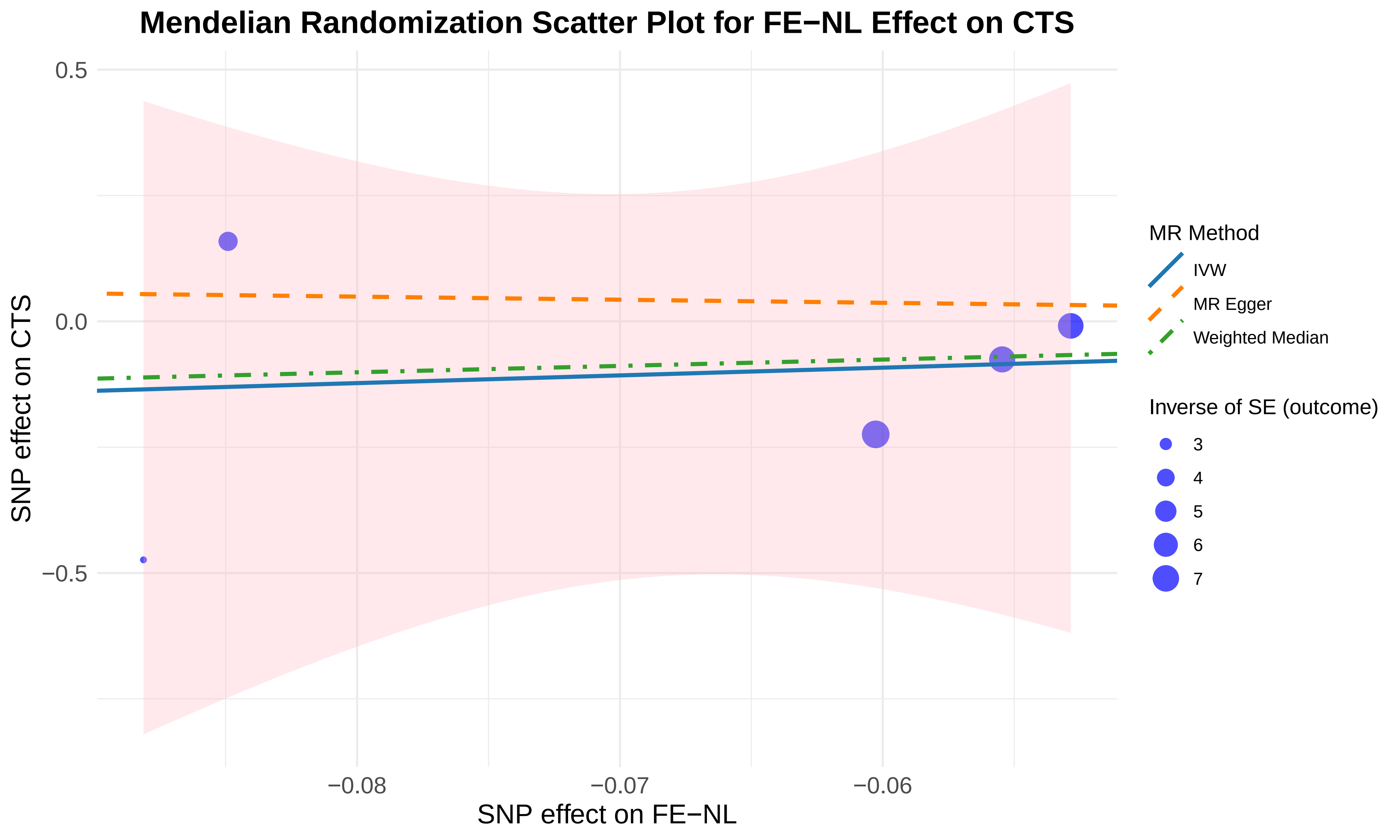

Mendelian Randomization Scatter Plot for FE−NL Effect on CTS
0.5
0.0
−0.5
SNP effect on CTS
−0.08
−0.07
−0.06
SNP effect on FE−NL
MR Method
IVW
MR Egger
Weighted Median
Inverse of SE (outcome)
3
4
5
6
7

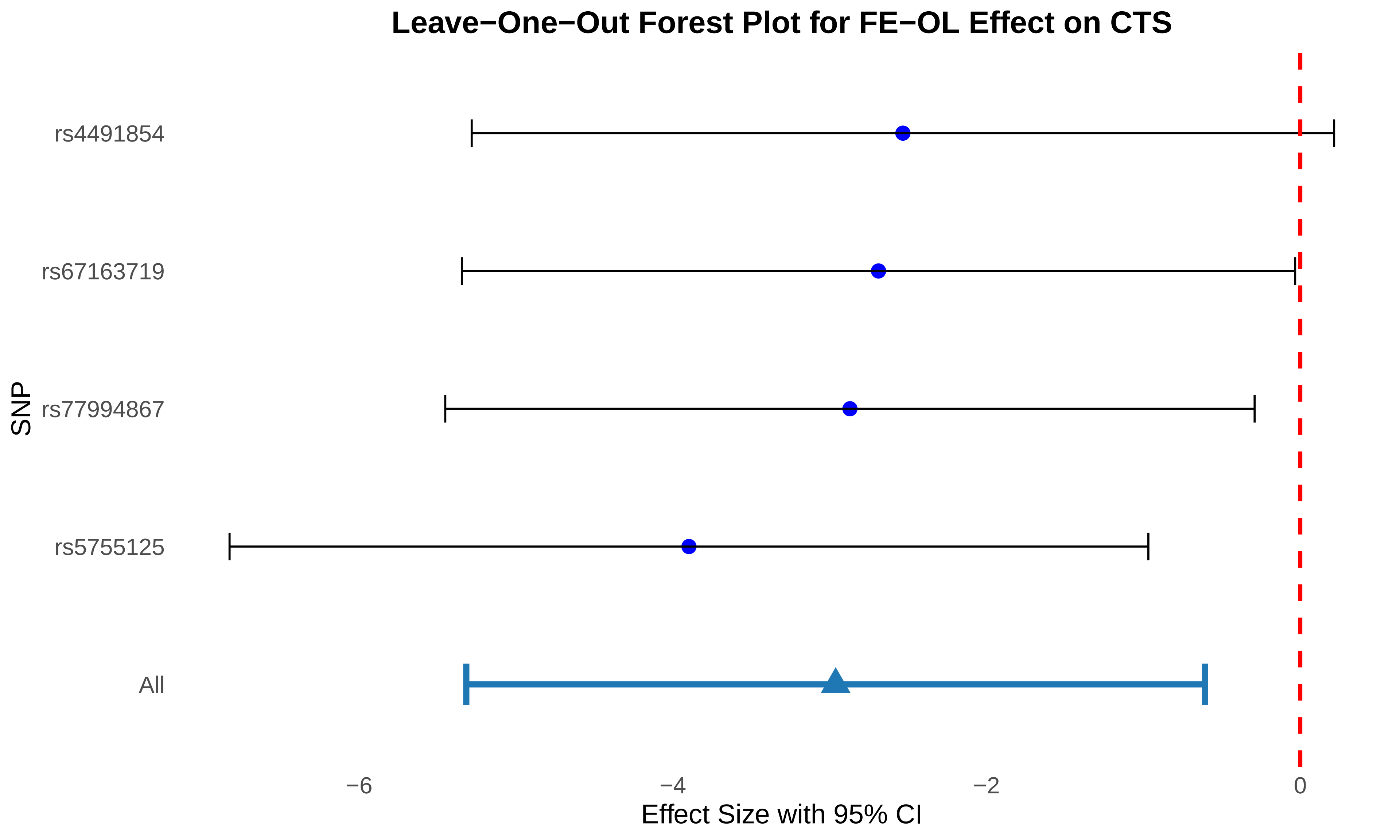
Leave−One−Out Forest Plot for FE−OL Effect on CTS
rs4491854
rs67163719
rs77994867
rs5755125
All
SNP
−6
−4
−2
0
Effect Size with 95% CI

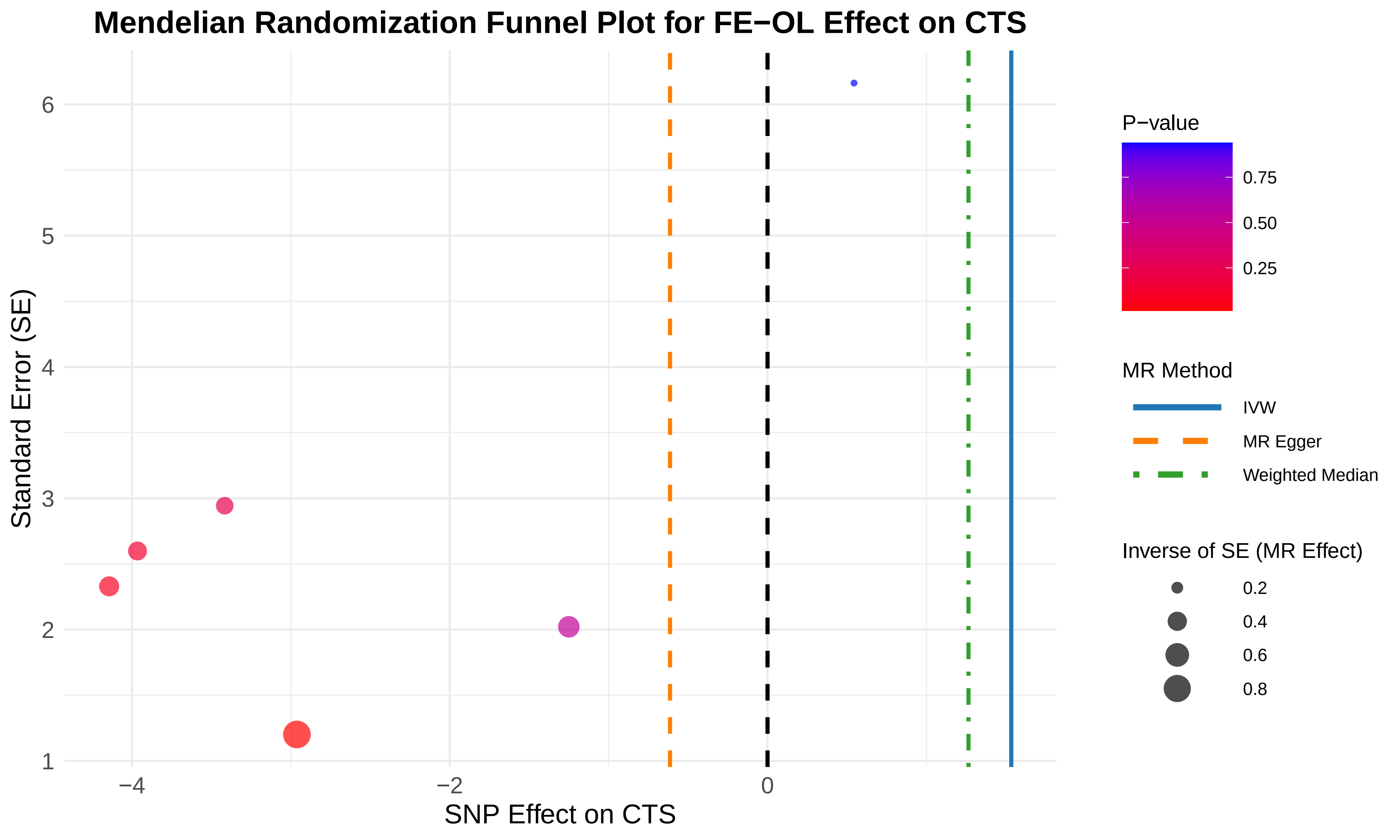

Mendelian Randomization Funnel Plot for FE−OL Effect on CTS
Standard Error (SE)
SNP Effect on CTS
6
5
4
3
2
1
−4
−2
0
P−value
0.75
0.50
0.25
MR Method
IVW
MR Egger
Weighted Median
Inverse of SE (MR Effect)
0.2
0.4
0.6
0.8

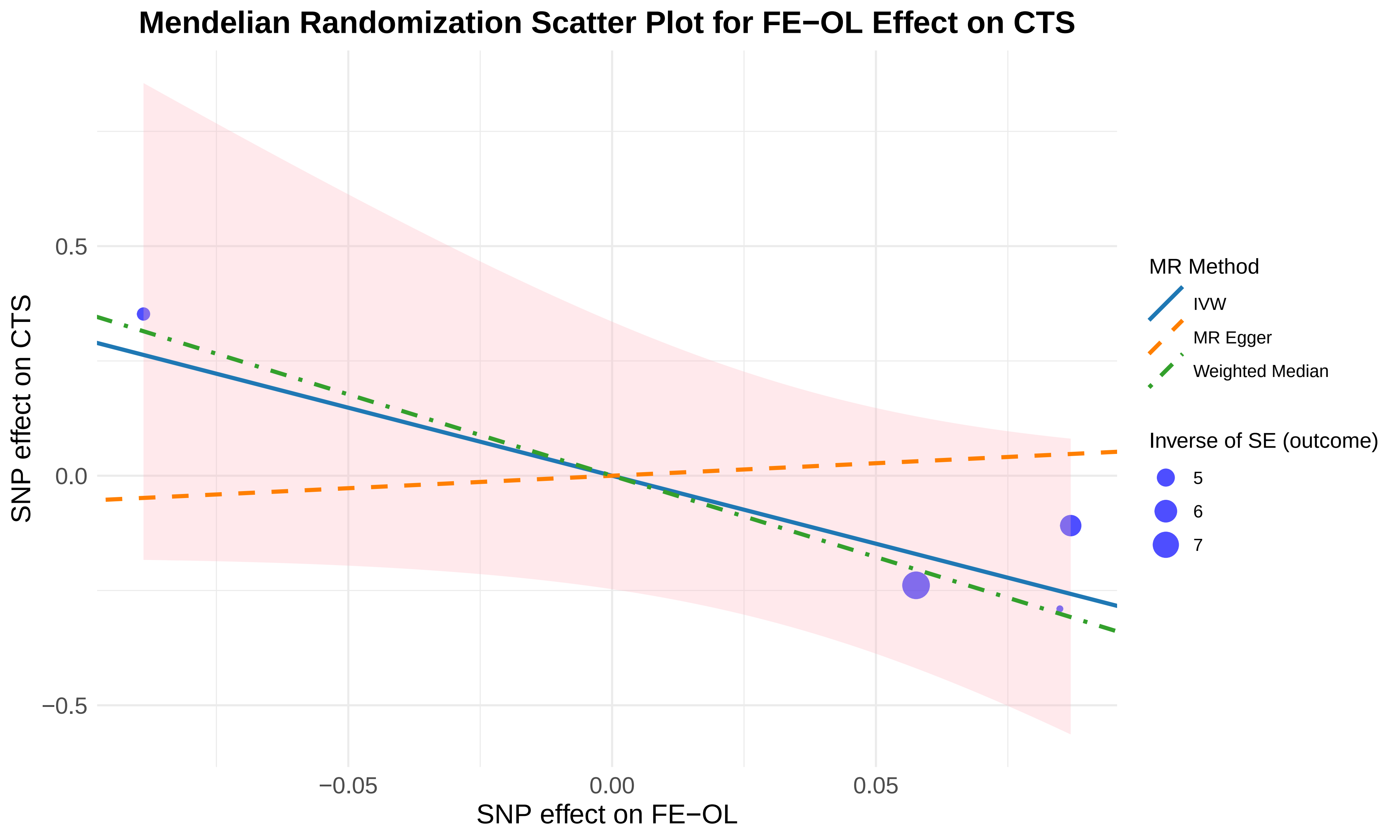

Mendelian Randomization Scatter Plot for FE−OL Effect on CTS
SNP effect on CTS
0.5
0.0
−0.5
−0.05
0.00
0.05
SNP effect on FE−OL
MR Method
IVW
MR Egger
Weighted Median
Inverse of SE (outcome)
5
6
7

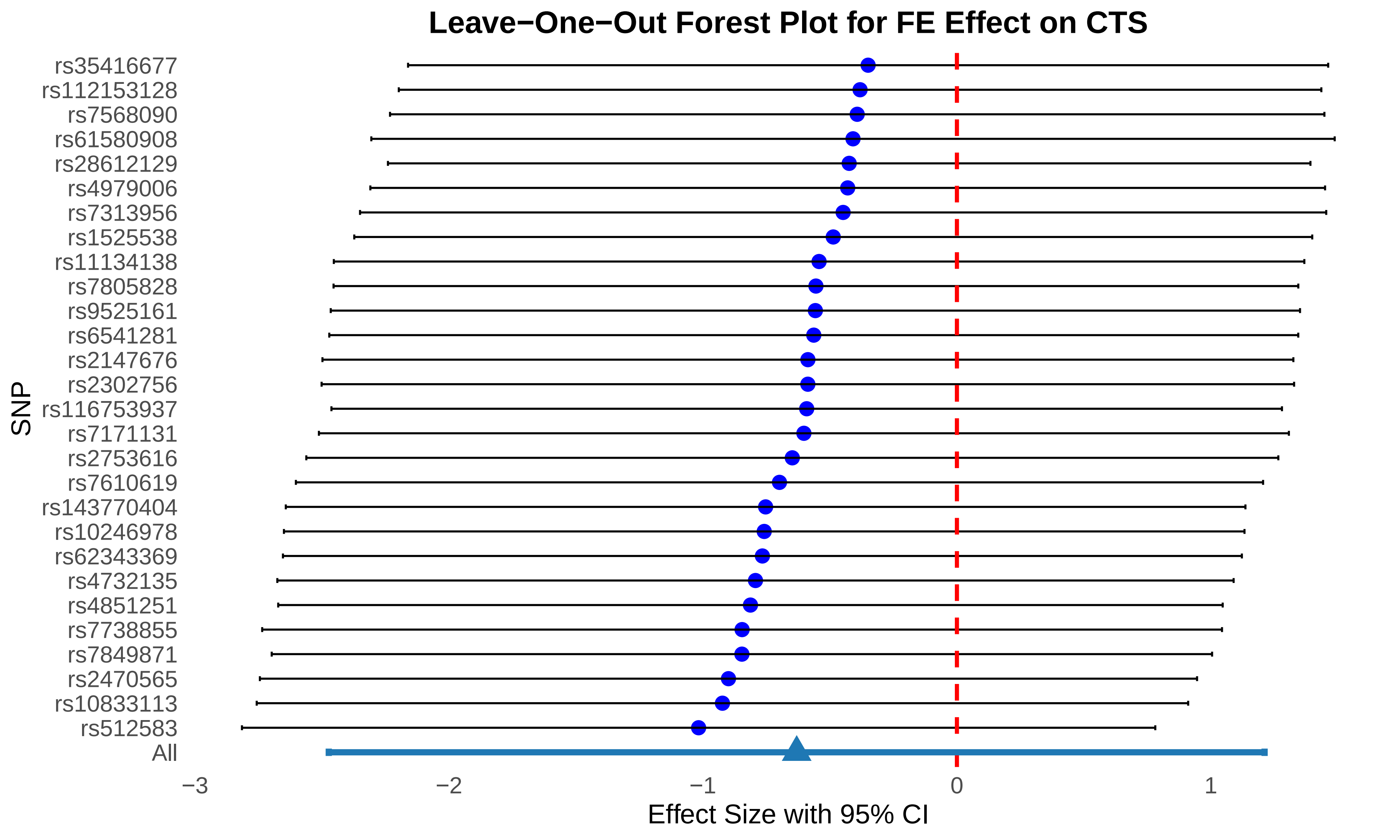
Leave−One−Out Forest Plot for FE Effect on CTS
SNP
rs35416677
rs112153128
rs7568090
rs61580908
rs28612129
rs4979006
rs7313956
rs1525538
rs11134138
rs7805828
rs9525161
rs6541281
rs2147676
rs2302756
rs116753937
rs7171131
rs2753616
rs7610619
rs143770404
rs10246978
rs62343369
rs4732135
rs4851251
rs7738855
rs7849871
rs2470565
rs10833113
rs512583
All
−3
−2
−1
0
1
Effect Size with 95% CI

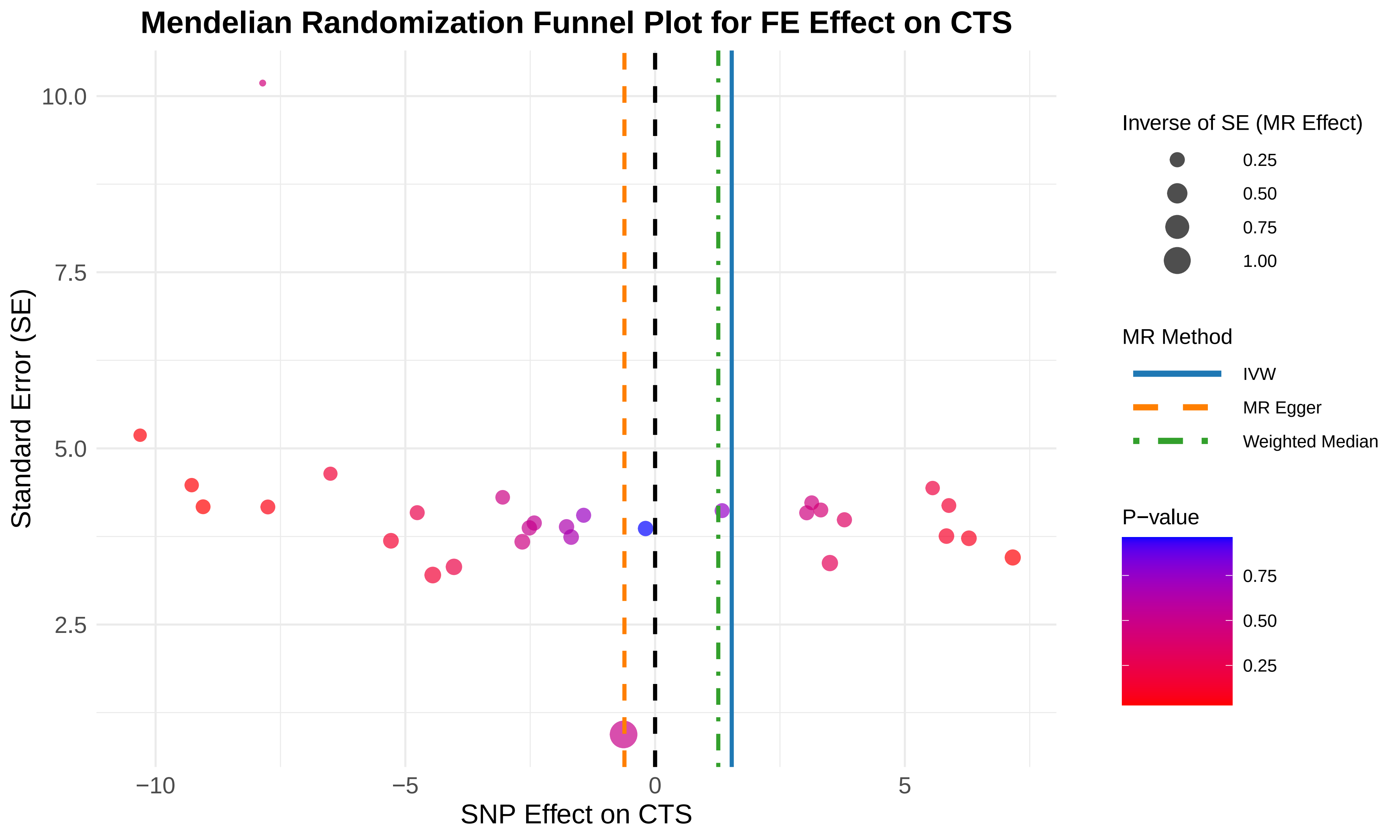
Mendelian Randomization Funnel Plot for FE Effect on CTS
Standard Error (SE)
SNP Effect on CTS
10.0
7.5
5.0
2.5
−10
−5
0
5
Inverse of SE (MR Effect)
0.25
0.50
0.75
1.00
MR Method
IVW
MR Egger
Weighted Median
P−value
0.75
0.50
0.25

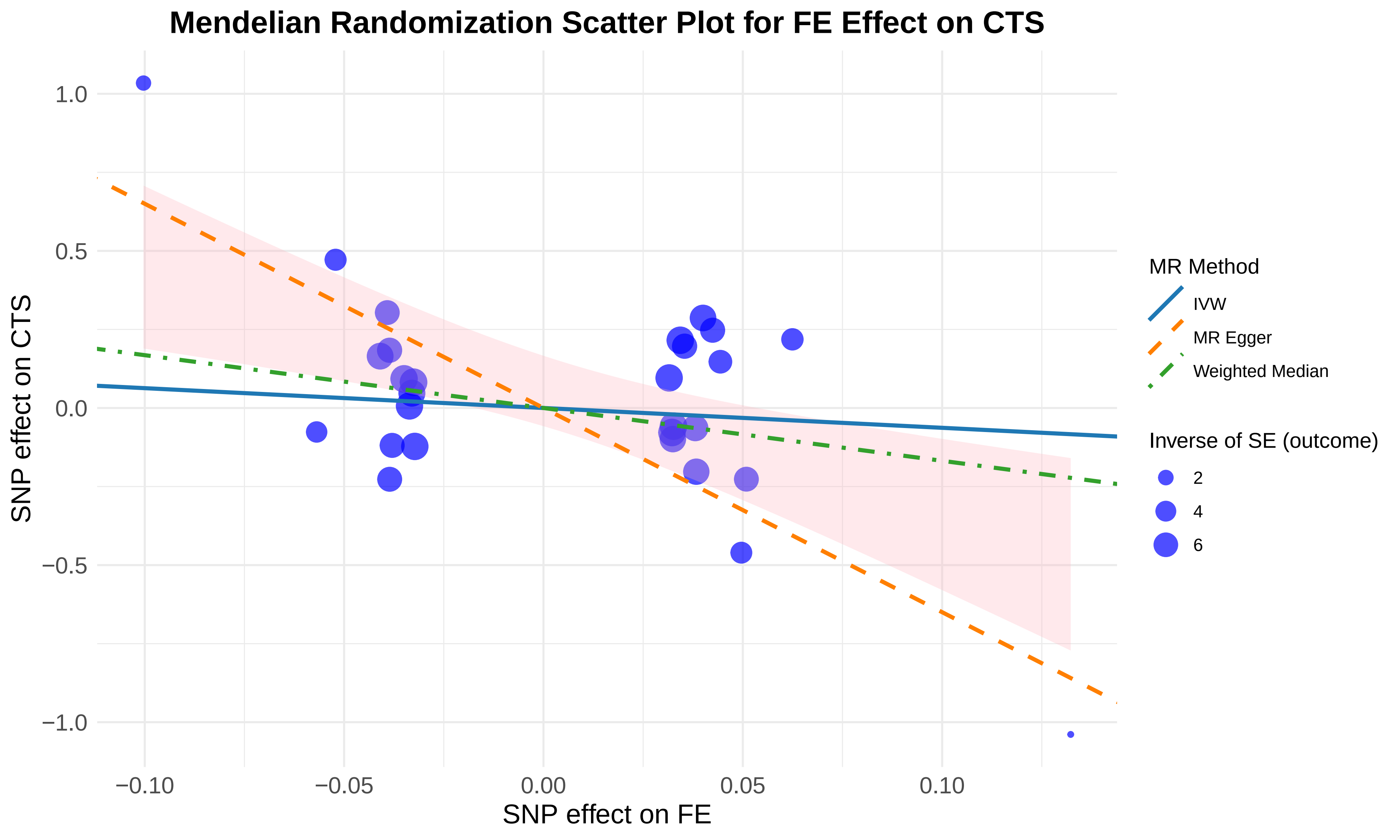
Mendelian Randomization Scatter Plot for FE Effect on CTS
SNP effect on CTS
SNP effect on FE
1.0
0.5
0.0
−0.5
−1.0
−0.10
−0.05
0.00
0.05
0.10
MR Method
IVW
MR Egger
Weighted Median
Inverse of SE (outcome)
2
4
6

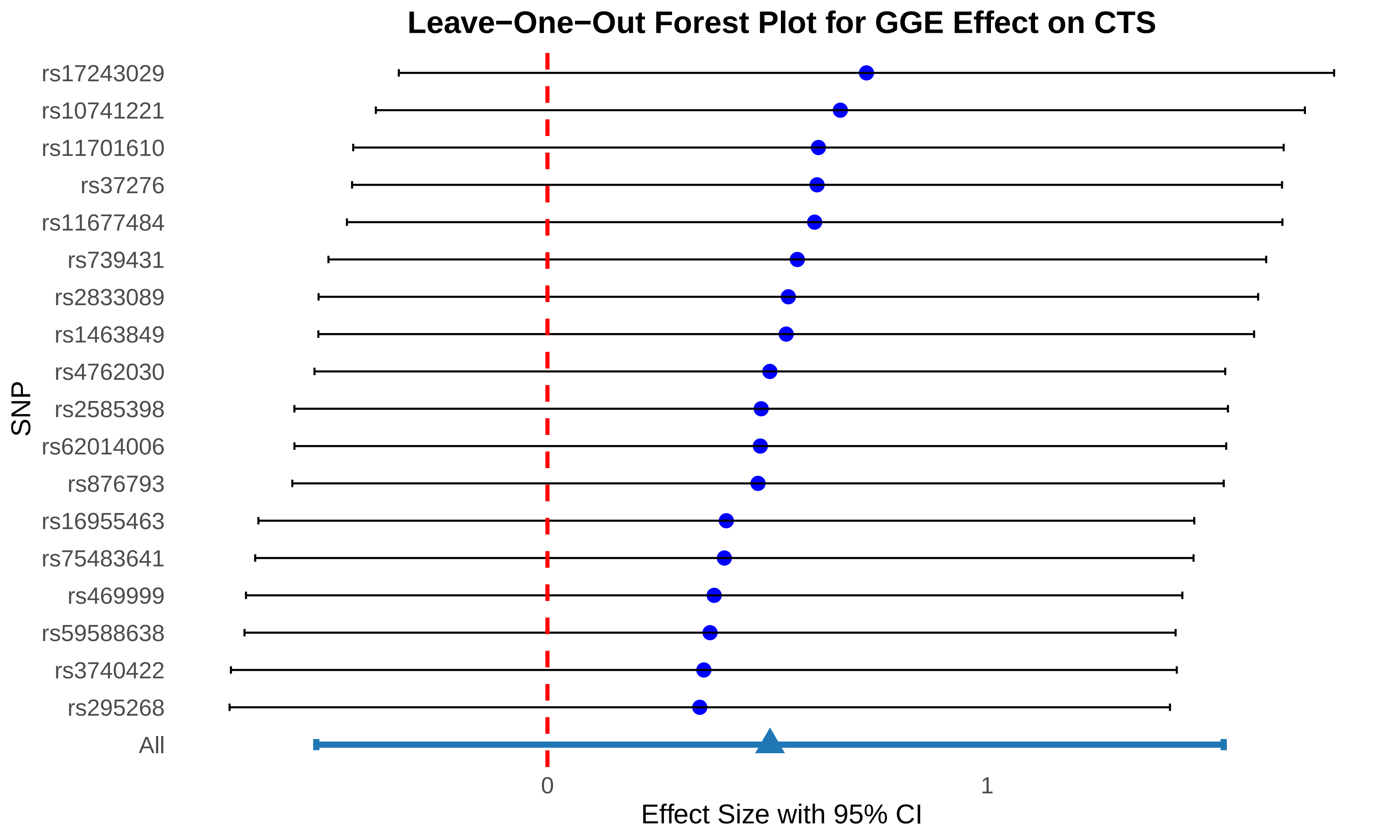
Leave−One−Out Forest Plot for GGE Effect on CTS
SNP
rs17243029
rs10741221
rs11701610
rs37276
rs11677484
rs739431
rs2833089
rs1463849
rs4762030
rs2585398
rs62014006
rs876793
rs16955463
rs75483641
rs469999
rs59588638
rs3740422
rs295268
All
0
1
Effect Size with 95% CI

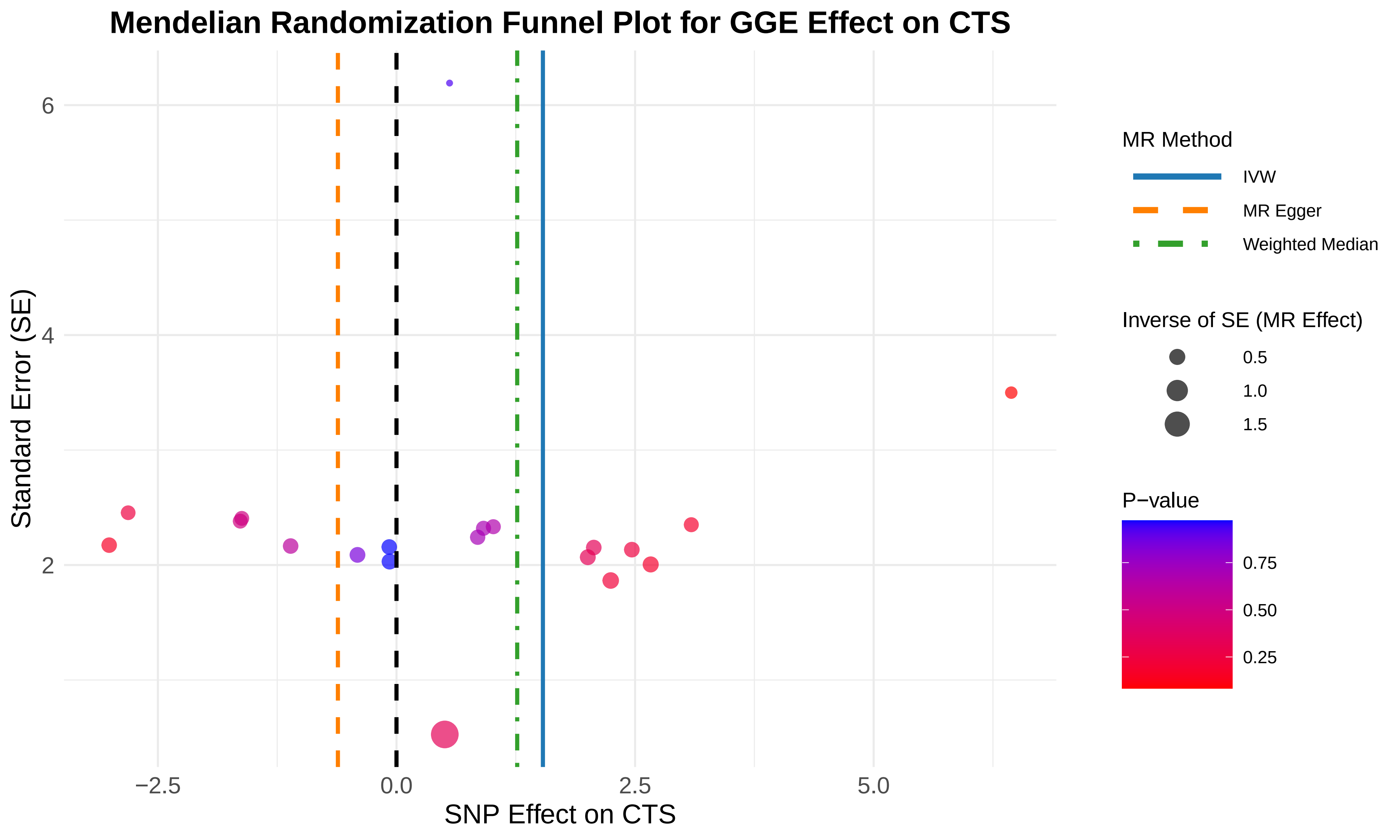
Mendelian Randomization Funnel Plot for GGE Effect on CTS
Standard Error (SE)
6
4
2
−2.5
0.0
2.5
5.0
SNP Effect on CTS
MR Method
IVW
MR Egger
Weighted Median
Inverse of SE (MR Effect)
0.5
1.0
1.5
P−value
0.75
0.50
0.25

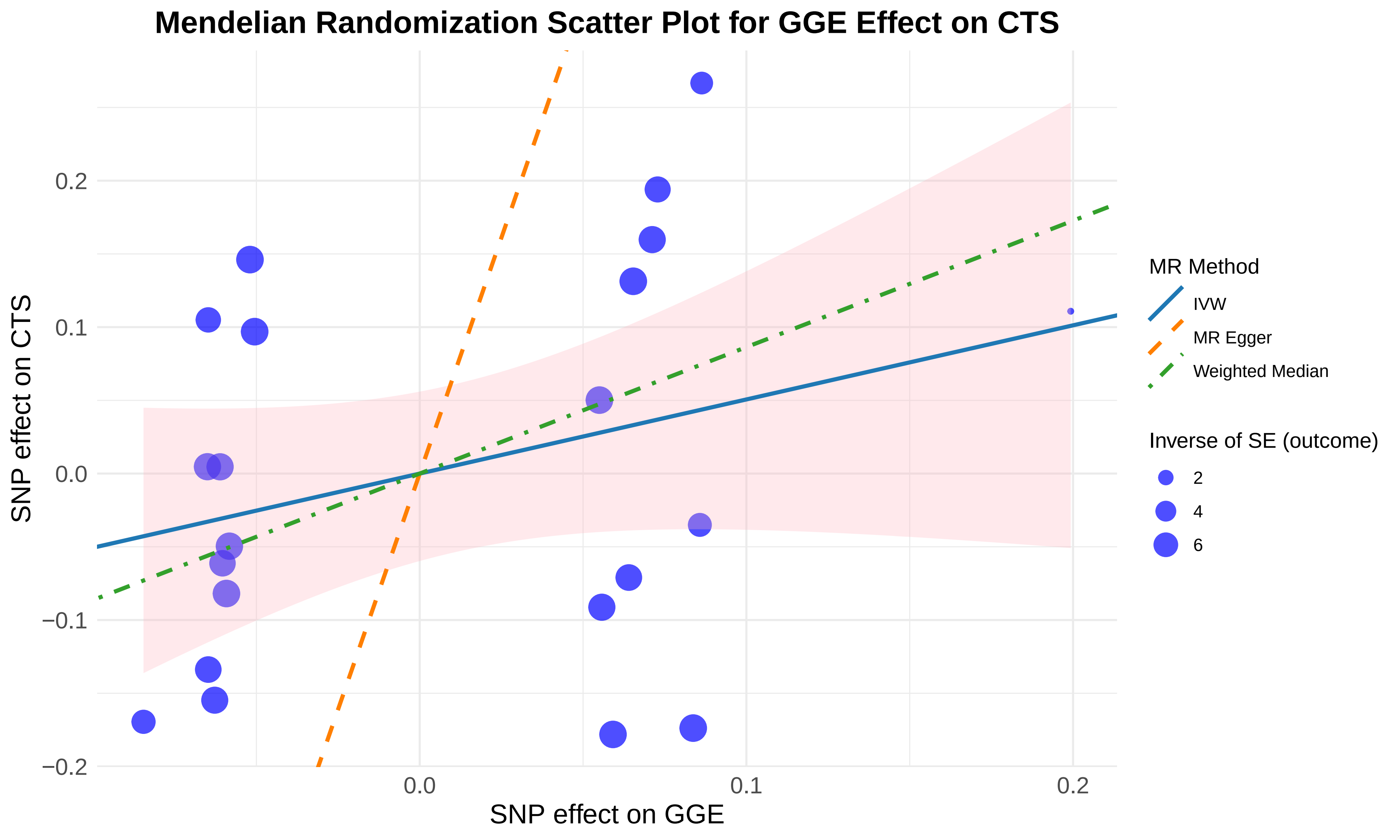

Mendelian Randomization Scatter Plot for GGE Effect on CTS
SNP effect on CTS
SNP effect on GGE
0.2
0.1
0.0
−0.1
−0.2
0.0
0.1
0.2
MR Method
IVW
MR Egger
Weighted Median
Inverse of SE (outcome)
2
4
6

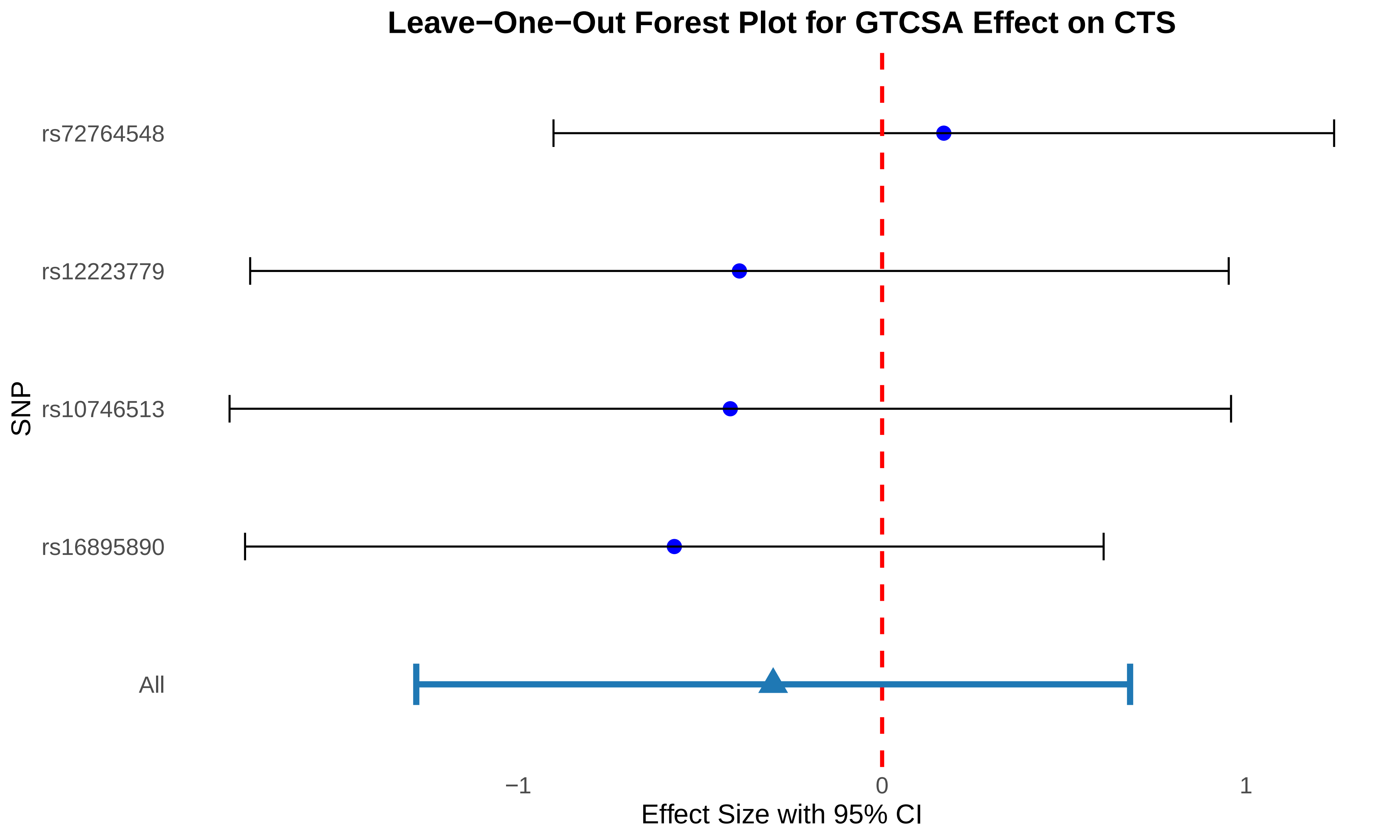

Leave−One−Out Forest Plot for GTCSA Effect on CTS
rs72764548
rs12223779
rs10746513
rs16895890
All
SNP
−1
0
1
Effect Size with 95% CI

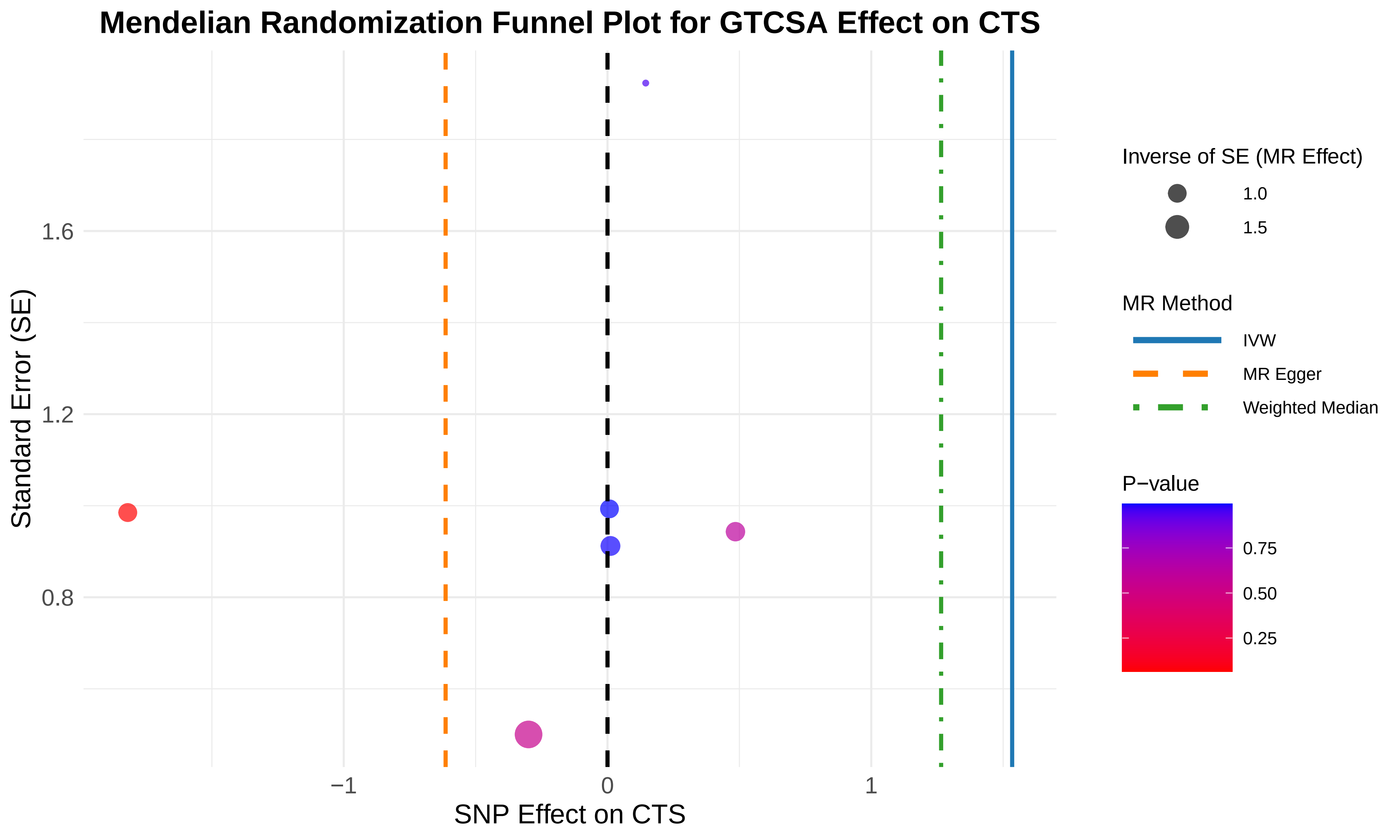

Mendelian Randomization Funnel Plot for GTCSA Effect on CTS
Standard Error (SE)
1.6
1.2
0.8
−1
0
1
SNP Effect on CTS
Inverse of SE (MR Effect)
1.0
1.5
MR Method
IVW
MR Egger
Weighted Median
P−value
0.75
0.50
0.25

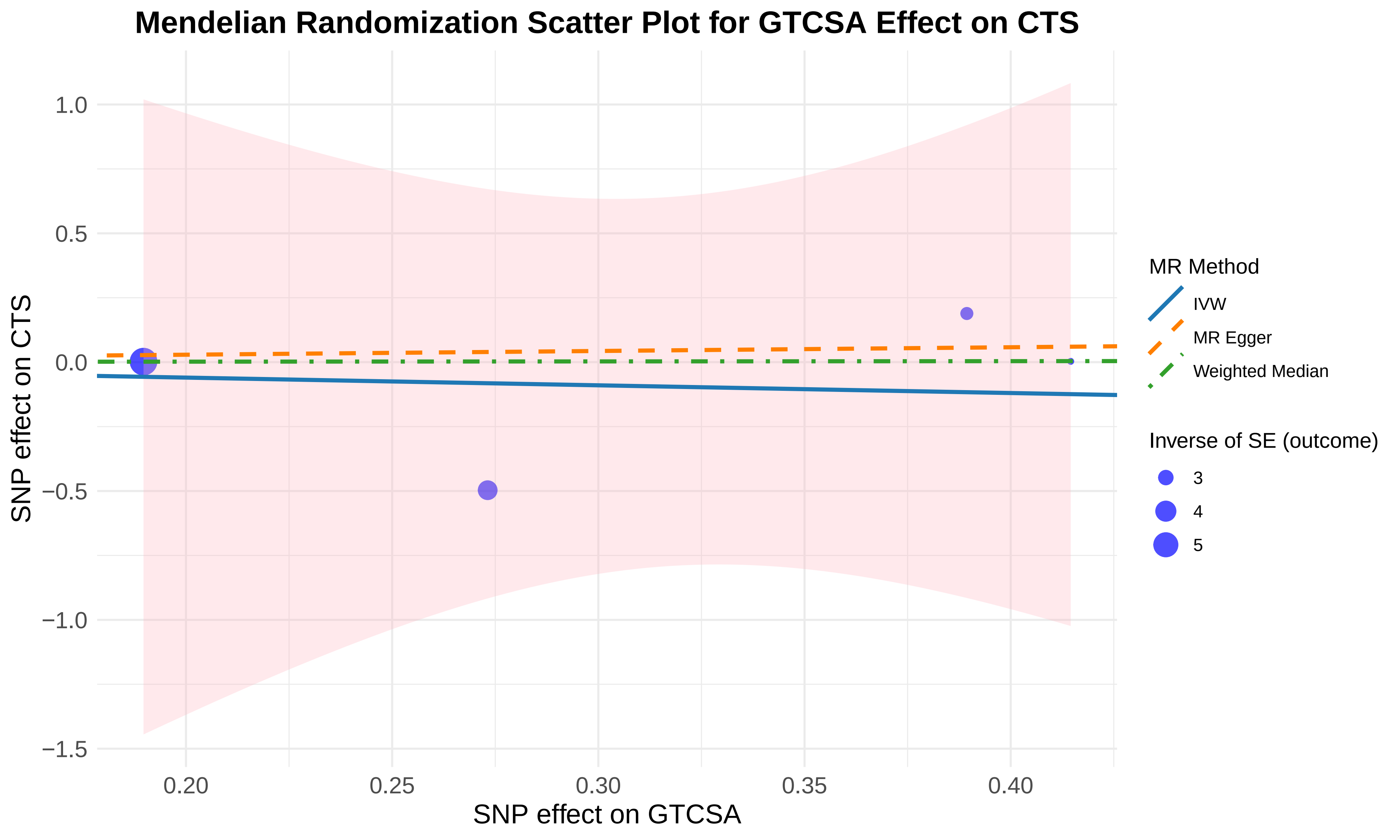

Mendelian Randomization Scatter Plot for GTCSA Effect on CTS
1.0
0.5
0.0
−0.5
−1.0
−1.5
SNP effect on CTS
0.20
0.25
0.30
0.35
0.40
SNP effect on GTCSA
MR Method
IVW
MR Egger
Weighted Median
Inverse of SE (outcome)
3
4
5

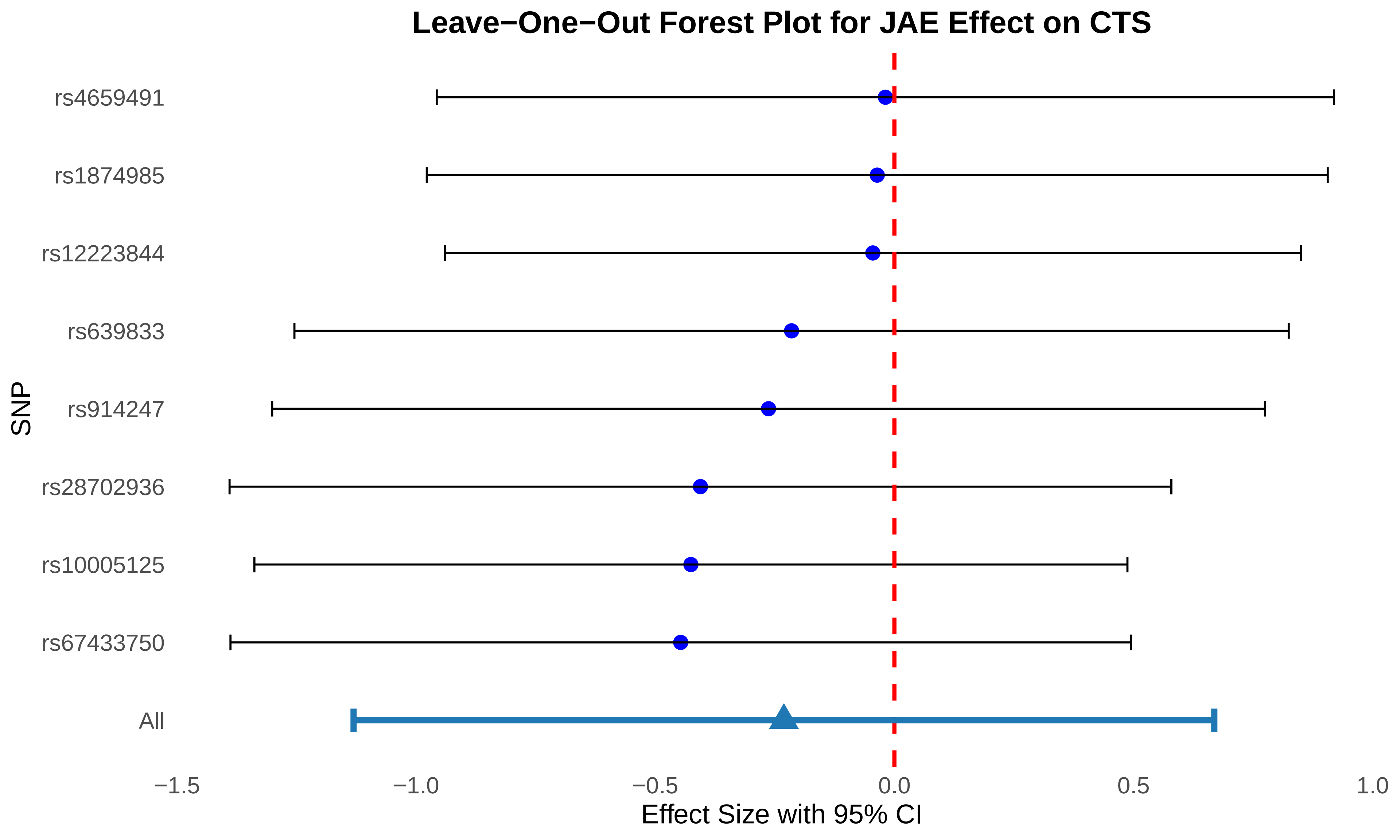

Leave−One−Out Forest Plot for JAE Effect on CTS
rs4659491
rs1874985
rs12223844
rs639833
rs914247
rs28702936
rs10005125
rs67433750
All
SNP
−1.5
−1.0
−0.5
0.0
0.5
1.0
Effect Size with 95% CI

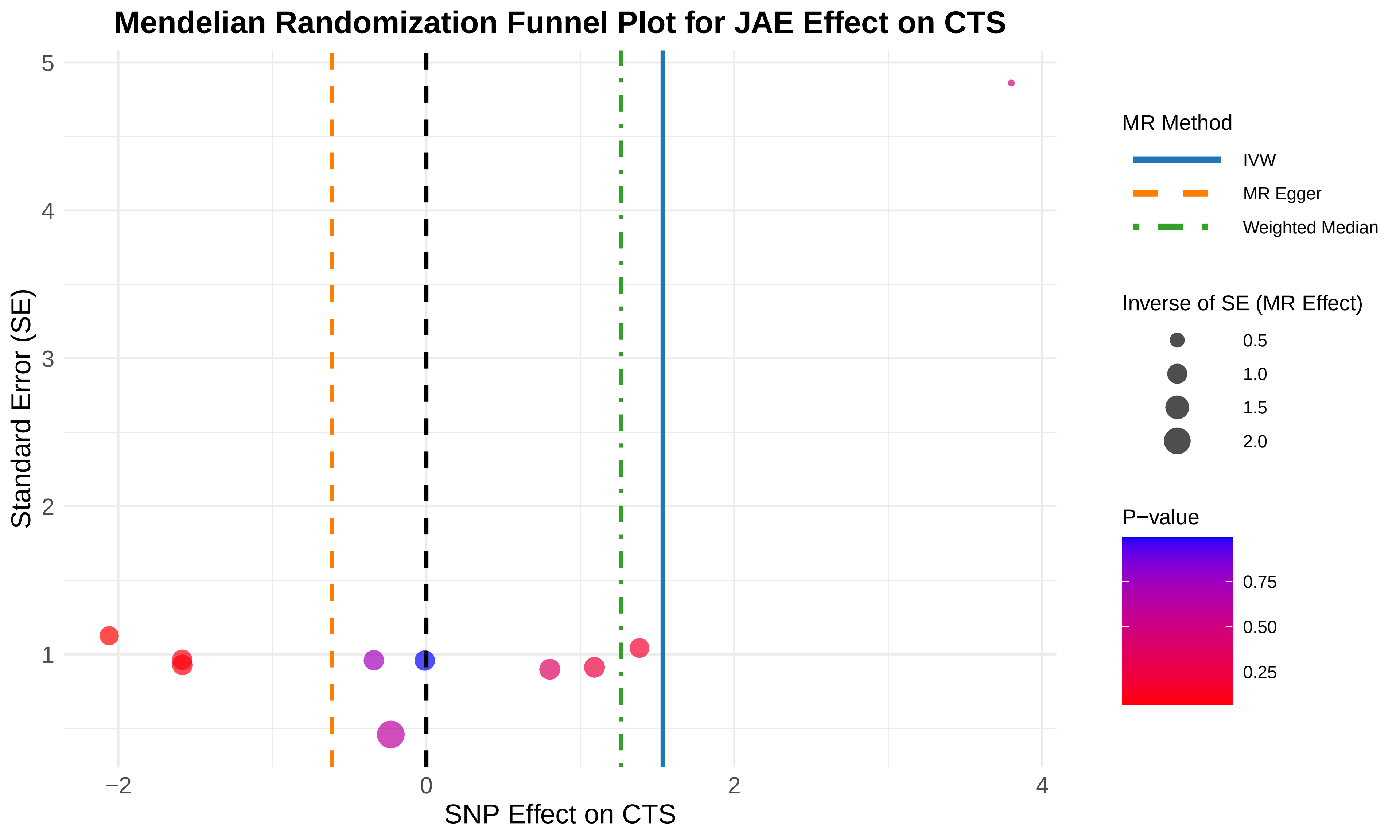
Mendelian Randomization Funnel Plot for JAE Effect on CTS
Standard Error (SE)
SNP Effect on CTS
5
4
3
2
1
−2
0
2
4
MR Method
IVW
MR Egger
Weighted Median
Inverse of SE (MR Effect)
0.5
1.0
1.5
2.0
P−value
0.75
0.50
0.25

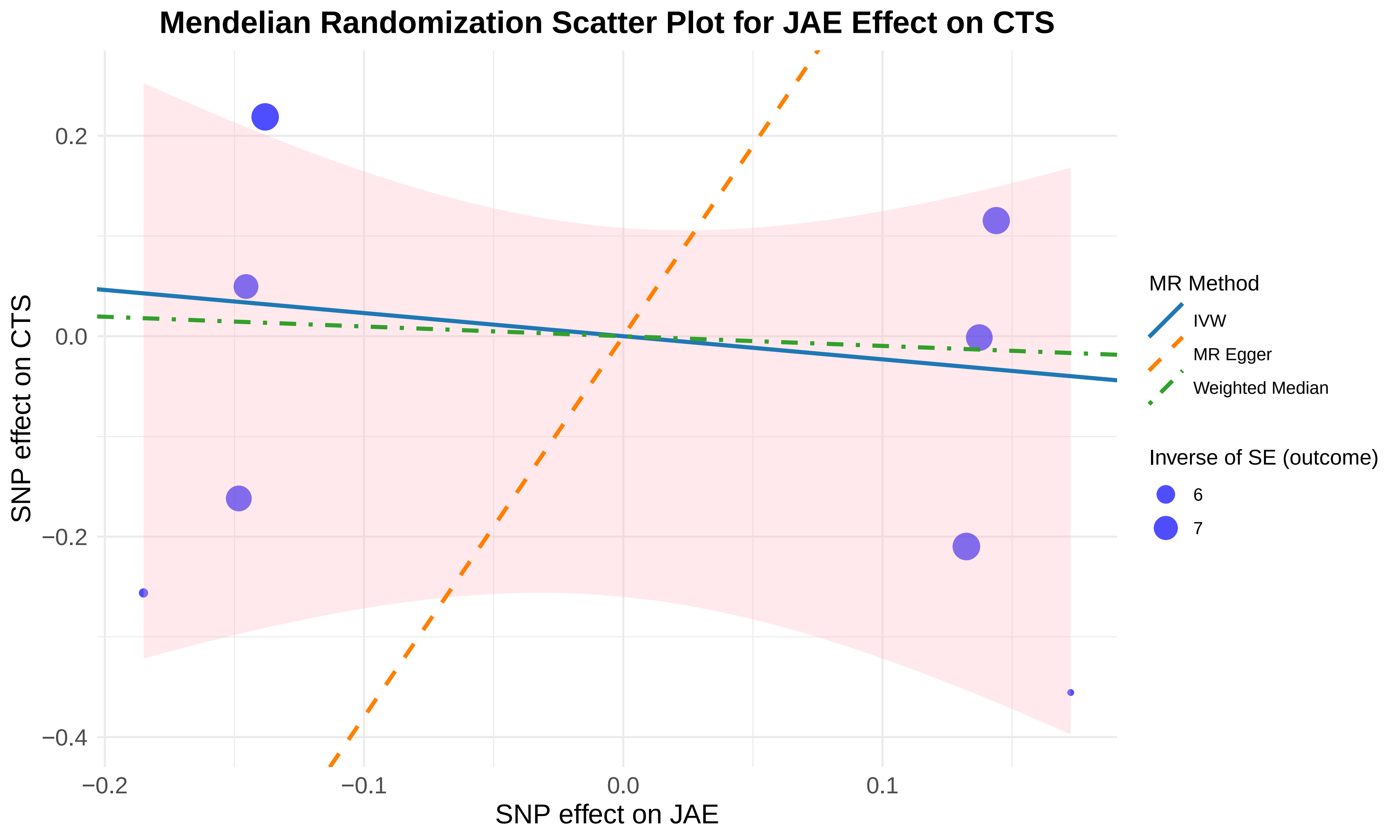
Mendelian Randomization Scatter Plot for JAE Effect on CTS
SNP effect on CTS
SNP effect on JAE
0.2
0.0
−0.2
−0.4
−0.2
−0.1
0.0
0.1
MR Method
IVW
MR Egger
Weighted Median
Inverse of SE (outcome)
6
7

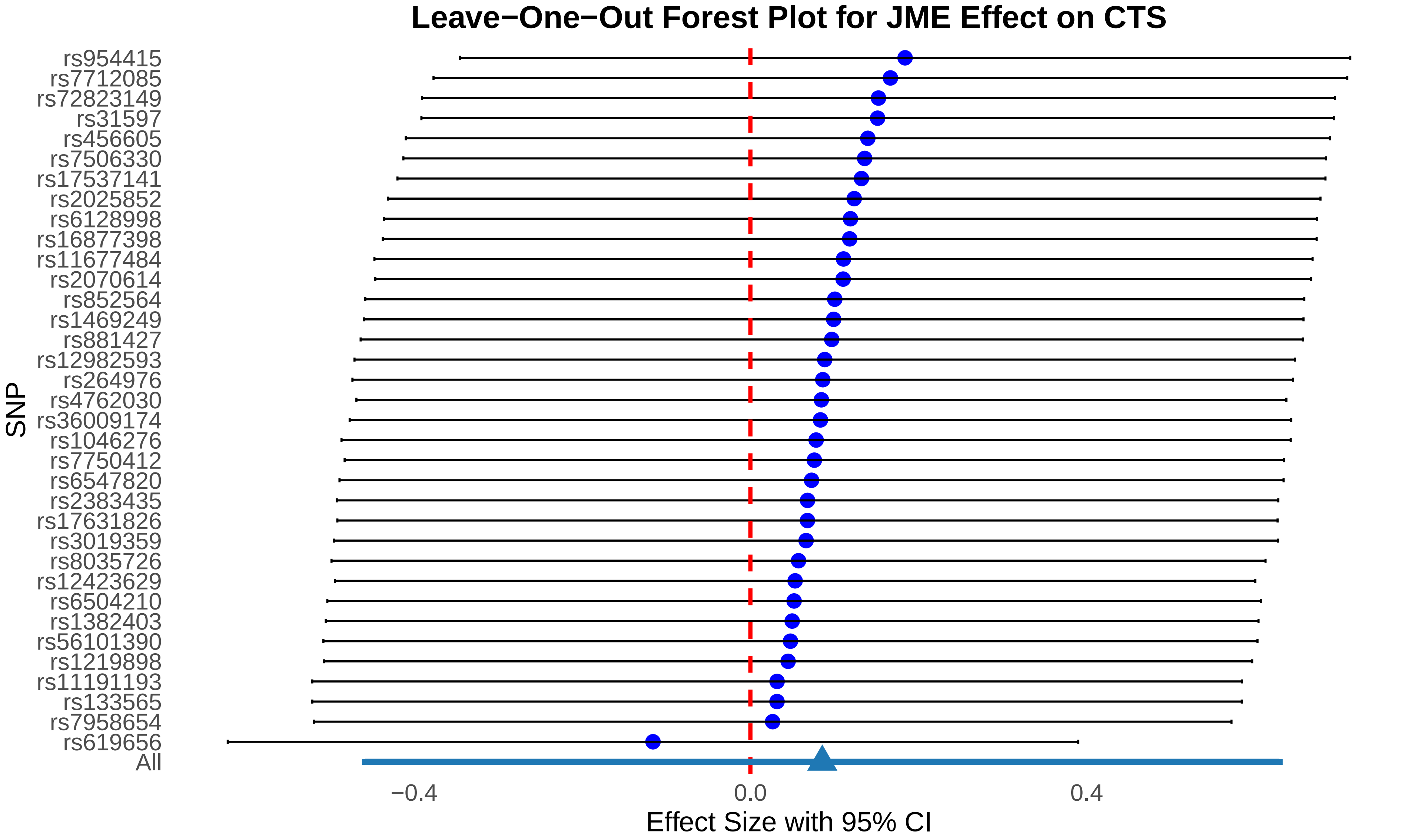
Leave−One−Out Forest Plot for JME Effect on CTS
SNP
rs954415
rs7712085
rs72823149
rs31597
rs456605
rs7506330
rs17537141
rs2025852
rs6128998
rs16877398
rs11677484
rs2070614
rs852564
rs1469249
rs881427
rs12982593
rs264976
rs4762030
rs36009174
rs1046276
rs7750412
rs6547820
rs2383435
rs17631826
rs3019359
rs8035726
rs12423629
rs6504210
rs1382403
rs56101390
rs1219898
rs11191193
rs133565
rs7958654
rs619656
All
−0.4
0.0
0.4
Effect Size with 95% CI

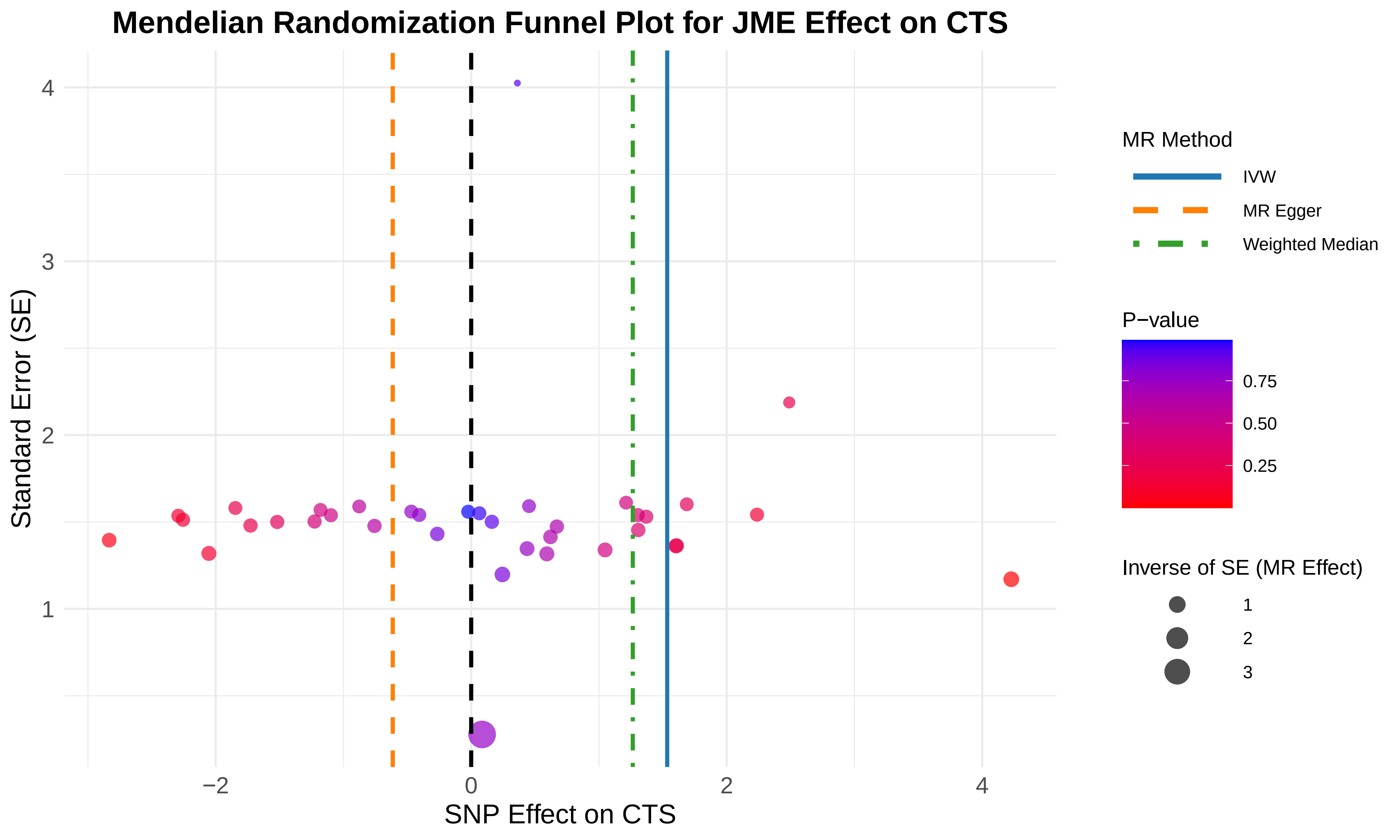
Mendelian Randomization Funnel Plot for JME Effect on CTS
Standard Error (SE)
SNP Effect on CTS
4
3
2
1
−2
0
2
4
MR Method
IVW
MR Egger
Weighted Median
P−value
0.75
0.50
0.25
Inverse of SE (MR Effect)
1
2
3

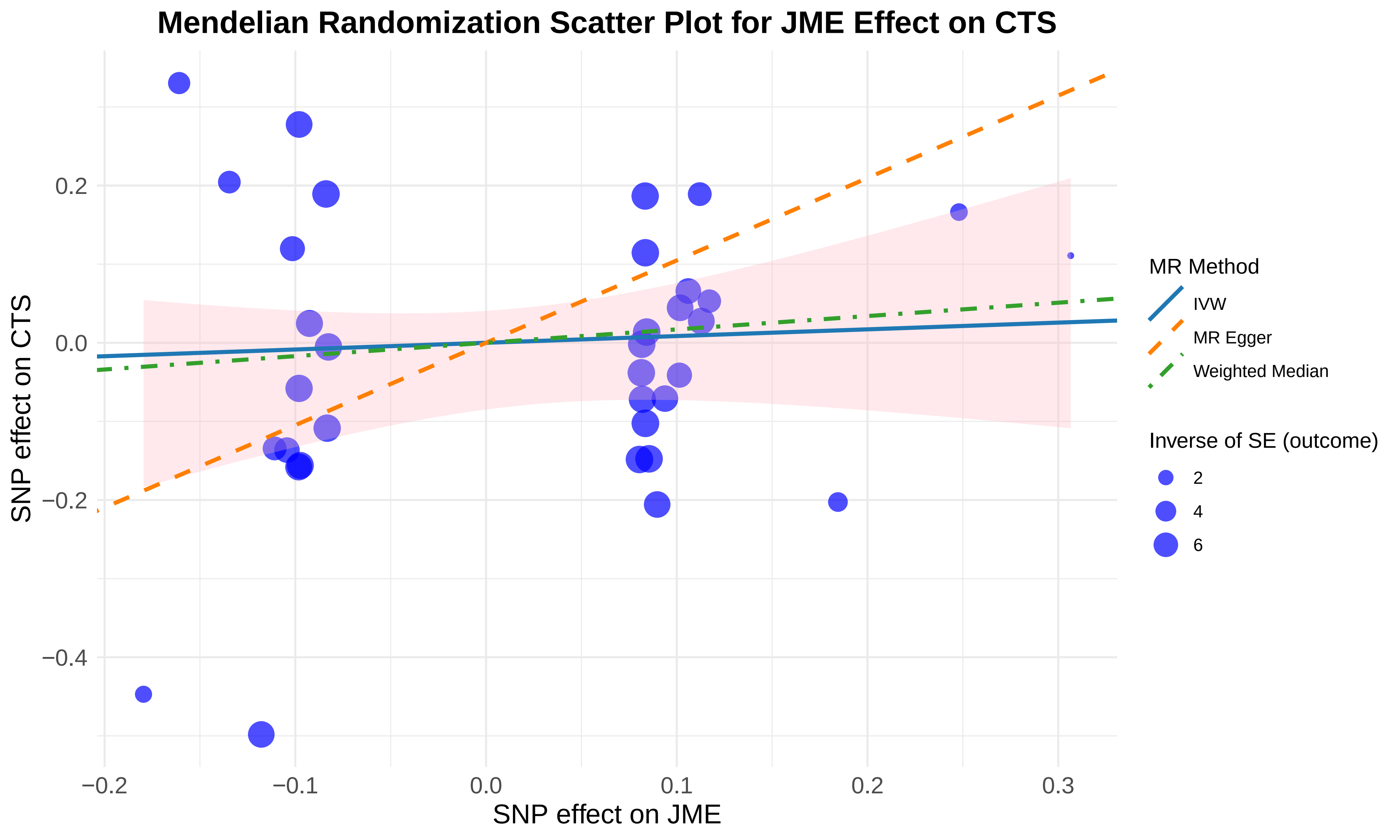

Mendelian Randomization Scatter Plot for JME Effect on CTS
SNP effect on CTS
SNP effect on JME
0.2
0.0
−0.2
−0.4
−0.2
−0.1
0.0
0.1
0.2
0.3
MR Method
IVW
MR Egger
Weighted Median
Inverse of SE (outcome)
2
4
6
